# Personalized MASLD and liver fibrosis risk assessment for adults: glycemic status determines optimal choice of non-invasive indices

**DOI:** 10.1186/s13098-025-02069-w

**Published:** 2025-12-22

**Authors:** Yuan Zhao, Dongyu Hu, Jiacheng Cheng, Huili Cao, Xiaojuan Wang, Junhua He, Yikun Zhu, Jin Li

**Affiliations:** 1 Department of Endocrinology, The Second Clinical Medical College of Shanxi Medical University, 382 Wuyi Road, Xinghualing, Taiyuan, 030001 China; 2 Department of Cardiology, The Second Clinical Medical College of Shanxi Medical University, Taiyuan, China

**Keywords:** Metabolic dysfunction-associated steatotic liver diseas, Liver fibrosis, Prediabetes, Diabetes, Non-invasive indices

## Abstract

**Background:**

Metabolic dysfunction–associated steatotic liver disease (MASLD) is a global health challenge, with early detection and risk stratification across glycemic states still unresolved. This study aimed to evaluate the performance of 18 non-invasive metabolic indices in predicting MASLD (defined as hepatic steatosis with metabolic risk factors) and significant fibrosis across glycemic states and demographic subgroups.

**Methods:**

Cross-sectional analyses were performed on 2,794 individuals aged ≥ 20 years from the 2017–2020 National Health and Nutrition Examination Survey cycle, stratified by glycemic status and demographic factors. Eighteen indices were assessed: triglyceride-glucose index (TyG), TyG-body mass index (TyG-BMI), TyG-waist circumference (TyG-WC), TyG-waist-to-height ratio (TyG-WHtR), TyG-weight-adjusted waist index (TyG-WWI), visceral adiposity index (VAI), homeostatic model assessment of insulin resistance (HOMA-IR), metabolic score for IR (METS-IR), Framingham steatosis index (FSI), fatty liver index (FLI), USFLI, Zhejiang University index (ZJU), lipid accumulation product (LAP), hepatic steatosis index (HSI), non-high-density lipoprotein cholesterol (HDL-C) to HDL-C ratio (NHHR), Nonalcoholic fatty liver disease fibrosis score (NFS), fibrosis-4 index (FIB-4), and BMI-aspartate aminotransferase/alanine aminotransferase ratio and diabetes score (BARD). Associations and diagnostic performance were examined using multivariable logistic regression and receiver operating characteristic analyses.

**Results:**

MASLD prevalence increased with worsening glycemic status: 40.6% in normoglycemia, 62.7% in prediabetes, and 85.5% in type 2 diabetes mellitus (T2DM). TyG-WHtR exhibited the strongest association with MASLD (odds ratio [OR] 3.83), with an even higher risk in T2DM (OR 5.58). FLI, TyG-WHtR, and NFS were associated with fibrosis, though the associations were weaker in normoglycemia. TyG-WC demonstrated the highest diagnostic accuracy for MASLD (area under the curve [AUC] 0.830), particularly in males and adults ≤ 50 years (AUCs 0.842–0.853). FLI performed consistently across glycemic strata (AUCs 0.769–0.821). FSI performed well in T2DM (AUC 0.823) and in males (AUC 0.829). For significant fibrosis, FLI showed the best performance, particularly in prediabetes (AUC 0.761) and T2DM (AUC 0.754). Traditional markers (FIB-4, BARD) demonstrated limited utility.

**Conclusions:**

Glycemic deterioration is strongly linked to MASLD and fibrosis. TyG-WC and FLI were the most consistent and high-performing indices across glycemic and demographic groups, supporting their use as broadly applicable non-invasive tools for individualized MASLD risk assessment.

**Supplementary Information:**

The online version contains supplementary material available at 10.1186/s13098-025-02069-w.

## Introduction

Metabolic dysfunction-associated steatotic liver disease (MASLD), formerly known as nonalcoholic fatty liver disease (NAFLD), has emerged as the leading cause of chronic liver disease worldwide [1]. MASLD encompasses a broad spectrum of liver pathologies, ranging from simple steatosis to steatohepatitis, fibrosis, and cirrhos [2]. As its new nomenclature implies, MASLD is inherently connected to systemic metabolic disturbances, particularly those involving glucose metabolism. Notably, impaired glucose metabolism is a key driver of hepatic lipogenesis, oxidative stress, and inflammation, which accelerates MASLD progression and increases the risk of adverse outcomes, such as cirrhosis and hepatocellular carcinoma. [3, 4]. Given the significant impact of glycemic status on the development and severity of MASLD, stratifying patients based on their glycemic profiles is crucial for accurate risk assessment and personalized clinical management.

MASLD is diagnosed based on the presence of hepatic steatosis along with at least one cardiometabolic risk facto [2]. While serum enzymes, imaging, and biopsy are commonly used to detect liver fat and assess disease severity, serum transaminases often fail to identify MASLD in its early or metabolically silent stages [5]. Imaging modalities such as transient elastography and magnetic resonance-based methods offer better sensitivity but are costly and less accessible for widespread screening. While liver biopsy is considered the diagnostic gold standard, it is invasive and not suitable for routine use [5]. Consequently, non-invasive metabolic indices derived from routine clinical and biochemical parameters have gained attention as potential surrogate markers for MASLD risk assessment [6–8]. The triglyceride-glucose (TyG) index and its derivatives (TyG-waist circumference (WC), TyG-body mass index (BMI), TyG-waist-to-height ratio (WHtR) and TyG-weight-adjusted waist index (WWI)) integrate fasting triglyceride and glucose levels with anthropometric measures to reflect underlying insulin resistance (IR) and adiposity [9, 10]. Other indices, such as homeostasis model assessment of IR (HOMA-IR), and the metabolic score for IR (METS-IR), estimate IR based on insulin or glucose metrics [11], while steatosis-related indices (Framingham steatosis index (FSI), fatty liver index (FLI), USFLI, visceral adiposity index (VAI), lipid accumulation product (LAP), hepatic steatosis index (HSI), Zhejiang University index (ZJU), and non-high-density lipoprotein cholesterol (HDL-C) to HDL-C ratio (NHHR)) and fibrosis-predictive scores (NAFLD fibrosis score (NFS), fibrosis-4 index (FIB-4), BMI-aspartate aminotransferase/alanine aminotransferase ratio and diabetes score (BARD)) offer broader estimations of hepatic fat accumulation and fibrosis risk [12–19]. Although current evidence highlights the predictive potential of certain non-invasive indicators for assessing the risk of MASLD in individuals with diabetes [20, 21], two critical research gaps persist: first, the utility of various non-invasive indicators in prediabetic and diabetic populations has yet to be fully explored; and second, there is no systematic comparison of their performance in predicting liver fibrosis across different glycemic states (normoglycemia, prediabetes, and diabetes). Moreover, sociodemographic factors, including age, sex, race, and obesity, interact with glycemic status and are linked to variations in MASLD susceptibility [22], further reinforcing the need for stratified, population-specific assessment tools.

Therefore, this study comprehensively evaluated 18 metabolic indices for predicting MASLD and significant fibrosis in a nationally representative US cohort, stratified by glycemic status and key demographic factors. Our study aims to identify robust, non-invasive biomarkers tailored to distinct metabolic profiles, facilitating precision screening and risk stratification. By optimizing biomarker selection, these findings will enhance early detection and support personalized intervention strategies for this high-burden disease.

## Methods

### Study population

This study conducted a cross-sectional analysis using data from the National Health and Nutrition Examination Survey (NHANES) database, covering the years 2017 to 2020. The NHANES survey employed a stratified, multistage probability cluster design to ensure that the sample population was nationally representative. Initially, data from 15,560 participants were obtained from the 2017–2020 NHANES cycles. Participants aged ≥ 20 years with complete FibroScan and metabolic data were included. Exclusion criteria included: positivity for viral hepatitis B or C (n = 220), heavy alcohol consumption (n = 1,325), missing FibroScan data (n = 1,310), and missing values for calculating the 18 metabolic indices (n = 3,583). Finally, 2,794 participants were included in this study (Fig. S1).

### Laboratory measurement and clinical data

Demographic information, physical measurements, and laboratory test values were obtained from the NHANES dataset and described in the Supplementary Methods. TyG, TyG-WC, TyG-BMI, TyG-WHtR, TyG-WWI, BARD, VAI, HOMA-IR, METS-IR, FSI, FLI, USFLI, ZJU, LAP, HSI, NFS, FIB-4, and NHHR were calculated according to the formulas in the Supplementary Methods.

### Definitions

The severity of liver steatosis in the NHANES cohort was assessed by the controlled attenuation parameter (CAP) derived from FibroScan®, with a criterion of CAP ≥ 248 dB/m [23]. According to the recent Delphi consensus statement [2], MASLD is defined as patients with hepatic steatosis and at least one of the five cardiovascular metabolic risk factors, including (1) Overweight or obesity: BMI ≥ 25 kg/m^2^ (23 Asia) or WC ≥ 94 cm for men and ≥ 80 cm for women; (2) diabetes or prediabetes: diabetes was diagnosed by a self-reported history of diabetes, use of oral hypoglycemic agents or insulin, fasting plasma glucose (FPG) ≥ 126 mg/dl, or HbA1c level ≥ 6.5%. Prediabetes was defined as an FPG between 100–125 mg/dL or HbA1c of 5.7%−6.4% without a diagnosis of diabetes or taking diabetes medications; (3) blood pressure ≥ 130/85 mmHg or receiving specific antihypertensive treatment; (4) plasma triglycerides (TG) ≥ 1.70 mmol/L or undergoing lipid-lowering therapy. Diabetes and prediabetes were defined based on a single measurement of FPG or HbA1c, in accordance with NHANES protocols. The use of a single measurement, rather than repeated tests or oral glucose tolerance tests, may lead to misclassification, which is acknowledged as a limitation; (5) plasma HDL-C ≤ 1.0 mmol/L (men) and ≤ 1.3 mmol/L (women), or receiving lipid-lowering therapy. A median liver stiffness measurement (LSM) ≥ 8.2 kPa, ≥ 9.7 kPa, and ≥ 13.6 kPa indicated significant fibrosis (≥ F2), advanced fibrosis (≥ F3), and cirrhosis (F4), respectively [24]. Hypertension was defined as systolic blood pressure ≥ 140 mmHg or diastolic blood pressure ≥ 90 mmHg or self-reported use of antihypertensive medications [25].

### Statistical analysis

Statistical analyses were performed according to NHANES guidelines using R software (version 4.4.3) with the packages pROC (v1.18.5), nhanesR, and survey (v4.2–1). Missing values were addressed using complete-case analysis. Normality of continuous variables was assessed with the Shapiro–Wilk test. Since continuous variables were non-normally distributed, they are presented as median (interquartile range). Qualitative variables are expressed as counts (percentages) and were compared using the χ^2^ test. Multivariate logistic regression models were adjusted for age, sex, poverty income ratio, education level, smoking status, race, hypertension, and obesity. Results are presented as odds ratios (OR) with 95% confidence intervals (CI). To account for multiple testing across metabolic indices and subgroups, the Benjamini–Hochberg false discovery rate (FDR) procedure was applied. The predictive value of the 18 indices for MASLD was assessed using receiver operating characteristic (ROC) curves and the area under the curve (AUC). Statistical significance was defined as a two-tailed p-value < 0.05.

## Results

### Clinical baseline characteristics

A total of 2,794 participants were included in this study, with population characteristics stratified by glycemic status and the presence of MASLD (Table 1). Of these, 1,476 (51.1%) were female, and 1,318 (48.9%) were male, with a median age of 50 years. Ethnic distribution revealed non-Hispanic Whites as the largest group (63.6%), followed by non-Hispanic Blacks (10.8%), Mexican Americans (7.9%), and other racial/ethnic minorities. Participants with T2DM and prediabetes showed progressively worse metabolic profiles compared to normoglycemic individuals, including elevated BMI, WC, FPG, HbA1c, and TG levels, along with reduced HDL-C. When further stratified by MASLD status, normoglycemic individuals with MASLD exhibited significant metabolic disturbances compared to their non-MASLD counterparts, including elevated metabolic indices and higher obesity prevalence. The metabolic deterioration was more pronounced in prediabetic subjects with MASLD, demonstrating exacerbated IR, dyslipidemia, and central adiposity. Notably, the diabetic group with MASLD exhibited the most severe metabolic and hepatic dysfunction, with the highest FPG, HbA1c, and 18 metabolic indices, along with a markedly elevated comorbidity burden. These findings underscore that metabolic abnormalities worsened progressively from normoglycemia to prediabetes and diabetes, with the presence of MASLD corresponding to more pronounced disturbances across all glycemic states.

**Table 1:** Baseline demographic and clinical characteristics of the study population, stratified by glycemic status and MASLD

Variables	No. (weighted %)							P-value
	Total (n = 2794)	Normoglycemia (n = 958)		Prediabetes (n = 1168)		T2DM (n = 668)		
		Without MASLD (n = 590)	With MASLD (n = 368)	Without MASLD (n = 424)	With MASLD (n = 744)	Without MASLD (n = 118)	With MASLD (n = 550)	
Age, median (IQR), years	50.0(34.0,63.0)	35.0(27.0,51.0)	43.0(32.0,61.0)	45.0(33.0,61.0)	55.0(41.0,65.0)	68.0(51.0,74.0)	62.0(53.0,70.0)	< 0.0001
Sex								< 0.0001
Female	1476(51.1)	372(63.1)	218(60.0)	213(48.3)	349(40.8)	58(46.3)	266(49.0)	
Male	1318(48.9)	218(36.9)	150(40.0)	211(51.7)	395(59.2)	60(53.7)	284(51.0)	
Race								< 0.001
Mexican American	330(7.9)	46(5.9)	48(9.9)	48(7.9)	95(7.6)	11(7.1)	82(10.0)	
Non-Hispanic Black	699(10.8)	188(15.0)	101(11.7)	109(10.4)	132(6.5)	46(22.5)	123(10.4)	
Non-Hispanic White	950(63.6)	192(62.1)	124(62.2)	127(61.1)	305(70.0)	24(49.1)	178(59.6)	
Other Hispanic	288(7.2)	57(7.4)	36(8.0)	41(7.1)	75(6.2)	13(8.5)	66(7.9)	
Other Race	527(10.5)	107(9.5)	59(8.2)	99(13.5)	137(9.6)	24(12.8)	101(12.1)	
Educational-level								0.002
Less than 9th grade	215(3.5)	25(1.9)	19(2.5)	30(3.4)	58(3.2)	17(8.3)	66(7.1)	
9-11th grade	300(7.4)	57(6.4)	28(3.9)	42(6.9)	81(7.8)	15(10.3)	77(11.8)	
High school graduate/GED or equivalent	631(24.6)	135(22.5)	77(23.0)	85(24.9)	175(23.3)	25(21.3)	134(31.9)	
Some college or AA degree	879(28.6)	194(30.1)	136(32.4)	132(27.3)	218(27.3)	32(19.7)	167(28.3)	
College graduate or above	767(35.8)	178(39.1)	108(38.3)	135(37.4)	211(38.3)	29(40.3)	106(21.0)	
Alcohol consumption								0.01
Mild	1176(47.4)	240(54.9)	148(51.6)	189(66.3)	337(65.7)	50(74.0)	212(63.2)	
Moderate	536(22.6)	148(34.9)	87(41.0)	80(25.9)	145(25.4)	16(16.3)	60(20.0)	
Never	284(7.5)	63(10.2)	36(7.4)	45(7.9)	76(8.9)	9(9.7)	55(16.8)	
Smoking status								0.1
Former	686(28.4)	104(23.7)	65(19.2)	89(26.6)	205(33.5)	33(33.7)	190(35.2)	
Never	1710(59.9)	385(65.2)	242(65.4)	267(61.4)	451(56.6)	66(51.0)	299(53.6)	
Now	396(11.7)	100(11.2)	61(15.5)	68(12.0)	88(9.9)	19(15.3)	60(11.2)	
PIR, median (IQR)	3.4(1.8,5.0)	3.7(1.7,5.0)	3.4(1.8,5.0)	3.7(2.1,5.0)	3.7(2.0,5.0)	3.3(1.9,5.0)	2.6(1.4,4.9)	0.03
BMI, median (IQR), kg.m2	28.3(24.5,32.8)	24.0(21.6,27.6)	29.6(26.4,34.8)	25.9(23.6,28.8)	30.4(26.6,34.7)	26.2(23.7,30.6)	32.4(28.7,37.3)	< 0.0001
WC, median (IQR), cm	98.3(87.8,110.6)	85.7(78.1, 94.2)	101.8(92.7,112.4)	92.5(85.4, 99.3)	104.3(94.8,114.7)	97.9(86.6,105.5)	113.1(102.4,124.2)	< 0.0001
FPG, median (IQR), mmol.L	5.7(5.3,6.2)	5.2(4.9,5.4)	5.3(5.1,5.4)	5.8(5.7,6.1)	5.9(5.7,6.2)	7.1(6.3,7.8)	7.6(6.8,9.5)	< 0.0001
HbA1c, median (IQR),%	5.5(5.2,5.8)	5.3(5.0,5.5)	5.3(5.1,5.6)	5.4(5.2,5.7)	5.5(5.3,5.8)	6.6(6.2,6.8)	6.7(6.1,7.5)	< 0.0001
TC, median (IQR), mmol.L	4.7(4.1,5.5)	4.5(4.0,5.3)	4.9(4.3,5.5)	4.8(4.1,5.5)	5.0(4.3,5.7)	4.3(3.7,5.2)	4.4(3.8,5.0)	0.002
TG, median (IQR), mmol.L	1.0(0.7,1.5)	0.7(0.6,0.9)	1.1(0.8,1.6)	0.8(0.6,1.3)	1.2(0.8,1.6)	1.1(0.7,1.3)	1.5(1.1,2.1)	< 0.0001
HDL-C, median (IQR), mmol.L	1.3(1.1,1.6)	1.5(1.3,1.8)	1.3(1.1,1.6)	1.4(1.2,1.7)	1.2(1.1,1.5)	1.4(1.2,1.6)	1.1(1.0,1.4)	< 0.0001
LDL-C, median (IQR), mmol.L	2.8(2.3,3.4)	2.7(2.2,3.2)	2.9(2.3,3.5)	2.8(2.3,3.5)	3.0(2.5,3.6)	2.3(1.9,3.1)	2.4(1.9,3.1)	< 0.001
Creatinine, median (IQR), mg.dL	0.8(0.7,1.0)	0.8(0.7,0.9)	0.8(0.7,0.9)	0.9(0.8,1.0)	0.9(0.7,1.0)	0.9(0.7,1.1)	0.9(0.7,1.0)	< 0.0001
AST, median (IQR), U.L	19.0(16.0,23.0)	18.0(16.0,21.0)	19.0(15.0,23.0)	20.0(16.0,23.0)	20.0(17.0,24.0)	17.0(14.0,20.0)	19.0(15.0,25.0)	0.001
ALT, median (IQR), U.L	18.0(13.0,25.0)	14.0(11.0,19.0)	18.0(14.0,24.0)	17.0(13.0,23.0)	21.0(15.0,30.0)	14.0(12.0,17.0)	20.0(14.0,31.0)	< 0.0001
GGT, median (IQR), IU.L	20.0(14.0,29.0)	14.0(10.0,22.0)	20.0(15.0,30.0)	17.0(13.0,24.0)	23.0(16.0,33.0)	20.0(13.0,42.0)	24.0(17.0,39.0)	< 0.0001
Albumin, median (IQR), g.dL	4.1(3.9,4.3)	4.2(3.9,4.4)	4.0(3.8,4.2)	4.1(3.9,4.3)	4.1(3.9,4.2)	3.9(3.7,4.1)	4.0(3.8,4.2)	< 0.001
Total bilirubin, median (IQR), umol.L	6.8(5.1,10.3)	6.8(5.1,10.3)	6.8(5.1,10.3)	6.8(5.1,10.3)	6.8(5.1,10.3)	8.6(5.1,10.3)	8.6(5.1,10.3)	0.1
TyG, median (IQR)	8.5(8.0,8.9)	8.0(7.7,8.3)	8.4(8.0,8.9)	8.3(7.9,8.7)	8.6(8.2,9.0)	8.7(8.3,9.1)	9.1(8.8,9.6)	< 0.0001
TyG-BMI, median (IQR)	241.5(202.6,287.8)	192.8(168.5,223.6)	256.8(220.6,297.8)	214.8(192.7,251.6)	259.3(228.6,305.2)	237.7(203.3,264.7)	308.6(257.8,348.3)	< 0.0001
TyG-WC, median (IQR)	844.7(715.8,968.9)	686.2(603.8, 770.2)	870.0(780.5, 968.0)	758.4(684.4, 852.5)	892.8(814.8,1008.9)	856.5(749.0, 930.0)	1054.6(928.2,1143.7)	< 0.0001
TyG-WHtR, median (IQR)	5.0(4.3,5.8)	4.1(3.7,4.6)	5.2(4.7,5.8)	4.6(4.1,5.1)	5.3(4.7,6.0)	5.1(4.6,5.6)	6.2(5.5,6.9)	< 0.0001
TyG-WWI, median (IQR)	93.8(85.1,102.5)	82.9(77.4, 90.1)	94.9(87.7,100.5)	89.8(81.6, 95.5)	96.9(89.6,103.5)	97.4(93.5,104.9)	108.0(101.0,114.0)	< 0.0001
VAI, median (IQR)	1.2(0.7,2.2)	0.8(0.5,1.1)	1.4(0.8,2.5)	1.0(0.6,1.6)	1.4(0.9,2.4)	1.2(0.8,1.7)	2.4(1.6,3.4)	< 0.0001
HOMA-IR, median (IQR)	2.5(1.5,4.3)	1.2(0.9, 1.9)	2.4(1.3, 3.4)	2.2(1.6, 3.2)	3.1(2.0, 5.0)	2.9(2.0, 4.2)	6.0(3.7,10.4)	< 0.0001
METS-IR, median (IQR)	41.7(34.3,50.4)	32.6(28.6,39.1)	44.2(35.8,52.4)	36.9(32.3,42.7)	45.2(39.3,53.6)	40.3(34.5,43.8)	53.6(44.3,61.8)	< 0.0001
FSI, median (IQR)	−1.4(−2.5,−0.2)	−2.8(−3.5,−2.0)	−1.2(−2.0,−0.3)	−2.2(−2.9,−1.3)	−0.9(−1.8, 0.3)	−1.5(−2.4,−0.9)	0.2(−0.9, 1.1)	< 0.0001
FLI, median (IQR)	50.4(18.1,83.4)	13.0(4.9,31.9)	62.8(40.0,88.5)	26.1(12.1,53.6)	68.0(40.3,89.2)	41.3(18.3,65.7)	87.6(65.3,96.0)	< 0.0001
USFLI, median (IQR)	19.5(8.3,41.1)	5.4(3.1,10.9)	18.4(11.1,35.6)	12.7(7.6,23.8)	30.2(16.0,48.2)	20.2(11.3,38.7)	51.0(34.6,74.1)	< 0.0001
ZJU, median (IQR)	39.7(35.0,45.3)	33.5(31.2,37.2)	40.7(36.4,45.7)	36.5(33.9,40.4)	41.8(37.7,47.3)	38.1(36.0,43.8)	47.9(42.6,53.8)	< 0.0001
LAP, median (IQR)	38.1(19.7,68.3)	16.9(10.2, 30.8)	46.2(28.5, 74.9)	26.5(14.1, 43.9)	48.4(30.1, 77.2)	36.0(20.3, 51.6)	77.9(52.1,109.7)	< 0.0001
HSI, median (IQR)	37.7(32.7,43.7)	32.0(29.1,35.8)	39.0(34.4,45.2)	34.4(31.2,38.5)	40.2(35.5,45.8)	36.2(32.8,41.6)	44.0(39.1,50.2)	< 0.0001
NHHR, median (IQR)	2.5(1.8,3.3)	2.0(1.5,2.6)	2.7(1.9,3.5)	2.3(1.7,3.0)	2.9(2.1,3.8)	2.1(1.6,3.0)	2.8(2.1,3.6)	< 0.0001
NFS, median (IQR)	−1.5(−2.5,−0.4)	−2.4(−3.1,−1.7)	−1.9(−2.8,−0.9)	−2.0(−2.9,−1.0)	−1.3(−2.2,−0.3)	−0.1(−1.2, 0.9)	0.1(−0.6, 1.1)	< 0.0001
FIB-4, median (IQR)	0.9(0.6,1.3)	0.8(0.5,1.1)	0.8(0.5,1.2)	0.9(0.6,1.3)	1.0(0.7,1.3)	1.2(0.8,1.9)	1.1(0.9,1.5)	< 0.0001
BARD, median (IQR)	1.8(1.3,2.1)	1.4(1.2,1.8)	1.8(1.3,2.1)	1.4(1.1,1.9)	1.8(1.3,2.0)	2.3(2.0,2.9)	2.7(1.9,3.0)	< 0.0001
Obesity	1159(39.9)	98(15.2)	192(48.5)	88(19.6)	393(52.6)	37(29.1)	351(67.8)	< 0.001
Hypertension	1274(38.4)	127(15.8)	136(29.0)	159(28.2)	361(45.0)	80(68.5)	411(74.2)	< 0.001
CVD	334(9.5)	40(4.2)	35(9.1)	29(4.6)	71(8.7)	31(26.6)	128(21.8)	< 0.001
CKD	518(14.2)	57(7.8)	43(8.2)	59(9.8)	105(10.8)	52(41.4)	202(36.7)	< 0.001

MASLD, metabolic dysfunction-associated steatotic liver disease; T2DM, type 2 diabetes mellitus; AA: Associate of Arts; PIR: poverty income ratio; BMI: body mass index; WC: waist circumference; FPG: fasting plasma glucose; HbA1c: Glycated hemoglobin; TC: total cholesterol; TG: triglycerides; HDL-C: high-density lipoprotein cholesterol; LDL-C: low-density lipoprotein cholesterol; AST: aspartate aminotransferase; ALT: alanine aminotransferase; GGT: gamma-glutamyl transferase; TyG: triglyceride-glucose index; WHtR: waist-to-height ratio; WWI: weight-adjusted waist index; VAI: visceral adiposity index; HOMA-IR: homeostatic model assessment of insulin resistance; METS-IR: metabolic score for insulin resistance; FSI: Framingham steatosis index; FLI: fatty liver index; ZJU: Zhejiang University index; LAP: lipid accumulation product; HSI: hepatic steatosis index; NHHR: non-high-density lipoprotein cholesterol (HDL-C) to HDL-C ratio; NFS: nonalcoholic fatty liver disease fibrosis score; FIB-4: fibrosis-4 index; BARD: BMI-AST/ALT ratio and diabetes score; CVD: cardiovascular disease; CKD: chronic kidney disease

### Prevalence of MASLD and fibrosis by glycemic status

**Fig. 1:**
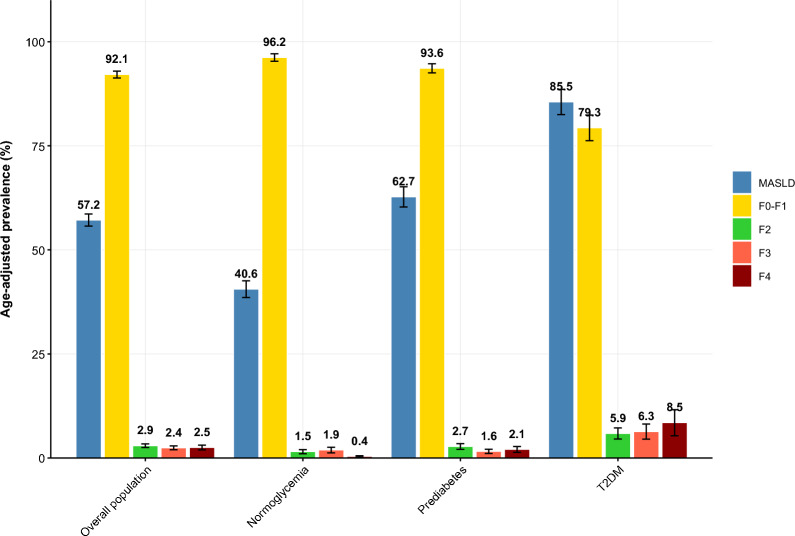
Age-adjusted prevalence of MASLD and liver fibrosis in normoglycemic, prediabetic and diabetic patients. MASLD, metabolic dysfunction-associated steatotic liver disease; T2DM, type 2 diabetes mellitus

The prevalence of MASLD (defined as hepatic steatosis with metabolic risk factors) and hepatic fibrosis among Americans aged ≥ 20 years were assessed using FibroScan data. In the overall population, the age-adjusted prevalence of MASLD was 57.2%, while the prevalence of significant fibrosis (≥ F2), advanced fibrosis (≥ F3), and cirrhosis (F4) was 7.9%, 4.9%, and 2.5%, respectively (Fig. 1). Stratified by glycemic status, the prevalence of MASLD exhibited a clear gradient, with the lowest rate observed in normoglycemic individuals (40.6%), followed by those with prediabetes (62.7%), and the highest in patients with T2DM (85.5%). Similarly, the burden of liver fibrosis increased with worsening glycemic control. In the normoglycemic group, the prevalence of fibrosis grades ≥ F2 and ≥ F3 was 3.8% and 2.3%, respectively. These data rose to 6.4% (≥ F2) and 3.6% (≥ F3) in prediabetes, and further increased to 20.7% (≥ F2) and 14.8% (≥ F3) in T2DM patients, highlighting the strong association between dysglycemia and progressive liver fibrosis. Notably, the prevalence of cirrhosis (F4) in T2DM patients was 8.5%, more than 20 times higher than that in nondiabetic patients (0.4%).

### Association of metabolic indices with MASLD and significant fibrosis across glycemic status

Table 2 shows the associations between 18 metabolic indices and MASLD/significant fibrosis in the overall population and across different glycemic states. In the overall population, multiple IR and lipid-related indices exhibited significant associations with MASLD. The TyG index and its derivatives (TyG-BMI, TyG-WC, TyG-WHtR, and TyG-WWI) consistently showed strong positive correlations, with TyG-WHtR presenting the highest OR (3.83, 95% CI 2.86–5.12). Similar trends were observed across glycemic subgroups. Other markers such as VAI, FSI, and METS-IR were also significantly associated with MASLD across all glycemic groups. In contrast, fibrosis scores (NFS, FIB-4, BARD) were less consistently associated with MASLD, often failing to reach statistical significance. Notably, T2DM individuals exhibited the strongest associations for most indices, such as FSI (OR: 4.08, 95% CI: 2.67–6.23) and TyG-WHtR (OR: 5.58, 95% CI: 3.08–10.13), underscoring their heightened metabolic risk.

**Table 2:** Association of 18 indices with MASLD and significant fibrosis across different glycemic states

Variables	Overall			Normoglycemia			Prediabetes			T2DM		
	OR (95% CI)	P-value (raw)	P-value (adjusted)	OR (95% CI)	P-value (raw)	P-value (adjusted)	OR (95% CI)	P-value (raw)	P-value (adjusted)	OR (95% CI)	P-value (raw)	P-value (adjusted)
MASLD												
TyG	3.08 (2.48, 3.81)	< 0.001	< 0.001	3.8 (2.28, 6.33)	< 0.001	0.004	2.17 (1.68, 2.8)	< 0.001	0.004	4.32 (2.11, 8.88)	0.003	0.043
TyG-BMI	1.02 (1.02, 1.03)	< 0.001	< 0.001	1.03 (1.02, 1.04)	< 0.001	< 0.001	1.02 (1.01, 1.03)	0.006	0.052	1.03 (1.02, 1.05)	< 0.001	0.004
TyG-WC	1.01 (1.01, 1.01)	< 0.001	< 0.001	1.01 (1.01, 1.01)	< 0.001	0.001	1.01 (1, 1.01)	0.001	0.011	1.01 (1.01, 1.01)	< 0.001	0.003
TyG-WHtR	3.83 (2.86, 5.12)	< 0.001	< 0.001	5.02 (3.1, 8.13)	< 0.001	< 0.001	2.59 (1.69, 3.97)	0.002	0.017	5.58 (3.08, 10.13)	< 0.001	0.006
TyG-WWI	1.08 (1.06, 1.1)	< 0.001	< 0.001	1.1 (1.07, 1.14)	< 0.001	0.002	1.06 (1.03, 1.08)	< 0.001	0.011	1.1 (1.06, 1.15)	0.001	0.027
VAI	1.82 (1.59, 2.08)	< 0.001	< 0.001	2.3 (1.66, 3.19)	< 0.001	0.003	1.43 (1.23, 1.66)	0.001	0.01	2.8 (1.66, 4.7)	0.004	0.068
HOMA-IR	1.31 (1.16, 1.49)	0.002	0.007	1.52 (1.17, 1.97)	0.011	0.04	1.22 (1.08, 1.38)	0.01	0.087	1.16 (0.99, 1.36)	0.102	0.458
METS-IR	1.13 (1.1, 1.16)	< 0.001	< 0.001	1.16 (1.11, 1.21)	< 0.001	< 0.001	1.09 (1.04, 1.14)	0.008	0.075	1.18 (1.12, 1.25)	< 0.001	0.005
FSI	2.52 (2.05, 3.1)	< 0.001	< 0.001	3.01 (2.23, 4.07)	< 0.001	< 0.001	1.92 (1.42, 2.58)	0.002	0.036	4.08 (2.67, 6.23)	< 0.001	0.002
FLI	1.05 (1.04, 1.05)	< 0.001	< 0.001	1.05 (1.04, 1.07)	< 0.001	< 0.001	1.04 (1.02, 1.05)	< 0.001	0.015	1.06 (1.04, 1.08)	< 0.001	0.008
USFLI	1.05 (1.04, 1.07)	< 0.001	0.002	1.06 (1.03, 1.1)	0.005	0.041	1.04 (1.02, 1.06)	0.002	0.04	1.05 (1.03, 1.08)	0.002	0.027
ZJU	1.22 (1.17, 1.27)	< 0.001	< 0.001	1.26 (1.17, 1.35)	< 0.001	0.001	1.15 (1.06, 1.25)	0.007	0.066	1.28 (1.18, 1.4)	< 0.001	0.005
LAP	1.03 (1.02, 1.04)	< 0.001	0.001	1.04 (1.02, 1.06)	0.001	0.007	1.02 (1.01, 1.03)	0.003	0.058	1.04 (1.02, 1.06)	0.002	0.036
HSI	1.17 (1.13, 1.21)	< 0.001	< 0.001	1.2 (1.13, 1.27)	< 0.001	0.002	1.11 (1.05, 1.18)	0.008	0.072	1.23 (1.11, 1.35)	0.003	0.05
NHHR	1.51 (1.3, 1.76)	< 0.001	0.002	1.59 (1.23, 2.05)	0.006	0.019	1.36 (1.13, 1.64)	0.009	0.084	1.93 (1.26, 2.97)	0.015	0.134
NFS	1.21 (1.03, 1.43)	0.048	0.108	1.1 (0.79, 1.52)	0.58	0.653	1.14 (0.88, 1.47)	0.352	0.656	1.1 (0.81, 1.51)	0.555	0.878
FIB-4	0.73 (0.57, 0.94)	0.036	0.072	0.9 (0.52, 1.54)	0.705	0.793	0.72 (0.49, 1.06)	0.134	0.408	0.71 (0.49, 1.03)	0.103	0.465
BARD	1.12 (0.85, 1.46)	0.451	0.695	0.99 (0.57, 1.72)	0.975	1.000	1.07 (0.66, 1.73)	0.801	0.876	0.73 (0.42, 1.26)	0.285	0.795
Significant fibrosis												
TyG	1.48 (1.07, 2.03)	0.041	0.147	0.53 (0.09, 3.13)	0.504	1.000	1.1 (0.63, 1.9)	0.749	0.963	1.35 (0.87, 2.08)	0.214	0.769
TyG-BMI	1.01 (1.01, 1.02)	< 0.001	0.001	1 (0.99, 1.02)	0.464	0.849	1.01 (1.01, 1.02)	0.008	0.072	1.01 (1.01, 1.02)	0.001	0.012
TyG-WC	1.01 (1, 1.01)	< 0.001	0.003	1 (1, 1.01)	0.289	0.804	1.01 (1, 1.01)	0.033	0.300	1.01 (1, 1.01)	0.001	0.013
TyG-WHtR	2.48 (1.77, 3.48)	< 0.001	0.005	1.8 (0.77, 4.21)	0.207	0.723	2.04 (1.16, 3.57)	0.035	0.311	2.57 (1.65, 4)	0.002	0.021
TyG-WWI	1.05 (1.02, 1.08)	0.006	0.027	1.04 (0.95, 1.14)	0.414	0.716	1.01 (0.98, 1.05)	0.507	0.774	1.05 (1.02, 1.09)	0.012	0.074
VAI	1.09 (1, 1.18)	0.08	0.232	0.91 (0.6, 1.37)	0.669	0.926	0.96 (0.77, 1.2)	0.722	0.929	1.08 (0.96, 1.2)	0.229	0.816
HOMA-IR	1.03 (1.02, 1.05)	0.006	0.028	1.02 (0.82, 1.26)	0.888	0.995	1.12 (1.02, 1.23)	0.041	0.183	1.02 (1.01, 1.04)	0.018	0.109
METS-IR	1.07 (1.04, 1.09)	< 0.001	0.003	1.02 (0.96, 1.08)	0.539	0.841	1.06 (1.02, 1.11)	0.017	0.151	1.06 (1.03, 1.09)	0.004	0.035
FSI	1.57 (1.33, 1.86)	< 0.001	0.005	1.23 (0.83, 1.8)	0.329	0.842	1.58 (1.14, 2.19)	0.022	0.200	1.51 (1.21, 1.88)	0.005	0.044
FLI	1.04 (1.02, 1.05)	0.003	0.019	1.02 (0.99, 1.06)	0.225	0.745	1.03 (1, 1.06)	0.092	0.552	1.06 (1.03, 1.09)	0.005	0.046
USFLI	1.04 (1.03, 1.04)	< 0.001	< 0.001	1.02 (1, 1.05)	0.12	0.432	1.03 (1.01, 1.06)	0.032	0.285	1.04 (1.02, 1.06)	0.003	0.027
ZJU	1.12 (1.08, 1.16)	< 0.001	0.001	1.05 (0.96, 1.14)	0.352	0.887	1.13 (1.04, 1.23)	0.015	0.131	1.1 (1.06, 1.15)	0.001	0.010
LAP	1.01 (1, 1.01)	0.004	0.018	1 (1, 1.01)	0.51	0.835	1 (1, 1.01)	0.277	0.554	1.01 (1, 1.01)	0.05	0.298
HSI	1.11 (1.07, 1.16)	< 0.001	0.003	1.04 (0.96, 1.13)	0.348	0.859	1.11 (1.03, 1.21)	0.03	0.266	1.11 (1.05, 1.18)	0.007	0.061
NHHR	0.94 (0.8, 1.11)	0.488	0.583	0.56 (0.26, 1.22)	0.181	0.542	0.91 (0.69, 1.2)	0.532	0.770	1.01 (0.78, 1.3)	0.958	1.000
NFS	1.78 (1.49, 2.11)	< 0.001	0.002	1.97 (1.36, 2.85)	0.006	0.053	1.59 (1.19, 2.13)	0.012	0.217	1.55 (1.15, 2.09)	0.018	0.105
FIB-4	2.15 (1.55, 3)	0.001	0.006	4.05 (1.76, 9.34)	0.009	0.057	1.98 (1.13, 3.5)	0.042	0.205	2.1 (1.42, 3.13)	0.005	0.030
BARD	1.77 (1.27, 2.46)	0.008	0.036	1.08 (0.57, 2.05)	0.81	0.980	0.76 (0.33, 1.79)	0.552	0.764	1.51 (0.95, 2.4)	0.115	0.519

Model was adjusted for age, sex, poverty income ratio, education level, smoking status, race, hypertension, and obesity. MASLD, metabolic dysfunction-associated steatotic liver disease; T2DM, type 2 diabetes mellitus; TyG: triglyceride-glucose index; BMI: body mass index; WC: waist circumference; WHtR: waist-to-height ratio; WWI: weight-adjusted waist index; VAI: visceral adiposity index; HOMA-IR: homeostatic model assessment of insulin resistance; METS-IR: metabolic score for insulin resistance; FSI: Framingham steatosis index; FLI: fatty liver index; ZJU: Zhejiang University index; LAP: lipid accumulation product; HSI: hepatic steatosis index; NHHR: non-high-density lipoprotein cholesterol (HDL-C) to HDL-C ratio; NFS: nonalcoholic fatty liver disease fibrosis score; FIB-4: fibrosis-4 index; BARD: BMI-aspartate aminotransferase/alanine aminotransferase ratio and diabetes score

For significant fibrosis, the predictive performance of metabolic indices exhibited substantial variation across glycemic states. In the overall population, TyG-related indices demonstrated consistent associations with fibrosis, particularly TyG-WHtR (OR: 2.48). Steatosis indices, including FSI (OR: 1.57), USFLI (OR: 1.04), and ZJU (OR: 1.12), also maintained significance, alongside traditional fibrosis markers NFS (OR: 1.78) and FIB-4 (OR: 2.15). However, these associations were largely attenuated or absent in individuals with normoglycemia and prediabetes, where no indices maintained statistical significance after correction. In stark contrast, T2DM patients exhibited robust and consistent associations across multiple index categories. TyG derivatives (TyG-BMI, TyG-WC, TyG-WHtR), insulin resistance markers (METS-IR), and steatosis indices (FSI, FLI, USFLI, ZJU) all demonstrated significant predictive value. Traditional fibrosis scores maintained utility in this subgroup, with FIB-4 showing particular promise (OR: 2.10). TyG and BARD were significantly associated with fibrosis in the overall population, but not within subgroups. Interestingly, VAI and NHHR showed no significant association with fibrosis in any subgroup, suggesting a limited role in fibrosis risk stratification.

### Diagnostic predictive value of 18 indices for MASLD/significant fibrosis across glycemic status

**Fig. 2:**
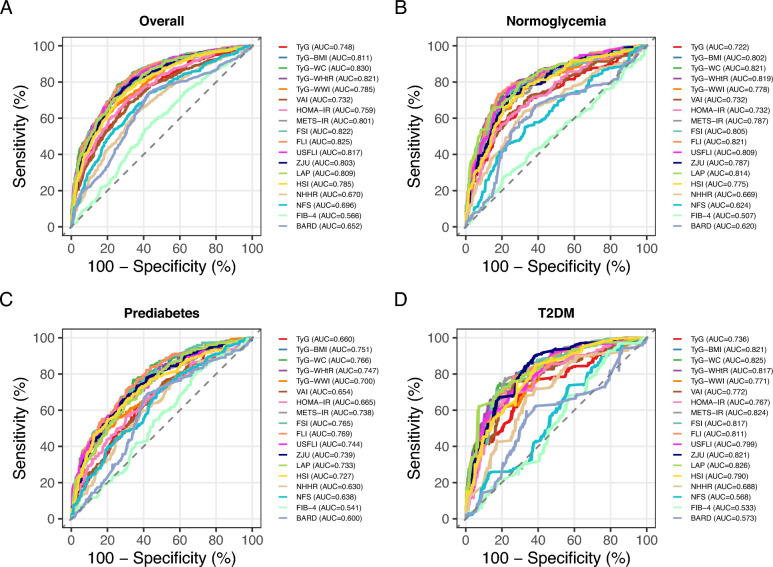
Receiver operating characteristic (ROC) curves and area under the ROC curve (AUC) values of 18 indexes for identifying MASLD across different glycemic states. (A) overall population; (B) normoglycemia; (C) prediabetes; and (D) type 2 diabetes mellitus (T2DM). MASLD, metabolic dysfunction–associated steatotic liver disease; TyG, triglyceride–glucose index; BMI, body mass index; WC, waist circumference; WHtR, waist-to-height ratio; WWI, weight-adjusted waist index; VAI, visceral adiposity index; HOMA-IR, homeostatic model assessment of insulin resistance; METS-IR, metabolic score for insulin resistance; FSI, Framingham steatosis index; FLI, fatty liver index; ZJU, Zhejiang University index; LAP, lipid accumulation product; HSI, hepatic steatosis index; NHHR, non–high-density lipoprotein cholesterol (HDL-C) to HDL-C ratio; NFS, nonalcoholic fatty liver disease fibrosis score; FIB-4, fibrosis-4 index; BARD, BMI–aspartate aminotransferase/alanine aminotransferase ratio and diabetes score.

The diagnostic performance of 18 metabolic indices for MASLD was assessed across different glycemic subgroups using ROC curve analysis (Fig. 2; Table S1). In the overall population, TyG-WC demonstrated the highest diagnostic accuracy for MASLD, with an AUC of 0.830, followed closely by FLI (AUC: 0.825), FSI (AUC: 0.822), and TyG-WHtR (AUC: 0.821). These indices exhibited balanced sensitivity (72.8–78.1%) and specificity (74.5–76.0%), with TyG-WC also showing the highest positive and negative predictive value. Among the glycemic subgroups, FLI and TyG-WC remained robust in normoglycemia (AUCs: 0.821), while LAP emerged as the top performer in T2DM (AUC: 0.826). Prediabetes showed slightly lower AUCs, with FLI (0.769) and TyG-WC (0.766) leading, although their sensitivity and specificity were less balanced compared to the other groups. Notably, traditional markers like HOMA-IR and TyG exhibited lower AUCs.

**Fig. 3:**
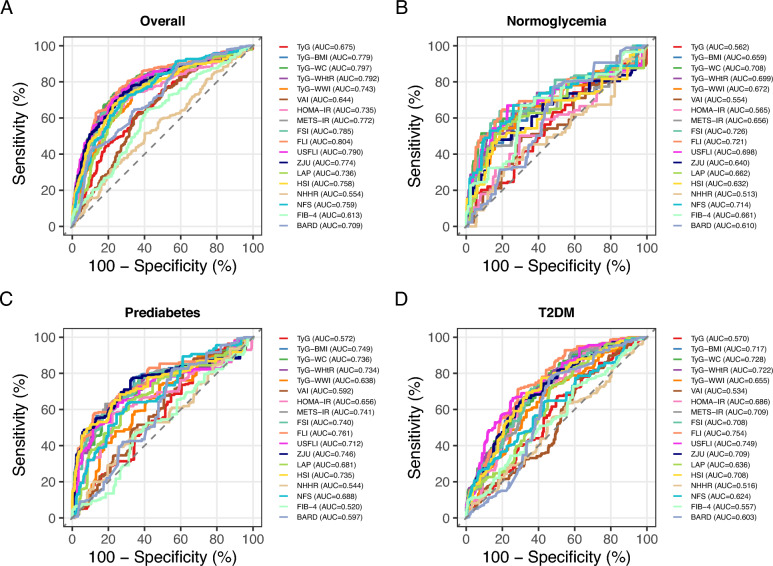
Receiver operating characteristic (ROC) curves and area under the ROC curve (AUC) values of 18 indexes for identifying significant fibrosis across different glycemic states. (A) overall population; (B) normoglycemia; (C) prediabetes; and (D) type 2 diabetes mellitus (T2DM). TyG, triglyceride–glucose index; BMI, body mass index; WC, waist circumference; WHtR, waist-to-height ratio; WWI, weight-adjusted waist index; VAI, visceral adiposity index; HOMA-IR, homeostatic model assessment of insulin resistance; METS-IR, metabolic score for insulin resistance; FSI, Framingham steatosis index; FLI, fatty liver index; ZJU, Zhejiang University index; LAP, lipid accumulation product; HSI, hepatic steatosis index; NHHR, non–high-density lipoprotein cholesterol (HDL-C) to HDL-C ratio; NFS, nonalcoholic fatty liver disease fibrosis score; FIB-4, fibrosis-4 index; BARD, BMI–aspartate aminotransferase/alanine aminotransferase ratio and diabetes score.

For significant fibrosis, FLI again led in the overall population (AUC: 0.804, 95% CI: 0.753–0.848), with TyG-WC (AUC: 0.797) and TyG-WHtR (AUC: 0.792) also performing well (Fig. 3; Table S2). In normoglycemia, FSI achieved the highest AUC (0.726), while FLI was optimal in prediabetes (AUC: 0.761) and T2DM (AUC: 0.754). Across all subgroups, commonly used indices such as FIB-4 and BARD for screening fibrosis performed poorly (AUCs: 0.520–0.661). These findings underscore TyG-WC and FLI as reliable indicators, with their diagnostic utility being notably influenced by glycemic status, highlighting the importance of glycemic-stratified approaches in assessing metabolic liver disease risk.

### Subgroup analysis of metabolic indicators for the predictive values of MASLD across different glycemic states and demographic characteristics

Subgroup analysis revealed significant variations in the diagnostic performance of 18 metabolic indices for MASLD across different glycemic states and demographic strata. TyG-WHtR and TyG-WC demonstrated the highest accuracy in females and males, respectively (Fig. S2; Table S3). Among normoglycemic individuals, TyG-WHtR and FLI were the most optimal in females, while TyG-WC and FLI prevailed in males (Fig. S3; Table S4). In prediabetes, USFLI and FLI performed best in females, whereas FSI and FLI excelled in males (Fig. S4; Table S5). For T2DM, FSI and METS-IR led in females, while TyG-WC and LAP showed superior performance in males (Fig. S5; Table S6). TyG-WC and USFLI exhibited dominance in Mexican Americans, with similar trends observed in non-Hispanic Whites and non-Hispanic Blacks. Age stratification revealed that TyG-WC was the top predictor in individuals aged 50 or younger, while FLI remained a robust predictor for those older than 50. Smoking status influenced performance, with TyG-WC and USFLI leading in never-smokers, whereas METS-IR and TyG-BMI excelled in current smokers with T2DM. Obesity further modulated diagnostic efficacy, with ZJU and LAP showing high accuracy in non-obese and obese T2DM subgroups, respectively. Hypertension status also impacted performance, with TyG-WC and FLI leading in non-hypertensive subjects, while USFLI and TyG-WC were optimal in hypertensive populations. These findings underscore the importance of tailored metabolic index selection based on glycemic and demographic profiles.

### Subgroup analysis of metabolic indicators for the predictive values of significant fibrosis across different glycemic states and demographic characteristics

The diagnostic performance of non-invasive indices for significant fibrosis varied considerably across different glycemic states and demographic subgroups. FLI consistently demonstrated robust accuracy in females (Fig. S6-S9 and Table S7-S10), particularly in those with prediabetes, while USFLI and TyG-WWI excelled in normoglycemic females. In males, FLI remained the strongest overall predictor, although sex-specific variations were noted, with NFS and FSI performing best in normoglycemic males. USFLI and FLI showed high diagnostic accuracy in Mexican Americans, whereas non-Hispanic Blacks exhibited lower overall performance. Age-stratified analyses identified FLI and FSI as leading indices in younger individuals (≤ 50 years), while FLI and TyG-WC dominated in older cohorts (> 50 years). Smoking status also influenced diagnostic efficacy, with BARD and NFS performing best in current smokers. Obesity status modulated the utility of certain indices, with TyG-WC and TyG-WHtR outperforming in obese individuals, while NFS and FIB-4 demonstrated moderate accuracy in non-obese subgroups. Among non-hypertensive individuals, USFLI exhibited the highest diagnostic accuracy, whereas FLI emerged as the top predictor in hypertensive populations. Notably, glycemic states introduced additional stratification—FLI consistently performed well in prediabetes and T2DM across most subgroups, while normoglycemic individuals often required distinct indices, such as NFS in older adults and Mexican Americans.

## Discussion

This comprehensive national study provides key insights into the complex relationship between dysglycemia and the burden of MASLD while rigorously evaluating the diagnostic performance of 18 metabolic indices across different glycemic states. The findings revealed that: (1) There is a significant glycemic gradient in MASLD prevalence and fibrosis risk, with a notably higher incidence of advanced liver disease in T2DM patients; (2) TyG-derived indicators, particularly TyG-WC and IR markers such as FLI, demonstrated high diagnostic accuracy, with optimal thresholds varying considerably based on glycemic status and demographic factors, highlighting the need for personalized clinical applications.

The redefinition of fatty liver disease from NAFLD to MASLD marks a significant shift in understanding its underlying pathophysiology, explicitly recognizing metabolic dysregulation as a core diagnostic criterion. Unlike NAFLD, which primarily focuses on hepatic steatosis in the absence of excessive alcohol consumption, MASLD incorporates metabolic dysregulation—particularly disturbances in glucose metabolism—as a central component of disease pathogenesis. Mechanistically, hyperglycemia and IR exacerbate hepatic lipid accumulation through enhanced de novo lipogenesis (DNL), impaired β-oxidation, and increased free fatty acid flux from adipose tissue [26]. Simultaneously, chronic hyperglycemia promotes hepatic inflammation and fibrogenesis via advanced glycation end-products and pro-inflammatory cytokines [27]. Our findings corroborate prior evidence that glycemic dysregulation is significantly associated with an increased MASLD risk, underscoring the metabolic heterogeneity inherent to this condition [28]. However, few studies have systematically evaluated whether glycemic stratification enhances the predictive utility of non-invasive indices—an important gap that this study aims to address.

Early detection of MASLD (defined as hepatic steatosis with metabolic risk factors) is crucial to prevent progression to steatohepatitis (MASH), fibrosis, and hepatocellular carcinoma. While liver biopsy remains the gold standard, its invasiveness limits widespread application, underscoring the need for reliable non-invasive alternatives. The TyG index and related markers have emerged as surrogates for insulin resistance (IR) and hepatic steatosis, yet their diagnostic performance varies considerably across metabolic subgroups. For example, TyG-BMI shows higher predictive accuracy for MASLD in males and older adults [29], while the HSI exhibits limited discriminative power in Korean T2DM patients—precisely the group at greatest risk for MASLD [30]. These discrepancies underscore the limitations of a uniform diagnostic approach and highlight the necessity for glycemic-stratified, context-specific tools that consider metabolic heterogeneity.

The TyG index, derived from fasting triglyceride and glucose levels, serves as a validated surrogate marker for IR. Its diagnostic accuracy is further enhanced when combined with anthropometric measures of obesity. Our research has found that TyG-WC consistently exhibited the highest diagnostic accuracy for MASLD across all glycemic states (AUC range: 0.766–0.830). This aligns with prior studies suggesting that visceral adiposity is a stronger determinant of hepatic steatosis than general obesity and that WC more directly reflects visceral fat mass and associated inflammatory cytokines. Notably, TyG-WC also maintained robust discriminative capacity in normoglycemic individuals (AUC: 0.821), highlighting its utility even in metabolically "silent" subpopulations. In contrast, traditional markers like HOMA-IR showed limited utility in prediabetes (AUC: 0.665), likely due to their reliance on static glucose-insulin measurements rather than dynamic lipid-glucose interactions.

MASH is the advanced period of MASLD, characterized by inflammatory responses and fibrosis. During this process, inflammation serves as a crucial bridge between fatty transformation and fibrosis. FLI, calculated using BMI, WC, triglycerides, and gamma-glutamyl transferase (GGT), is a clinically valuable, non-invasive biomarker that integrates parameters indicative of both hepatic steatosis and inflammation. Our study confirmed the superior performance of FLI in diagnosing both MASLD and significant fibrosis, reflecting its ability to detect early fibrogenic shifts driven by adipose inflammation and lipotoxicity. This is critical given that prediabetes represents a high-risk window in which fibrosis may advance more readily, as evidenced by our prevalence data (significant fibrosis: 6.4% in prediabetes vs. 3.8% in normoglycemia). Interestingly, while FLI outperformed other indices for fibrosis in patients with T2DM, its diagnostic accuracy was slightly lower compared to that in prediabetic patients, likely due to advanced disease stages where fibrosis progresses independently of steatosis. The dual capacity of FLI to capture steatosis and the stage of fibrosis underscores its potential value in long-term monitoring, particularly in high-risk groups such as those with prediabetes and metabolic syndrome.

The profound heterogeneity in the performance of metabolic indices across demographic and glycemic subgroups underscores the necessity of a nuanced, stratified approach to MASLD risk assessment. There are several common characteristics across different subgroups: firstly, TyG-WC and FLI consistently outperformed other indices across nearly all demographic and glycemic strata, solidifying their role as core tools for MASLD screening. Secondly, glycemic status was significantly associated with variations in index performance, with prediabetes representing an inflection point where IR markers (such as TyG-BMI) gained sensitivity, while fibrosis-related indices (such as FLI) reached peak accuracy. Thirdly, in the T2DM group, FSI, LAP, and METS-IR demonstrated high diagnostic accuracy, mirroring the escalating contributions of IR and lipotoxicity in advanced disease. However, demographic variables—such as age, sex, race, smoking status, obesity, hypertension, and glycemic status—further refined the utility of the indices. TyG-WC demonstrated superior accuracy in males and Mexican Americans, reflecting visceral adiposity-driven IR [31], whereas FLI outperformed in females and older individuals, likely due to sex-specific adipose distribution and cumulative hepatocyte injury [32]. These findings highlight that the efficacy of metabolic indices is context-dependent, emphasizing the need for glycemic- and demographic-tailored tool selection in clinical practice.

Our findings challenge the utility of uniform biomarker thresholds in MASLD screening, advocating for glycemic-stratified, phenotype-specific strategies. This study introduces a novel ‘glycemic stratification + individualized index matching’ model, proposing a two-tiered approach: TyG-WC and FLI for baseline assessment, supplemented by metabolic-specific indices in high-risk subgroups. This paradigm shift aligns with precision medicine, optimizing early detection through personalized risk prediction. This study is to our knowledge the first to assess the performance differences of non-invasive indices in predicting MASLD and fibrosis across varying glycemic states. The strengths include a large, nationally representative sample, FibroScan for objective steatosis and fibrosis assessment, and a comprehensive analysis of metabolic indices across subgroups. However, the cross-sectional design limits causal inference, and future longitudinal studies are needed to evaluate the predictive value of these indices for hard endpoints such as cirrhosis and cardiovascular events, especially in prediabetes. Additionally, some indices, such as ZJU and NHHR, were developed in specific populations and may require recalibration for broader use. While FibroScan is an ethical alternative to liver biopsy, it may lead to misclassification, particularly in early fibrosis stages. Furthermore, NHANES includes information on medication use, diet, and physical activity, but these variables have substantial missing data in the FibroScan subset. As a result, they were not included in the adjusted models to avoid loss of statistical power and potential selection bias. Future studies with more complete data are needed to better evaluate the role of these factors.

## Conclusions

Our study establishes the clinical relevance of glycemic-status–based dynamic index selection for MASLD risk assessment. The consistent performance of TyG-WC and FLI across diverse populations supports their role as cornerstones of non-invasive MASLD evaluation, while subgroup-adapted application of IR-specific indices can enhance diagnostic precision in high-risk groups. Although FSI demonstrated strong performance in males and in individuals with T2DM, TyG-WC and FLI were emphasized because they exhibited stable and superior diagnostic accuracy across glycemic and demographic subgroups, underscoring their suitability as universal screening tools. This stratified approach marks a crucial step toward metabolic phenotype–index matching, redefining MASLD management within the framework of precision medicine.

## Supplementary Information

Additional file 1.

Additional file 2.

## Data Availability

Raw data supporting the results of this study will be available upon reasonable request.

## Abbreviations

AA: Associate of Arts
ALT: Alanine aminotransferase
AST: Aspartate aminotransferase
AUC: Area under the curve
BARD: BMI-AST/ALT ratio and diabetes score
BMI: Body mass index
CAP: Controlled attenuation parameter
CI: Confidence interval
CKD: Chronic kidney disease
CVD: Cardiovascular disease
DNL: De novo lipogenesis
FBG: Fasting blood glucose
FIB-4: Fibrosis-4 index
FLI: Fatty liver index
FPG: Fasting plasma glucose
FSI: Framingham steatosis index
GGT: Gamma-glutamyl transferase
HbA1c: Glycated hemoglobin
HDL-C: High-density lipoprotein cholesterol
HOMA-IR: Homeostatic model assessment of insulin resistance
HSI: Hepatic steatosis index; IR: Insulin resistance
LAP: Lipid accumulation product
LDL-C: Low-density lipoprotein cholesterol
LSM: Liver stiffness measurement
MASLD: Metabolic dysfunction-associated steatotic liver disease
MASH: Metabolic dysfunction-associated steatohepatitis
METS-IR: Metabolic score for insulin resistance
NAFLD: Nonalcoholic fatty liver disease
NHANES: National Health and Nutrition Examination Survey
NHHR: Non-high-density lipoprotein cholesterol to HDL-C ratio
NFS: Nonalcoholic fatty liver disease fibrosis score
OR: Odds ratio
PIR: Poverty income ratio
ROC: Receiver operating characteristic
T2DM: Type 2 diabetes mellitus
TC: Total cholesterol
TG: Triglycerides
TyG: Triglyceride-glucose index
TyG-BMI: Triglyceride-glucose-body mass index
TyG-WC: Triglyceride-glucose-waist circumference
TyG-WHtR: Triglyceride-glucose-waist-to-height ratio
TyG-WWI: Triglyceride-glucose-weight-adjusted waist index
USFLI: US fatty liver index
VAI: Visceral adiposity index
VLDL: Very-low-density lipoprotein
WC: Waist circumference
WHtR: Waist-to-height ratio
WWI: Weight-adjusted waist index
ZJU: Zhejiang University index
