# Supplementary material for: Personalized MASLD and liver fibrosis risk assessment for adults: glycemic status determines optimal choice of non-invasive indices
Source: Diabetol Metab Syndr. 2025 Dec 22;17:461. doi: 10.1186/s13098-025-02069-w (PMC12751846; doi:10.1186/s13098-025-02069-w)
Supplement: Supplementary file 1 — Additional file 1. [file 13098_2025_2069_MOESM1_ESM.docx]

STROBE Statement—checklist of items that should be included in reports of observational studies

|  | **Item No.** | **Recommendation** | **Page** | **Relevant text from manuscript** |
| --- | --- | --- | --- | --- |
|  |  |  | **No.** |  |
| **Title and abstract** | 1 | (a) Indicate the study’s design with a commonly used term in the title or the abstract | 1 | “Cross-sectional analyses were performed…” |
|  |  | (b) Provide in the abstract an informative and balanced summary of what was done and what was found | 3 | “Background:…Methods:…Results:…Conclusions:…” |
| **Introduction** | | | |  |
| Background/rationale | 2 | Explain the scientific background and rationale for the investigation being reported | 5 | “MASLD…is inherently connected to systemic metabolic disturbances…” |
| Objectives | 3 | State specific objectives, including any prespecified hypotheses | 6 | “Our study aims to identify robust, non-invasive biomarkers tailored to distinct metabolic profiles…” |
| **Methods** | | | |  |
| Study design | 4 | Present key elements of study design early in the paper | 6 | “Cross-sectional analyses were performed on 2,794 individuals…” |
| Setting | 5 | Describe the setting, locations, and relevant dates, including periods of recruitment, exposure, follow-up, and data collection | 6 | “data from the 2017–2020 National Health and Nutrition Examination Survey (NHANES)…” |
| Participants | 6 | (a) Cohort study—Give the eligibility criteria, and the sources and methods of selection of participants. Describe methods of follow-up | 6 | “Participants aged ≥20 years with complete FibroScan and metabolic data were included…” |
|  |  | *Case-control study*—Give the eligibility criteria, and the sources and methods of case ascertainment and control selection. Give the rationale for the choice of cases and controls |  |  |
|  |  | *Cross-sectional study*—Give the eligibility criteria, and the sources and methods of selection of participants |  |  |
|  |  | (b) Cohort study—For matched studies, give matching criteria and number of exposed and unexposed |  |  |
|  |  | *Case-control study*—For matched studies, give matching criteria and the number of controls per case |  |  |
| Variables | 7 | Clearly define all outcomes, exposures, predictors, potential confounders, and effect modifiers. Give diagnostic criteria, if applicable | 6月7日 | “MASLD is defined as…; significant fibrosis…; hypertension…” |
| Data sources/ measurement | 8* | For each variable of interest, give sources of data and details of methods of assessment (measurement). Describe comparability of assessment methods if there is more than one group | *6* | *“Demographic information…obtained from the NHANES dataset…”* |
| Bias | 9 | Describe any efforts to address potential sources of bias | 7 | “Missing values were addressed using complete-case analysis…” |
| Study size | 10 | Explain how the study size was arrived at | 6 | “Finally, 2,794 participants were included…” |
| Quantitative variables | 11 | Explain how quantitative variables were handled in the analyses. If applicable, describe which groupings were chosen and why | 7 | “continuous variables were non-normally distributed…presented as median (IQR)” |
| Statistical methods | 12 | (a) Describe all statistical methods, including those used to control for confounding | 7 | “multivariable logistic regression…ROC curve analysis…Benjamini-Hochberg FDR…” |
|  |  | (b) Describe any methods used to examine subgroups and interactions | 7 | “Subgroup analysis revealed significant variations…” |
|  |  | (c) Explain how missing data were addressed | 7 | “Missing values were addressed using complete-case analysis” |
|  |  | (d) Cohort study—If applicable, explain how loss to follow-up was addressed | 7 | “Statistical analyses were performed according to NHANES guidelines using R…survey package” |
|  |  | *Case-control study*—If applicable, explain how matching of cases and controls was addressed |  |  |
|  |  | *Cross-sectional study*—If applicable, describe analytical methods taking account of sampling strategy |  |  |
|  |  | (e) Describe any sensitivity analyses |  |  |
| **Results** | | | | |
| Participants | 13* | (a) Report numbers of individuals at each stage of study—eg numbers potentially eligible, examined for eligibility, confirmed eligible, included in the study, completing follow-up, and analysed | 6 | “Initially, data from 15,560 participants…Finally, 2,794 participants were included” |
|  |  | (b) Give reasons for non-participation at each stage | 6 | “Exclusion criteria included: …missing FibroScan data…” “Exclusion criteria included: …missing FibroScan data…” |
|  |  | (c) Consider use of a flow diagram | 6 | “(Fig. S1)” |
| Descriptive data | 14* | (a) Give characteristics of study participants (eg demographic, clinical, social) and information on exposures and potential confounders | 7 | “Table 1…stratified by glycemic status and the presence of MASLD” |
|  |  | (b) Indicate number of participants with missing data for each variable of interest | 6 | “missing values for calculating the 18 metabolic indices” |
|  |  | (c) Cohort study—Summarise follow-up time (eg, average and total amount) |  |  |
| Outcome data | 15* | *Cohort study*—Report numbers of outcome events or summary measures over time |  |  |
|  |  | *Case-control study—*Report numbers in each exposure category, or summary measures of exposure |  |  |
|  |  | *Cross-sectional study—*Report numbers of outcome events or summary measures | *7* | *“The prevalence of MASLD…40.6% in normoglycemia…85.5% in T2DM”* |
| Main results | 16 | (a) Give unadjusted estimates and, if applicable, confounder-adjusted estimates and their precision (eg, 95% confidence interval). Make clear which confounders were adjusted for and why they were included | 7月8日 | “TyG-WHtR exhibited the strongest association with MASLD (OR: 3.83)…” |
|  |  | (b) Report category boundaries when continuous variables were categorized | 7 | “CAP ≥248 dB/m…LSM ≥8.2 kPa…” |
|  |  | (c) If relevant, consider translating estimates of relative risk into absolute risk for a meaningful time period |  |  |
| Other analyses | 17 | Report other analyses done—eg analyses of subgroups and interactions, and sensitivity analyses | 8–9 | “Subgroup analysis revealed significant variations…across glycemic states and demographic strata” |
| **Discussion** | | | | |
| Key results | 18 | Summarise key results with reference to study objectives | 13 | “The findings revealed that: (1)…(2)…” |
| Limitations | 19 | Discuss limitations of the study, taking into account sources of potential bias or imprecision. Discuss both direction and magnitude of any potential bias | 17 | “cross-sectional design limits causal inference…misclassification…missing data…” |
| Interpretation | 20 | Give a cautious overall interpretation of results considering objectives, limitations, multiplicity of analyses, results from similar studies, and other relevant evidence | 17 | “Our findings challenge the utility of uniform biomarker thresholds…” |
| Generalisability | 21 | Discuss the generalisability (external validity) of the study results | 17 | “nationally representative sample…future longitudinal studies are needed” |
| **Other information** | |  | | |
| Funding | 22 | Give the source of funding and the role of the funders for the present study and, if applicable, for the original study on which the present article is based | 17 | “This study was supported by National Natural Science Foundation of China…” |

*Give information separately for cases and controls in case-control studies and, if applicable, for exposed and unexposed groups in cohort and cross-sectional studies.

**Note:** An Explanation and Elaboration article discusses each checklist item and gives methodological background and published examples of transparent reporting. The STROBE checklist is best used in conjunction with this article (freely available on the Web sites of PLoS Medicine at http://www.plosmedicine.org/, Annals of Internal Medicine at http://www.annals.org/, and Epidemiology at http://www.epidem.com/). Information on the STROBE Initiative is available at www.strobe-statement.org.
