# Supplementary material for: Personalized MASLD and liver fibrosis risk assessment for adults: glycemic status determines optimal choice of non-invasive indices
Source: Diabetol Metab Syndr. 2025 Dec 22;17:461. doi: 10.1186/s13098-025-02069-w (PMC12751846; doi:10.1186/s13098-025-02069-w)
Supplement: Supplementary file 2 — Additional file 2. [file 13098_2025_2069_MOESM2_ESM.docx]

**SUPPLEMENTARY MATERIAL**

**Personalized MASLD Risk Assessment for Adults: Glycemic Status Determines Optimal Choice of Non-invasive Indices**

Yuan Zhao, Dongyu Hu, Jiacheng Cheng, Huili Cao, Xiaojuan Wang, Junhua He, Yikun Zhu, [Jin Li](https://www.tandfonline.com/author/Li,+Jin)

[SUPPLEMENTARY MATERIAL 1](#_Toc16221)

[Supplementary Methods 3](#_Toc29017)

[Fig. S1. Flowchart for selection of study population 5](#_Toc17088)

[Fig. S2. ROC curves and area under the curve (AUC) values of 18 indices for MASLD in patients across different demographic characteristics 6](#_Toc10856)

[Fig. S3. ROC curves and area under the curve (AUC) values of 18 indices for MASLD in patients with normoglycemia across different demographic characteristics 7](#_Toc27145)

[Fig. S4. ROC curves and area under the curve (AUC) values of 18 indices for MASLD in patients with prediabetes across different demographic characteristics 8](#_Toc26826)

[Fig. S5. ROC curves and area under the curve (AUC) values of 18 indices for MASLD in patients with T2DM across different demographic characteristics 9](#_Toc3918)

[Fig. S6. ROC curves and area under the curve (AUC) values of 18 indices for significant fibrosis in patients across different demographic characteristics 10](#_Toc24669)

[Fig. S7. ROC curves and area under the curve (AUC) values of 18 indices for significant fibrosis in patients with normoglycemia across different demographic characteristics 12](#_Toc22666)

[Fig. S8. ROC curves and area under the curve (AUC) values of 18 indices for significant fibrosis in patients with prediabetes across different demographic characteristics 13](#_Toc16995)

[Fig. S9. ROC curves and area under the curve (AUC) values of 18 indices for significant fibrosis in patients with T2DM across different demographic characteristics 14](#_Toc4198)

[Table. S1. Diagnostic efficacy of different indices for MASLD across different glycemic states 15](#_Toc15269)

[Table. S2. Diagnostic efficacy of different indices for significant fibrosis across different glycemic states 21](#_Toc4672)

[Table. S3. Diagnostic efficacy of 18 indices for MASLD in patients across different demographic characteristics 27](#_Toc31961)

[Table. S4. Diagnostic efficacy of 18 indices for MASLD in patients with normoglycemia across different demographic characteristics 48](#_Toc19327)

[Table. S5. Diagnostic efficacy of 18 indices for MASLD in patients with prediabetes across different demographic characteristics 69](#_Toc26412)

[Table. S6. Diagnostic efficacy of 18 indices for MASLD in patients with T2DM across different demographic characteristics 90](#_Toc9334)

[Table. S7. Diagnostic efficacy of 18 indices for significant fibrosis in patients across different demographic characteristics 111](#_Toc1561)

[Table. S8. Diagnostic efficacy of 18 indices for significant fibrosis in patients with normoglycemia across different demographic characteristics 132](#_Toc30046)

[Table. S9. Diagnostic efficacy of 18 indices for significant fibrosis in patients with prediabetes across different demographic characteristics 147](#_Toc2292)

[Table. S10. Diagnostic efficacy of 18 indices for significant fibrosis in patients with T2DM across different demographic characteristics 167](#_Toc4150)

[Supplementary References 187](#_Toc16849)

**Supplementary Methods**

**Laboratory measurement and clinical data**

The following variables were obtained from the original database: 1) Demographic variables: age, sex, race, education level, and family income poverty rate (PIR); 2) Examination variables: vibration-controlled transient elastography (VCTE) parameters (liver stiffness measurement (LSM) and controlled attenuation parameter (CAP)), height (cm), weight (kg), and waist circumference (WC), blood pressure (BP) and body mass index (BMI); 3) Laboratory variables: alanine aminotransferase (ALT), aspartate aminotransferase (AST), gamma-glutamyltransferase (GGT), albumin, fasting plasma glucose (FPG), insulin, total cholesterol (TC), triglycerides (TG), low-density lipoprotein cholesterol (LDL-C), high-density lipoprotein lipids (HDL-C), uric acid (UA), creatinine (CRE), estimated glomerular filtration rate (eGFR), high-sensitivity C-reactive protein (hs-CRP) (mg/L); 4) Questionnaires: smoking status, alcohol consumption, prescription drug use, history of hypertension or diabetes.

Formulas for calculating triglyceride-glucose (TyG)^1^, TyG-WC^2^, TyG-BMI^3^, TyG-waist-to-height (WHtR)^4^, TyG-weight-adjusted waist index (WWI)^5^, BMI-aspartate aminotransferase/alanine aminotransferase ratio and diabetes score (BARD)^6^, visceral adiposity index (VAI)^7^, homeostasis model assessment of insulin resistance (HOMA-IR)^8^, metabolic score for IR^9^, Framingham steatosis index (FSI)^10^, fatty liver index (FLI)^11^, USFLI^12^, Zhejiang University index (ZJU)^13^, lipid accumulation product (LAP)^14^, hepatic steatosis index (HSI)^15^, nonalcoholic fatty liver disease fibrosis score (NFS)^16^, fibrosis-4 index (FIB-4)^17^, and non-high-density lipoprotein cholesterol (HDL-C) to HDL-C ratio (NHHR)^18^ were as follows:

TyG = Ln [TG (mg/dL) × FPG (mg/dL) / 2];

TyG-WC = TyG index × WC (cm);

TyG-BMI = TyG index × BMI (kg/m2);

TyG-WtHR = TyG index × [WC (cm) / Height (cm)];

TyG-WWI = TyG index × WC/√weight.

BARD = BMI≥28 (1 point) +AST/ALT Ratio≥0.8 (2 points) +Diabetes (1 point)

VAI = [WC (cm) / (39.68 + 1.88 × BMI (kg/m^2^))] × [TG (mmol/L) / 1.03] × [1.31 / HDL (mmol/L)], for male; [WC (cm)/ (36.58 + 1.89 × BMI (kg/m^2^))] × (TG (mmol/L) / 0.81) × (1.52 / HDL (mmol/L)), for female;

HOMA-IR= Fasting Insulin (μU/mL) × Fasting Glucose (mmol/L) /22.5

METS-IR= Fasting Insulin (μU/mL) × Fasting Glucose (mmol/L) / (22.5 × BMI (kg/m2))

FSI = -7.981 + 0.011 × age (years) - 0.146 × Sex (female = 1, male = 0) + 0.173 × BMI (kg/m^2^) + 0.007 × TG (mg/dL) + 0.593 × Hypertension (yes = 1, no = 0) + 0.789 × Diabetes (yes = 1, no = 0) + 11 × [ALT (U/L) / AST (U/L) > 1.33 (yes = 1, no = 0)]

FLI = (e ^0.953^ × ln TG (mg/dL) + 0.139 × BMI (kg/m2) + 0.718 × ln GGT (U/L) + 0.053 × WC - 15.745)/ (1 +e ^0.953^ × ln TG (mg/dL) + 0.139 × BMI (kg/m2) + 0.718 × ln GGT (U/L) + 0.053 × WC - 15.745)) × 100;

USFLI= (e^−0.8073^×non-Hispanic black+0.3458×Mexican American+0.0093×age (years)+0.6151×ln (GGT)+0.0249×WC+1.1792×ln(insulin)+0.8242×ln(glucose)−14.7812)/ (1 +e−0.8073×non-Hispanic black+0.3458×Mexican American+0.0093×age (years)+0.6151×ln (GGT)+0.0249×WC+1.1792×ln(insulin)+0.8242×ln(glucose)−14.7812) ×100

ZJU = BMI (kg/m^2^) + FPG (mmol/L) + TG (mmol/L) + 3 × [ALT (U/L) / AST (U/L)] (+ 2, if female);

LAP = [WC (cm) - 65] × TG (mmol/L) for males, LAP = [WC (cm) - 58] × TG (mmol/L) for females;

HSI=8 × (AST/ ALT) + BMI+2×Diabetes+2×Sex; Diabetes is a binary variable (1 for presence of diabetes, 0 for absence), Sex is a binary variable (1 for female, 0 for male)

NFS = -1.675+0.037×age (years) + 0.094×BMI (kg/m^2^) +1.13×intermitent fasting glucose/diabetes (yes= 1, no= 0) +0.99×AST/ALT-0.013×platelet count (10^9^/L)-0.66×albumin (g/dL)

FIB-4= age (years)×AST (U/L) / platelet count (109/L) ×√ALT (U/L)

​NHHR = Non-HDL Cholesterol/HDL Cholesterol

**Fig. S1.** Flowchart for selection of study population

**Fig. S2**. ROC curves and area under the curve (AUC) values of 18 indices for MASLD in patients across different demographic characteristics

(A, B) Subgroup analysis based on sex. (C-G) Subgroup analysis based on race. (H, I) Subgroup analysis based on age. (J-L) Subgroup analyses based on smoking status. (M, N) Subgroup analysis based on obesity. (O, P) Subgroup analysis based on hypertension. MASLD, metabolic dysfunction-associated steatotic liver disease; T2DM, type 2 diabetes mellitus; TyG: triglyceride-glucose index; BMI: body mass index; WC: waist circumference; WHtR: waist-to-height ratio; WWI: weight-adjusted waist index; VAI: visceral adiposity index; HOMA-IR: homeostatic model assessment of insulin resistance; METS-IR: metabolic score for insulin resistance; FSI: Framingham steatosis index; FLI: fatty liver index; ZJU: Zhejiang University index; LAP: lipid accumulation product; HSI: hepatic steatosis index; NHHR: non-high-density lipoprotein cholesterol (HDL-C) to HDL-C ratio; NFS: nonalcoholic fatty liver disease fibrosis score; FIB-4: fibrosis-4 index; BARD: BMI-aspartate aminotransferase/alanine aminotransferase ratio and diabetes scoreroduct; HSI: hepatic steatosis index; NHHR: non-high-density lipoprotein cholesterol (HDL-C) to HDL-C ratio; NFS: nonalcoholic fatty liver disease fibrosis score; FIB-4: fibrosis-4 index; BARD: BMI-aspartate aminotransferase/alanine aminotransferase ratio and diabetes score

**Fig. S3**. ROC curves and area under the curve (AUC) values of 18 indices for MASLD in patients with normoglycemia across different demographic characteristics

(A, B) Subgroup analysis based on sex. (C-G) Subgroup analysis based on race. (H, I) Subgroup analysis based on age. (J-L) Subgroup analyses based on smoking status. (M, N) Subgroup analysis based on obesity. (O, P) Subgroup analysis based on hypertension. MASLD, metabolic dysfunction-associated steatotic liver disease; TyG: triglyceride-glucose index; BMI: body mass index; WC: waist circumference; WHtR: waist-to-height ratio; WWI: weight-adjusted waist index; VAI: visceral adiposity index; HOMA-IR: homeostatic model assessment of insulin resistance; METS-IR: metabolic score for insulin resistance; FSI: Framingham steatosis index; FLI: fatty liver index; ZJU: Zhejiang University index; LAP: lipid accumulation product; HSI: hepatic steatosis index; NHHR: non-high-density lipoprotein cholesterol (HDL-C) to HDL-C ratio; NFS: nonalcoholic fatty liver disease fibrosis score; FIB-4: fibrosis-4 index; BARD: BMI-aspartate aminotransferase/alanine aminotransferase ratio and diabetes score

**Fig. S4**. ROC curves and area under the curve (AUC) values of 18 indices for MASLD in patients with prediabetes across different demographic characteristics

(A, B) Subgroup analysis based on sex. (C-F) Subgroup analysis based on race. (G, H) Subgroup analysis based on age. (I-K) Subgroup analyses based on smoking status. (L, M) Subgroup analysis based on obesity. (N, O) Subgroup analysis based on hypertension. MASLD, metabolic dysfunction-associated steatotic liver disease; TyG: triglyceride-glucose index; BMI: body mass index; WC: waist circumference; WHtR: waist-to-height ratio; WWI: weight-adjusted waist index; VAI: visceral adiposity index; HOMA-IR: homeostatic model assessment of insulin resistance; METS-IR: metabolic score for insulin resistance; FSI: Framingham steatosis index; FLI: fatty liver index; ZJU: Zhejiang University index; LAP: lipid accumulation product; HSI: hepatic steatosis index; NHHR: non-high-density lipoprotein cholesterol (HDL-C) to HDL-C ratio; NFS: nonalcoholic fatty liver disease fibrosis score; FIB-4: fibrosis-4 index; BARD: BMI-aspartate aminotransferase/alanine aminotransferase ratio and diabetes score

**Fig. S5**. ROC curves and area under the curve (AUC) values of 18 indices for MASLD in patients with T2DM across different demographic characteristics

(A, B) Subgroup analysis based on sex. (C-G) Subgroup analysis based on race. (H, I) Subgroup analysis based on age. (J-L) Subgroup analyses based on smoking status. (M, N) Subgroup analysis based on obesity. (O, P) Subgroup analysis based on hypertension. MASLD, metabolic dysfunction-associated steatotic liver disease; T2DM, type 2 diabetes mellitus; TyG: triglyceride-glucose index; BMI: body mass index; WC: waist circumference; WHtR: waist-to-height ratio; WWI: weight-adjusted waist index; VAI: visceral adiposity index; HOMA-IR: homeostatic model assessment of insulin resistance; METS-IR: metabolic score for insulin resistance; FSI: Framingham steatosis index; FLI: fatty liver index; ZJU: Zhejiang University index; LAP: lipid accumulation product; HSI: hepatic steatosis index; NHHR: non-high-density lipoprotein cholesterol (HDL-C) to HDL-C ratio; NFS: nonalcoholic fatty liver disease fibrosis score; FIB-4: fibrosis-4 index; BARD: BMI-aspartate aminotransferase/alanine aminotransferase ratio and diabetes score

**Fig. S6**. ROC curves and area under the curve (AUC) values of 18 indices for significant fibrosis in patients across different demographic characteristics

(A, B) Subgroup analysis based on sex. (C-G) Subgroup analysis based on race. (H, I) Subgroup analysis based on age. (J-L) Subgroup analyses based on smoking status. (M, N) Subgroup analysis based on obesity. (O, P) Subgroup analysis based on hypertension. MASLD, metabolic dysfunction-associated steatotic liver disease; T2DM, type 2 diabetes mellitus; TyG: triglyceride-glucose index; BMI: body mass index; WC: waist circumference; WHtR: waist-to-height ratio; WWI: weight-adjusted waist index; VAI: visceral adiposity index; HOMA-IR: homeostatic model assessment of insulin resistance; METS-IR: metabolic score for insulin resistance; FSI: Framingham steatosis index; FLI: fatty liver index; ZJU: Zhejiang University index; LAP: lipid accumulation product; HSI: hepatic steatosis index; NHHR: non-high-density lipoprotein cholesterol (HDL-C) to HDL-C ratio; NFS: nonalcoholic fatty liver disease fibrosis score; FIB-4: fibrosis-4 index; BARD: BMI-aspartate aminotransferase/alanine aminotransferase ratio and diabetes scoreroduct; HSI: hepatic steatosis index; NHHR: non-high-density lipoprotein cholesterol (HDL-C) to HDL-C ratio; NFS: nonalcoholic fatty liver disease fibrosis score; FIB-4: fibrosis-4 index; BARD: BMI-aspartate aminotransferase/alanine aminotransferase ratio and diabetes score

**Fig. S7.** ROC curves and area under the curve (AUC) values of 18 indices for significant fibrosis in patients with normoglycemia across different demographic characteristics

(A, B) Subgroup analysis based on sex. (C-G) Subgroup analysis based on race. (H, I) Subgroup analysis based on age. (J-L) Subgroup analyses based on smoking status. (M, N) Subgroup analysis based on obesity. (O, P) Subgroup analysis based on hypertension. TyG: triglyceride-glucose index; BMI: body mass index; WC: waist circumference; WHtR: waist-to-height ratio; WWI: weight-adjusted waist index; VAI: visceral adiposity index; HOMA-IR: homeostatic model assessment of insulin resistance; METS-IR: metabolic score for insulin resistance; FSI: Framingham steatosis index; FLI: fatty liver index; ZJU: Zhejiang University index; LAP: lipid accumulation product; HSI: hepatic steatosis index; NHHR: non-high-density lipoprotein cholesterol (HDL-C) to HDL-C ratio; NFS: nonalcoholic fatty liver disease fibrosis score; FIB-4: fibrosis-4 index; BARD: BMI-aspartate aminotransferase/alanine aminotransferase ratio and diabetes score

Note: We were unable to complete the subgroup analyses due to the insufficient positive cases of Mexican American and other race subgroups.

**Fig. S8**. ROC curves and area under the curve (AUC) values of 18 indices for significant fibrosis in patients with prediabetes across different demographic characteristics

(A, B) Subgroup analysis based on sex. (C-F) Subgroup analysis based on race. (G, H) Subgroup analysis based on age. (I-K) Subgroup analyses based on smoking status. (L, M) Subgroup analysis based on obesity. (N, O) Subgroup analysis based on hypertension. TyG: triglyceride-glucose index; BMI: body mass index; WC: waist circumference; WHtR: waist-to-height ratio; WWI: weight-adjusted waist index; VAI: visceral adiposity index; HOMA-IR: homeostatic model assessment of insulin resistance; METS-IR: metabolic score for insulin resistance; FSI: Framingham steatosis index; FLI: fatty liver index; ZJU: Zhejiang University index; LAP: lipid accumulation product; HSI: hepatic steatosis index; NHHR: non-high-density lipoprotein cholesterol (HDL-C) to HDL-C ratio; NFS: nonalcoholic fatty liver disease fibrosis score; FIB-4: fibrosis-4 index; BARD: BMI-aspartate aminotransferase/alanine aminotransferase ratio and diabetes score

Note: We were unable to complete the subgroup analyses due to the insufficient positive cases of other Hispanic subgroup.

**Fig. S9.** ROC curves and area under the curve (AUC) values of 18 indices for significant fibrosis in patients with T2DM across different demographic characteristics

(A, B) Subgroup analysis based on sex. (C-G) Subgroup analysis based on race. (H, I) Subgroup analysis based on age. (J-L) Subgroup analyses based on smoking status. (M, N) Subgroup analysis based on obesity. (O, P) Subgroup analysis based on hypertension. T2DM, type 2 diabetes mellitus; TyG: triglyceride-glucose index; BMI: body mass index; WC: waist circumference; WHtR: waist-to-height ratio; WWI: weight-adjusted waist index; VAI: visceral adiposity index; HOMA-IR: homeostatic model assessment of insulin resistance; METS-IR: metabolic score for insulin resistance; FSI: Framingham steatosis index; FLI: fatty liver index; ZJU: Zhejiang University index; LAP: lipid accumulation product; HSI: hepatic steatosis index; NHHR: non-high-density lipoprotein cholesterol (HDL-C) to HDL-C ratio; NFS: nonalcoholic fatty liver disease fibrosis score; FIB-4: fibrosis-4 index; BARD: BMI-aspartate aminotransferase/alanine aminotransferase ratio and diabetes score

**Table. S1.** Diagnostic efficacy of different indices for MASLD across different glycemic states

| Group | Variables | AUC (95%CI) | Sensitivity (%) | Specificity (%) | PPV | NPV | Cut-off Values | p-Value |
| --- | --- | --- | --- | --- | --- | --- | --- | --- |
| Overall | TyG | 0.748 (0.718-0.775) | 71.0 | 68.1 | 76.1 | 62.1 | 8.369 | <0.001 |
|  | TyG-BMI | 0.811 (0.784-0.834) | 76.3 | 72.8 | 80.1 | 68.3 | 232.708 | <0.001 |
|  | TyG-WC | 0.830 (0.805-0.852) | 78.1 | 74.6 | 81.5 | 70.4 | 813.164 | <0.001 |
|  | TyG-WHtR | 0.821 (0.797-0.844) | 72.8 | 76.0 | 81.3 | 66.1 | 4.939 | <0.001 |
|  | TyG-WWI | 0.785 (0.759-0.810) | 67.1 | 75.7 | 79.8 | 61.7 | 94.029 | <0.001 |
|  | VAI | 0.732 (0.701-0.761) | 68.3 | 68.2 | 75.5 | 60.1 | 1.159 | <0.001 |
|  | HOMA-IR | 0.759 (0.727-0.787) | 71.7 | 67.8 | 76.1 | 62.6 | 2.217 | <0.001 |
|  | METS-IR | 0.801 (0.775-0.825) | 70.9 | 78.3 | 82.4 | 65.3 | 41.472 | <0.001 |
|  | FSI | 0.822 (0.798-0.845) | 74.8 | 74.8 | 80.9 | 67.4 | -1.610 | <0.001 |
|  | FLI | 0.825 (0.801-0.849) | 77.3 | 74.5 | 81.3 | 69.6 | 42.742 | <0.001 |
|  | USFLI | 0.817 (0.791-0.841) | 77.7 | 70.4 | 79.0 | 68.8 | 15.676 | <0.001 |
|  | ZJU | 0.803 (0.778-0.829) | 76.3 | 71.7 | 79.4 | 67.9 | 38.222 | <0.001 |
|  | LAP | 0.809 (0.783-0.833) | 70.7 | 76.5 | 81.2 | 64.6 | 36.937 | <0.001 |
|  | HSI | 0.785 (0.759-0.810) | 73.2 | 71.4 | 78.6 | 65.1 | 36.595 | <0.001 |
|  | NHHR | 0.670 (0.636-0.701) | 63.3 | 64.3 | 71.7 | 55.0 | 2.428 | <0.001 |
|  | NFS | 0.696 (0.665-0.724) | 66.8 | 64.1 | 72.7 | 57.4 | -1.708 | <0.001 |
|  | FIB-4 | 0.566 (0.531-0.598) | 57.5 | 55.8 | 65.1 | 47.9 | 0.885 | 0.017 |
|  | BARD | 0.652 (0.623-0.680) | 73.1 | 58.0 | 71.3 | 60.1 | 1.583 | <0.001 |
| Normoglycemia | TyG | 0.722 (0.670-0.767) | 66.5 | 70.8 | 59.8 | 76.4 | 8.221 | <0.001 |
|  | TyG-BMI | 0.802 (0.756-0.842) | 71.9 | 77.4 | 67.5 | 80.9 | 227.016 | <0.001 |
|  | TyG-WC | 0.821 (0.776-0.860) | 73.8 | 79.4 | 70.1 | 82.3 | 789.240 | <0.001 |
|  | TyG-WHtR | 0.819 (0.777-0.859) | 78.8 | 74.0 | 66.4 | 84.2 | 4.584 | <0.001 |
|  | TyG-WWI | 0.778 (0.734-0.818) | 73.6 | 71.6 | 62.9 | 80.6 | 88.627 | <0.001 |
|  | VAI | 0.732 (0.682-0.782) | 62.8 | 75.6 | 62.8 | 75.7 | 1.151 | <0.001 |
|  | HOMA-IR | 0.732 (0.682-0.781) | 66.4 | 69.3 | 58.6 | 76.0 | 1.699 | <0.001 |
|  | METS-IR | 0.787 (0.743-0.828) | 67.2 | 77.8 | 66.4 | 78.4 | 39.646 | <0.001 |
|  | FSI | 0.805 (0.758-0.847) | 78.7 | 72.5 | 65.2 | 83.9 | -2.145 | <0.001 |
|  | FLI | 0.821 (0.777-0.860) | 73.3 | 81.9 | 72.6 | 82.5 | 43.056 | <0.001 |
|  | USFLI | 0.809 (0.764-0.850) | 76.4 | 75.9 | 67.5 | 83.2 | 10.899 | <0.001 |
|  | ZJU | 0.787 (0.741-0.828) | 71.6 | 77.0 | 67.0 | 80.6 | 37.595 | <0.001 |
|  | LAP | 0.814 (0.774-0.853) | 79.5 | 70.4 | 63.7 | 84.0 | 26.781 | <0.001 |
|  | HSI | 0.775 (0.726-0.819) | 72.0 | 72.5 | 63.1 | 79.9 | 35.200 | <0.001 |
|  | NHHR | 0.669 (0.616-0.721) | 63.2 | 68.3 | 56.6 | 74.0 | 2.369 | <0.001 |
|  | NFS | 0.624 (0.571-0.676) | 55.2 | 67.3 | 52.5 | 69.7 | -2.009 | <0.001 |
|  | FIB-4 | 0.507 (0.454-0.561) | 42.7 | 62.7 | 42.8 | 62.6 | 0.888 | 0.349 |
|  | BARD | 0.620 (0.565-0.673) | 67.3 | 61.6 | 53.4 | 74.2 | 1.585 | <0.001 |
| Prediabetes | TyG | 0.660 (0.601-0.711) | 62.9 | 63.1 | 75.5 | 48.6 | 8.457 | <0.001 |
|  | TyG-BMI | 0.751 (0.700-0.796) | 72.2 | 66.2 | 79.4 | 56.9 | 233.047 | <0.001 |
|  | TyG-WC | 0.766 (0.718-0.807) | 76.8 | 65.7 | 80.2 | 61.1 | 810.545 | <0.001 |
|  | TyG-WHtR | 0.747 (0.703-0.790) | 63.4 | 74.8 | 81.9 | 53.1 | 5.045 | <0.001 |
|  | TyG-WWI | 0.700 (0.651-0.745) | 61.0 | 67.3 | 77.1 | 48.9 | 93.939 | <0.001 |
|  | VAI | 0.654 (0.597-0.708) | 68.3 | 56.1 | 73.7 | 49.6 | 1.047 | <0.001 |
|  | HOMA-IR | 0.665 (0.609-0.719) | 56.7 | 69.6 | 77.1 | 47.1 | 2.813 | <0.001 |
|  | METS-IR | 0.738 (0.691-0.784) | 67.4 | 72.3 | 81.4 | 55.2 | 41.472 | <0.001 |
|  | FSI | 0.765 (0.722-0.810) | 68.5 | 69.9 | 80.4 | 55.2 | -1.476 | <0.001 |
|  | FLI | 0.769 (0.722-0.811) | 71.2 | 69.6 | 80.9 | 57.3 | 44.788 | <0.001 |
|  | USFLI | 0.744 (0.696-0.785) | 63.0 | 73.6 | 81.1 | 52.4 | 21.696 | <0.001 |
|  | ZJU | 0.739 (0.691-0.781) | 65.8 | 71.4 | 80.6 | 53.7 | 39.372 | <0.001 |
|  | LAP | 0.733 (0.684-0.782) | 63.6 | 70.1 | 79.3 | 51.7 | 38.155 | <0.001 |
|  | HSI | 0.727 (0.681-0.771) | 66.6 | 68.6 | 79.3 | 53.3 | 37.276 | <0.001 |
|  | NHHR | 0.630 (0.575-0.680) | 58.9 | 62.0 | 73.7 | 45.6 | 2.621 | <0.001 |
|  | NFS | 0.638 (0.585-0.689) | 65.7 | 55.5 | 72.7 | 47.3 | -1.803 | <0.001 |
|  | FIB-4 | 0.541 (0.483-0.594) | 53.9 | 54.5 | 68.1 | 39.6 | 0.944 | 0.281 |
|  | BARD | 0.600 (0.545-0.649) | 66.8 | 59.8 | 75.0 | 50.0 | 1.560 | 0.007 |
| T2DM | TyG | 0.736 (0.667-0.794) | 73.7 | 67.8 | 92.9 | 31.1 | 8.800 | <0.001 |
|  | TyG-BMI | 0.821 (0.757-0.871) | 80.8 | 70.6 | 94.0 | 39.2 | 250.987 | <0.001 |
|  | TyG-WC | 0.825 (0.773-0.870) | 74.9 | 81.1 | 95.8 | 36.1 | 930.787 | <0.001 |
|  | TyG-WHtR | 0.817 (0.761-0.866) | 80.6 | 72.1 | 94.3 | 39.4 | 5.387 | <0.001 |
|  | TyG-WWI | 0.771 (0.708-0.823) | 60.8 | 83.8 | 95.6 | 27.2 | 105.807 | <0.001 |
|  | VAI | 0.772 (0.698-0.832) | 75.8 | 73.1 | 94.2 | 34.6 | 1.549 | <0.001 |
|  | HOMA-IR | 0.767 (0.703-0.825) | 69.8 | 77.9 | 94.8 | 31.1 | 4.255 | 0.009 |
|  | METS-IR | 0.824 (0.768-0.873) | 77.7 | 78.3 | 95.3 | 38.0 | 43.987 | <0.001 |
|  | FSI | 0.817 (0.755-0.870) | 70.9 | 82.6 | 95.9 | 33.2 | -0.697 | <0.001 |
|  | FLI | 0.811 (0.752-0.866) | 77.9 | 74.0 | 94.5 | 36.9 | 62.152 | <0.001 |
|  | USFLI | 0.799 (0.730-0.859) | 70.7 | 74.0 | 94.0 | 30.7 | 38.053 | <0.001 |
|  | ZJU | 0.821 (0.752-0.877) | 77.6 | 71.7 | 94.0 | 35.9 | 41.995 | <0.001 |
|  | LAP | 0.826 (0.771-0.875) | 72.7 | 78.7 | 95.1 | 33.5 | 55.884 | <0.001 |
|  | HSI | 0.790 (0.722-0.851) | 75.7 | 71.6 | 93.8 | 34.0 | 39.033 | <0.001 |
|  | NHHR | 0.688 (0.601-0.772) | 68.0 | 66.6 | 92.1 | 26.7 | 2.364 | 0.010 |
|  | NFS | 0.568 (0.474-0.663) | 72.4 | 44.1 | 88.1 | 21.8 | -0.399 | 0.138 |
|  | FIB-4 | 0.533 (0.432-0.639) | 68.1 | 43.1 | 87.3 | 19.2 | 1.357 | 0.024 |
|  | BARD | 0.573 (0.477-0.659) | 59.5 | 66.5 | 91.0 | 22.3 | 2.509 | 0.068 |

MASLD, metabolic dysfunction-associated steatotic liver disease; T2DM, type 2 diabetes mellitus; PIR: Poverty income ratio; TyG: triglyceride-glucose index; BMI: body mass index; WC: waist circumference; WHtR: waist-to-height ratio; WWI: weight-adjusted waist index; VAI: visceral adiposity index; HOMA-IR: homeostatic model assessment of insulin resistance; METS-IR: metabolic score for insulin resistance; FSI: Framingham steatosis index; FLI: fatty liver index; ZJU: Zhejiang University index; LAP: lipid accumulation product; HSI: hepatic steatosis index; NHHR: non-high-density lipoprotein cholesterol (HDL-C) to HDL-C ratio; NFS: nonalcoholic fatty liver disease fibrosis score; FIB-4: fibrosis-4 index; BARD: BMI-aspartate aminotransferase/alanine aminotransferase ratio and diabetes scoreroduct; HSI: hepatic steatosis index; NHHR: non-high-density lipoprotein cholesterol (HDL-C) to HDL-C ratio; NFS: nonalcoholic fatty liver disease fibrosis score; FIB-4: fibrosis-4 index; BARD: BMI-aspartate aminotransferase/alanine aminotransferase ratio and diabetes score

**Table. S2**. Diagnostic efficacy of different indices for significant fibrosis across different glycemic states

| Group | Variables | AUC (95%CI) | Sensitivity (%) | Specificity (%) | PPV | NPV | Cut-off Values | p-Value |
| --- | --- | --- | --- | --- | --- | --- | --- | --- |
| Overall | TyG | 0.675 (0.630-0.719) | 63.2 | 63.5 | 13.6 | 95.0 | 8.652 | <0.001 |
|  | TyG-BMI | 0.779 (0.723-0.824) | 72.7 | 72.8 | 19.6 | 96.7 | 272.953 | <0.001 |
|  | TyG-WC | 0.797 (0.748-0.845) | 73.6 | 76.7 | 22.4 | 97.0 | 949.669 | <0.001 |
|  | TyG-WHtR | 0.792 (0.740-0.838) | 74.0 | 75.6 | 21.7 | 97.0 | 5.636 | <0.001 |
|  | TyG-WWI | 0.743 (0.692-0.789) | 74.0 | 66.3 | 16.7 | 96.5 | 97.823 | <0.001 |
|  | VAI | 0.644 (0.601-0.693) | 61.2 | 64.9 | 13.7 | 94.8 | 1.614 | <0.001 |
|  | HOMA-IR | 0.735 (0.679-0.786) | 68.9 | 71.5 | 18.1 | 96.2 | 3.685 | <0.001 |
|  | METS-IR | 0.772 (0.719-0.818) | 70.9 | 72.4 | 19.0 | 96.5 | 47.359 | <0.001 |
|  | FSI | 0.785 (0.736-0.827) | 73.6 | 73.5 | 20.2 | 96.8 | -0.555 | <0.001 |
|  | FLI | 0.804 (0.753-0.848) | 76.3 | 73.8 | 21.0 | 97.2 | 76.647 | <0.001 |
|  | USFLI | 0.790 (0.740-0.841) | 75.7 | 72.0 | 19.8 | 97.0 | 34.477 | <0.001 |
|  | ZJU | 0.774 (0.717-0.822) | 72.4 | 73.3 | 19.8 | 96.7 | 43.475 | <0.001 |
|  | LAP | 0.736 (0.689-0.779) | 71.7 | 70.2 | 18.0 | 96.4 | 54.573 | <0.001 |
|  | HSI | 0.758 (0.700-0.809) | 70.7 | 73.8 | 19.7 | 96.5 | 42.114 | <0.001 |
|  | NHHR | 0.554 (0.501-0.600) | 51.5 | 59.9 | 10.5 | 93.1 | 2.766 | 0.051 |
|  | NFS | 0.759 (0.714-0.803) | 68.7 | 73.8 | 19.3 | 96.3 | -0.634 | <0.001 |
|  | FIB-4 | 0.613 (0.566-0.661) | 63.4 | 59.6 | 12.5 | 94.7 | 1.015 | <0.001 |
|  | BARD | 0.709 (0.659-0.757) | 64.7 | 65.7 | 14.7 | 95.3 | 1.926 | <0.001 |
| Normoglycemia | TyG | 0.562 (0.421-0.707) | 63.2 | 52.0 | 4.7 | 97.4 | 8.158 | 0.671 |
|  | TyG-BMI | 0.659 (0.498-0.804) | 67.6 | 62.6 | 6.4 | 98.1 | 232.727 | 0.004 |
|  | TyG-WC | 0.708 (0.552-0.852) | 58.1 | 84.2 | 12.2 | 98.1 | 923.368 | 0.003 |
|  | TyG-WHtR | 0.699 (0.536-0.832) | 69.4 | 67.6 | 7.5 | 98.3 | 4.955 | 0.015 |
|  | TyG-WWI | 0.672 (0.499-0.805) | 66.2 | 70.6 | 7.9 | 98.2 | 93.166 | 0.104 |
|  | VAI | 0.554 (0.410-0.695) | 49.7 | 69.6 | 5.8 | 97.3 | 1.368 | 0.479 |
|  | HOMA-IR | 0.565 (0.403-0.706) | 50.2 | 70.5 | 6.1 | 97.4 | 2.377 | 0.044 |
|  | METS-IR | 0.656 (0.514-0.799) | 62.5 | 70.6 | 7.5 | 98.0 | 42.267 | 0.002 |
|  | FSI | 0.726 (0.572-0.860) | 63.8 | 81.4 | 11.5 | 98.3 | -0.889 | <0.001 |
|  | FLI | 0.721 (0.546-0.861) | 66.8 | 76.9 | 9.9 | 98.4 | 64.638 | 0.019 |
|  | USFLI | 0.698 (0.519-0.837) | 66.9 | 77.2 | 10.0 | 98.4 | 20.389 | <0.001 |
|  | ZJU | 0.640 (0.489-0.784) | 60.9 | 63.8 | 6.0 | 97.7 | 38.280 | 0.013 |
|  | LAP | 0.662 (0.503-0.800) | 60.4 | 71.8 | 7.5 | 98.0 | 41.976 | 0.003 |
|  | HSI | 0.632 (0.483-0.781) | 51.8 | 77.5 | 8.0 | 97.7 | 40.600 | 0.014 |
|  | NHHR | 0.513 (0.385-0.654) | 43.6 | 67.9 | 4.9 | 97.0 | 2.736 | 0.940 |
|  | NFS | 0.714 (0.541-0.851) | 67.1 | 73.9 | 8.9 | 98.3 | -1.503 | 0.004 |
|  | FIB-4 | 0.661 (0.522-0.784) | 66.9 | 69.7 | 7.7 | 98.2 | 1.023 | <0.001 |
|  | BARD | 0.610 (0.502-0.717) | 76.6 | 52.0 | 5.7 | 98.3 | 1.600 | 0.059 |
| Prediabetes | TyG | 0.572 (0.488-0.645) | 68.1 | 45.4 | 7.9 | 95.4 | 8.434 | 0.102 |
|  | TyG-BMI | 0.749 (0.627-0.843) | 77.6 | 65.9 | 13.5 | 97.7 | 261.901 | <0.001 |
|  | TyG-WC | 0.736 (0.622-0.829) | 65.0 | 77.0 | 16.2 | 97.0 | 955.102 | <0.001 |
|  | TyG-WHtR | 0.734 (0.624-0.834) | 70.1 | 71.2 | 14.3 | 97.2 | 5.492 | <0.001 |
|  | TyG-WWI | 0.638 (0.552-0.728) | 59.0 | 65.5 | 10.5 | 95.9 | 97.823 | 0.005 |
|  | VAI | 0.592 (0.516-0.666) | 67.7 | 49.2 | 8.4 | 95.7 | 1.202 | 0.260 |
|  | HOMA-IR | 0.656 (0.536-0.763) | 57.7 | 77.9 | 15.2 | 96.4 | 4.306 | <0.001 |
|  | METS-IR | 0.741 (0.622-0.842) | 63.1 | 81.6 | 19.1 | 97.0 | 51.538 | <0.001 |
|  | FSI | 0.740 (0.627-0.839) | 74.4 | 65.4 | 12.9 | 97.4 | -0.844 | <0.001 |
|  | FLI | 0.761 (0.640-0.858) | 69.8 | 72.7 | 14.9 | 97.2 | 76.647 | 0.004 |
|  | USFLI | 0.712 (0.601-0.818) | 63.8 | 74.7 | 14.8 | 96.8 | 38.056 | <0.001 |
|  | ZJU | 0.746 (0.620-0.847) | 77.1 | 67.2 | 13.9 | 97.7 | 42.200 | <0.001 |
|  | LAP | 0.681 (0.580-0.775) | 64.9 | 68.8 | 12.5 | 96.6 | 54.573 | 0.001 |
|  | HSI | 0.735 (0.611-0.835) | 68.4 | 73.5 | 15.0 | 97.1 | 42.500 | <0.001 |
|  | NHHR | 0.544 (0.441-0.634) | 48.8 | 64.3 | 8.6 | 94.8 | 3.054 | 0.350 |
|  | NFS | 0.688 (0.598-0.771) | 63.6 | 69.3 | 12.4 | 96.5 | -0.854 | 0.002 |
|  | FIB-4 | 0.520 (0.432-0.600) | 52.3 | 59.0 | 8.0 | 94.7 | 1.030 | 0.116 |
|  | BARD | 0.597 (0.514-0.681) | 76.5 | 47.5 | 9.1 | 96.7 | 1.610 | 0.048 |
| T2DM | TyG | 0.570 (0.502-0.629) | 67.3 | 49.6 | 28.9 | 83.2 | 9.019 | 0.088 |
|  | TyG-BMI | 0.717 (0.656-0.772) | 65.1 | 69.9 | 39.7 | 86.8 | 317.755 | <0.001 |
|  | TyG-WC | 0.728 (0.672-0.781) | 62.1 | 70.8 | 39.4 | 86.0 | 1087.053 | <0.001 |
|  | TyG-WHtR | 0.722 (0.653-0.784) | 65.9 | 68.8 | 39.2 | 86.9 | 6.322 | <0.001 |
|  | TyG-WWI | 0.655 (0.575-0.720) | 59.5 | 66.5 | 35.2 | 84.3 | 109.071 | <0.001 |
|  | VAI | 0.534 (0.472-0.607) | 76.7 | 39.6 | 27.9 | 84.8 | 1.675 | 0.118 |
|  | HOMA-IR | 0.686 (0.618-0.756) | 63.6 | 70.8 | 39.9 | 86.4 | 7.143 | 0.001 |
|  | METS-IR | 0.709 (0.646-0.765) | 66.8 | 68.2 | 39.1 | 87.1 | 55.753 | <0.001 |
|  | FSI | 0.708 (0.645-0.770) | 71.5 | 64.1 | 37.8 | 88.0 | 0.223 | <0.001 |
|  | FLI | 0.754 (0.692-0.807) | 71.4 | 71.2 | 43.1 | 89.1 | 90.429 | <0.001 |
|  | USFLI | 0.749 (0.682-0.814) | 66.3 | 74.2 | 44.0 | 87.8 | 61.911 | <0.001 |
|  | ZJU | 0.709 (0.646-0.770) | 65.9 | 68.2 | 38.7 | 86.8 | 49.031 | <0.001 |
|  | LAP | 0.636 (0.570-0.695) | 71.5 | 53.8 | 32.1 | 86.1 | 69.265 | 0.008 |
|  | HSI | 0.708 (0.640-0.763) | 69.3 | 66.6 | 38.7 | 87.7 | 44.933 | <0.001 |
|  | NHHR | 0.516 (0.445-0.591) | 53.4 | 55.1 | 26.6 | 79.5 | 2.766 | 0.629 |
|  | NFS | 0.624 (0.558-0.701) | 64.1 | 56.9 | 31.2 | 83.8 | 0.198 | 0.001 |
|  | FIB-4 | 0.557 (0.486-0.631) | 51.0 | 59.2 | 27.6 | 79.8 | 1.241 | 0.003 |
|  | BARD | 0.603 (0.540-0.667) | 77.1 | 54.1 | 33.9 | 88.5 | 2.576 | 0.004 |

T2DM, type 2 diabetes mellitus; TyG: triglyceride-glucose index; BMI: body mass index; WC: waist circumference; WHtR: waist-to-height ratio; WWI: weight-adjusted waist index; VAI: visceral adiposity index; HOMA-IR: homeostatic model assessment of insulin resistance; METS-IR: metabolic score for insulin resistance; FSI: Framingham steatosis index; FLI: fatty liver index; ZJU: Zhejiang University index; LAP: lipid accumulation product; HSI: hepatic steatosis index; NHHR: non-high-density lipoprotein cholesterol (HDL-C) to HDL-C ratio; NFS: nonalcoholic fatty liver disease fibrosis score; FIB-4: fibrosis-4 index; BARD: BMI-aspartate aminotransferase/alanine aminotransferase ratio and diabetes scoreroduct; HSI: hepatic steatosis index; NHHR: non-high-density lipoprotein cholesterol (HDL-C) to HDL-C ratio; NFS: nonalcoholic fatty liver disease fibrosis score; FIB-4: fibrosis-4 index; BARD: BMI-aspartate aminotransferase/alanine aminotransferase ratio and diabetes score

**Table. S3**. Diagnostic efficacy of 18 indices for MASLD in patients across different demographic characteristics

| Group | Variables | AUC (95%CI) | Sensitivity (%) | Specificity (%) | PPV | NPV | Cut-off Values | p-Value |
| --- | --- | --- | --- | --- | --- | --- | --- | --- |
| Female | TyG | 0.763 (0.728-0.795) | 68.9 | 72.7 | 75.4 | 65.8 | 8.391 | <0.001 |
|  | TyG-BMI | 0.820 (0.789-0.849) | 77.8 | 72.3 | 77.3 | 72.9 | 232.467 | <0.001 |
|  | TyG-WC | 0.836 (0.807-0.864) | 75.3 | 78.8 | 81.2 | 72.5 | 813.164 | <0.001 |
|  | TyG-WHtR | 0.837 (0.805-0.865) | 77.7 | 76.2 | 79.8 | 73.8 | 4.977 | <0.001 |
|  | TyG-WWI | 0.802 (0.770-0.835) | 73.7 | 75.7 | 78.6 | 70.3 | 94.157 | <0.001 |
|  | VAI | 0.751 (0.712-0.787) | 66.7 | 72.5 | 74.7 | 64.2 | 1.332 | <0.001 |
|  | HOMA-IR | 0.775 (0.740-0.808) | 72.0 | 69.1 | 73.9 | 67.0 | 2.221 | <0.001 |
|  | METS-IR | 0.806 (0.771-0.837) | 70.6 | 75.8 | 77.9 | 68.0 | 40.834 | <0.001 |
|  | FSI | 0.830 (0.800-0.859) | 73.1 | 76.8 | 79.3 | 70.2 | -1.613 | <0.001 |
|  | FLI | 0.835 (0.802-0.863) | 81.3 | 73.1 | 78.6 | 76.3 | 35.142 | <0.001 |
|  | USFLI | 0.836 (0.808-0.865) | 75.3 | 77.0 | 79.9 | 72.0 | 16.962 | <0.001 |
|  | ZJU | 0.815 (0.782-0.844) | 76.3 | 72.0 | 76.8 | 71.5 | 39.572 | <0.001 |
|  | LAP | 0.825 (0.791-0.854) | 73.5 | 77.6 | 79.9 | 70.7 | 37.023 | <0.001 |
|  | HSI | 0.802 (0.770-0.834) | 74.4 | 71.5 | 76.0 | 69.7 | 37.435 | <0.001 |
|  | NHHR | 0.680 (0.635-0.719) | 61.9 | 65.6 | 68.6 | 58.7 | 2.275 | <0.001 |
|  | NFS | 0.704 (0.667-0.743) | 69.7 | 62.2 | 69.1 | 62.9 | -1.728 | <0.001 |
|  | FIB-4 | 0.548 (0.507-0.591) | 57.7 | 54.3 | 60.5 | 51.4 | 0.885 | 0.083 |
|  | BARD | 0.662 (0.621-0.702) | 69.2 | 62.2 | 69.0 | 62.5 | 1.750 | <0.001 |
| Male | TyG | 0.727 (0.685-0.769) | 66.9 | 68.7 | 78.5 | 54.8 | 8.457 | <0.001 |
|  | TyG-BMI | 0.806 (0.767-0.843) | 76.3 | 72.9 | 82.8 | 64.2 | 231.444 | <0.001 |
|  | TyG-WC | 0.820 (0.781-0.857) | 81.6 | 69.1 | 81.9 | 68.7 | 811.731 | <0.001 |
|  | TyG-WHtR | 0.815 (0.774-0.852) | 68.6 | 78.3 | 84.4 | 59.3 | 4.883 | <0.001 |
|  | TyG-WWI | 0.779 (0.735-0.821) | 78.2 | 62.5 | 78.1 | 62.7 | 88.684 | <0.001 |
|  | VAI | 0.721 (0.673-0.761) | 63.4 | 71.6 | 79.2 | 53.3 | 1.159 | <0.001 |
|  | HOMA-IR | 0.741 (0.689-0.788) | 67.6 | 71.6 | 80.3 | 56.4 | 2.394 | <0.001 |
|  | METS-IR | 0.797 (0.753-0.837) | 73.6 | 77.9 | 85.1 | 63.3 | 41.495 | <0.001 |
|  | FSI | 0.812 (0.773-0.850) | 76.2 | 73.2 | 83.0 | 64.3 | -1.573 | <0.001 |
|  | FLI | 0.813 (0.773-0.849) | 78.3 | 71.6 | 82.5 | 65.8 | 44.061 | <0.001 |
|  | USFLI | 0.792 (0.748-0.832) | 71.5 | 71.1 | 80.9 | 59.4 | 19.479 | <0.001 |
|  | ZJU | 0.806 (0.764-0.849) | 72.9 | 75.9 | 83.8 | 62.1 | 38.082 | <0.001 |
|  | LAP | 0.793 (0.752-0.834) | 75.6 | 68.2 | 80.3 | 62.0 | 32.333 | <0.001 |
|  | HSI | 0.777 (0.734-0.815) | 67.8 | 76.9 | 83.4 | 58.2 | 36.929 | <0.001 |
|  | NHHR | 0.650 (0.601-0.701) | 66.4 | 58.2 | 73.1 | 50.3 | 2.568 | <0.001 |
|  | NFS | 0.693 (0.644-0.738) | 70.0 | 61.6 | 75.7 | 54.5 | -1.919 | <0.001 |
|  | FIB-4 | 0.582 (0.527-0.634) | 54.7 | 61.2 | 70.7 | 44.1 | 0.941 | 0.060 |
|  | BARD | 0.654 (0.610-0.700) | 73.6 | 61.5 | 76.6 | 57.6 | 1.543 | <0.001 |
| Mexican American | TyG | 0.720 (0.654-0.783) | 64.5 | 70.3 | 79.9 | 51.9 | 8.517 | <0.001 |
|  | TyG-BMI | 0.824 (0.769-0.873) | 75.4 | 75.6 | 85.0 | 62.6 | 243.361 | 0.002 |
|  | TyG-WC | 0.836 (0.781-0.882) | 77.9 | 74.0 | 84.6 | 64.6 | 813.610 | <0.001 |
|  | TyG-WHtR | 0.829 (0.771-0.883) | 76.8 | 75.9 | 85.4 | 64.1 | 4.985 | <0.001 |
|  | TyG-WWI | 0.777 (0.711-0.830) | 60.8 | 85.9 | 88.8 | 54.4 | 97.828 | <0.001 |
|  | VAI | 0.746 (0.684-0.805) | 66.3 | 78.5 | 85.0 | 55.9 | 1.446 | <0.001 |
|  | HOMA-IR | 0.806 (0.747-0.862) | 78.1 | 72.5 | 83.9 | 64.3 | 2.528 | 0.065 |
|  | METS-IR | 0.829 (0.777-0.879) | 72.3 | 86.2 | 90.6 | 62.9 | 43.746 | <0.001 |
|  | FSI | 0.827 (0.768-0.880) | 77.7 | 76.9 | 86.0 | 65.2 | -1.572 | <0.001 |
|  | FLI | 0.833 (0.774-0.885) | 74.7 | 78.7 | 86.6 | 62.9 | 49.474 | <0.001 |
|  | USFLI | 0.834 (0.780-0.887) | 83.8 | 70.3 | 83.8 | 70.3 | 22.578 | <0.001 |
|  | ZJU | 0.822 (0.764-0.875) | 71.2 | 81.9 | 87.8 | 60.8 | 40.632 | 0.001 |
|  | LAP | 0.811 (0.748-0.861) | 80.1 | 69.8 | 83.0 | 65.6 | 32.333 | <0.001 |
|  | HSI | 0.805 (0.744-0.860) | 71.4 | 79.1 | 86.2 | 60.1 | 38.374 | <0.001 |
|  | NHHR | 0.679 (0.612-0.745) | 64.1 | 68.9 | 79.1 | 51.1 | 2.587 | 0.003 |
|  | NFS | 0.687 (0.618-0.752) | 63.9 | 66.5 | 77.8 | 50.1 | -2.044 | 0.002 |
|  | FIB-4 | 0.527 (0.453-0.602) | 52.1 | 63.8 | 72.5 | 42.1 | 0.730 | 0.211 |
|  | BARD | 0.642 (0.570-0.716) | 72.6 | 54.9 | 74.7 | 52.2 | 1.583 | 0.002 |
| Non-Hispanic Black | TyG | 0.698 (0.655-0.743) | 67.6 | 63.9 | 63.3 | 68.2 | 8.120 | <0.001 |
|  | TyG-BMI | 0.813 (0.771-0.847) | 73.7 | 75.7 | 73.6 | 75.8 | 251.130 | <0.001 |
|  | TyG-WC | 0.822 (0.784-0.858) | 79.8 | 73.8 | 73.7 | 79.8 | 813.704 | <0.001 |
|  | TyG-WHtR | 0.807 (0.768-0.842) | 78.5 | 71.7 | 71.9 | 78.4 | 4.842 | <0.001 |
|  | TyG-WWI | 0.748 (0.707-0.789) | 74.4 | 65.8 | 66.7 | 73.6 | 87.637 | <0.001 |
|  | VAI | 0.699 (0.653-0.745) | 64.4 | 69.2 | 65.8 | 67.9 | 1.009 | <0.001 |
|  | HOMA-IR | 0.794 (0.752-0.831) | 74.1 | 72.4 | 71.2 | 75.3 | 2.504 | <0.001 |
|  | METS-IR | 0.805 (0.767-0.839) | 76.0 | 71.6 | 71.2 | 76.4 | 42.398 | <0.001 |
|  | FSI | 0.822 (0.783-0.853) | 76.4 | 73.2 | 72.4 | 77.1 | -1.464 | <0.001 |
|  | FLI | 0.829 (0.794-0.863) | 76.0 | 77.3 | 75.5 | 77.7 | 53.657 | <0.001 |
|  | USFLI | 0.840 (0.806-0.869) | 76.9 | 78.0 | 76.3 | 78.5 | 11.078 | <0.001 |
|  | ZJU | 0.808 (0.768-0.843) | 77.8 | 70.0 | 70.5 | 77.4 | 40.267 | <0.001 |
|  | LAP | 0.791 (0.750-0.831) | 71.3 | 74.8 | 72.2 | 73.9 | 32.620 | <0.001 |
|  | HSI | 0.812 (0.774-0.848) | 79.9 | 68.6 | 70.1 | 78.8 | 37.685 | <0.001 |
|  | NHHR | 0.639 (0.592-0.688) | 66.4 | 60.7 | 60.9 | 66.2 | 2.176 | 0.003 |
|  | NFS | 0.655 (0.606-0.704) | 64.9 | 63.0 | 61.8 | 66.1 | -1.427 | <0.001 |
|  | FIB-4 | 0.514 (0.463-0.558) | 46.5 | 54.2 | 48.3 | 52.4 | 0.786 | 0.207 |
|  | BARD | 0.607 (0.558-0.659) | 72.4 | 47.9 | 56.1 | 65.3 | 1.824 | <0.001 |
| Non-Hispanic White | TyG | 0.758 (0.717-0.797) | 69.8 | 71.4 | 79.0 | 60.6 | 8.418 | <0.001 |
|  | TyG-BMI | 0.819 (0.782-0.855) | 76.5 | 73.9 | 81.9 | 67.1 | 232.727 | <0.001 |
|  | TyG-WC | 0.837 (0.804-0.868) | 80.6 | 73.8 | 82.6 | 71.2 | 811.731 | <0.001 |
|  | TyG-WHtR | 0.826 (0.791-0.859) | 69.5 | 81.4 | 85.2 | 63.4 | 5.066 | <0.001 |
|  | TyG-WWI | 0.794 (0.753-0.830) | 69.3 | 76.2 | 81.8 | 61.7 | 94.029 | <0.001 |
|  | VAI | 0.732 (0.688-0.774) | 68.7 | 69.2 | 77.4 | 58.9 | 1.159 | <0.001 |
|  | HOMA-IR | 0.749 (0.701-0.790) | 67.9 | 69.5 | 77.4 | 58.5 | 2.217 | <0.001 |
|  | METS-IR | 0.806 (0.770-0.842) | 70.3 | 80.8 | 85.0 | 63.9 | 41.495 | <0.001 |
|  | FSI | 0.828 (0.791-0.863) | 75.2 | 76.3 | 83.0 | 66.7 | -1.610 | <0.001 |
|  | FLI | 0.836 (0.801-0.869) | 77.8 | 75.9 | 83.3 | 68.9 | 42.803 | <0.001 |
|  | USFLI | 0.814 (0.775-0.850) | 77.3 | 69.8 | 79.8 | 66.6 | 15.991 | <0.001 |
|  | ZJU | 0.811 (0.772-0.845) | 76.0 | 74.0 | 81.8 | 66.7 | 38.153 | <0.001 |
|  | LAP | 0.813 (0.775-0.848) | 72.1 | 76.3 | 82.4 | 64.0 | 36.937 | <0.001 |
|  | HSI | 0.789 (0.748-0.825) | 72.2 | 74.5 | 81.4 | 63.5 | 36.664 | <0.001 |
|  | NHHR | 0.675 (0.629-0.720) | 62.2 | 66.2 | 73.9 | 53.2 | 2.403 | <0.001 |
|  | NFS | 0.709 (0.667-0.751) | 65.0 | 67.8 | 75.7 | 55.7 | -1.566 | <0.001 |
|  | FIB-4 | 0.580 (0.527-0.628) | 59.3 | 56.6 | 67.8 | 47.5 | 0.941 | 0.024 |
|  | BARD | 0.677 (0.630-0.721) | 73.8 | 62.0 | 74.9 | 60.6 | 1.583 | <0.001 |
| Other Hispanic | TyG | 0.791 (0.729-0.848) | 82.4 | 69.8 | 79.0 | 74.2 | 8.372 | <0.001 |
|  | TyG-BMI | 0.815 (0.752-0.870) | 75.1 | 77.2 | 82.0 | 69.2 | 241.652 | <0.001 |
|  | TyG-WC | 0.811 (0.746-0.871) | 76.0 | 69.7 | 77.6 | 67.7 | 808.711 | <0.001 |
|  | TyG-WHtR | 0.814 (0.751-0.873) | 80.0 | 70.3 | 78.8 | 71.8 | 4.916 | <0.001 |
|  | TyG-WWI | 0.768 (0.703-0.827) | 76.1 | 65.3 | 75.2 | 66.4 | 91.792 | <0.001 |
|  | VAI | 0.765 (0.700-0.823) | 74.9 | 69.0 | 77.0 | 66.6 | 1.210 | <0.001 |
|  | HOMA-IR | 0.815 (0.759-0.867) | 77.6 | 72.2 | 79.4 | 70.0 | 2.282 | <0.001 |
|  | METS-IR | 0.823 (0.766-0.880) | 77.1 | 77.0 | 82.3 | 70.8 | 41.659 | <0.001 |
|  | FSI | 0.825 (0.765-0.879) | 74.3 | 75.8 | 80.9 | 68.1 | -1.514 | <0.001 |
|  | FLI | 0.798 (0.734-0.856) | 75.7 | 67.8 | 76.5 | 66.9 | 41.854 | <0.001 |
|  | USFLI | 0.815 (0.747-0.873) | 71.8 | 76.8 | 81.1 | 66.3 | 21.408 | <0.001 |
|  | ZJU | 0.798 (0.730-0.854) | 74.7 | 73.6 | 79.7 | 67.8 | 39.684 | <0.001 |
|  | LAP | 0.798 (0.733-0.858) | 75.6 | 72.5 | 79.2 | 68.2 | 37.286 | <0.001 |
|  | HSI | 0.780 (0.710-0.841) | 75.7 | 68.1 | 76.7 | 67.0 | 37.733 | <0.001 |
|  | NHHR | 0.701 (0.623-0.770) | 66.9 | 64.9 | 72.5 | 58.6 | 2.523 | <0.001 |
|  | NFS | 0.710 (0.639-0.778) | 66.7 | 70.7 | 75.9 | 60.6 | -1.732 | <0.001 |
|  | FIB-4 | 0.472 (0.379-0.561) | 48.0 | 53.1 | 58.6 | 42.5 | 0.801 | 0.853 |
|  | BARD | 0.640 (0.565-0.712) | 75.5 | 53.1 | 69.0 | 61.1 | 1.515 | 0.001 |
| Other Race | TyG | 0.717 (0.653-0.776) | 70.8 | 62.0 | 70.1 | 62.9 | 8.425 | <0.001 |
|  | TyG-BMI | 0.788 (0.724-0.846) | 73.6 | 75.2 | 78.8 | 69.4 | 223.598 | <0.001 |
|  | TyG-WC | 0.812 (0.747-0.866) | 82.6 | 70.5 | 77.9 | 76.4 | 757.004 | <0.001 |
|  | TyG-WHtR | 0.815 (0.758-0.868) | 75.9 | 74.5 | 78.9 | 71.1 | 4.758 | <0.001 |
|  | TyG-WWI | 0.779 (0.727-0.825) | 69.3 | 70.0 | 74.3 | 64.5 | 93.309 | <0.001 |
|  | VAI | 0.709 (0.644-0.768) | 63.5 | 69.2 | 72.1 | 60.2 | 1.399 | <0.001 |
|  | HOMA-IR | 0.731 (0.659-0.806) | 74.8 | 64.6 | 72.6 | 67.1 | 2.221 | 0.002 |
|  | METS-IR | 0.777 (0.713-0.834) | 77.3 | 68.8 | 75.6 | 70.7 | 37.031 | <0.001 |
|  | FSI | 0.803 (0.743-0.854) | 79.5 | 68.4 | 75.9 | 72.6 | -2.186 | <0.001 |
|  | FLI | 0.794 (0.732-0.847) | 68.5 | 78.6 | 80.1 | 66.6 | 41.639 | <0.001 |
|  | USFLI | 0.786 (0.722-0.839) | 71.5 | 73.6 | 77.2 | 67.3 | 17.571 | <0.001 |
|  | ZJU | 0.791 (0.725-0.848) | 74.3 | 72.7 | 77.3 | 69.3 | 36.964 | <0.001 |
|  | LAP | 0.792 (0.733-0.846) | 71.4 | 74.9 | 78.1 | 67.6 | 31.391 | <0.001 |
|  | HSI | 0.766 (0.707-0.828) | 75.5 | 66.8 | 74.0 | 68.5 | 34.300 | <0.001 |
|  | NHHR | 0.646 (0.584-0.709) | 65.4 | 58.5 | 66.4 | 57.4 | 2.606 | 0.003 |
|  | NFS | 0.696 (0.641-0.749) | 69.3 | 60.9 | 69.0 | 61.3 | -1.997 | <0.001 |
|  | FIB-4 | 0.607 (0.543-0.669) | 71.3 | 50.2 | 64.2 | 58.2 | 0.721 | 0.013 |
|  | BARD | 0.632 (0.569-0.694) | 61.5 | 68.3 | 70.9 | 58.5 | 1.600 | <0.001 |
| Age ≤50 | TyG | 0.740 (0.697-0.780) | 66.4 | 73.1 | 69.4 | 70.4 | 8.369 | <0.001 |
|  | TyG-BMI | 0.824 (0.785-0.861) | 77.7 | 74.4 | 73.6 | 78.4 | 231.826 | <0.001 |
|  | TyG-WC | 0.839 (0.805-0.873) | 76.2 | 80.2 | 77.9 | 78.6 | 811.731 | <0.001 |
|  | TyG-WHtR | 0.828 (0.792-0.864) | 77.2 | 74.5 | 73.5 | 78.1 | 4.695 | <0.001 |
|  | TyG-WWI | 0.791 (0.754-0.829) | 74.2 | 71.7 | 70.6 | 75.2 | 88.511 | <0.001 |
|  | VAI | 0.738 (0.694-0.782) | 67.2 | 70.3 | 67.5 | 70.0 | 1.159 | <0.001 |
|  | HOMA-IR | 0.754 (0.703-0.796) | 68.1 | 72.4 | 69.3 | 71.2 | 2.279 | <0.001 |
|  | METS-IR | 0.819 (0.778-0.858) | 75.6 | 78.3 | 76.1 | 77.7 | 41.497 | <0.001 |
|  | FSI | 0.829 (0.789-0.864) | 76.5 | 75.2 | 73.9 | 77.8 | -1.821 | <0.001 |
|  | FLI | 0.837 (0.799-0.871) | 78.2 | 78.0 | 76.5 | 79.6 | 42.636 | <0.001 |
|  | USFLI | 0.811 (0.770-0.848) | 74.9 | 74.0 | 72.6 | 76.3 | 13.337 | <0.001 |
|  | ZJU | 0.814 (0.774-0.851) | 73.5 | 76.7 | 74.4 | 76.0 | 38.904 | <0.001 |
|  | LAP | 0.816 (0.772-0.849) | 75.3 | 73.3 | 72.1 | 76.4 | 32.333 | <0.001 |
|  | HSI | 0.803 (0.761-0.839) | 75.3 | 73.9 | 72.5 | 76.5 | 36.943 | <0.001 |
|  | NHHR | 0.704 (0.659-0.748) | 67.4 | 64.6 | 63.6 | 68.4 | 2.475 | <0.001 |
|  | NFS | 0.676 (0.631-0.718) | 61.6 | 64.4 | 61.4 | 64.7 | -2.315 | <0.001 |
|  | FIB-4 | 0.510 (0.460-0.554) | 46.5 | 56.3 | 49.4 | 53.4 | 0.583 | 0.624 |
|  | BARD | 0.639 (0.596-0.683) | 71.8 | 60.0 | 62.2 | 69.9 | 1.561 | <0.001 |
| Age >50 | TyG | 0.725 (0.686-0.765) | 68.2 | 64.5 | 82.0 | 46.1 | 8.476 | <0.001 |
|  | TyG-BMI | 0.789 (0.755-0.822) | 76.1 | 69.5 | 85.5 | 55.1 | 232.467 | <0.001 |
|  | TyG-WC | 0.792 (0.753-0.827) | 75.1 | 69.2 | 85.2 | 54.1 | 830.141 | <0.001 |
|  | TyG-WHtR | 0.779 (0.743-0.811) | 72.5 | 70.1 | 85.2 | 51.9 | 5.065 | <0.001 |
|  | TyG-WWI | 0.728 (0.691-0.766) | 71.3 | 67.7 | 83.9 | 49.9 | 95.201 | <0.001 |
|  | VAI | 0.709 (0.668-0.751) | 68.5 | 65.1 | 82.3 | 46.7 | 1.172 | <0.001 |
|  | HOMA-IR | 0.751 (0.711-0.789) | 64.9 | 71.8 | 84.5 | 46.4 | 2.612 | <0.001 |
|  | METS-IR | 0.788 (0.753-0.822) | 67.9 | 78.3 | 88.1 | 50.8 | 41.360 | <0.001 |
|  | FSI | 0.784 (0.747-0.818) | 73.1 | 70.4 | 85.4 | 52.6 | -1.407 | <0.001 |
|  | FLI | 0.795 (0.756-0.831) | 68.6 | 76.6 | 87.4 | 50.8 | 53.128 | <0.001 |
|  | USFLI | 0.786 (0.747-0.820) | 70.2 | 73.4 | 86.2 | 51.0 | 21.564 | <0.001 |
|  | ZJU | 0.782 (0.745-0.817) | 76.9 | 68.5 | 85.2 | 55.6 | 38.153 | <0.001 |
|  | LAP | 0.774 (0.740-0.810) | 68.8 | 74.7 | 86.5 | 50.3 | 39.958 | <0.001 |
|  | HSI | 0.764 (0.726-0.801) | 72.3 | 69.3 | 84.8 | 51.4 | 36.400 | <0.001 |
|  | NHHR | 0.650 (0.603-0.694) | 59.1 | 67.2 | 81.0 | 41.0 | 2.428 | <0.001 |
|  | NFS | 0.618 (0.575-0.661) | 63.8 | 57.0 | 77.8 | 39.9 | -0.776 | <0.001 |
|  | FIB-4 | 0.577 (0.531-0.627) | 54.8 | 60.6 | 76.7 | 36.2 | 1.288 | 0.002 |
|  | BARD | 0.639 (0.589-0.683) | 70.5 | 57.4 | 79.7 | 45.2 | 1.675 | <0.001 |
| Never-smoker | TyG | 0.750 (0.714-0.783) | 70.0 | 68.9 | 74.9 | 63.5 | 8.364 | <0.001 |
|  | TyG-BMI | 0.824 (0.796-0.852) | 78.6 | 73.7 | 79.8 | 72.2 | 232.727 | <0.001 |
|  | TyG-WC | 0.839 (0.813-0.865) | 78.1 | 77.2 | 81.9 | 72.7 | 813.164 | <0.001 |
|  | TyG-WHtR | 0.830 (0.801-0.855) | 74.0 | 76.7 | 80.8 | 69.1 | 4.939 | <0.001 |
|  | TyG-WWI | 0.786 (0.757-0.814) | 69.3 | 72.1 | 76.7 | 64.0 | 92.705 | <0.001 |
|  | VAI | 0.729 (0.688-0.766) | 68.5 | 67.0 | 73.3 | 61.6 | 1.147 | <0.001 |
|  | HOMA-IR | 0.782 (0.747-0.813) | 74.4 | 68.6 | 75.8 | 66.9 | 2.217 | <0.001 |
|  | METS-IR | 0.811 (0.783-0.839) | 72.9 | 77.6 | 81.2 | 68.4 | 41.659 | <0.001 |
|  | FSI | 0.833 (0.807-0.860) | 81.2 | 71.5 | 79.0 | 74.1 | -1.796 | <0.001 |
|  | FLI | 0.834 (0.803-0.860) | 77.8 | 75.3 | 80.7 | 72.0 | 42.778 | <0.001 |
|  | USFLI | 0.835 (0.808-0.862) | 75.0 | 76.0 | 80.5 | 69.7 | 17.780 | <0.001 |
|  | ZJU | 0.821 (0.794-0.848) | 76.1 | 73.9 | 79.4 | 70.1 | 38.899 | <0.001 |
|  | LAP | 0.814 (0.782-0.841) | 71.6 | 75.7 | 79.6 | 66.8 | 35.724 | <0.001 |
|  | HSI | 0.804 (0.774-0.834) | 72.6 | 74.6 | 79.1 | 67.3 | 37.458 | <0.001 |
|  | NHHR | 0.672 (0.632-0.713) | 66.3 | 61.6 | 69.6 | 58.0 | 2.369 | <0.001 |
|  | NFS | 0.693 (0.660-0.727) | 65.7 | 65.6 | 71.7 | 59.1 | -1.728 | <0.001 |
|  | FIB-4 | 0.553 (0.512-0.595) | 57.0 | 55.8 | 63.1 | 49.5 | 0.863 | 0.025 |
|  | BARD | 0.635 (0.597-0.673) | 71.5 | 56.7 | 68.6 | 60.1 | 1.605 | <0.001 |
| Former smoker | TyG | 0.756 (0.694-0.811) | 71.9 | 70.5 | 80.6 | 59.5 | 8.371 | <0.001 |
|  | TyG-BMI | 0.788 (0.724-0.843) | 78.2 | 66.6 | 80.0 | 64.1 | 226.298 | <0.001 |
|  | TyG-WC | 0.806 (0.749-0.858) | 79.2 | 68.2 | 81.0 | 65.7 | 811.731 | <0.001 |
|  | TyG-WHtR | 0.802 (0.744-0.851) | 68.4 | 79.0 | 84.8 | 59.4 | 5.071 | <0.001 |
|  | TyG-WWI | 0.785 (0.729-0.840) | 69.7 | 77.1 | 83.9 | 59.8 | 94.166 | <0.001 |
|  | VAI | 0.755 (0.691-0.811) | 68.4 | 73.8 | 81.7 | 57.7 | 1.179 | <0.001 |
|  | HOMA-IR | 0.720 (0.642-0.791) | 65.0 | 71.3 | 79.5 | 54.4 | 2.484 | <0.001 |
|  | METS-IR | 0.782 (0.714-0.837) | 69.6 | 79.2 | 85.1 | 60.4 | 41.382 | <0.001 |
|  | FSI | 0.797 (0.732-0.853) | 70.8 | 77.2 | 84.2 | 60.7 | -1.391 | <0.001 |
|  | FLI | 0.804 (0.742-0.855) | 75.8 | 71.6 | 82.0 | 63.4 | 43.720 | <0.001 |
|  | USFLI | 0.776 (0.712-0.835) | 66.4 | 72.6 | 80.5 | 55.8 | 21.739 | <0.001 |
|  | ZJU | 0.772 (0.712-0.826) | 72.2 | 72.1 | 81.5 | 60.2 | 38.070 | <0.001 |
|  | LAP | 0.801 (0.744-0.852) | 70.2 | 78.9 | 85.1 | 60.8 | 38.251 | <0.001 |
|  | HSI | 0.749 (0.683-0.805) | 70.2 | 72.5 | 81.4 | 58.8 | 36.400 | <0.001 |
|  | NHHR | 0.678 (0.611-0.739) | 60.6 | 67.0 | 75.9 | 49.9 | 2.434 | <0.001 |
|  | NFS | 0.710 (0.640-0.773) | 76.3 | 56.4 | 75.0 | 58.2 | -1.952 | <0.001 |
|  | FIB-4 | 0.607 (0.528-0.677) | 60.4 | 59.4 | 71.8 | 46.7 | 0.956 | 0.227 |
|  | BARD | 0.686 (0.629-0.752) | 76.9 | 60.9 | 77.1 | 60.6 | 1.543 | <0.001 |
| Current smoker | TyG | 0.710 (0.628-0.788) | 71.6 | 64.4 | 73.9 | 61.7 | 8.398 | 0.001 |
|  | TyG-BMI | 0.810 (0.738-0.871) | 73.0 | 78.5 | 82.7 | 67.4 | 231.826 | <0.001 |
|  | TyG-WC | 0.834 (0.774-0.891) | 81.7 | 73.8 | 81.5 | 74.1 | 803.850 | <0.001 |
|  | TyG-WHtR | 0.823 (0.756-0.885) | 79.7 | 77.3 | 83.2 | 73.0 | 4.858 | <0.001 |
|  | TyG-WWI | 0.776 (0.702-0.846) | 68.4 | 75.7 | 79.9 | 63.0 | 93.279 | <0.001 |
|  | VAI | 0.701 (0.627-0.775) | 67.5 | 67.7 | 74.6 | 59.6 | 1.323 | <0.001 |
|  | HOMA-IR | 0.738 (0.655-0.810) | 81.1 | 58.6 | 73.4 | 68.8 | 1.685 | 0.003 |
|  | METS-IR | 0.803 (0.729-0.866) | 78.5 | 73.7 | 80.8 | 70.9 | 38.569 | <0.001 |
|  | FSI | 0.822 (0.757-0.882) | 69.6 | 81.4 | 84.0 | 65.5 | -1.521 | <0.001 |
|  | FLI | 0.832 (0.770-0.889) | 78.1 | 78.4 | 83.6 | 71.8 | 43.375 | <0.001 |
|  | USFLI | 0.823 (0.758-0.877) | 75.0 | 82.1 | 85.5 | 70.0 | 16.309 | <0.001 |
|  | ZJU | 0.790 (0.724-0.857) | 71.8 | 76.1 | 80.9 | 65.7 | 38.188 | <0.001 |
|  | LAP | 0.803 (0.730-0.875) | 78.5 | 72.0 | 79.8 | 70.4 | 32.504 | <0.001 |
|  | HSI | 0.777 (0.707-0.842) | 71.5 | 72.6 | 78.6 | 64.4 | 35.745 | <0.001 |
|  | NHHR | 0.652 (0.570-0.734) | 69.3 | 61.3 | 71.7 | 58.6 | 2.442 | 0.007 |
|  | NFS | 0.673 (0.586-0.753) | 60.3 | 71.7 | 75.0 | 56.2 | -1.505 | <0.001 |
|  | FIB-4 | 0.506 (0.429-0.588) | 48.4 | 59.1 | 62.5 | 44.8 | 0.776 | 0.581 |
|  | BARD | 0.653 (0.567-0.729) | 67.8 | 64.8 | 73.1 | 58.8 | 1.561 | 0.002 |
| Non-Obese | TyG | 0.715 (0.676-0.753) | 63.9 | 70.0 | 62.3 | 71.4 | 8.369 | <0.001 |
|  | TyG-BMI | 0.761 (0.719-0.798) | 68.7 | 72.5 | 66.0 | 74.9 | 215.118 | <0.001 |
|  | TyG-WC | 0.781 (0.743-0.816) | 68.2 | 77.4 | 70.0 | 75.8 | 780.454 | <0.001 |
|  | TyG-WHtR | 0.767 (0.728-0.801) | 72.4 | 69.9 | 65.1 | 76.5 | 4.520 | <0.001 |
|  | TyG-WWI | 0.738 (0.699-0.774) | 68.1 | 63.0 | 58.8 | 71.8 | 88.844 | <0.001 |
|  | VAI | 0.682 (0.642-0.724) | 69.2 | 60.5 | 57.6 | 71.7 | 0.939 | <0.001 |
|  | HOMA-IR | 0.679 (0.636-0.724) | 68.6 | 58.1 | 56.0 | 70.4 | 1.685 | <0.001 |
|  | METS-IR | 0.732 (0.691-0.771) | 63.7 | 71.0 | 63.0 | 71.6 | 36.770 | <0.001 |
|  | FSI | 0.770 (0.731-0.808) | 72.5 | 72.7 | 67.3 | 77.3 | -2.177 | <0.001 |
|  | FLI | 0.777 (0.737-0.812) | 68.1 | 76.6 | 69.3 | 75.6 | 30.049 | <0.001 |
|  | USFLI | 0.756 (0.718-0.792) | 72.7 | 68.7 | 64.3 | 76.4 | 11.350 | <0.001 |
|  | ZJU | 0.749 (0.707-0.787) | 68.6 | 68.4 | 62.8 | 73.8 | 35.974 | <0.001 |
|  | LAP | 0.756 (0.716-0.794) | 75.9 | 65.1 | 62.8 | 77.7 | 23.484 | <0.001 |
|  | HSI | 0.711 (0.670-0.755) | 64.0 | 67.6 | 60.5 | 70.7 | 33.767 | <0.001 |
|  | NHHR | 0.635 (0.592-0.679) | 55.9 | 66.1 | 56.1 | 65.8 | 2.403 | <0.001 |
|  | NFS | 0.653 (0.610-0.698) | 65.6 | 61.6 | 57.0 | 69.8 | -1.995 | <0.001 |
|  | FIB-4 | 0.615 (0.573-0.657) | 59.4 | 59.0 | 52.9 | 65.2 | 0.944 | <0.001 |
|  | BARD | 0.542 (0.498-0.583) | 46.8 | 67.9 | 53.1 | 62.2 | 1.551 | <0.001 |
| Obese | TyG | 0.728 (0.681-0.775) | 73.8 | 61.2 | 89.4 | 34.4 | 8.425 | <0.001 |
|  | TyG-BMI | 0.723 (0.665-0.771) | 70.0 | 63.9 | 89.6 | 32.3 | 286.863 | <0.001 |
|  | TyG-WC | 0.760 (0.712-0.801) | 69.8 | 75.3 | 92.6 | 35.9 | 951.403 | <0.001 |
|  | TyG-WHtR | 0.744 (0.695-0.786) | 66.4 | 75.8 | 92.5 | 33.6 | 5.757 | <0.001 |
|  | TyG-WWI | 0.732 (0.682-0.780) | 66.2 | 73.3 | 91.7 | 32.7 | 97.642 | <0.001 |
|  | VAI | 0.708 (0.656-0.757) | 63.5 | 69.0 | 90.1 | 29.8 | 1.526 | <0.001 |
|  | HOMA-IR | 0.713 (0.658-0.766) | 57.4 | 72.6 | 90.3 | 27.7 | 4.051 | <0.001 |
|  | METS-IR | 0.723 (0.674-0.771) | 66.7 | 67.3 | 90.1 | 31.2 | 50.452 | <0.001 |
|  | FSI | 0.746 (0.697-0.797) | 70.6 | 67.0 | 90.5 | 33.8 | -0.428 | <0.001 |
|  | FLI | 0.747 (0.697-0.790) | 73.5 | 68.9 | 91.3 | 36.8 | 78.943 | <0.001 |
|  | USFLI | 0.766 (0.715-0.817) | 69.9 | 67.4 | 90.5 | 33.4 | 33.523 | <0.001 |
|  | ZJU | 0.697 (0.644-0.748) | 62.0 | 70.0 | 90.2 | 29.2 | 46.041 | <0.001 |
|  | LAP | 0.741 (0.692-0.786) | 68.1 | 71.3 | 91.4 | 33.4 | 54.573 | <0.001 |
|  | HSI | 0.683 (0.629-0.732) | 68.6 | 62.7 | 89.1 | 30.9 | 43.415 | <0.001 |
|  | NHHR | 0.635 (0.571-0.694) | 69.5 | 53.2 | 86.9 | 28.1 | 2.443 | <0.001 |
|  | NFS | 0.632 (0.572-0.686) | 55.6 | 65.8 | 87.9 | 24.9 | -0.758 | <0.001 |
|  | FIB-4 | 0.551 (0.489-0.616) | 59.9 | 55.1 | 85.6 | 23.6 | 0.776 | 0.446 |
|  | BARD | 0.557 (0.501-0.613) | 54.1 | 60.1 | 85.8 | 22.7 | 2.062 | 0.444 |
| Non-Hypertension | TyG | 0.727 (0.692-0.767) | 65.3 | 72.1 | 69.4 | 68.1 | 8.350 | <0.001 |
|  | TyG-BMI | 0.801 (0.763-0.835) | 75.8 | 73.3 | 73.4 | 75.7 | 227.876 | <0.001 |
|  | TyG-WC | 0.821 (0.788-0.852) | 72.8 | 80.4 | 78.3 | 75.3 | 811.731 | <0.001 |
|  | TyG-WHtR | 0.815 (0.779-0.848) | 75.7 | 73.8 | 73.8 | 75.8 | 4.695 | <0.001 |
|  | TyG-WWI | 0.777 (0.740-0.811) | 74.6 | 66.7 | 68.6 | 73.0 | 88.972 | <0.001 |
|  | VAI | 0.706 (0.661-0.747) | 62.9 | 69.6 | 66.8 | 65.9 | 1.151 | <0.001 |
|  | HOMA-IR | 0.730 (0.683-0.772) | 63.8 | 72.9 | 69.6 | 67.4 | 2.217 | <0.001 |
|  | METS-IR | 0.785 (0.746-0.820) | 68.6 | 77.9 | 75.2 | 71.8 | 40.814 | <0.001 |
|  | FSI | 0.811 (0.775-0.846) | 79.6 | 72.4 | 73.7 | 78.5 | -2.186 | <0.001 |
|  | FLI | 0.815 (0.779-0.849) | 76.5 | 75.5 | 75.2 | 76.8 | 39.367 | <0.001 |
|  | USFLI | 0.796 (0.759-0.833) | 74.9 | 71.5 | 71.9 | 74.5 | 12.902 | <0.001 |
|  | ZJU | 0.792 (0.754-0.826) | 71.9 | 75.4 | 73.9 | 73.4 | 38.222 | <0.001 |
|  | LAP | 0.796 (0.761-0.831) | 79.6 | 66.5 | 69.8 | 77.0 | 26.612 | <0.001 |
|  | HSI | 0.771 (0.736-0.807) | 72.0 | 72.0 | 71.4 | 72.5 | 36.167 | <0.001 |
|  | NHHR | 0.665 (0.621-0.707) | 62.6 | 63.8 | 62.7 | 63.7 | 2.428 | <0.001 |
|  | NFS | 0.667 (0.627-0.711) | 62.6 | 66.3 | 64.4 | 64.6 | -2.009 | <0.001 |
|  | FIB-4 | 0.538 (0.497-0.583) | 45.2 | 63.8 | 54.8 | 54.5 | 0.885 | 0.033 |
|  | BARD | 0.627 (0.582-0.666) | 68.5 | 61.2 | 63.2 | 66.6 | 1.548 | <0.001 |
| Hypertension | TyG | 0.726 (0.683-0.767) | 62.8 | 69.3 | 85.5 | 39.2 | 8.628 | <0.001 |
|  | TyG-BMI | 0.783 (0.745-0.820) | 73.3 | 68.2 | 86.9 | 47.0 | 243.383 | <0.001 |
|  | TyG-WC | 0.798 (0.759-0.835) | 67.0 | 77.6 | 89.6 | 44.9 | 892.839 | <0.001 |
|  | TyG-WHtR | 0.779 (0.739-0.815) | 68.4 | 74.7 | 88.6 | 45.0 | 5.315 | <0.001 |
|  | TyG-WWI | 0.733 (0.686-0.773) | 69.3 | 70.0 | 86.9 | 44.2 | 96.553 | <0.001 |
|  | VAI | 0.734 (0.689-0.776) | 69.4 | 68.6 | 86.4 | 43.7 | 1.339 | <0.001 |
|  | HOMA-IR | 0.756 (0.716-0.798) | 69.9 | 68.5 | 86.5 | 44.1 | 2.819 | <0.001 |
|  | METS-IR | 0.789 (0.752-0.824) | 68.8 | 77.1 | 89.6 | 46.2 | 43.527 | <0.001 |
|  | FSI | 0.783 (0.744-0.823) | 68.5 | 75.3 | 88.9 | 45.3 | -0.825 | <0.001 |
|  | FLI | 0.796 (0.757-0.831) | 68.3 | 76.5 | 89.3 | 45.6 | 62.152 | <0.001 |
|  | USFLI | 0.801 (0.761-0.838) | 76.7 | 72.1 | 88.8 | 51.8 | 21.727 | <0.001 |
|  | ZJU | 0.775 (0.735-0.814) | 74.3 | 70.3 | 87.8 | 48.8 | 39.825 | <0.001 |
|  | LAP | 0.787 (0.750-0.826) | 71.7 | 71.4 | 87.8 | 46.7 | 42.300 | <0.001 |
|  | HSI | 0.763 (0.722-0.803) | 67.7 | 72.8 | 87.8 | 43.9 | 38.729 | <0.001 |
|  | NHHR | 0.674 (0.628-0.720) | 64.1 | 65.7 | 84.3 | 38.9 | 2.415 | <0.001 |
|  | NFS | 0.637 (0.585-0.686) | 59.5 | 62.8 | 82.2 | 35.0 | -0.776 | <0.001 |
|  | FIB-4 | 0.519 (0.462-0.572) | 60.2 | 47.3 | 76.7 | 29.2 | 1.236 | 0.248 |
|  | BARD | 0.627 (0.576-0.676) | 69.1 | 52.5 | 80.7 | 37.1 | 1.756 | <0.001 |

MASLD, metabolic dysfunction-associated steatotic liver disease; TyG: triglyceride-glucose index; BMI: body mass index; WC: waist circumference; WHtR: waist-to-height ratio; WWI: weight-adjusted waist index; VAI: visceral adiposity index; HOMA-IR: homeostatic model assessment of insulin resistance; METS-IR: metabolic score for insulin resistance; FSI: Framingham steatosis index; FLI: fatty liver index; ZJU: Zhejiang University index; LAP: lipid accumulation product; HSI: hepatic steatosis index; NHHR: non-high-density lipoprotein cholesterol (HDL-C) to HDL-C ratio; NFS: nonalcoholic fatty liver disease fibrosis score; FIB-4: fibrosis-4 index; BARD: BMI-aspartate aminotransferase/alanine aminotransferase ratio and diabetes score

**Table. S4**. Diagnostic efficacy of 18 indices for MASLD in patients with normoglycemia across different demographic characteristics

| Group | Variables | AUC (95%CI) | Sensitivity (%) | Specificity (%) | PPV | NPV | Cut-off Values | p-Value |
| --- | --- | --- | --- | --- | --- | --- | --- | --- |
| Female | TyG | 0.740 (0.682-0.794) | 66.5 | 70.2 | 58.1 | 77.2 | 8.130 | <0.001 |
|  | TyG-BMI | 0.827 (0.778-0.868) | 72.1 | 80.8 | 69.9 | 82.4 | 233.056 | <0.001 |
|  | TyG-WC | 0.842 (0.792-0.885) | 71.8 | 81.7 | 70.9 | 82.3 | 780.454 | <0.001 |
|  | TyG-WHtR | 0.848 (0.803-0.889) | 74.6 | 80.8 | 70.6 | 83.7 | 4.857 | <0.001 |
|  | TyG-WWI | 0.804 (0.754-0.852) | 71.4 | 77.1 | 65.9 | 81.3 | 90.728 | <0.001 |
|  | VAI | 0.741 (0.679-0.798) | 65.3 | 73.9 | 60.8 | 77.4 | 1.151 | <0.001 |
|  | HOMA-IR | 0.747 (0.691-0.806) | 65.3 | 73.2 | 60.2 | 77.3 | 1.880 | <0.001 |
|  | METS-IR | 0.806 (0.757-0.851) | 73.0 | 72.4 | 62.1 | 81.2 | 36.997 | <0.001 |
|  | FSI | 0.829 (0.783-0.871) | 76.4 | 78.1 | 68.4 | 84.2 | -1.983 | <0.001 |
|  | FLI | 0.843 (0.796-0.885) | 75.9 | 81.1 | 71.4 | 84.4 | 37.165 | <0.001 |
|  | USFLI | 0.833 (0.789-0.872) | 77.0 | 77.6 | 68.1 | 84.5 | 10.991 | <0.001 |
|  | ZJU | 0.814 (0.766-0.859) | 78.5 | 74.4 | 65.6 | 84.8 | 37.595 | <0.001 |
|  | LAP | 0.833 (0.783-0.876) | 79.6 | 70.4 | 62.5 | 84.7 | 27.081 | <0.001 |
|  | HSI | 0.801 (0.748-0.846) | 73.6 | 77.0 | 66.5 | 82.5 | 36.600 | <0.001 |
|  | NHHR | 0.673 (0.603-0.746) | 57.1 | 73.7 | 57.4 | 73.5 | 2.259 | <0.001 |
|  | NFS | 0.660 (0.593-0.722) | 62.1 | 65.0 | 52.4 | 73.4 | -2.009 | 0.002 |
|  | FIB-4 | 0.531 (0.460-0.601) | 51.0 | 57.0 | 42.4 | 65.2 | 0.811 | 0.340 |
|  | BARD | 0.658 (0.597-0.720) | 65.8 | 70.5 | 58.0 | 76.8 | 1.762 | <0.001 |
| Male | TyG | 0.698 (0.609-0.784) | 74.4 | 64.0 | 59.4 | 77.9 | 8.221 | 0.006 |
|  | TyG-BMI | 0.763 (0.671-0.843) | 69.5 | 76.7 | 67.9 | 78.0 | 227.016 | <0.001 |
|  | TyG-WC | 0.790 (0.707-0.868) | 80.2 | 73.9 | 68.5 | 84.1 | 789.240 | <0.001 |
|  | TyG-WHtR | 0.778 (0.688-0.853) | 75.1 | 78.0 | 70.7 | 81.5 | 4.584 | <0.001 |
|  | TyG-WWI | 0.745 (0.672-0.823) | 69.0 | 76.3 | 67.3 | 77.6 | 88.771 | <0.001 |
|  | VAI | 0.720 (0.631-0.796) | 71.0 | 67.2 | 60.5 | 76.6 | 0.946 | <0.001 |
|  | HOMA-IR | 0.708 (0.619-0.792) | 63.2 | 72.0 | 61.5 | 73.4 | 1.695 | 0.011 |
|  | METS-IR | 0.762 (0.676-0.845) | 70.2 | 74.9 | 66.4 | 78.0 | 39.646 | <0.001 |
|  | FSI | 0.772 (0.682-0.858) | 80.6 | 66.4 | 62.9 | 82.8 | -2.120 | 0.003 |
|  | FLI | 0.790 (0.703-0.863) | 74.6 | 79.4 | 71.9 | 81.6 | 43.722 | <0.001 |
|  | USFLI | 0.768 (0.691-0.846) | 75.4 | 74.7 | 67.8 | 81.1 | 10.899 | 0.008 |
|  | ZJU | 0.752 (0.662-0.830) | 71.9 | 69.4 | 62.4 | 77.7 | 35.751 | <0.001 |
|  | LAP | 0.784 (0.699-0.859) | 76.0 | 74.8 | 68.1 | 81.5 | 29.643 | 0.006 |
|  | HSI | 0.737 (0.647-0.822) | 69.7 | 71.5 | 63.4 | 76.9 | 34.400 | 0.001 |
|  | NHHR | 0.682 (0.590-0.771) | 72.0 | 58.5 | 55.1 | 74.7 | 2.566 | 0.011 |
|  | NFS | 0.576 (0.491-0.662) | 47.5 | 67.6 | 50.9 | 64.5 | -2.115 | 0.025 |
|  | FIB-4 | 0.476 (0.390-0.563) | 39.1 | 63.6 | 43.2 | 59.6 | 0.941 | 0.934 |
|  | BARD | 0.571 (0.484-0.663) | 63.1 | 60.2 | 52.9 | 69.7 | 1.465 | 0.026 |
| Mexican American | TyG | 0.648 (0.511-0.775) | 56.1 | 70.4 | 67.2 | 59.7 | 8.370 | 0.077 |
|  | TyG-BMI | 0.779 (0.660-0.879) | 70.2 | 84.4 | 82.9 | 72.3 | 241.254 | 0.066 |
|  | TyG-WC | 0.806 (0.688-0.903) | 72.6 | 85.2 | 84.2 | 74.2 | 794.320 | 0.026 |
|  | TyG-WHtR | 0.788 (0.678-0.892) | 70.3 | 81.9 | 80.8 | 71.8 | 4.883 | 0.061 |
|  | TyG-WWI | 0.742 (0.618-0.846) | 77.7 | 67.0 | 71.8 | 73.5 | 89.467 | 0.057 |
|  | VAI | 0.751 (0.627-0.856) | 67.2 | 78.1 | 76.9 | 68.8 | 1.332 | 0.006 |
|  | HOMA-IR | 0.765 (0.644-0.862) | 70.3 | 72.4 | 73.4 | 69.3 | 1.912 | 0.008 |
|  | METS-IR | 0.805 (0.689-0.902) | 73.8 | 83.6 | 82.9 | 74.7 | 40.870 | 0.016 |
|  | FSI | 0.777 (0.659-0.883) | 75.2 | 80.8 | 80.9 | 75.1 | -1.744 | 0.059 |
|  | FLI | 0.815 (0.700-0.916) | 74.2 | 88.0 | 87.0 | 75.9 | 43.550 | 0.006 |
|  | USFLI | 0.829 (0.729-0.905) | 81.8 | 77.0 | 79.3 | 79.6 | 15.511 | 0.005 |
|  | ZJU | 0.765 (0.646-0.866) | 76.4 | 68.9 | 72.6 | 73.0 | 37.660 | 0.048 |
|  | LAP | 0.799 (0.691-0.888) | 72.1 | 84.5 | 83.4 | 73.7 | 32.333 | 0.025 |
|  | HSI | 0.736 (0.613-0.852) | 66.9 | 72.2 | 72.2 | 66.8 | 36.418 | 0.024 |
|  | NHHR | 0.692 (0.556-0.810) | 64.8 | 70.0 | 70.1 | 64.8 | 2.412 | 0.023 |
|  | NFS | 0.586 (0.453-0.721) | 52.4 | 70.4 | 65.7 | 57.7 | -2.227 | 0.335 |
|  | FIB-4 | 0.579 (0.439-0.705) | 51.6 | 64.6 | 61.2 | 55.3 | 0.585 | 0.252 |
|  | BARD | 0.537 (0.397-0.688) | 62.7 | 61.2 | 63.6 | 60.2 | 1.583 | 0.652 |
| Non-Hispanic Black | TyG | 0.696 (0.624-0.765) | 60.5 | 68.1 | 49.2 | 77.2 | 8.021 | <0.001 |
|  | TyG-BMI | 0.820 (0.758-0.872) | 71.4 | 82.3 | 67.2 | 84.9 | 251.130 | <0.001 |
|  | TyG-WC | 0.829 (0.768-0.885) | 76.0 | 80.2 | 66.1 | 86.7 | 803.850 | <0.001 |
|  | TyG-WHtR | 0.808 (0.747-0.862) | 78.5 | 74.4 | 60.9 | 87.1 | 4.606 | <0.001 |
|  | TyG-WWI | 0.748 (0.685-0.817) | 73.1 | 68.9 | 54.6 | 83.4 | 85.336 | <0.001 |
|  | VAI | 0.717 (0.647-0.781) | 61.4 | 75.1 | 55.7 | 79.2 | 0.946 | 0.007 |
|  | HOMA-IR | 0.755 (0.682-0.822) | 67.4 | 71.4 | 54.6 | 81.1 | 2.095 | <0.001 |
|  | METS-IR | 0.811 (0.748-0.868) | 69.4 | 80.6 | 64.6 | 83.8 | 43.476 | <0.001 |
|  | FSI | 0.814 (0.758-0.869) | 79.0 | 71.5 | 58.6 | 87.0 | -1.842 | <0.001 |
|  | FLI | 0.835 (0.774-0.886) | 76.6 | 78.0 | 64.0 | 86.7 | 47.984 | <0.001 |
|  | USFLI | 0.815 (0.752-0.869) | 67.1 | 85.5 | 70.3 | 83.6 | 10.214 | <0.001 |
|  | ZJU | 0.805 (0.739-0.861) | 78.2 | 72.1 | 58.8 | 86.6 | 38.789 | <0.001 |
|  | LAP | 0.800 (0.735-0.859) | 77.9 | 76.5 | 62.8 | 87.2 | 27.428 | <0.001 |
|  | HSI | 0.810 (0.742-0.864) | 77.8 | 74.0 | 60.4 | 86.8 | 37.685 | <0.001 |
|  | NHHR | 0.702 (0.630-0.766) | 67.5 | 67.6 | 51.5 | 80.3 | 2.214 | 0.015 |
|  | NFS | 0.549 (0.475-0.629) | 57.2 | 48.8 | 36.3 | 69.1 | -2.274 | 0.228 |
|  | FIB-4 | 0.564 (0.477-0.646) | 57.5 | 54.8 | 39.4 | 71.7 | 0.693 | 0.157 |
|  | BARD | 0.584 (0.503-0.658) | 67.6 | 54.1 | 42.9 | 76.6 | 1.838 | 0.038 |
| Non-Hispanic White | TyG | 0.732 (0.654-0.802) | 70.1 | 71.2 | 61.4 | 78.4 | 8.221 | <0.001 |
|  | TyG-BMI | 0.799 (0.730-0.857) | 76.9 | 74.6 | 66.5 | 83.2 | 216.064 | <0.001 |
|  | TyG-WC | 0.821 (0.758-0.879) | 75.4 | 78.0 | 69.2 | 82.9 | 789.240 | <0.001 |
|  | TyG-WHtR | 0.822 (0.757-0.878) | 78.9 | 74.4 | 66.8 | 84.3 | 4.584 | <0.001 |
|  | TyG-WWI | 0.792 (0.724-0.854) | 76.5 | 71.8 | 64.0 | 82.3 | 88.740 | <0.001 |
|  | VAI | 0.723 (0.648-0.798) | 61.5 | 76.9 | 63.6 | 75.3 | 1.157 | <0.001 |
|  | HOMA-IR | 0.706 (0.635-0.774) | 66.0 | 66.7 | 56.5 | 75.0 | 1.469 | 0.013 |
|  | METS-IR | 0.778 (0.708-0.842) | 79.2 | 67.8 | 61.6 | 83.3 | 35.505 | <0.001 |
|  | FSI | 0.804 (0.742-0.861) | 78.2 | 74.5 | 66.7 | 83.9 | -2.120 | <0.001 |
|  | FLI | 0.825 (0.757-0.880) | 80.1 | 75.8 | 68.4 | 85.3 | 31.350 | <0.001 |
|  | USFLI | 0.800 (0.735-0.857) | 77.5 | 74.6 | 66.6 | 83.5 | 10.899 | 0.002 |
|  | ZJU | 0.789 (0.721-0.857) | 69.9 | 80.4 | 70.0 | 80.3 | 37.595 | <0.001 |
|  | LAP | 0.815 (0.750-0.874) | 67.6 | 83.7 | 73.1 | 79.8 | 35.724 | <0.001 |
|  | HSI | 0.775 (0.704-0.839) | 75.5 | 73.1 | 64.7 | 82.0 | 34.400 | <0.001 |
|  | NHHR | 0.648 (0.563-0.734) | 61.0 | 69.2 | 56.4 | 73.0 | 2.378 | <0.001 |
|  | NFS | 0.627 (0.551-0.701) | 59.3 | 66.2 | 53.4 | 71.3 | -2.003 | 0.009 |
|  | FIB-4 | 0.522 (0.436-0.614) | 49.5 | 59.7 | 44.6 | 64.4 | 0.890 | 0.284 |
|  | BARD | 0.642 (0.563-0.718) | 68.1 | 64.6 | 55.7 | 75.6 | 1.596 | 0.002 |
| Other Hispanic | TyG | 0.760 (0.643-0.863) | 77.7 | 76.1 | 69.6 | 82.9 | 8.372 | 0.001 |
|  | TyG-BMI | 0.831 (0.719-0.920) | 71.3 | 85.1 | 77.2 | 80.8 | 243.010 | 0.002 |
|  | TyG-WC | 0.820 (0.709-0.912) | 78.5 | 76.6 | 70.3 | 83.5 | 778.429 | 0.007 |
|  | TyG-WHtR | 0.822 (0.710-0.916) | 82.5 | 72.1 | 67.6 | 85.4 | 4.652 | 0.005 |
|  | TyG-WWI | 0.733 (0.613-0.838) | 69.7 | 68.7 | 61.1 | 76.3 | 90.634 | 0.012 |
|  | VAI | 0.758 (0.644-0.852) | 77.0 | 72.7 | 66.6 | 81.8 | 1.224 | 0.004 |
|  | HOMA-IR | 0.779 (0.667-0.883) | 66.6 | 74.4 | 64.7 | 76.0 | 1.855 | 0.025 |
|  | METS-IR | 0.842 (0.743-0.921) | 73.6 | 84.6 | 77.2 | 82.0 | 41.947 | 0.002 |
|  | FSI | 0.846 (0.738-0.933) | 79.1 | 78.8 | 72.5 | 84.3 | -1.714 | 0.001 |
|  | FLI | 0.800 (0.673-0.906) | 80.9 | 71.4 | 66.6 | 84.2 | 33.008 | 0.003 |
|  | USFLI | 0.788 (0.666-0.891) | 66.0 | 81.0 | 71.0 | 77.2 | 17.268 | 0.004 |
|  | ZJU | 0.780 (0.658-0.887) | 76.9 | 66.6 | 61.9 | 80.3 | 37.853 | 0.007 |
|  | LAP | 0.818 (0.695-0.908) | 73.1 | 75.7 | 68.0 | 80.0 | 37.286 | 0.003 |
|  | HSI | 0.752 (0.623-0.860) | 68.1 | 71.7 | 62.9 | 76.1 | 37.733 | 0.012 |
|  | NHHR | 0.688 (0.557-0.811) | 63.2 | 72.7 | 62.0 | 73.7 | 2.541 | 0.012 |
|  | NFS | 0.776 (0.669-0.874) | 79.7 | 73.2 | 67.7 | 83.7 | -1.976 | 0.001 |
|  | FIB-4 | 0.575 (0.427-0.725) | 71.1 | 51.3 | 50.7 | 71.6 | 0.684 | 0.678 |
|  | BARD | 0.658 (0.523-0.791) | 70.9 | 58.0 | 54.3 | 73.9 | 1.613 | 0.023 |
| Other Race | TyG | 0.736 (0.637-0.823) | 66.3 | 75.2 | 60.1 | 79.8 | 8.446 | 0.001 |
|  | TyG-BMI | 0.846 (0.766-0.911) | 70.3 | 89.4 | 78.8 | 84.2 | 223.770 | <0.001 |
|  | TyG-WC | 0.844 (0.764-0.917) | 77.7 | 82.5 | 71.4 | 86.8 | 727.941 | <0.001 |
|  | TyG-WHtR | 0.845 (0.764-0.912) | 81.1 | 75.6 | 65.2 | 87.6 | 4.244 | <0.001 |
|  | TyG-WWI | 0.771 (0.673-0.854) | 76.7 | 66.4 | 56.3 | 83.5 | 85.992 | <0.001 |
|  | VAI | 0.767 (0.680-0.853) | 66.5 | 80.2 | 65.4 | 80.9 | 1.467 | 0.002 |
|  | HOMA-IR | 0.826 (0.747-0.889) | 73.9 | 80.8 | 68.5 | 84.6 | 2.035 | <0.001 |
|  | METS-IR | 0.836 (0.754-0.900) | 72.8 | 80.9 | 68.3 | 84.1 | 36.485 | <0.001 |
|  | FSI | 0.835 (0.738-0.903) | 72.5 | 87.6 | 76.7 | 84.9 | -2.186 | <0.001 |
|  | FLI | 0.838 (0.759-0.903) | 78.2 | 79.9 | 68.7 | 86.7 | 22.410 | <0.001 |
|  | USFLI | 0.856 (0.785-0.917) | 87.2 | 78.4 | 69.5 | 91.6 | 10.014 | 0.003 |
|  | ZJU | 0.840 (0.767-0.900) | 78.3 | 77.3 | 66.0 | 86.4 | 34.873 | <0.001 |
|  | LAP | 0.846 (0.766-0.911) | 73.7 | 84.2 | 72.5 | 85.1 | 30.521 | <0.001 |
|  | HSI | 0.813 (0.727-0.880) | 75.9 | 77.8 | 65.9 | 85.1 | 33.200 | <0.001 |
|  | NHHR | 0.726 (0.630-0.817) | 79.8 | 60.4 | 53.2 | 84.2 | 2.369 | <0.001 |
|  | NFS | 0.701 (0.587-0.792) | 72.3 | 64.8 | 53.6 | 80.6 | -2.610 | 0.002 |
|  | FIB-4 | 0.581 (0.465-0.690) | 62.6 | 58.6 | 46.0 | 73.5 | 0.714 | 0.234 |
|  | BARD | 0.620 (0.506-0.728) | 49.0 | 81.3 | 59.6 | 73.9 | 1.629 | 0.080 |
| Age ≤50 | TyG | 0.723 (0.657-0.787) | 68.9 | 67.3 | 52.4 | 80.6 | 8.116 | <0.001 |
|  | TyG-BMI | 0.805 (0.743-0.856) | 72.1 | 77.4 | 62.4 | 84.2 | 227.659 | <0.001 |
|  | TyG-WC | 0.828 (0.778-0.878) | 77.6 | 82.0 | 69.2 | 87.5 | 777.579 | <0.001 |
|  | TyG-WHtR | 0.814 (0.757-0.869) | 77.6 | 77.2 | 64.0 | 86.9 | 4.579 | <0.001 |
|  | TyG-WWI | 0.773 (0.716-0.827) | 74.2 | 73.6 | 59.4 | 84.5 | 85.992 | <0.001 |
|  | VAI | 0.742 (0.671-0.803) | 72.2 | 68.0 | 54.1 | 82.4 | 0.969 | <0.001 |
|  | HOMA-IR | 0.742 (0.679-0.806) | 70.0 | 69.8 | 54.7 | 81.7 | 1.783 | <0.001 |
|  | METS-IR | 0.803 (0.745-0.858) | 73.2 | 76.4 | 61.8 | 84.5 | 39.646 | <0.001 |
|  | FSI | 0.808 (0.748-0.859) | 77.7 | 77.3 | 64.1 | 86.9 | -2.174 | <0.001 |
|  | FLI | 0.826 (0.778-0.872) | 78.5 | 79.1 | 66.2 | 87.6 | 31.350 | <0.001 |
|  | USFLI | 0.811 (0.754-0.859) | 74.2 | 80.3 | 66.3 | 85.7 | 10.899 | 0.001 |
|  | ZJU | 0.784 (0.728-0.840) | 69.6 | 76.7 | 60.9 | 82.9 | 37.549 | <0.001 |
|  | LAP | 0.814 (0.751-0.867) | 78.7 | 75.4 | 62.5 | 87.2 | 26.374 | <0.001 |
|  | HSI | 0.771 (0.705-0.832) | 66.5 | 78.9 | 62.2 | 81.9 | 36.538 | <0.001 |
|  | NHHR | 0.712 (0.646-0.774) | 67.5 | 69.6 | 53.7 | 80.4 | 2.387 | <0.001 |
|  | NFS | 0.582 (0.511-0.647) | 58.6 | 56.5 | 41.3 | 72.4 | -2.554 | 0.011 |
|  | FIB-4 | 0.572 (0.508-0.640) | 54.7 | 59.9 | 41.6 | 71.7 | 0.576 | 0.200 |
|  | BARD | 0.593 (0.525-0.659) | 66.2 | 60.9 | 46.9 | 77.6 | 1.550 | 0.006 |
| Age >50 | TyG | 0.683 (0.596-0.769) | 72.0 | 61.0 | 65.9 | 67.6 | 8.223 | 0.002 |
|  | TyG-BMI | 0.794 (0.725-0.856) | 75.1 | 75.1 | 75.9 | 74.2 | 224.055 | 0.001 |
|  | TyG-WC | 0.777 (0.699-0.842) | 67.7 | 79.7 | 77.7 | 70.2 | 815.660 | 0.002 |
|  | TyG-WHtR | 0.797 (0.728-0.859) | 72.4 | 74.9 | 75.1 | 72.2 | 4.910 | <0.001 |
|  | TyG-WWI | 0.740 (0.661-0.808) | 68.4 | 77.5 | 76.1 | 70.2 | 93.938 | <0.001 |
|  | VAI | 0.688 (0.602-0.775) | 65.0 | 65.8 | 66.5 | 64.3 | 1.092 | <0.001 |
|  | HOMA-IR | 0.721 (0.642-0.793) | 57.5 | 78.0 | 73.2 | 63.7 | 1.891 | 0.010 |
|  | METS-IR | 0.774 (0.699-0.834) | 78.8 | 65.6 | 70.5 | 74.7 | 35.505 | <0.001 |
|  | FSI | 0.770 (0.696-0.831) | 75.0 | 68.7 | 71.5 | 72.4 | -1.793 | <0.001 |
|  | FLI | 0.796 (0.722-0.854) | 75.4 | 75.1 | 76.0 | 74.5 | 40.920 | <0.001 |
|  | USFLI | 0.769 (0.700-0.835) | 79.3 | 67.7 | 71.9 | 75.8 | 11.418 | 0.011 |
|  | ZJU | 0.794 (0.718-0.855) | 76.2 | 76.9 | 77.5 | 75.6 | 37.398 | <0.001 |
|  | LAP | 0.776 (0.704-0.839) | 70.8 | 75.9 | 75.4 | 71.3 | 35.724 | 0.018 |
|  | HSI | 0.785 (0.706-0.848) | 75.7 | 73.0 | 74.5 | 74.2 | 35.308 | <0.001 |
|  | NHHR | 0.581 (0.484-0.681) | 56.6 | 65.6 | 63.2 | 59.1 | 2.378 | 0.088 |
|  | NFS | 0.576 (0.489-0.655) | 62.0 | 54.3 | 58.6 | 57.8 | -1.175 | 0.095 |
|  | FIB-4 | 0.574 (0.484-0.667) | 54.1 | 57.3 | 57.0 | 54.5 | 1.330 | 0.342 |
|  | BARD | 0.661 (0.579-0.732) | 71.0 | 62.7 | 66.6 | 67.4 | 1.615 | 0.002 |
| Never-smoker | TyG | 0.738 (0.680-0.793) | 65.0 | 73.2 | 61.4 | 76.1 | 8.228 | <0.001 |
|  | TyG-BMI | 0.841 (0.789-0.882) | 76.4 | 79.7 | 71.2 | 83.7 | 228.313 | <0.001 |
|  | TyG-WC | 0.851 (0.807-0.890) | 77.5 | 81.5 | 73.4 | 84.7 | 780.454 | <0.001 |
|  | TyG-WHtR | 0.847 (0.802-0.886) | 81.7 | 77.0 | 70.0 | 86.5 | 4.584 | <0.001 |
|  | TyG-WWI | 0.792 (0.741-0.839) | 77.0 | 71.2 | 63.7 | 82.5 | 87.388 | <0.001 |
|  | VAI | 0.747 (0.687-0.803) | 72.6 | 68.5 | 60.2 | 79.2 | 0.969 | <0.001 |
|  | HOMA-IR | 0.762 (0.705-0.816) | 65.6 | 75.7 | 63.9 | 77.1 | 1.995 | <0.001 |
|  | METS-IR | 0.824 (0.777-0.869) | 66.5 | 86.8 | 76.8 | 79.8 | 41.763 | <0.001 |
|  | FSI | 0.842 (0.791-0.885) | 82.5 | 77.6 | 70.7 | 87.1 | -2.145 | <0.001 |
|  | FLI | 0.854 (0.808-0.893) | 80.7 | 80.5 | 73.1 | 86.4 | 34.707 | <0.001 |
|  | USFLI | 0.838 (0.794-0.878) | 77.3 | 78.1 | 69.9 | 84.0 | 10.918 | <0.001 |
|  | ZJU | 0.832 (0.785-0.871) | 77.0 | 77.9 | 69.6 | 83.8 | 37.595 | <0.001 |
|  | LAP | 0.838 (0.790-0.877) | 82.7 | 72.9 | 66.7 | 86.5 | 27.055 | <0.001 |
|  | HSI | 0.826 (0.780-0.866) | 70.9 | 81.2 | 71.2 | 81.0 | 36.667 | <0.001 |
|  | NHHR | 0.699 (0.634-0.768) | 64.3 | 72.4 | 60.4 | 75.6 | 2.349 | <0.001 |
|  | NFS | 0.627 (0.560-0.692) | 57.3 | 68.9 | 54.7 | 71.1 | -2.009 | <0.001 |
|  | FIB-4 | 0.513 (0.446-0.579) | 38.4 | 69.8 | 45.5 | 63.3 | 0.571 | 0.958 |
|  | BARD | 0.618 (0.557-0.685) | 68.9 | 60.3 | 53.2 | 74.7 | 1.593 | 0.002 |
| Former smoker | TyG | 0.730 (0.596-0.836) | 68.1 | 77.4 | 61.4 | 82.1 | 8.339 | 0.011 |
|  | TyG-BMI | 0.696 (0.565-0.805) | 65.2 | 69.9 | 53.4 | 79.2 | 225.049 | 0.010 |
|  | TyG-WC | 0.746 (0.628-0.847) | 64.7 | 78.3 | 61.2 | 80.7 | 816.670 | 0.004 |
|  | TyG-WHtR | 0.771 (0.647-0.861) | 63.8 | 89.0 | 75.5 | 82.3 | 5.059 | 0.003 |
|  | TyG-WWI | 0.803 (0.697-0.890) | 73.4 | 88.0 | 76.4 | 86.2 | 93.158 | 0.002 |
|  | VAI | 0.737 (0.600-0.841) | 68.5 | 77.3 | 61.5 | 82.3 | 1.157 | 0.001 |
|  | HOMA-IR | 0.715 (0.584-0.828) | 66.7 | 72.8 | 56.4 | 80.5 | 1.555 | 0.108 |
|  | METS-IR | 0.668 (0.541-0.779) | 60.5 | 74.0 | 55.2 | 78.0 | 39.842 | 0.018 |
|  | FSI | 0.704 (0.590-0.818) | 60.3 | 79.4 | 60.7 | 79.1 | -1.688 | 0.013 |
|  | FLI | 0.729 (0.607-0.835) | 65.2 | 80.5 | 63.9 | 81.4 | 48.497 | 0.004 |
|  | USFLI | 0.769 (0.665-0.858) | 76.4 | 75.7 | 62.5 | 85.8 | 12.245 | 0.059 |
|  | ZJU | 0.679 (0.559-0.787) | 64.6 | 73.1 | 55.9 | 79.6 | 37.449 | 0.020 |
|  | LAP | 0.783 (0.664-0.870) | 66.1 | 86.6 | 72.3 | 82.8 | 39.958 | 0.001 |
|  | HSI | 0.658 (0.533-0.776) | 64.1 | 72.3 | 55.1 | 79.2 | 35.904 | 0.037 |
|  | NHHR | 0.594 (0.445-0.732) | 56.8 | 66.7 | 47.5 | 74.5 | 2.475 | 0.147 |
|  | NFS | 0.647 (0.522-0.767) | 64.8 | 56.1 | 43.8 | 75.1 | -2.259 | 0.023 |
|  | FIB-4 | 0.607 (0.458-0.726) | 57.8 | 57.3 | 41.7 | 71.9 | 0.888 | 0.041 |
|  | BARD | 0.626 (0.502-0.745) | 66.7 | 65.0 | 50.2 | 78.7 | 1.700 | 0.033 |
| Current smoker | TyG | 0.634 (0.479-0.795) | 62.4 | 66.0 | 62.5 | 65.9 | 8.327 | 0.060 |
|  | TyG-BMI | 0.782 (0.658-0.897) | 74.3 | 75.6 | 73.4 | 76.4 | 217.269 | <0.001 |
|  | TyG-WC | 0.795 (0.659-0.898) | 71.4 | 82.1 | 78.3 | 76.0 | 803.850 | <0.001 |
|  | TyG-WHtR | 0.771 (0.629-0.885) | 67.8 | 78.9 | 74.5 | 73.0 | 4.709 | <0.001 |
|  | TyG-WWI | 0.693 (0.535-0.835) | 66.0 | 72.4 | 68.5 | 70.1 | 88.844 | 0.003 |
|  | VAI | 0.652 (0.493-0.801) | 68.0 | 58.8 | 60.0 | 66.9 | 1.019 | 0.005 |
|  | HOMA-IR | 0.624 (0.498-0.761) | 63.2 | 63.3 | 61.0 | 65.5 | 1.449 | 0.208 |
|  | METS-IR | 0.781 (0.655-0.882) | 77.2 | 79.9 | 77.7 | 79.5 | 38.649 | <0.001 |
|  | FSI | 0.780 (0.632-0.897) | 74.8 | 76.7 | 74.4 | 77.1 | -1.952 | <0.001 |
|  | FLI | 0.799 (0.677-0.898) | 74.6 | 76.3 | 74.1 | 76.8 | 37.165 | <0.001 |
|  | USFLI | 0.751 (0.624-0.869) | 72.7 | 74.4 | 72.1 | 75.1 | 10.497 | 0.014 |
|  | ZJU | 0.749 (0.611-0.864) | 79.4 | 61.6 | 65.2 | 76.8 | 33.889 | <0.001 |
|  | LAP | 0.755 (0.617-0.872) | 64.8 | 77.5 | 72.4 | 70.8 | 32.504 | <0.001 |
|  | HSI | 0.715 (0.567-0.853) | 64.2 | 73.1 | 68.4 | 69.2 | 34.433 | 0.002 |
|  | NHHR | 0.644 (0.480-0.789) | 66.3 | 71.7 | 68.0 | 70.1 | 2.710 | 0.059 |
|  | NFS | 0.628 (0.493-0.755) | 49.6 | 72.3 | 61.9 | 61.2 | -1.866 | 0.078 |
|  | FIB-4 | 0.520 (0.385-0.662) | 44.9 | 68.1 | 56.1 | 57.7 | 0.595 | 0.365 |
|  | BARD | 0.631 (0.495-0.763) | 59.6 | 73.0 | 66.7 | 66.6 | 1.682 | 0.068 |
| Non-Obese | TyG | 0.688 (0.612-0.756) | 62.8 | 71.9 | 47.0 | 83.0 | 8.223 | <0.001 |
|  | TyG-BMI | 0.753 (0.685-0.814) | 66.1 | 73.1 | 49.4 | 84.5 | 206.114 | <0.001 |
|  | TyG-WC | 0.771 (0.705-0.828) | 70.8 | 70.6 | 48.9 | 85.9 | 715.445 | <0.001 |
|  | TyG-WHtR | 0.766 (0.691-0.827) | 73.2 | 69.9 | 49.1 | 86.8 | 4.243 | <0.001 |
|  | TyG-WWI | 0.735 (0.670-0.802) | 71.1 | 68.8 | 47.4 | 85.7 | 85.658 | <0.001 |
|  | VAI | 0.679 (0.609-0.749) | 60.2 | 69.5 | 44.0 | 81.5 | 1.003 | <0.001 |
|  | HOMA-IR | 0.663 (0.600-0.729) | 66.8 | 57.8 | 38.6 | 81.5 | 1.255 | <0.001 |
|  | METS-IR | 0.728 (0.661-0.785) | 62.7 | 74.0 | 48.9 | 83.3 | 35.342 | <0.001 |
|  | FSI | 0.748 (0.682-0.811) | 67.7 | 72.9 | 49.8 | 85.0 | -2.450 | <0.001 |
|  | FLI | 0.776 (0.712-0.834) | 64.9 | 77.2 | 53.0 | 84.7 | 22.410 | <0.001 |
|  | USFLI | 0.764 (0.706-0.819) | 67.6 | 76.3 | 53.1 | 85.6 | 8.414 | <0.001 |
|  | ZJU | 0.739 (0.667-0.800) | 76.1 | 61.3 | 43.9 | 86.6 | 33.883 | <0.001 |
|  | LAP | 0.757 (0.689-0.823) | 74.2 | 68.1 | 48.0 | 86.9 | 20.555 | <0.001 |
|  | HSI | 0.717 (0.642-0.782) | 63.5 | 70.4 | 46.0 | 83.0 | 33.191 | <0.001 |
|  | NHHR | 0.612 (0.529-0.694) | 52.4 | 71.1 | 41.8 | 79.0 | 2.378 | 0.001 |
|  | NFS | 0.606 (0.535-0.679) | 50.3 | 69.9 | 39.9 | 78.0 | -1.995 | 0.005 |
|  | FIB-4 | 0.588 (0.520-0.655) | 53.3 | 60.7 | 35.0 | 76.6 | 0.888 | 0.026 |
|  | BARD | 0.502 (0.417-0.584) | 39.1 | 73.8 | 37.2 | 75.3 | 1.607 | 0.202 |
| Obese | TyG | 0.726 (0.640-0.801) | 64.0 | 76.5 | 85.1 | 50.4 | 8.314 | <0.001 |
|  | TyG-BMI | 0.729 (0.646-0.807) | 70.7 | 63.4 | 80.1 | 50.9 | 273.522 | <0.001 |
|  | TyG-WC | 0.749 (0.671-0.823) | 71.5 | 69.3 | 82.9 | 53.8 | 893.771 | <0.001 |
|  | TyG-WHtR | 0.750 (0.673-0.824) | 70.6 | 65.6 | 81.1 | 51.7 | 5.377 | <0.001 |
|  | TyG-WWI | 0.732 (0.637-0.804) | 68.1 | 72.0 | 83.5 | 51.9 | 94.336 | <0.001 |
|  | VAI | 0.738 (0.660-0.814) | 72.9 | 67.3 | 82.3 | 54.4 | 1.151 | <0.001 |
|  | HOMA-IR | 0.639 (0.538-0.750) | 66.1 | 61.1 | 78.0 | 46.4 | 2.597 | 0.061 |
|  | METS-IR | 0.706 (0.612-0.784) | 64.9 | 69.6 | 81.7 | 48.7 | 49.081 | <0.001 |
|  | FSI | 0.741 (0.643-0.819) | 69.0 | 68.2 | 81.9 | 51.3 | -0.668 | <0.001 |
|  | FLI | 0.748 (0.666-0.824) | 65.8 | 77.7 | 86.1 | 52.1 | 79.142 | <0.001 |
|  | USFLI | 0.723 (0.625-0.830) | 70.7 | 69.0 | 82.6 | 53.0 | 20.286 | 0.026 |
|  | ZJU | 0.678 (0.587-0.766) | 70.3 | 59.2 | 78.2 | 48.8 | 43.175 | <0.001 |
|  | LAP | 0.766 (0.686-0.838) | 73.7 | 70.5 | 83.9 | 56.2 | 44.377 | 0.003 |
|  | HSI | 0.664 (0.573-0.749) | 60.7 | 70.5 | 81.1 | 46.2 | 43.733 | <0.001 |
|  | NHHR | 0.634 (0.541-0.731) | 71.2 | 56.7 | 77.4 | 48.5 | 2.443 | 0.035 |
|  | NFS | 0.559 (0.449-0.649) | 62.0 | 53.3 | 73.5 | 40.2 | -2.053 | 0.215 |
|  | FIB-4 | 0.502 (0.401-0.599) | 44.3 | 63.7 | 71.9 | 35.4 | 0.679 | 0.733 |
|  | BARD | 0.622 (0.530-0.731) | 69.4 | 53.7 | 75.8 | 45.7 | 2.071 | 0.018 |
| Non-Hypertension | TyG | 0.708 (0.640-0.766) | 63.4 | 73.3 | 56.7 | 78.4 | 8.228 | <0.001 |
|  | TyG-BMI | 0.790 (0.733-0.841) | 69.0 | 78.7 | 64.1 | 82.2 | 227.659 | <0.001 |
|  | TyG-WC | 0.811 (0.755-0.859) | 73.6 | 79.8 | 66.7 | 84.6 | 780.454 | <0.001 |
|  | TyG-WHtR | 0.816 (0.762-0.866) | 77.0 | 77.3 | 65.1 | 85.9 | 4.584 | <0.001 |
|  | TyG-WWI | 0.778 (0.724-0.827) | 71.6 | 75.4 | 61.6 | 82.8 | 88.627 | <0.001 |
|  | VAI | 0.716 (0.650-0.777) | 60.4 | 77.5 | 59.6 | 78.0 | 1.151 | <0.001 |
|  | HOMA-IR | 0.731 (0.673-0.793) | 70.3 | 65.5 | 52.9 | 80.0 | 1.526 | <0.001 |
|  | METS-IR | 0.775 (0.721-0.826) | 76.3 | 66.3 | 55.5 | 83.5 | 35.505 | <0.001 |
|  | FSI | 0.797 (0.742-0.844) | 72.6 | 79.4 | 66.0 | 84.0 | -2.174 | <0.001 |
|  | FLI | 0.809 (0.756-0.855) | 76.0 | 77.6 | 65.1 | 85.4 | 31.656 | <0.001 |
|  | USFLI | 0.805 (0.751-0.851) | 74.2 | 78.0 | 65.0 | 84.6 | 10.991 | 0.001 |
|  | ZJU | 0.778 (0.723-0.829) | 68.1 | 78.3 | 63.3 | 81.7 | 37.549 | <0.001 |
|  | LAP | 0.800 (0.737-0.853) | 75.9 | 73.3 | 61.0 | 84.7 | 26.612 | <0.001 |
|  | HSI | 0.764 (0.703-0.824) | 65.2 | 80.2 | 64.4 | 80.7 | 36.538 | <0.001 |
|  | NHHR | 0.647 (0.568-0.714) | 59.4 | 70.0 | 52.1 | 75.8 | 2.412 | <0.001 |
|  | NFS | 0.589 (0.521-0.650) | 46.7 | 70.5 | 46.5 | 70.6 | -2.042 | 0.013 |
|  | FIB-4 | 0.469 (0.403-0.540) | 43.6 | 53.3 | 34.0 | 63.2 | 0.742 | 0.926 |
|  | BARD | 0.608 (0.539-0.670) | 65.1 | 63.8 | 49.7 | 76.8 | 1.585 | <0.001 |
| Hypertension | TyG | 0.714 (0.624-0.791) | 68.1 | 66.0 | 70.6 | 63.3 | 8.306 | <0.001 |
|  | TyG-BMI | 0.804 (0.715-0.872) | 74.9 | 75.4 | 78.5 | 71.5 | 232.727 | <0.001 |
|  | TyG-WC | 0.827 (0.759-0.887) | 73.6 | 76.1 | 78.7 | 70.6 | 815.660 | <0.001 |
|  | TyG-WHtR | 0.782 (0.703-0.854) | 70.5 | 73.1 | 75.9 | 67.4 | 5.065 | <0.001 |
|  | TyG-WWI | 0.714 (0.627-0.791) | 62.5 | 70.9 | 72.0 | 61.2 | 94.197 | <0.001 |
|  | VAI | 0.746 (0.661-0.816) | 81.5 | 61.2 | 71.6 | 73.3 | 0.983 | <0.001 |
|  | HOMA-IR | 0.722 (0.640-0.803) | 65.5 | 74.1 | 75.2 | 64.1 | 1.843 | 0.002 |
|  | METS-IR | 0.796 (0.719-0.866) | 76.6 | 73.2 | 77.4 | 72.3 | 38.772 | <0.001 |
|  | FSI | 0.792 (0.708-0.865) | 70.6 | 72.5 | 75.5 | 67.3 | -1.126 | <0.001 |
|  | FLI | 0.829 (0.763-0.887) | 78.6 | 75.2 | 79.2 | 74.5 | 44.412 | <0.001 |
|  | USFLI | 0.808 (0.739-0.866) | 82.4 | 68.6 | 75.9 | 76.5 | 10.732 | <0.001 |
|  | ZJU | 0.783 (0.696-0.850) | 82.0 | 68.7 | 75.9 | 76.1 | 37.449 | <0.001 |
|  | LAP | 0.812 (0.744-0.876) | 80.4 | 72.7 | 78.0 | 75.6 | 35.724 | <0.001 |
|  | HSI | 0.780 (0.697-0.853) | 83.5 | 66.8 | 75.1 | 77.2 | 35.200 | <0.001 |
|  | NHHR | 0.729 (0.647-0.797) | 68.8 | 72.3 | 74.9 | 65.9 | 2.380 | <0.001 |
|  | NFS | 0.620 (0.518-0.719) | 73.2 | 52.3 | 64.8 | 61.9 | -1.660 | 0.036 |
|  | FIB-4 | 0.512 (0.411-0.609) | 70.1 | 40.7 | 58.6 | 53.1 | 1.330 | 0.751 |
|  | BARD | 0.624 (0.521-0.721) | 65.0 | 68.2 | 71.1 | 61.9 | 1.756 | 0.015 |

MASLD, metabolic dysfunction-associated steatotic liver disease; TyG: triglyceride-glucose index; BMI: body mass index; WC: waist circumference; WHtR: waist-to-height ratio; WWI: weight-adjusted waist index; VAI: visceral adiposity index; HOMA-IR: homeostatic model assessment of insulin resistance; METS-IR: metabolic score for insulin resistance; FSI: Framingham steatosis index; FLI: fatty liver index; ZJU: Zhejiang University index; LAP: lipid accumulation product; HSI: hepatic steatosis index; NHHR: non-high-density lipoprotein cholesterol (HDL-C) to HDL-C ratio; NFS: nonalcoholic fatty liver disease fibrosis score; FIB-4: fibrosis-4 index; BARD: BMI-aspartate aminotransferase/alanine aminotransferase ratio and diabetes score

**Table. S5**. Diagnostic efficacy of 18 indices for MASLD in patients with prediabetes across different demographic characteristics

| Group | Variables | AUC (95%CI) | Sensitivity (%) | Specificity (%) | PPV | NPV | Cut-off Values | p-Value |
| --- | --- | --- | --- | --- | --- | --- | --- | --- |
| Female | TyG | 0.654 (0.580-0.720) | 61.9 | 63.8 | 72.3 | 52.3 | 8.487 | <0.001 |
|  | TyG-BMI | 0.719 (0.656-0.781) | 69.3 | 63.0 | 74.1 | 57.4 | 235.311 | <0.001 |
|  | TyG-WC | 0.737 (0.670-0.796) | 72.5 | 67.7 | 77.4 | 61.7 | 813.164 | <0.001 |
|  | TyG-WHtR | 0.729 (0.664-0.788) | 71.4 | 69.1 | 77.9 | 61.3 | 5.048 | <0.001 |
|  | TyG-WWI | 0.694 (0.628-0.753) | 67.0 | 71.2 | 78.0 | 58.6 | 95.423 | <0.001 |
|  | VAI | 0.659 (0.576-0.731) | 63.0 | 62.8 | 72.1 | 52.7 | 1.325 | <0.001 |
|  | HOMA-IR | 0.687 (0.620-0.754) | 53.8 | 74.2 | 76.1 | 51.3 | 3.101 | <0.001 |
|  | METS-IR | 0.708 (0.640-0.772) | 63.6 | 66.9 | 74.6 | 54.7 | 40.977 | <0.001 |
|  | FSI | 0.732 (0.661-0.793) | 57.9 | 75.4 | 78.2 | 54.0 | -1.245 | <0.001 |
|  | FLI | 0.742 (0.679-0.804) | 76.2 | 63.5 | 76.1 | 63.6 | 35.142 | <0.001 |
|  | USFLI | 0.753 (0.680-0.814) | 71.2 | 68.7 | 77.6 | 61.0 | 18.147 | <0.001 |
|  | ZJU | 0.716 (0.644-0.782) | 69.7 | 61.0 | 73.2 | 56.9 | 39.719 | <0.001 |
|  | LAP | 0.725 (0.657-0.784) | 65.6 | 72.2 | 78.2 | 57.9 | 42.177 | 0.001 |
|  | HSI | 0.707 (0.643-0.770) | 63.6 | 66.8 | 74.5 | 54.6 | 38.144 | <0.001 |
|  | NHHR | 0.622 (0.541-0.690) | 49.9 | 66.0 | 69.1 | 46.4 | 2.643 | 0.002 |
|  | NFS | 0.610 (0.543-0.674) | 66.6 | 49.8 | 66.9 | 49.4 | -1.727 | 0.002 |
|  | FIB-4 | 0.503 (0.435-0.574) | 57.9 | 46.8 | 62.4 | 42.2 | 0.946 | 0.576 |
|  | BARD | 0.537 (0.462-0.607) | 58.4 | 55.9 | 66.8 | 46.8 | 1.750 | 0.373 |
| Male | TyG | 0.666 (0.582-0.745) | 62.4 | 64.5 | 78.4 | 45.4 | 8.457 | <0.001 |
|  | TyG-BMI | 0.786 (0.718-0.845) | 72.8 | 71.7 | 84.2 | 56.1 | 233.433 | <0.001 |
|  | TyG-WC | 0.784 (0.718-0.848) | 79.6 | 64.1 | 82.1 | 60.4 | 809.009 | <0.001 |
|  | TyG-WHtR | 0.783 (0.714-0.845) | 72.7 | 65.5 | 81.3 | 53.8 | 4.695 | <0.001 |
|  | TyG-WWI | 0.732 (0.660-0.798) | 69.8 | 60.4 | 78.5 | 49.3 | 89.827 | <0.001 |
|  | VAI | 0.667 (0.586-0.743) | 71.8 | 56.7 | 77.4 | 49.3 | 0.939 | 0.001 |
|  | HOMA-IR | 0.649 (0.564-0.726) | 56.6 | 71.7 | 80.5 | 44.5 | 2.741 | <0.001 |
|  | METS-IR | 0.769 (0.700-0.827) | 71.6 | 75.4 | 85.7 | 56.2 | 41.657 | <0.001 |
|  | FSI | 0.792 (0.721-0.853) | 72.8 | 72.9 | 84.7 | 56.5 | -1.515 | <0.001 |
|  | FLI | 0.791 (0.729-0.846) | 75.2 | 69.1 | 83.4 | 57.5 | 44.810 | <0.001 |
|  | USFLI | 0.735 (0.669-0.800) | 58.2 | 77.3 | 84.1 | 47.2 | 25.631 | <0.001 |
|  | ZJU | 0.782 (0.711-0.837) | 71.0 | 73.0 | 84.4 | 55.0 | 38.222 | <0.001 |
|  | LAP | 0.748 (0.669-0.816) | 71.6 | 63.8 | 80.3 | 52.1 | 31.764 | <0.001 |
|  | HSI | 0.756 (0.696-0.816) | 65.9 | 77.7 | 85.9 | 52.5 | 37.276 | <0.001 |
|  | NHHR | 0.629 (0.538-0.708) | 65.0 | 58.5 | 76.4 | 44.8 | 2.622 | 0.013 |
|  | NFS | 0.667 (0.590-0.733) | 68.4 | 59.7 | 77.8 | 47.8 | -1.934 | 0.005 |
|  | FIB-4 | 0.576 (0.491-0.656) | 56.3 | 57.3 | 73.1 | 38.8 | 0.866 | 0.383 |
|  | BARD | 0.662 (0.586-0.730) | 72.0 | 67.7 | 82.1 | 53.9 | 1.508 | 0.002 |
| Mexican American | TyG | 0.693 (0.582-0.789) | 66.0 | 66.1 | 77.0 | 53.1 | 8.615 | 0.001 |
|  | TyG-BMI | 0.793 (0.701-0.870) | 64.4 | 86.5 | 89.1 | 58.6 | 259.744 | 0.003 |
|  | TyG-WC | 0.788 (0.690-0.870) | 61.5 | 85.4 | 87.8 | 56.3 | 876.572 | <0.001 |
|  | TyG-WHtR | 0.792 (0.708-0.873) | 74.8 | 74.4 | 83.4 | 63.2 | 4.985 | <0.001 |
|  | TyG-WWI | 0.740 (0.640-0.836) | 67.8 | 75.9 | 82.9 | 57.9 | 94.532 | <0.001 |
|  | VAI | 0.684 (0.577-0.783) | 66.8 | 69.6 | 79.0 | 55.0 | 1.448 | 0.005 |
|  | HOMA-IR | 0.809 (0.717-0.886) | 82.8 | 67.2 | 81.3 | 69.5 | 2.601 | 0.015 |
|  | METS-IR | 0.788 (0.703-0.866) | 72.8 | 76.7 | 84.3 | 62.1 | 43.811 | 0.003 |
|  | FSI | 0.793 (0.702-0.867) | 74.4 | 73.4 | 82.8 | 62.6 | -1.497 | 0.001 |
|  | FLI | 0.790 (0.700-0.867) | 64.0 | 82.8 | 86.5 | 57.2 | 62.325 | <0.001 |
|  | USFLI | 0.820 (0.736-0.898) | 75.7 | 77.9 | 85.5 | 65.1 | 35.070 | <0.001 |
|  | ZJU | 0.804 (0.714-0.879) | 69.8 | 83.0 | 87.6 | 61.5 | 40.955 | 0.006 |
|  | LAP | 0.768 (0.676-0.852) | 64.6 | 81.7 | 85.9 | 57.3 | 46.111 | <0.001 |
|  | HSI | 0.779 (0.686-0.863) | 64.5 | 87.4 | 89.8 | 58.9 | 40.270 | 0.004 |
|  | NHHR | 0.635 (0.525-0.738) | 70.3 | 56.1 | 73.3 | 52.3 | 2.587 | 0.108 |
|  | NFS | 0.660 (0.565-0.764) | 60.1 | 64.2 | 74.3 | 48.4 | -2.044 | 0.017 |
|  | FIB-4 | 0.564 (0.457-0.676) | 56.0 | 66.5 | 74.2 | 46.8 | 0.734 | 0.064 |
|  | BARD | 0.565 (0.445-0.689) | 69.5 | 52.3 | 71.4 | 49.9 | 1.552 | 0.353 |
| Non-Hispanic Black | TyG | 0.629 (0.535-0.721) | 66.2 | 55.5 | 62.7 | 59.2 | 8.132 | 0.003 |
|  | TyG-BMI | 0.783 (0.714-0.849) | 68.6 | 75.1 | 75.7 | 67.9 | 258.429 | <0.001 |
|  | TyG-WC | 0.781 (0.710-0.851) | 76.0 | 68.0 | 72.9 | 71.5 | 825.226 | <0.001 |
|  | TyG-WHtR | 0.767 (0.686-0.838) | 71.4 | 72.4 | 74.5 | 69.1 | 4.974 | <0.001 |
|  | TyG-WWI | 0.697 (0.610-0.782) | 74.0 | 61.2 | 68.4 | 67.6 | 87.994 | 0.018 |
|  | VAI | 0.632 (0.543-0.716) | 58.7 | 63.9 | 64.8 | 57.7 | 1.057 | 0.033 |
|  | HOMA-IR | 0.789 (0.710-0.853) | 72.4 | 70.6 | 73.6 | 69.3 | 2.873 | 0.028 |
|  | METS-IR | 0.776 (0.704-0.840) | 64.7 | 79.5 | 78.1 | 66.5 | 46.932 | <0.001 |
|  | FSI | 0.796 (0.729-0.861) | 70.8 | 81.1 | 80.9 | 71.1 | -1.047 | <0.001 |
|  | FLI | 0.801 (0.728-0.866) | 77.4 | 72.9 | 76.4 | 74.0 | 50.731 | <0.001 |
|  | USFLI | 0.844 (0.781-0.897) | 78.5 | 75.4 | 78.3 | 75.6 | 14.089 | <0.001 |
|  | ZJU | 0.781 (0.710-0.849) | 65.8 | 81.4 | 80.0 | 67.8 | 43.185 | <0.001 |
|  | LAP | 0.749 (0.665-0.822) | 69.0 | 70.7 | 72.7 | 66.9 | 34.303 | 0.002 |
|  | HSI | 0.785 (0.723-0.850) | 72.2 | 73.6 | 75.6 | 70.1 | 39.400 | <0.001 |
|  | NHHR | 0.545 (0.457-0.634) | 64.5 | 53.2 | 60.9 | 57.0 | 2.176 | 0.562 |
|  | NFS | 0.639 (0.556-0.720) | 59.2 | 68.2 | 67.8 | 59.6 | -1.019 | 0.009 |
|  | FIB-4 | 0.547 (0.459-0.628) | 48.2 | 61.5 | 58.7 | 51.2 | 0.814 | 0.041 |
|  | BARD | 0.570 (0.486-0.651) | 70.3 | 48.1 | 60.5 | 58.9 | 1.824 | 0.028 |
| Non-Hispanic White | TyG | 0.648 (0.563-0.729) | 63.9 | 62.8 | 78.0 | 45.7 | 8.457 | <0.001 |
|  | TyG-BMI | 0.751 (0.685-0.816) | 71.1 | 68.1 | 82.2 | 53.2 | 234.573 | <0.001 |
|  | TyG-WC | 0.766 (0.694-0.825) | 78.7 | 65.7 | 82.6 | 59.8 | 811.731 | <0.001 |
|  | TyG-WHtR | 0.738 (0.668-0.802) | 62.1 | 74.6 | 83.5 | 48.7 | 5.045 | <0.001 |
|  | TyG-WWI | 0.687 (0.608-0.756) | 58.2 | 69.1 | 79.6 | 44.4 | 94.157 | <0.001 |
|  | VAI | 0.640 (0.564-0.716) | 67.5 | 57.6 | 76.7 | 46.2 | 1.047 | <0.001 |
|  | HOMA-IR | 0.638 (0.554-0.718) | 55.2 | 68.0 | 78.1 | 42.3 | 2.691 | 0.001 |
|  | METS-IR | 0.739 (0.671-0.801) | 66.4 | 75.7 | 85.0 | 52.1 | 41.657 | <0.001 |
|  | FSI | 0.763 (0.699-0.823) | 72.0 | 66.7 | 81.7 | 53.6 | -1.609 | <0.001 |
|  | FLI | 0.775 (0.707-0.836) | 71.8 | 71.0 | 83.7 | 54.9 | 45.216 | <0.001 |
|  | USFLI | 0.729 (0.657-0.794) | 68.0 | 65.1 | 80.1 | 49.6 | 18.152 | <0.001 |
|  | ZJU | 0.738 (0.667-0.802) | 71.7 | 65.2 | 81.0 | 52.7 | 38.239 | <0.001 |
|  | LAP | 0.721 (0.645-0.788) | 63.1 | 69.3 | 81.0 | 47.6 | 38.251 | <0.001 |
|  | HSI | 0.721 (0.658-0.778) | 71.8 | 64.2 | 80.6 | 52.4 | 36.276 | <0.001 |
|  | NHHR | 0.643 (0.554-0.724) | 56.8 | 66.3 | 77.7 | 42.6 | 2.622 | 0.002 |
|  | NFS | 0.652 (0.575-0.723) | 72.6 | 52.5 | 76.0 | 48.1 | -1.990 | 0.003 |
|  | FIB-4 | 0.548 (0.462-0.631) | 62.9 | 46.6 | 70.9 | 37.8 | 0.866 | 0.326 |
|  | BARD | 0.632 (0.556-0.704) | 68.0 | 64.4 | 79.8 | 49.3 | 1.568 | 0.013 |
| Other Hispanic | TyG | 0.784 (0.672-0.865) | 81.7 | 70.2 | 81.3 | 70.7 | 8.370 | <0.001 |
|  | TyG-BMI | 0.766 (0.645-0.862) | 68.0 | 73.8 | 80.5 | 59.2 | 241.204 | 0.011 |
|  | TyG-WC | 0.761 (0.643-0.860) | 59.1 | 83.7 | 85.2 | 56.3 | 855.798 | <0.001 |
|  | TyG-WHtR | 0.767 (0.652-0.869) | 68.9 | 78.0 | 83.3 | 61.2 | 5.140 | 0.002 |
|  | TyG-WWI | 0.719 (0.603-0.821) | 65.5 | 79.2 | 83.3 | 59.1 | 96.791 | <0.001 |
|  | VAI | 0.780 (0.671-0.869) | 87.2 | 63.4 | 79.1 | 75.7 | 0.975 | 0.010 |
|  | HOMA-IR | 0.784 (0.682-0.875) | 70.5 | 80.8 | 85.4 | 63.3 | 2.820 | <0.001 |
|  | METS-IR | 0.774 (0.664-0.872) | 72.5 | 76.6 | 83.1 | 63.6 | 41.744 | 0.012 |
|  | FSI | 0.760 (0.648-0.857) | 65.1 | 70.0 | 77.5 | 55.8 | -1.471 | 0.005 |
|  | FLI | 0.748 (0.630-0.845) | 72.5 | 64.4 | 76.4 | 59.6 | 41.971 | 0.003 |
|  | USFLI | 0.777 (0.658-0.882) | 69.8 | 74.4 | 81.2 | 60.8 | 23.933 | 0.008 |
|  | ZJU | 0.757 (0.635-0.859) | 83.7 | 65.2 | 79.3 | 71.6 | 37.714 | 0.020 |
|  | LAP | 0.770 (0.657-0.873) | 71.5 | 74.1 | 81.4 | 62.1 | 37.080 | 0.005 |
|  | HSI | 0.751 (0.626-0.854) | 77.8 | 66.4 | 78.6 | 65.3 | 36.559 | 0.020 |
|  | NHHR | 0.711 (0.603-0.808) | 56.6 | 83.1 | 84.2 | 54.7 | 3.061 | 0.001 |
|  | NFS | 0.572 (0.444-0.690) | 58.7 | 59.1 | 69.5 | 47.4 | -1.716 | 0.277 |
|  | FIB-4 | 0.582 (0.428-0.725) | 75.7 | 49.6 | 70.4 | 56.2 | 1.100 | 0.098 |
|  | BARD | 0.558 (0.427-0.693) | 75.1 | 55.3 | 72.7 | 58.3 | 1.417 | 0.398 |
| Other Race | TyG | 0.644 (0.541-0.748) | 52.8 | 69.9 | 69.3 | 53.5 | 8.690 | 0.002 |
|  | TyG-BMI | 0.713 (0.599-0.827) | 66.9 | 70.0 | 74.2 | 62.2 | 230.184 | 0.021 |
|  | TyG-WC | 0.744 (0.639-0.844) | 65.8 | 73.3 | 76.0 | 62.5 | 806.782 | 0.012 |
|  | TyG-WHtR | 0.745 (0.630-0.846) | 66.4 | 73.6 | 76.4 | 63.0 | 4.874 | 0.002 |
|  | TyG-WWI | 0.718 (0.627-0.803) | 57.4 | 71.3 | 72.0 | 56.6 | 95.162 | <0.001 |
|  | VAI | 0.652 (0.547-0.759) | 58.7 | 64.6 | 68.0 | 54.9 | 1.496 | 0.008 |
|  | HOMA-IR | 0.603 (0.479-0.737) | 71.4 | 49.9 | 64.7 | 57.5 | 2.221 | 0.117 |
|  | METS-IR | 0.699 (0.589-0.814) | 68.0 | 66.6 | 72.4 | 61.8 | 39.121 | 0.036 |
|  | FSI | 0.742 (0.646-0.841) | 63.5 | 71.0 | 73.8 | 60.2 | -1.597 | 0.003 |
|  | FLI | 0.737 (0.628-0.835) | 72.6 | 65.5 | 73.0 | 65.0 | 34.445 | 0.004 |
|  | USFLI | 0.689 (0.585-0.808) | 59.2 | 74.6 | 75.0 | 58.7 | 21.409 | 0.014 |
|  | ZJU | 0.708 (0.591-0.821) | 68.5 | 67.9 | 73.3 | 62.6 | 37.863 | 0.031 |
|  | LAP | 0.725 (0.623-0.823) | 68.7 | 63.7 | 70.9 | 61.3 | 30.360 | 0.012 |
|  | HSI | 0.698 (0.581-0.802) | 66.6 | 64.4 | 70.6 | 60.0 | 35.200 | 0.056 |
|  | NHHR | 0.617 (0.520-0.719) | 54.5 | 68.2 | 68.8 | 53.9 | 3.058 | 0.068 |
|  | NFS | 0.601 (0.507-0.689) | 66.9 | 53.9 | 65.1 | 55.9 | -1.975 | 0.066 |
|  | FIB-4 | 0.573 (0.467-0.671) | 54.2 | 64.2 | 66.0 | 52.1 | 0.946 | 0.395 |
|  | BARD | 0.545 (0.444-0.646) | 51.9 | 65.3 | 65.8 | 51.4 | 1.551 | 0.569 |
| Age ≤50 | TyG | 0.653 (0.572-0.726) | 62.9 | 63.6 | 69.3 | 56.8 | 8.459 | <0.001 |
|  | TyG-BMI | 0.779 (0.710-0.843) | 76.6 | 67.4 | 75.4 | 68.8 | 236.167 | <0.001 |
|  | TyG-WC | 0.786 (0.720-0.850) | 77.9 | 70.3 | 77.4 | 70.9 | 811.731 | <0.001 |
|  | TyG-WHtR | 0.772 (0.702-0.836) | 76.3 | 64.4 | 73.7 | 67.5 | 4.681 | <0.001 |
|  | TyG-WWI | 0.725 (0.652-0.793) | 72.5 | 58.6 | 69.6 | 62.0 | 88.148 | <0.001 |
|  | VAI | 0.654 (0.572-0.739) | 64.0 | 58.9 | 67.0 | 55.6 | 1.197 | <0.001 |
|  | HOMA-IR | 0.669 (0.584-0.756) | 61.3 | 65.3 | 69.8 | 56.4 | 2.731 | <0.001 |
|  | METS-IR | 0.771 (0.693-0.838) | 80.8 | 65.4 | 75.3 | 72.4 | 40.759 | <0.001 |
|  | FSI | 0.784 (0.713-0.850) | 67.2 | 74.0 | 77.1 | 63.3 | -1.510 | <0.001 |
|  | FLI | 0.788 (0.718-0.850) | 74.5 | 71.5 | 77.3 | 68.3 | 44.827 | <0.001 |
|  | USFLI | 0.741 (0.666-0.810) | 62.1 | 75.3 | 76.6 | 60.4 | 21.696 | <0.001 |
|  | ZJU | 0.779 (0.715-0.841) | 75.5 | 70.0 | 76.6 | 68.7 | 38.992 | <0.001 |
|  | LAP | 0.743 (0.671-0.813) | 67.9 | 68.8 | 73.9 | 62.2 | 36.693 | <0.001 |
|  | HSI | 0.770 (0.699-0.839) | 68.6 | 79.1 | 81.1 | 65.9 | 39.235 | <0.001 |
|  | NHHR | 0.626 (0.548-0.711) | 66.9 | 56.3 | 66.6 | 56.6 | 2.622 | 0.022 |
|  | NFS | 0.677 (0.603-0.743) | 61.0 | 67.8 | 71.2 | 57.1 | -2.219 | <0.001 |
|  | FIB-4 | 0.532 (0.459-0.607) | 59.5 | 52.5 | 62.1 | 49.9 | 0.596 | 0.547 |
|  | BARD | 0.614 (0.544-0.686) | 73.3 | 58.9 | 69.9 | 62.8 | 1.514 | 0.067 |
| Age >50 | TyG | 0.674 (0.610-0.737) | 62.2 | 64.5 | 81.3 | 40.7 | 8.486 | <0.001 |
|  | TyG-BMI | 0.731 (0.663-0.793) | 74.0 | 63.5 | 83.4 | 49.6 | 226.633 | <0.001 |
|  | TyG-WC | 0.737 (0.673-0.798) | 69.4 | 66.1 | 83.6 | 46.5 | 828.007 | <0.001 |
|  | TyG-WHtR | 0.706 (0.643-0.763) | 63.3 | 69.7 | 83.8 | 43.3 | 5.062 | <0.001 |
|  | TyG-WWI | 0.647 (0.579-0.706) | 63.2 | 65.2 | 81.8 | 41.6 | 94.178 | <0.001 |
|  | VAI | 0.661 (0.597-0.726) | 66.2 | 62.5 | 81.4 | 42.7 | 1.078 | <0.001 |
|  | HOMA-IR | 0.679 (0.605-0.750) | 54.8 | 73.5 | 83.7 | 39.6 | 2.820 | <0.001 |
|  | METS-IR | 0.735 (0.674-0.793) | 60.9 | 78.3 | 87.4 | 44.6 | 41.530 | <0.001 |
|  | FSI | 0.734 (0.668-0.797) | 69.4 | 65.8 | 83.4 | 46.4 | -1.422 | <0.001 |
|  | FLI | 0.750 (0.690-0.809) | 75.1 | 63.8 | 83.8 | 50.8 | 38.407 | <0.001 |
|  | USFLI | 0.729 (0.663-0.790) | 64.0 | 71.2 | 84.7 | 44.4 | 21.564 | <0.001 |
|  | ZJU | 0.705 (0.635-0.766) | 69.9 | 64.4 | 83.0 | 46.3 | 38.153 | <0.001 |
|  | LAP | 0.719 (0.652-0.777) | 61.6 | 71.9 | 84.5 | 43.0 | 38.512 | <0.001 |
|  | HSI | 0.697 (0.635-0.758) | 67.2 | 64.6 | 82.5 | 44.2 | 36.291 | <0.001 |
|  | NHHR | 0.665 (0.598-0.724) | 59.9 | 63.6 | 80.3 | 39.0 | 2.428 | <0.001 |
|  | NFS | 0.551 (0.485-0.612) | 54.4 | 61.8 | 77.9 | 35.3 | -0.774 | 0.198 |
|  | FIB-4 | 0.592 (0.525-0.655) | 58.2 | 63.0 | 79.6 | 37.7 | 1.264 | 0.048 |
|  | BARD | 0.582 (0.506-0.652) | 63.7 | 60.4 | 80.0 | 40.1 | 1.583 | 0.098 |
| Never-smoker | TyG | 0.653 (0.590-0.713) | 64.5 | 59.9 | 72.8 | 50.3 | 8.459 | <0.001 |
|  | TyG-BMI | 0.753 (0.702-0.805) | 75.6 | 65.8 | 78.6 | 61.9 | 234.573 | <0.001 |
|  | TyG-WC | 0.765 (0.710-0.813) | 74.3 | 68.2 | 79.5 | 61.5 | 823.350 | <0.001 |
|  | TyG-WHtR | 0.748 (0.692-0.798) | 65.2 | 75.2 | 81.4 | 56.5 | 5.062 | <0.001 |
|  | TyG-WWI | 0.695 (0.641-0.754) | 57.2 | 72.9 | 77.8 | 50.6 | 95.577 | <0.001 |
|  | VAI | 0.640 (0.571-0.704) | 63.6 | 57.6 | 71.4 | 48.7 | 1.161 | <0.001 |
|  | HOMA-IR | 0.701 (0.641-0.761) | 60.5 | 69.4 | 76.7 | 51.3 | 2.813 | <0.001 |
|  | METS-IR | 0.736 (0.678-0.791) | 70.5 | 66.8 | 77.9 | 57.7 | 41.668 | <0.001 |
|  | FSI | 0.766 (0.717-0.814) | 69.9 | 66.8 | 77.8 | 57.2 | -1.456 | <0.001 |
|  | FLI | 0.768 (0.717-0.818) | 75.0 | 66.4 | 78.8 | 61.5 | 42.636 | <0.001 |
|  | USFLI | 0.767 (0.716-0.814) | 74.9 | 66.0 | 78.6 | 61.3 | 18.147 | <0.001 |
|  | ZJU | 0.750 (0.695-0.796) | 69.7 | 67.7 | 78.2 | 57.3 | 39.719 | <0.001 |
|  | LAP | 0.728 (0.667-0.785) | 66.2 | 66.2 | 76.5 | 54.1 | 36.693 | <0.001 |
|  | HSI | 0.739 (0.687-0.789) | 71.6 | 65.8 | 77.7 | 58.2 | 37.500 | <0.001 |
|  | NHHR | 0.605 (0.535-0.674) | 60.5 | 57.1 | 70.1 | 46.5 | 2.621 | 0.011 |
|  | NFS | 0.639 (0.576-0.699) | 65.5 | 56.2 | 71.3 | 49.4 | -1.802 | <0.001 |
|  | FIB-4 | 0.531 (0.461-0.597) | 53.4 | 53.7 | 65.8 | 41.0 | 0.941 | 0.306 |
|  | BARD | 0.576 (0.510-0.630) | 68.7 | 53.6 | 71.1 | 50.7 | 1.551 | 0.106 |
| Former smoker | TyG | 0.696 (0.578-0.800) | 72.5 | 64.0 | 82.1 | 50.6 | 8.228 | 0.010 |
|  | TyG-BMI | 0.760 (0.650-0.850) | 75.4 | 66.2 | 83.5 | 54.2 | 223.446 | 0.023 |
|  | TyG-WC | 0.761 (0.650-0.851) | 76.7 | 67.6 | 84.3 | 56.1 | 807.246 | 0.012 |
|  | TyG-WHtR | 0.743 (0.632-0.833) | 61.6 | 71.0 | 82.9 | 44.9 | 4.898 | 0.013 |
|  | TyG-WWI | 0.714 (0.587-0.817) | 56.3 | 74.5 | 83.4 | 42.9 | 94.166 | 0.016 |
|  | VAI | 0.711 (0.594-0.807) | 78.1 | 61.3 | 82.1 | 55.2 | 0.808 | 0.015 |
|  | HOMA-IR | 0.553 (0.427-0.688) | 59.1 | 56.4 | 75.5 | 37.7 | 2.279 | 0.132 |
|  | METS-IR | 0.746 (0.654-0.832) | 64.4 | 84.4 | 90.3 | 51.0 | 41.389 | 0.005 |
|  | FSI | 0.760 (0.637-0.855) | 79.3 | 65.7 | 84.0 | 58.2 | -1.826 | 0.019 |
|  | FLI | 0.766 (0.655-0.856) | 80.2 | 64.2 | 83.6 | 58.7 | 32.353 | 0.009 |
|  | USFLI | 0.664 (0.567-0.774) | 54.3 | 77.8 | 84.7 | 42.8 | 24.834 | 0.005 |
|  | ZJU | 0.724 (0.604-0.817) | 60.0 | 77.9 | 86.1 | 46.2 | 39.059 | 0.033 |
|  | LAP | 0.744 (0.629-0.836) | 60.8 | 77.9 | 86.2 | 46.6 | 38.251 | 0.011 |
|  | HSI | 0.700 (0.593-0.799) | 60.7 | 75.4 | 84.9 | 45.7 | 36.977 | 0.025 |
|  | NHHR | 0.708 (0.590-0.795) | 78.2 | 63.0 | 82.8 | 56.0 | 1.931 | 0.022 |
|  | NFS | 0.653 (0.527-0.760) | 78.9 | 54.9 | 79.9 | 53.4 | -2.219 | 0.123 |
|  | FIB-4 | 0.594 (0.448-0.714) | 56.6 | 61.9 | 77.2 | 38.6 | 0.960 | 0.578 |
|  | BARD | 0.665 (0.560-0.748) | 69.1 | 69.9 | 83.9 | 49.9 | 1.509 | 0.038 |
| Current smoker | TyG | 0.656 (0.532-0.784) | 71.8 | 60.2 | 72.7 | 59.1 | 8.398 | 0.075 |
|  | TyG-BMI | 0.755 (0.645-0.872) | 70.4 | 70.6 | 77.9 | 61.7 | 231.444 | <0.001 |
|  | TyG-WC | 0.789 (0.687-0.881) | 74.5 | 69.6 | 78.4 | 64.9 | 825.226 | <0.001 |
|  | TyG-WHtR | 0.786 (0.679-0.888) | 81.4 | 73.1 | 81.7 | 72.7 | 4.861 | <0.001 |
|  | TyG-WWI | 0.733 (0.612-0.843) | 58.2 | 81.3 | 82.1 | 56.8 | 95.552 | <0.001 |
|  | VAI | 0.655 (0.527-0.790) | 58.3 | 74.5 | 77.2 | 54.7 | 1.539 | 0.044 |
|  | HOMA-IR | 0.770 (0.657-0.871) | 66.1 | 79.5 | 82.7 | 61.3 | 2.565 | 0.025 |
|  | METS-IR | 0.740 (0.617-0.858) | 59.0 | 88.1 | 88.0 | 59.2 | 44.000 | 0.002 |
|  | FSI | 0.786 (0.682-0.888) | 69.0 | 78.9 | 82.8 | 63.2 | -1.597 | <0.001 |
|  | FLI | 0.798 (0.686-0.889) | 72.6 | 77.4 | 82.6 | 65.6 | 44.827 | <0.001 |
|  | USFLI | 0.849 (0.768-0.924) | 80.0 | 78.4 | 84.6 | 72.6 | 16.459 | 0.002 |
|  | ZJU | 0.742 (0.628-0.854) | 65.8 | 80.0 | 82.9 | 61.3 | 39.246 | <0.001 |
|  | LAP | 0.767 (0.656-0.872) | 63.1 | 80.4 | 82.7 | 59.6 | 48.443 | 0.019 |
|  | HSI | 0.758 (0.646-0.870) | 64.0 | 80.2 | 82.7 | 60.1 | 38.634 | <0.001 |
|  | NHHR | 0.631 (0.503-0.761) | 66.0 | 54.8 | 68.3 | 52.2 | 2.552 | 0.101 |
|  | NFS | 0.599 (0.482-0.726) | 49.9 | 74.6 | 74.4 | 50.2 | -1.063 | 0.121 |
|  | FIB-4 | 0.549 (0.418-0.674) | 65.9 | 50.5 | 66.3 | 50.0 | 0.983 | 0.761 |
|  | BARD | 0.590 (0.450-0.727) | 68.8 | 59.4 | 71.4 | 56.2 | 1.514 | 0.430 |
| Non-Obese | TyG | 0.640 (0.571-0.709) | 60.2 | 62.8 | 63.2 | 59.7 | 8.413 | <0.001 |
|  | TyG-BMI | 0.684 (0.612-0.751) | 67.6 | 63.6 | 66.4 | 64.9 | 215.118 | <0.001 |
|  | TyG-WC | 0.709 (0.643-0.771) | 69.0 | 67.9 | 69.6 | 67.4 | 778.165 | <0.001 |
|  | TyG-WHtR | 0.677 (0.611-0.740) | 69.6 | 59.1 | 64.4 | 64.7 | 4.535 | <0.001 |
|  | TyG-WWI | 0.646 (0.578-0.712) | 48.0 | 70.1 | 63.0 | 55.9 | 92.973 | <0.001 |
|  | VAI | 0.620 (0.547-0.693) | 67.1 | 54.9 | 61.3 | 61.1 | 0.939 | <0.001 |
|  | HOMA-IR | 0.547 (0.473-0.615) | 59.8 | 49.7 | 55.8 | 53.8 | 1.889 | 0.384 |
|  | METS-IR | 0.645 (0.572-0.713) | 57.0 | 69.8 | 66.7 | 60.4 | 37.707 | 0.001 |
|  | FSI | 0.709 (0.641-0.769) | 72.4 | 62.9 | 67.5 | 68.2 | -2.183 | <0.001 |
|  | FLI | 0.709 (0.645-0.770) | 66.1 | 69.1 | 69.5 | 65.8 | 29.734 | <0.001 |
|  | USFLI | 0.646 (0.574-0.718) | 66.2 | 57.0 | 62.1 | 61.4 | 12.902 | <0.001 |
|  | ZJU | 0.659 (0.591-0.725) | 55.6 | 70.0 | 66.3 | 59.7 | 37.273 | 0.002 |
|  | LAP | 0.686 (0.620-0.752) | 69.7 | 59.7 | 64.7 | 65.0 | 25.536 | <0.001 |
|  | HSI | 0.624 (0.557-0.692) | 55.1 | 66.1 | 63.3 | 58.1 | 34.644 | 0.007 |
|  | NHHR | 0.585 (0.512-0.651) | 73.1 | 44.4 | 58.3 | 60.8 | 1.994 | 0.055 |
|  | NFS | 0.602 (0.528-0.668) | 64.0 | 56.7 | 61.1 | 59.7 | -1.990 | 0.009 |
|  | FIB-4 | 0.588 (0.519-0.658) | 59.0 | 55.5 | 58.4 | 56.0 | 0.944 | 0.070 |
|  | BARD | 0.517 (0.451-0.583) | 42.8 | 63.4 | 55.4 | 51.1 | 1.429 | 0.416 |
| Obese | TyG | 0.626 (0.542-0.708) | 56.0 | 68.1 | 89.5 | 24.2 | 8.643 | 0.009 |
|  | TyG-BMI | 0.650 (0.557-0.733) | 66.2 | 63.6 | 89.8 | 27.9 | 286.909 | 0.030 |
|  | TyG-WC | 0.693 (0.619-0.771) | 71.8 | 66.1 | 91.1 | 32.6 | 934.072 | 0.002 |
|  | TyG-WHtR | 0.662 (0.576-0.744) | 59.7 | 73.6 | 91.7 | 27.4 | 5.757 | 0.012 |
|  | TyG-WWI | 0.649 (0.573-0.722) | 59.8 | 69.0 | 90.3 | 26.1 | 97.905 | 0.009 |
|  | VAI | 0.601 (0.501-0.690) | 52.0 | 61.9 | 86.9 | 21.0 | 1.694 | 0.080 |
|  | HOMA-IR | 0.683 (0.600-0.771) | 57.9 | 68.2 | 89.8 | 25.0 | 4.016 | 0.006 |
|  | METS-IR | 0.665 (0.573-0.760) | 61.6 | 65.5 | 89.6 | 26.0 | 50.452 | 0.013 |
|  | FSI | 0.679 (0.586-0.759) | 67.2 | 62.7 | 89.7 | 28.3 | -0.423 | 0.010 |
|  | FLI | 0.684 (0.596-0.765) | 67.2 | 67.5 | 90.9 | 29.8 | 79.771 | 0.001 |
|  | USFLI | 0.730 (0.654-0.800) | 57.9 | 80.3 | 93.5 | 28.3 | 41.076 | <0.001 |
|  | ZJU | 0.634 (0.540-0.728) | 60.7 | 64.3 | 89.2 | 25.2 | 45.173 | 0.059 |
|  | LAP | 0.645 (0.562-0.727) | 63.7 | 63.1 | 89.3 | 26.4 | 53.894 | 0.031 |
|  | HSI | 0.651 (0.551-0.738) | 65.3 | 61.2 | 89.1 | 26.7 | 43.431 | 0.056 |
|  | NHHR | 0.633 (0.534-0.723) | 55.2 | 71.3 | 90.3 | 24.7 | 3.053 | 0.011 |
|  | NFS | 0.563 (0.470-0.651) | 52.3 | 59.6 | 86.2 | 20.5 | -0.844 | 0.098 |
|  | FIB-4 | 0.504 (0.405-0.610) | 60.0 | 46.3 | 84.4 | 19.3 | 0.776 | 0.995 |
|  | BARD | 0.678 (0.593-0.766) | 70.2 | 60.0 | 89.5 | 29.4 | 2.048 | 0.010 |
| Non-Hypertension | TyG | 0.648 (0.577-0.713) | 59.9 | 64.0 | 69.7 | 53.6 | 8.455 | <0.001 |
|  | TyG-BMI | 0.761 (0.696-0.818) | 73.1 | 69.1 | 76.6 | 65.0 | 231.444 | <0.001 |
|  | TyG-WC | 0.778 (0.715-0.837) | 73.7 | 73.0 | 79.1 | 66.8 | 811.731 | <0.001 |
|  | TyG-WHtR | 0.759 (0.696-0.821) | 75.2 | 64.6 | 74.6 | 65.3 | 4.677 | <0.001 |
|  | TyG-WWI | 0.704 (0.640-0.765) | 73.9 | 54.0 | 68.9 | 59.9 | 88.649 | <0.001 |
|  | VAI | 0.635 (0.561-0.713) | 68.6 | 54.5 | 67.5 | 55.6 | 0.983 | <0.001 |
|  | HOMA-IR | 0.637 (0.557-0.715) | 62.4 | 62.5 | 69.7 | 54.6 | 2.273 | 0.002 |
|  | METS-IR | 0.740 (0.675-0.800) | 69.7 | 72.5 | 77.8 | 63.3 | 40.814 | <0.001 |
|  | FSI | 0.772 (0.712-0.828) | 79.2 | 64.5 | 75.5 | 69.2 | -2.159 | <0.001 |
|  | FLI | 0.776 (0.716-0.835) | 75.9 | 68.5 | 76.9 | 67.3 | 39.367 | <0.001 |
|  | USFLI | 0.727 (0.664-0.789) | 57.7 | 76.4 | 77.2 | 56.6 | 21.696 | <0.001 |
|  | ZJU | 0.754 (0.698-0.810) | 73.0 | 68.3 | 76.1 | 64.7 | 38.222 | <0.001 |
|  | LAP | 0.731 (0.660-0.794) | 70.3 | 65.0 | 73.5 | 61.3 | 31.795 | <0.001 |
|  | HSI | 0.737 (0.675-0.798) | 72.4 | 66.2 | 74.8 | 63.4 | 36.291 | <0.001 |
|  | NHHR | 0.627 (0.554-0.699) | 58.9 | 60.8 | 67.5 | 51.7 | 2.621 | 0.006 |
|  | NFS | 0.654 (0.581-0.723) | 64.1 | 63.1 | 70.6 | 56.0 | -1.919 | 0.001 |
|  | FIB-4 | 0.540 (0.467-0.611) | 61.6 | 47.2 | 61.8 | 47.1 | 0.725 | 0.428 |
|  | BARD | 0.619 (0.551-0.684) | 67.2 | 63.4 | 71.7 | 58.3 | 1.515 | 0.017 |
| Hypertension | TyG | 0.665 (0.588-0.730) | 64.9 | 63.2 | 83.5 | 38.6 | 8.492 | <0.001 |
|  | TyG-BMI | 0.711 (0.641-0.774) | 56.4 | 77.1 | 87.6 | 38.1 | 258.307 | <0.001 |
|  | TyG-WC | 0.714 (0.644-0.778) | 63.5 | 65.2 | 84.0 | 38.4 | 863.052 | <0.001 |
|  | TyG-WHtR | 0.693 (0.622-0.762) | 62.6 | 72.7 | 86.8 | 40.4 | 5.225 | <0.001 |
|  | TyG-WWI | 0.653 (0.584-0.725) | 63.6 | 67.5 | 84.9 | 39.2 | 94.963 | <0.001 |
|  | VAI | 0.676 (0.604-0.743) | 66.7 | 63.0 | 83.8 | 39.7 | 1.153 | 0.019 |
|  | HOMA-IR | 0.673 (0.594-0.740) | 64.2 | 62.1 | 82.9 | 37.7 | 2.821 | <0.001 |
|  | METS-IR | 0.720 (0.654-0.782) | 59.5 | 79.8 | 89.4 | 40.7 | 43.966 | <0.001 |
|  | FSI | 0.721 (0.652-0.788) | 61.4 | 74.9 | 87.5 | 40.3 | -0.820 | <0.001 |
|  | FLI | 0.726 (0.659-0.795) | 67.3 | 64.7 | 84.5 | 40.8 | 54.209 | <0.001 |
|  | USFLI | 0.730 (0.653-0.798) | 69.1 | 67.0 | 85.7 | 43.0 | 22.233 | <0.001 |
|  | ZJU | 0.689 (0.624-0.762) | 66.1 | 66.3 | 84.9 | 40.5 | 39.831 | <0.001 |
|  | LAP | 0.712 (0.648-0.774) | 62.0 | 70.8 | 85.9 | 39.4 | 42.300 | 0.002 |
|  | HSI | 0.685 (0.611-0.751) | 56.4 | 72.9 | 85.7 | 36.8 | 39.200 | <0.001 |
|  | NHHR | 0.646 (0.566-0.715) | 64.8 | 61.6 | 82.9 | 37.8 | 2.428 | 0.008 |
|  | NFS | 0.559 (0.494-0.625) | 49.3 | 68.8 | 81.9 | 32.1 | -0.770 | 0.053 |
|  | FIB-4 | 0.535 (0.458-0.615) | 63.9 | 51.3 | 79.0 | 33.1 | 1.236 | 0.400 |
|  | BARD | 0.536 (0.455-0.619) | 66.2 | 48.7 | 78.8 | 33.4 | 1.603 | 0.397 |

MASLD, metabolic dysfunction-associated steatotic liver disease; TyG: triglyceride-glucose index; BMI: body mass index; WC: waist circumference; WHtR: waist-to-height ratio; WWI: weight-adjusted waist index; VAI: visceral adiposity index; HOMA-IR: homeostatic model assessment of insulin resistance; METS-IR: metabolic score for insulin resistance; FSI: Framingham steatosis index; FLI: fatty liver index; ZJU: Zhejiang University index; LAP: lipid accumulation product; HSI: hepatic steatosis index; NHHR: non-high-density lipoprotein cholesterol (HDL-C) to HDL-C ratio; NFS: nonalcoholic fatty liver disease fibrosis score; FIB-4: fibrosis-4 index; BARD: BMI-aspartate aminotransferase/alanine aminotransferase ratio and diabetes score

Note: We were unable to complete the subgroup analyses due to the insufficient population of other Hispanic subgroups.

**Table. S6**. Diagnostic efficacy of 18 indices for MASLD in patients with T2DM across different demographic characteristics

| Group | Variables | AUC (95%CI) | Sensitivity (%) | Specificity (%) | PPV | NPV | Cut-off Values | p-Value |
| --- | --- | --- | --- | --- | --- | --- | --- | --- |
| Female | TyG | 0.744 (0.665-0.816) | 77.5 | 68.8 | 93.8 | 33.6 | 8.722 | <0.001 |
|  | TyG-BMI | 0.849 (0.773-0.905) | 77.6 | 83.1 | 96.5 | 38.1 | 266.265 | <0.001 |
|  | TyG-WC | 0.850 (0.773-0.910) | 78.6 | 81.3 | 96.2 | 38.6 | 908.369 | <0.001 |
|  | TyG-WHtR | 0.842 (0.768-0.902) | 75.7 | 83.1 | 96.4 | 36.1 | 5.852 | <0.001 |
|  | TyG-WWI | 0.771 (0.673-0.843) | 64.3 | 78.4 | 94.7 | 26.7 | 106.487 | <0.001 |
|  | VAI | 0.797 (0.721-0.855) | 81.0 | 72.6 | 94.7 | 38.7 | 1.551 | <0.001 |
|  | HOMA-IR | 0.757 (0.668-0.833) | 69.3 | 78.9 | 95.2 | 29.8 | 4.272 | 0.024 |
|  | METS-IR | 0.864 (0.792-0.917) | 82.7 | 82.6 | 96.6 | 44.2 | 43.987 | <0.001 |
|  | FSI | 0.870 (0.799-0.925) | 76.9 | 85.8 | 97.0 | 38.0 | -0.688 | <0.001 |
|  | FLI | 0.851 (0.776-0.910) | 80.4 | 81.3 | 96.3 | 40.7 | 62.505 | <0.001 |
|  | USFLI | 0.828 (0.754-0.888) | 68.0 | 88.1 | 97.2 | 31.3 | 38.053 | <0.001 |
|  | ZJU | 0.857 (0.791-0.915) | 78.8 | 83.4 | 96.6 | 39.5 | 44.042 | <0.001 |
|  | LAP | 0.850 (0.772-0.908) | 73.7 | 86.2 | 97.0 | 35.2 | 60.006 | <0.001 |
|  | HSI | 0.854 (0.785-0.913) | 72.3 | 83.4 | 96.3 | 33.2 | 42.900 | <0.001 |
|  | NHHR | 0.710 (0.625-0.786) | 67.1 | 76.3 | 94.5 | 27.8 | 2.369 | 0.001 |
|  | NFS | 0.578 (0.445-0.716) | 59.2 | 52.0 | 88.2 | 17.4 | -0.065 | 0.194 |
|  | FIB-4 | 0.594 (0.473-0.726) | 80.0 | 49.5 | 90.5 | 29.1 | 1.334 | 0.013 |
|  | BARD | 0.574 (0.431-0.720) | 64.3 | 56.9 | 90.0 | 20.9 | 2.528 | 0.228 |
| Male | TyG | 0.737 (0.645-0.820) | 73.5 | 66.8 | 92.3 | 31.7 | 8.800 | <0.001 |
|  | TyG-BMI | 0.787 (0.693-0.865) | 75.1 | 76.3 | 94.5 | 36.0 | 250.439 | <0.001 |
|  | TyG-WC | 0.811 (0.737-0.867) | 77.6 | 80.5 | 95.6 | 39.7 | 930.787 | <0.001 |
|  | TyG-WHtR | 0.793 (0.709-0.865) | 73.9 | 77.9 | 94.8 | 35.5 | 5.387 | <0.001 |
|  | TyG-WWI | 0.767 (0.673-0.848) | 67.1 | 73.1 | 93.1 | 29.0 | 101.845 | <0.001 |
|  | VAI | 0.748 (0.631-0.856) | 71.2 | 73.6 | 93.6 | 31.9 | 1.514 | 0.018 |
|  | HOMA-IR | 0.780 (0.690-0.864) | 70.3 | 77.1 | 94.3 | 32.3 | 4.255 | 0.062 |
|  | METS-IR | 0.782 (0.690-0.861) | 76.7 | 72.8 | 93.9 | 36.5 | 43.412 | <0.001 |
|  | FSI | 0.762 (0.667-0.842) | 65.7 | 79.8 | 94.7 | 30.0 | -0.706 | <0.001 |
|  | FLI | 0.769 (0.670-0.846) | 67.6 | 78.3 | 94.4 | 30.7 | 69.582 | <0.001 |
|  | USFLI | 0.779 (0.677-0.875) | 61.3 | 81.0 | 94.6 | 27.8 | 46.106 | <0.001 |
|  | ZJU | 0.791 (0.678-0.868) | 84.9 | 69.9 | 93.9 | 46.0 | 39.303 | <0.001 |
|  | LAP | 0.798 (0.704-0.879) | 72.6 | 74.5 | 93.9 | 33.3 | 52.877 | <0.001 |
|  | HSI | 0.733 (0.614-0.829) | 63.9 | 79.3 | 94.4 | 28.8 | 39.067 | 0.005 |
|  | NHHR | 0.671 (0.533-0.810) | 74.1 | 57.5 | 90.4 | 29.0 | 2.236 | 0.086 |
|  | NFS | 0.557 (0.418-0.679) | 67.6 | 49.3 | 87.9 | 21.8 | -0.241 | 0.336 |
|  | FIB-4 | 0.483 (0.324-0.669) | 66.3 | 37.5 | 85.2 | 17.0 | 1.496 | 0.585 |
|  | BARD | 0.563 (0.467-0.651) | 57.9 | 70.0 | 91.3 | 23.4 | 2.391 | 0.215 |
| Mexican American | TyG | 0.730 (0.585-0.843) | 66.8 | 72.1 | 95.0 | 21.3 | 8.929 | 0.009 |
|  | TyG-BMI | 0.879 (0.742-0.969) | 87.2 | 80.7 | 97.3 | 44.1 | 250.189 | 0.012 |
|  | TyG-WC | 0.876 (0.772-0.962) | 71.2 | 100.0 | 100.0 | 30.2 | 931.795 | 0.011 |
|  | TyG-WHtR | 0.867 (0.773-0.946) | 77.5 | 94.9 | 99.2 | 34.5 | 5.703 | 0.001 |
|  | TyG-WWI | 0.767 (0.636-0.880) | 71.7 | 94.9 | 99.1 | 29.5 | 104.277 | 0.021 |
|  | VAI | 0.827 (0.705-0.908) | 76.2 | 85.2 | 97.6 | 30.9 | 1.446 | 0.004 |
|  | HOMA-IR | 0.640 (0.393-0.890) | 81.6 | 56.1 | 93.7 | 27.5 | 3.392 | 0.358 |
|  | METS-IR | 0.852 (0.683-0.969) | 84.4 | 80.7 | 97.2 | 39.2 | 43.871 | 0.039 |
|  | FSI | 0.863 (0.684-0.987) | 81.9 | 80.7 | 97.1 | 35.7 | -0.941 | 0.040 |
|  | FLI | 0.818 (0.626-0.983) | 70.7 | 88.5 | 98.0 | 27.4 | 73.215 | 0.009 |
|  | USFLI | 0.673 (0.411-0.945) | 75.8 | 69.2 | 95.2 | 26.3 | 44.096 | 0.180 |
|  | ZJU | 0.856 (0.731-0.969) | 77.7 | 80.7 | 97.0 | 31.1 | 44.398 | 0.026 |
|  | LAP | 0.876 (0.769-0.949) | 77.1 | 85.2 | 97.7 | 31.7 | 38.170 | 0.001 |
|  | HSI | 0.830 (0.602-0.979) | 84.8 | 80.7 | 97.2 | 39.9 | 38.700 | 0.037 |
|  | NHHR | 0.724 (0.575-0.835) | 64.3 | 91.6 | 98.4 | 24.2 | 2.565 | 0.057 |
|  | NFS | 0.508 (0.305-0.710) | 59.0 | 55.4 | 91.4 | 14.4 | -0.434 | 0.901 |
|  | FIB-4 | 0.624 (0.439-0.812) | 52.8 | 75.1 | 94.5 | 16.6 | 1.026 | 0.128 |
|  | BARD | 0.783 (0.629-0.897) | 76.4 | 88.5 | 98.2 | 31.9 | 2.250 | 0.015 |
| Non-Hispanic Black | TyG | 0.550 (0.443-0.661) | 57.0 | 56.2 | 77.5 | 33.1 | 8.648 | 0.393 |
|  | TyG-BMI | 0.725 (0.611-0.827) | 76.4 | 59.5 | 83.3 | 48.8 | 251.422 | 0.006 |
|  | TyG-WC | 0.711 (0.585-0.808) | 71.5 | 59.8 | 82.5 | 44.2 | 899.765 | <0.001 |
|  | TyG-WHtR | 0.693 (0.577-0.806) | 70.3 | 58.0 | 81.6 | 42.4 | 5.306 | 0.002 |
|  | TyG-WWI | 0.582 (0.479-0.683) | 61.4 | 59.2 | 79.9 | 36.7 | 97.976 | 0.076 |
|  | VAI | 0.650 (0.552-0.743) | 57.2 | 69.5 | 83.2 | 38.0 | 1.341 | 0.009 |
|  | HOMA-IR | 0.733 (0.609-0.834) | 71.6 | 76.2 | 88.8 | 50.4 | 4.400 | 0.081 |
|  | METS-IR | 0.722 (0.610-0.833) | 74.7 | 61.2 | 83.6 | 47.8 | 44.509 | 0.006 |
|  | FSI | 0.738 (0.632-0.845) | 77.8 | 60.1 | 83.8 | 50.7 | -1.134 | 0.003 |
|  | FLI | 0.734 (0.632-0.839) | 70.8 | 64.6 | 84.1 | 45.5 | 67.041 | <0.001 |
|  | USFLI | 0.758 (0.655-0.845) | 76.1 | 69.6 | 86.9 | 52.4 | 24.193 | <0.001 |
|  | ZJU | 0.714 (0.595-0.825) | 74.5 | 63.4 | 84.3 | 48.5 | 42.644 | 0.005 |
|  | LAP | 0.714 (0.607-0.815) | 64.7 | 66.3 | 83.5 | 41.5 | 43.147 | 0.002 |
|  | HSI | 0.759 (0.654-0.860) | 87.3 | 60.2 | 85.3 | 64.1 | 37.338 | 0.003 |
|  | NHHR | 0.614 (0.503-0.735) | 60.1 | 64.8 | 81.9 | 38.1 | 2.409 | 0.054 |
|  | NFS | 0.506 (0.388-0.632) | 48.6 | 60.2 | 76.3 | 30.7 | -0.014 | 0.964 |
|  | FIB-4 | 0.644 (0.527-0.760) | 69.0 | 59.3 | 81.8 | 42.0 | 1.158 | 0.026 |
|  | BARD | 0.504 (0.395-0.605) | 34.1 | 77.7 | 80.2 | 30.8 | 2.148 | 0.744 |
| Non-Hispanic White | TyG | 0.818 (0.691-0.919) | 83.0 | 75.7 | 95.9 | 39.1 | 8.805 | 0.002 |
|  | TyG-BMI | 0.905 (0.830-0.961) | 76.0 | 94.7 | 99.0 | 36.3 | 267.489 | <0.001 |
|  | TyG-WC | 0.926 (0.872-0.969) | 87.0 | 92.0 | 98.7 | 50.4 | 930.787 | <0.001 |
|  | TyG-WHtR | 0.913 (0.846-0.964) | 87.9 | 80.6 | 96.9 | 49.1 | 5.404 | <0.001 |
|  | TyG-WWI | 0.865 (0.762-0.936) | 70.2 | 91.4 | 98.3 | 30.7 | 105.807 | <0.001 |
|  | VAI | 0.830 (0.704-0.923) | 85.0 | 75.8 | 96.1 | 42.2 | 1.549 | 0.011 |
|  | HOMA-IR | 0.829 (0.721-0.920) | 71.4 | 88.9 | 97.8 | 31.0 | 4.255 | 0.043 |
|  | METS-IR | 0.905 (0.834-0.958) | 83.3 | 88.9 | 98.1 | 43.4 | 43.995 | <0.001 |
|  | FSI | 0.891 (0.798-0.959) | 76.8 | 94.7 | 99.0 | 37.0 | -0.697 | <0.001 |
|  | FLI | 0.903 (0.829-0.959) | 85.6 | 84.0 | 97.4 | 45.6 | 61.778 | <0.001 |
|  | USFLI | 0.862 (0.754-0.952) | 75.1 | 75.3 | 95.5 | 30.3 | 38.442 | 0.002 |
|  | ZJU | 0.906 (0.802-0.969) | 87.3 | 78.4 | 96.6 | 47.1 | 40.901 | <0.001 |
|  | LAP | 0.913 (0.843-0.968) | 87.5 | 79.2 | 96.7 | 47.7 | 51.282 | <0.001 |
|  | HSI | 0.840 (0.710-0.933) | 76.2 | 78.4 | 96.1 | 32.2 | 39.033 | 0.005 |
|  | NHHR | 0.705 (0.521-0.870) | 75.4 | 66.4 | 94.0 | 28.0 | 2.236 | 0.100 |
|  | NFS | 0.613 (0.436-0.793) | 71.7 | 49.8 | 90.8 | 20.2 | -0.131 | 0.161 |
|  | FIB-4 | 0.488 (0.292-0.714) | 63.9 | 45.5 | 89.1 | 15.4 | 1.354 | 0.679 |
|  | BARD | 0.628 (0.451-0.767) | 61.7 | 76.4 | 94.8 | 22.3 | 2.509 | 0.129 |
| Other Hispanic | TyG | 0.586 (0.360-0.764) | 72.9 | 47.7 | 88.1 | 24.9 | 8.792 | 0.105 |
|  | TyG-BMI | 0.709 (0.508-0.884) | 52.5 | 86.3 | 95.3 | 25.4 | 303.139 | 0.022 |
|  | TyG-WC | 0.684 (0.484-0.872) | 56.0 | 73.3 | 91.8 | 23.9 | 971.668 | 0.071 |
|  | TyG-WHtR | 0.696 (0.508-0.868) | 59.9 | 73.3 | 92.3 | 25.6 | 5.953 | 0.046 |
|  | TyG-WWI | 0.649 (0.442-0.812) | 49.1 | 84.3 | 94.3 | 23.7 | 107.297 | 0.106 |
|  | VAI | 0.611 (0.414-0.791) | 70.9 | 53.4 | 89.0 | 25.6 | 1.485 | 0.224 |
|  | HOMA-IR | 0.770 (0.626-0.885) | 69.1 | 84.1 | 95.9 | 33.8 | 4.998 | 0.252 |
|  | METS-IR | 0.772 (0.613-0.923) | 58.7 | 86.3 | 95.8 | 28.2 | 48.447 | 0.023 |
|  | FSI | 0.749 (0.583-0.889) | 62.2 | 76.1 | 93.3 | 27.5 | -0.482 | 0.008 |
|  | FLI | 0.713 (0.532-0.883) | 57.7 | 76.1 | 92.8 | 25.3 | 84.276 | 0.035 |
|  | USFLI | 0.725 (0.517-0.878) | 71.5 | 84.1 | 96.0 | 35.7 | 38.071 | 0.010 |
|  | ZJU | 0.738 (0.569-0.889) | 59.1 | 76.1 | 92.9 | 25.9 | 45.724 | 0.007 |
|  | LAP | 0.628 (0.411-0.840) | 52.1 | 73.3 | 91.2 | 22.3 | 61.197 | 0.274 |
|  | HSI | 0.745 (0.592-0.884) | 61.7 | 73.3 | 92.5 | 26.4 | 43.500 | <0.001 |
|  | NHHR | 0.698 (0.513-0.853) | 67.8 | 83.7 | 95.7 | 32.8 | 2.565 | 0.050 |
|  | NFS | 0.526 (0.273-0.760) | 57.9 | 55.4 | 87.4 | 19.8 | -0.206 | 0.650 |
|  | FIB-4 | 0.604 (0.348-0.843) | 73.3 | 55.4 | 89.7 | 28.1 | 1.219 | 0.090 |
|  | BARD | 0.596 (0.372-0.812) | 71.6 | 50.1 | 88.4 | 24.9 | 2.808 | 0.400 |
| Other Race | TyG | 0.641 (0.479-0.785) | 50.8 | 72.9 | 91.0 | 21.5 | 8.994 | 0.148 |
|  | TyG-BMI | 0.675 (0.510-0.834) | 69.3 | 65.8 | 91.6 | 28.4 | 240.359 | 0.156 |
|  | TyG-WC | 0.682 (0.500-0.849) | 73.1 | 65.4 | 92.0 | 31.0 | 857.701 | 0.201 |
|  | TyG-WHtR | 0.660 (0.511-0.814) | 61.8 | 74.2 | 92.8 | 26.4 | 5.387 | 0.169 |
|  | TyG-WWI | 0.597 (0.450-0.756) | 59.7 | 63.3 | 89.8 | 22.5 | 101.845 | 0.219 |
|  | VAI | 0.666 (0.511-0.836) | 79.6 | 62.3 | 92.0 | 36.0 | 1.167 | 0.244 |
|  | HOMA-IR | 0.627 (0.466-0.787) | 65.6 | 58.2 | 89.5 | 23.8 | 3.171 | 0.301 |
|  | METS-IR | 0.693 (0.535-0.843) | 62.9 | 80.0 | 94.5 | 28.5 | 42.060 | 0.152 |
|  | FSI | 0.656 (0.512-0.812) | 63.8 | 65.7 | 91.0 | 25.1 | -1.214 | 0.065 |
|  | FLI | 0.663 (0.505-0.819) | 75.5 | 68.2 | 92.8 | 33.9 | 42.275 | 0.097 |
|  | USFLI | 0.626 (0.460-0.779) | 75.8 | 55.8 | 90.3 | 29.8 | 24.513 | 0.226 |
|  | ZJU | 0.697 (0.551-0.852) | 63.7 | 71.4 | 92.4 | 26.6 | 41.228 | 0.069 |
|  | LAP | 0.679 (0.502-0.843) | 72.3 | 61.7 | 91.1 | 29.1 | 37.438 | 0.301 |
|  | HSI | 0.641 (0.498-0.786) | 62.6 | 68.9 | 91.6 | 25.4 | 38.315 | 0.080 |
|  | NHHR | 0.649 (0.461-0.816) | 62.8 | 67.1 | 91.2 | 25.0 | 2.340 | 0.493 |
|  | NFS | 0.565 (0.428-0.701) | 58.2 | 61.1 | 89.0 | 21.2 | -0.378 | 0.448 |
|  | FIB-4 | 0.495 (0.339-0.641) | 55.7 | 54.1 | 86.8 | 18.4 | 1.175 | 0.810 |
|  | BARD | 0.515 (0.375-0.677) | 69.6 | 39.1 | 86.1 | 19.2 | 2.737 | 0.971 |
| Age ≤50 | TyG | 0.779 (0.651-0.873) | 76.7 | 77.3 | 95.9 | 32.4 | 8.800 | 0.001 |
|  | TyG-BMI | 0.865 (0.739-0.954) | 93.2 | 78.5 | 96.8 | 62.4 | 243.437 | <0.001 |
|  | TyG-WC | 0.859 (0.750-0.947) | 80.2 | 86.0 | 97.5 | 38.5 | 932.824 | <0.001 |
|  | TyG-WHtR | 0.882 (0.788-0.950) | 78.9 | 86.0 | 97.5 | 37.0 | 5.661 | <0.001 |
|  | TyG-WWI | 0.843 (0.752-0.918) | 81.6 | 84.2 | 97.3 | 39.7 | 97.935 | <0.001 |
|  | VAI | 0.897 (0.816-0.961) | 79.6 | 95.6 | 99.2 | 40.3 | 1.526 | 0.002 |
|  | HOMA-IR | 0.703 (0.491-0.878) | 86.3 | 62.2 | 94.1 | 39.5 | 3.506 | 0.258 |
|  | METS-IR | 0.872 (0.746-0.960) | 94.2 | 78.5 | 96.8 | 66.2 | 41.688 | 0.001 |
|  | FSI | 0.903 (0.800-0.972) | 95.6 | 76.7 | 96.6 | 71.6 | -1.821 | <0.001 |
|  | FLI | 0.844 (0.736-0.933) | 80.8 | 83.6 | 97.2 | 38.6 | 79.042 | <0.001 |
|  | USFLI | 0.795 (0.627-0.913) | 73.9 | 76.1 | 95.6 | 29.6 | 43.575 | 0.004 |
|  | ZJU | 0.855 (0.718-0.956) | 88.3 | 78.5 | 96.6 | 49.2 | 42.081 | 0.002 |
|  | LAP | 0.893 (0.811-0.965) | 78.3 | 96.0 | 99.3 | 38.9 | 56.585 | <0.001 |
|  | HSI | 0.875 (0.762-0.951) | 90.3 | 78.5 | 96.7 | 53.7 | 39.095 | <0.001 |
|  | NHHR | 0.658 (0.430-0.923) | 94.2 | 50.3 | 92.9 | 55.3 | 1.974 | 0.237 |
|  | NFS | 0.669 (0.465-0.807) | 61.3 | 74.9 | 94.4 | 21.8 | -0.975 | 0.075 |
|  | FIB-4 | 0.483 (0.291-0.726) | 59.2 | 48.8 | 88.9 | 14.7 | 0.688 | 0.119 |
|  | BARD | 0.654 (0.482-0.779) | 63.8 | 75.8 | 94.8 | 23.2 | 2.509 | 0.044 |
| Age >50 | TyG | 0.728 (0.651-0.794) | 76.2 | 63.8 | 92.0 | 33.0 | 8.768 | <0.001 |
|  | TyG-BMI | 0.801 (0.727-0.863) | 65.9 | 82.4 | 95.3 | 30.8 | 267.146 | <0.001 |
|  | TyG-WC | 0.814 (0.752-0.864) | 73.4 | 80.0 | 95.2 | 35.6 | 930.787 | <0.001 |
|  | TyG-WHtR | 0.799 (0.728-0.857) | 79.4 | 70.7 | 93.6 | 38.7 | 5.387 | <0.001 |
|  | TyG-WWI | 0.763 (0.686-0.828) | 63.7 | 81.6 | 94.9 | 29.2 | 105.807 | <0.001 |
|  | VAI | 0.739 (0.648-0.818) | 75.1 | 68.0 | 92.7 | 33.4 | 1.551 | 0.001 |
|  | HOMA-IR | 0.779 (0.718-0.832) | 66.8 | 81.0 | 95.0 | 31.0 | 4.255 | <0.001 |
|  | METS-IR | 0.803 (0.730-0.860) | 73.3 | 78.2 | 94.8 | 35.0 | 43.995 | <0.001 |
|  | FSI | 0.791 (0.717-0.852) | 67.5 | 83.4 | 95.7 | 32.1 | -0.697 | <0.001 |
|  | FLI | 0.801 (0.732-0.859) | 74.8 | 79.7 | 95.3 | 36.8 | 62.152 | <0.001 |
|  | USFLI | 0.798 (0.725-0.868) | 69.0 | 74.7 | 93.7 | 30.7 | 38.053 | <0.001 |
|  | ZJU | 0.805 (0.725-0.865) | 76.4 | 69.2 | 93.1 | 35.0 | 41.791 | <0.001 |
|  | LAP | 0.807 (0.735-0.864) | 79.4 | 69.4 | 93.4 | 38.3 | 47.898 | <0.001 |
|  | HSI | 0.770 (0.683-0.847) | 77.3 | 68.3 | 93.0 | 35.6 | 38.215 | <0.001 |
|  | NHHR | 0.679 (0.590-0.760) | 63.3 | 70.3 | 92.0 | 26.0 | 2.364 | 0.021 |
|  | NFS | 0.560 (0.460-0.673) | 63.5 | 46.7 | 86.6 | 19.0 | 0.022 | 0.215 |
|  | FIB-4 | 0.521 (0.396-0.646) | 59.9 | 50.3 | 86.8 | 18.7 | 1.354 | 0.096 |
|  | BARD | 0.551 (0.450-0.652) | 58.2 | 64.4 | 89.9 | 22.1 | 2.528 | 0.238 |
| Never-smoker | TyG | 0.762 (0.690-0.827) | 68.2 | 76.4 | 94.6 | 28.6 | 8.800 | <0.001 |
|  | TyG-BMI | 0.777 (0.705-0.853) | 66.7 | 78.9 | 95.0 | 28.3 | 266.265 | <0.001 |
|  | TyG-WC | 0.793 (0.716-0.862) | 74.4 | 78.5 | 95.4 | 33.8 | 909.932 | <0.001 |
|  | TyG-WHtR | 0.774 (0.697-0.842) | 60.5 | 85.0 | 96.0 | 26.4 | 5.850 | <0.001 |
|  | TyG-WWI | 0.752 (0.674-0.817) | 77.7 | 66.4 | 93.3 | 33.2 | 99.384 | <0.001 |
|  | VAI | 0.780 (0.708-0.844) | 71.4 | 80.5 | 95.7 | 32.0 | 1.526 | <0.001 |
|  | HOMA-IR | 0.756 (0.670-0.836) | 68.2 | 74.4 | 94.1 | 28.0 | 4.244 | 0.059 |
|  | METS-IR | 0.789 (0.710-0.861) | 74.3 | 76.2 | 94.9 | 33.1 | 43.987 | <0.001 |
|  | FSI | 0.789 (0.690-0.865) | 79.1 | 71.4 | 94.3 | 36.2 | -1.140 | <0.001 |
|  | FLI | 0.759 (0.679-0.842) | 66.1 | 78.7 | 94.9 | 27.9 | 69.562 | <0.001 |
|  | USFLI | 0.781 (0.700-0.855) | 64.2 | 81.3 | 95.4 | 27.4 | 38.053 | <0.001 |
|  | ZJU | 0.784 (0.699-0.856) | 65.5 | 80.8 | 95.4 | 28.1 | 44.042 | <0.001 |
|  | LAP | 0.799 (0.738-0.856) | 66.8 | 86.9 | 96.8 | 30.4 | 55.884 | <0.001 |
|  | HSI | 0.725 (0.633-0.814) | 73.9 | 65.8 | 92.8 | 29.5 | 39.067 | <0.001 |
|  | NHHR | 0.685 (0.556-0.792) | 77.1 | 60.0 | 92.1 | 30.4 | 2.067 | 0.031 |
|  | NFS | 0.495 (0.377-0.617) | 70.5 | 37.6 | 87.2 | 17.5 | -0.399 | 0.666 |
|  | FIB-4 | 0.543 (0.427-0.667) | 71.5 | 49.2 | 89.4 | 22.3 | 1.336 | 0.064 |
|  | BARD | 0.502 (0.395-0.609) | 55.5 | 58.7 | 89.0 | 18.0 | 2.509 | 0.929 |
| Former smoker | TyG | 0.693 (0.571-0.810) | 50.2 | 92.8 | 97.7 | 23.8 | 9.237 | <0.001 |
|  | TyG-BMI | 0.851 (0.760-0.915) | 80.6 | 82.2 | 96.4 | 41.5 | 263.321 | <0.001 |
|  | TyG-WC | 0.853 (0.771-0.906) | 82.7 | 82.4 | 96.5 | 44.3 | 930.787 | <0.001 |
|  | TyG-WHtR | 0.864 (0.774-0.919) | 89.9 | 78.3 | 96.1 | 56.6 | 5.389 | <0.001 |
|  | TyG-WWI | 0.781 (0.663-0.867) | 71.0 | 79.5 | 95.4 | 31.5 | 105.534 | <0.001 |
|  | VAI | 0.780 (0.644-0.889) | 77.4 | 67.4 | 93.4 | 33.3 | 1.907 | 0.003 |
|  | HOMA-IR | 0.790 (0.673-0.873) | 75.5 | 82.5 | 96.3 | 36.1 | 4.261 | 0.112 |
|  | METS-IR | 0.849 (0.751-0.908) | 81.6 | 82.2 | 96.5 | 42.8 | 44.456 | <0.001 |
|  | FSI | 0.847 (0.758-0.903) | 81.0 | 83.6 | 96.7 | 42.5 | -0.697 | <0.001 |
|  | FLI | 0.866 (0.764-0.919) | 90.5 | 79.6 | 96.4 | 58.3 | 58.516 | <0.001 |
|  | USFLI | 0.821 (0.682-0.925) | 70.6 | 92.6 | 98.3 | 34.6 | 47.221 | <0.001 |
|  | ZJU | 0.845 (0.711-0.912) | 91.0 | 70.7 | 94.9 | 56.7 | 40.352 | <0.001 |
|  | LAP | 0.856 (0.784-0.913) | 77.8 | 95.2 | 99.0 | 41.8 | 65.630 | <0.001 |
|  | HSI | 0.834 (0.707-0.914) | 90.0 | 72.6 | 95.1 | 54.8 | 37.237 | 0.006 |
|  | NHHR | 0.683 (0.529-0.810) | 69.5 | 57.4 | 90.7 | 24.0 | 2.356 | 0.040 |
|  | NFS | 0.584 (0.435-0.724) | 67.4 | 52.3 | 89.4 | 21.2 | -0.028 | 0.463 |
|  | FIB-4 | 0.564 (0.355-0.770) | 70.6 | 44.1 | 88.3 | 20.1 | 1.483 | 0.168 |
|  | BARD | 0.622 (0.461-0.741) | 68.3 | 76.2 | 94.5 | 28.7 | 2.364 | 0.094 |
| Current smoker | TyG | 0.795 (0.599-0.937) | 84.8 | 77.5 | 94.0 | 54.9 | 8.650 | 0.025 |
|  | TyG-BMI | 0.899 (0.726-0.982) | 88.5 | 81.7 | 95.3 | 63.0 | 243.437 | 0.020 |
|  | TyG-WC | 0.898 (0.713-0.987) | 88.7 | 84.6 | 96.0 | 64.2 | 918.212 | 0.011 |
|  | TyG-WHtR | 0.889 (0.701-0.982) | 80.0 | 81.7 | 94.8 | 49.4 | 5.416 | 0.017 |
|  | TyG-WWI | 0.845 (0.614-0.966) | 89.7 | 69.8 | 92.6 | 61.8 | 97.752 | 0.039 |
|  | VAI | 0.775 (0.594-0.947) | 74.2 | 85.5 | 95.5 | 44.2 | 1.602 | 0.060 |
|  | HOMA-IR | 0.724 (0.555-0.877) | 72.6 | 76.8 | 92.9 | 40.1 | 2.937 | 0.264 |
|  | METS-IR | 0.902 (0.693-0.988) | 94.1 | 84.6 | 96.2 | 77.4 | 40.880 | 0.025 |
|  | FSI | 0.896 (0.712-0.980) | 78.7 | 85.0 | 95.6 | 48.7 | -1.117 | 0.022 |
|  | FLI | 0.892 (0.680-0.984) | 97.4 | 78.0 | 94.9 | 87.7 | 43.375 | 0.005 |
|  | USFLI | 0.854 (0.633-0.961) | 92.7 | 76.5 | 94.3 | 71.6 | 17.862 | 0.086 |
|  | ZJU | 0.878 (0.683-0.973) | 95.0 | 73.2 | 93.7 | 77.7 | 37.927 | 0.013 |
|  | LAP | 0.888 (0.626-0.994) | 88.9 | 85.5 | 96.2 | 64.7 | 47.320 | 0.145 |
|  | HSI | 0.877 (0.693-0.970) | 86.1 | 73.2 | 93.1 | 55.7 | 36.262 | 0.008 |
|  | NHHR | 0.722 (0.527-0.900) | 72.2 | 82.5 | 94.5 | 41.5 | 2.236 | 0.301 |
|  | NFS | 0.785 (0.523-0.901) | 80.6 | 73.2 | 92.6 | 47.4 | -0.705 | 0.060 |
|  | FIB-4 | 0.546 (0.387-0.762) | 48.3 | 83.9 | 92.6 | 27.9 | 0.791 | 0.706 |
|  | BARD | 0.685 (0.427-0.849) | 76.0 | 65.2 | 90.1 | 39.4 | 2.071 | 0.237 |
| Non-Obese | TyG | 0.733 (0.645-0.810) | 69.4 | 70.3 | 85.9 | 46.9 | 8.802 | 0.001 |
|  | TyG-BMI | 0.807 (0.734-0.877) | 80.0 | 66.1 | 86.0 | 56.0 | 230.043 | <0.001 |
|  | TyG-WC | 0.784 (0.688-0.880) | 70.1 | 74.6 | 87.8 | 49.0 | 856.659 | <0.001 |
|  | TyG-WHtR | 0.777 (0.695-0.857) | 68.3 | 71.3 | 86.1 | 46.4 | 5.144 | <0.001 |
|  | TyG-WWI | 0.744 (0.656-0.814) | 76.3 | 64.1 | 84.7 | 51.0 | 97.821 | <0.001 |
|  | VAI | 0.719 (0.624-0.816) | 58.4 | 82.4 | 89.6 | 43.3 | 1.907 | 0.009 |
|  | HOMA-IR | 0.757 (0.676-0.831) | 71.6 | 73.1 | 87.4 | 49.7 | 3.489 | 0.146 |
|  | METS-IR | 0.801 (0.700-0.888) | 75.5 | 69.4 | 86.5 | 52.2 | 39.194 | <0.001 |
|  | FSI | 0.776 (0.666-0.867) | 66.5 | 77.8 | 88.6 | 47.2 | -1.391 | <0.001 |
|  | FLI | 0.771 (0.665-0.873) | 73.4 | 72.0 | 87.2 | 51.1 | 42.275 | <0.001 |
|  | USFLI | 0.761 (0.638-0.858) | 79.3 | 68.3 | 86.7 | 56.0 | 24.513 | 0.001 |
|  | ZJU | 0.839 (0.771-0.890) | 74.4 | 81.4 | 91.2 | 55.1 | 39.009 | <0.001 |
|  | LAP | 0.755 (0.651-0.858) | 71.8 | 71.5 | 86.7 | 49.4 | 39.379 | 0.005 |
|  | HSI | 0.762 (0.656-0.848) | 68.2 | 73.5 | 87.0 | 47.1 | 36.227 | <0.001 |
|  | NHHR | 0.692 (0.584-0.800) | 74.8 | 65.4 | 84.9 | 50.0 | 2.248 | 0.030 |
|  | NFS | 0.540 (0.405-0.669) | 59.6 | 52.8 | 76.6 | 33.5 | -0.467 | 0.569 |
|  | FIB-4 | 0.464 (0.333-0.620) | 68.4 | 34.4 | 73.0 | 29.5 | 1.536 | 0.826 |
|  | BARD | 0.496 (0.393-0.599) | 35.3 | 83.0 | 84.4 | 33.1 | 2.364 | 0.644 |
| Obese | TyG | 0.704 (0.587-0.803) | 79.5 | 61.5 | 96.5 | 18.4 | 8.713 | 0.008 |
|  | TyG-BMI | 0.739 (0.618-0.839) | 69.6 | 72.9 | 97.2 | 15.3 | 313.169 | 0.015 |
|  | TyG-WC | 0.756 (0.641-0.841) | 72.3 | 70.5 | 97.0 | 16.0 | 1044.299 | 0.002 |
|  | TyG-WHtR | 0.747 (0.653-0.829) | 71.8 | 72.7 | 97.2 | 16.2 | 6.127 | <0.001 |
|  | TyG-WWI | 0.704 (0.583-0.799) | 62.0 | 77.3 | 97.3 | 13.3 | 106.803 | 0.006 |
|  | VAI | 0.775 (0.670-0.866) | 82.0 | 71.4 | 97.5 | 22.9 | 1.549 | 0.009 |
|  | HOMA-IR | 0.690 (0.579-0.794) | 75.6 | 58.8 | 96.1 | 15.3 | 4.255 | 0.092 |
|  | METS-IR | 0.741 (0.620-0.839) | 67.1 | 72.8 | 97.0 | 14.2 | 55.097 | 0.012 |
|  | FSI | 0.718 (0.582-0.831) | 84.8 | 52.4 | 96.0 | 20.5 | -0.310 | 0.014 |
|  | FLI | 0.731 (0.605-0.833) | 67.9 | 66.6 | 96.4 | 13.5 | 88.906 | <0.001 |
|  | USFLI | 0.740 (0.631-0.835) | 67.6 | 75.3 | 97.3 | 14.9 | 47.698 | <0.001 |
|  | ZJU | 0.694 (0.587-0.792) | 59.3 | 73.4 | 96.7 | 11.9 | 49.680 | 0.022 |
|  | LAP | 0.781 (0.663-0.863) | 79.7 | 74.3 | 97.6 | 21.5 | 62.378 | 0.004 |
|  | HSI | 0.670 (0.546-0.776) | 66.1 | 66.8 | 96.4 | 12.9 | 45.431 | 0.042 |
|  | NHHR | 0.684 (0.540-0.798) | 68.2 | 66.3 | 96.4 | 13.5 | 2.364 | 0.178 |
|  | NFS | 0.542 (0.422-0.658) | 61.9 | 52.4 | 94.5 | 9.4 | 0.606 | 0.357 |
|  | FIB-4 | 0.572 (0.445-0.699) | 64.4 | 54.4 | 95.0 | 10.3 | 1.236 | <0.001 |
|  | BARD | 0.672 (0.537-0.788) | 73.6 | 58.7 | 96.0 | 14.3 | 3.045 | 0.014 |
| Non-Hypertension | TyG | 0.805 (0.723-0.878) | 74.4 | 78.6 | 94.2 | 39.6 | 8.807 | <0.001 |
|  | TyG-BMI | 0.827 (0.727-0.928) | 75.6 | 77.6 | 94.1 | 40.4 | 248.567 | <0.001 |
|  | TyG-WC | 0.844 (0.766-0.922) | 73.6 | 91.2 | 97.5 | 42.4 | 916.746 | <0.001 |
|  | TyG-WHtR | 0.810 (0.708-0.907) | 73.2 | 74.5 | 93.1 | 37.2 | 5.295 | <0.001 |
|  | TyG-WWI | 0.758 (0.649-0.852) | 75.0 | 64.1 | 90.7 | 35.4 | 100.202 | <0.001 |
|  | VAI | 0.813 (0.730-0.896) | 71.5 | 84.3 | 95.5 | 38.7 | 1.526 | <0.001 |
|  | HOMA-IR | 0.758 (0.632-0.863) | 71.2 | 78.5 | 93.9 | 36.8 | 4.261 | 0.140 |
|  | METS-IR | 0.844 (0.761-0.925) | 67.6 | 90.8 | 97.2 | 37.4 | 43.871 | <0.001 |
|  | FSI | 0.878 (0.803-0.939) | 72.5 | 92.6 | 97.9 | 41.8 | -1.391 | <0.001 |
|  | FLI | 0.809 (0.707-0.914) | 67.7 | 76.1 | 93.0 | 33.5 | 62.173 | <0.001 |
|  | USFLI | 0.808 (0.725-0.887) | 70.2 | 89.8 | 97.0 | 39.2 | 38.071 | <0.001 |
|  | ZJU | 0.828 (0.718-0.924) | 80.8 | 69.4 | 92.5 | 43.5 | 40.590 | <0.001 |
|  | LAP | 0.833 (0.747-0.919) | 66.5 | 93.0 | 97.8 | 37.2 | 53.321 | <0.001 |
|  | HSI | 0.746 (0.630-0.872) | 63.2 | 77.6 | 93.0 | 31.0 | 39.226 | <0.001 |
|  | NHHR | 0.738 (0.540-0.894) | 74.1 | 75.1 | 93.3 | 38.2 | 2.387 | 0.095 |
|  | NFS | 0.528 (0.365-0.717) | 60.0 | 51.2 | 85.2 | 21.5 | -0.278 | 0.616 |
|  | FIB-4 | 0.517 (0.344-0.697) | 66.3 | 51.1 | 86.4 | 24.5 | 1.321 | 0.378 |
|  | BARD | 0.523 (0.355-0.680) | 45.5 | 59.4 | 84.0 | 18.9 | 2.143 | 0.794 |
| Hypertension | TyG | 0.702 (0.617-0.791) | 75.7 | 61.6 | 92.4 | 29.1 | 8.779 | 0.001 |
|  | TyG-BMI | 0.811 (0.723-0.876) | 74.2 | 76.6 | 95.2 | 32.4 | 267.489 | <0.001 |
|  | TyG-WC | 0.813 (0.742-0.871) | 77.1 | 78.0 | 95.6 | 35.5 | 932.824 | <0.001 |
|  | TyG-WHtR | 0.821 (0.744-0.882) | 71.4 | 83.6 | 96.4 | 32.1 | 5.834 | <0.001 |
|  | TyG-WWI | 0.774 (0.694-0.839) | 64.1 | 81.6 | 95.6 | 26.8 | 104.966 | <0.001 |
|  | VAI | 0.751 (0.649-0.832) | 77.5 | 68.0 | 93.7 | 32.8 | 1.549 | 0.002 |
|  | HOMA-IR | 0.772 (0.691-0.838) | 69.3 | 77.6 | 95.1 | 29.0 | 4.255 | 0.015 |
|  | METS-IR | 0.810 (0.735-0.878) | 81.3 | 72.5 | 94.8 | 38.6 | 43.995 | <0.001 |
|  | FSI | 0.804 (0.714-0.870) | 77.8 | 75.8 | 95.2 | 35.5 | -0.697 | <0.001 |
|  | FLI | 0.802 (0.716-0.870) | 82.8 | 72.2 | 94.9 | 40.4 | 59.495 | <0.001 |
|  | USFLI | 0.794 (0.713-0.877) | 79.8 | 63.4 | 93.1 | 33.7 | 32.381 | <0.001 |
|  | ZJU | 0.811 (0.718-0.877) | 81.5 | 69.6 | 94.3 | 37.7 | 41.936 | <0.001 |
|  | LAP | 0.818 (0.748-0.882) | 72.8 | 77.3 | 95.2 | 31.4 | 59.874 | <0.001 |
|  | HSI | 0.794 (0.681-0.871) | 85.2 | 66.8 | 94.1 | 42.1 | 38.215 | <0.001 |
|  | NHHR | 0.660 (0.554-0.748) | 66.2 | 63.0 | 91.7 | 23.1 | 2.356 | 0.035 |
|  | NFS | 0.580 (0.459-0.689) | 75.7 | 43.0 | 89.2 | 22.2 | -0.447 | 0.202 |
|  | FIB-4 | 0.531 (0.406-0.672) | 68.6 | 40.1 | 87.6 | 17.1 | 1.357 | 0.035 |
|  | BARD | 0.604 (0.500-0.697) | 64.2 | 67.8 | 92.5 | 23.4 | 2.509 | 0.053 |

MASLD, metabolic dysfunction-associated steatotic liver disease; T2DM, type 2 diabetes mellitus; TyG: triglyceride-glucose index; BMI: body mass index; WC: waist circumference; WHtR: waist-to-height ratio; WWI: weight-adjusted waist index; VAI: visceral adiposity index; HOMA-IR: homeostatic model assessment of insulin resistance; METS-IR: metabolic score for insulin resistance; FSI: Framingham steatosis index; FLI: fatty liver index; ZJU: Zhejiang University index; LAP: lipid accumulation product; HSI: hepatic steatosis index; NHHR: non-high-density lipoprotein cholesterol (HDL-C) to HDL-C ratio; NFS: nonalcoholic fatty liver disease fibrosis score; FIB-4: fibrosis-4 index; BARD: BMI-aspartate aminotransferase/alanine aminotransferase ratio and diabetes score

**Table. S7**. Diagnostic efficacy of 18 indices for significant fibrosis in patients across different demographic characteristics

| Group | Variables | AUC (95%CI) | Sensitivity (%) | Specificity (%) | PPV | NPV | Cut-off Values | p-Value |
| --- | --- | --- | --- | --- | --- | --- | --- | --- |
| Female | TyG | 0.697 (0.636-0.764) | 60.1 | 66.0 | 11.9 | 95.6 | 8.652 | <0.001 |
|  | TyG-BMI | 0.823 (0.766-0.874) | 81.9 | 70.3 | 17.4 | 98.1 | 273.489 | <0.001 |
|  | TyG-WC | 0.841 (0.789-0.884) | 76.3 | 80.3 | 22.8 | 97.8 | 960.545 | <0.001 |
|  | TyG-WHtR | 0.847 (0.803-0.890) | 82.8 | 74.1 | 19.6 | 98.3 | 5.751 | <0.001 |
|  | TyG-WWI | 0.790 (0.731-0.846) | 68.3 | 77.4 | 18.7 | 97.0 | 103.172 | <0.001 |
|  | VAI | 0.660 (0.601-0.718) | 64.7 | 62.7 | 11.7 | 95.9 | 1.619 | 0.002 |
|  | HOMA-IR | 0.774 (0.692-0.853) | 73.5 | 73.8 | 17.6 | 97.3 | 3.685 | 0.003 |
|  | METS-IR | 0.806 (0.752-0.856) | 75.5 | 72.7 | 17.4 | 97.5 | 47.422 | <0.001 |
|  | FSI | 0.831 (0.788-0.870) | 81.3 | 75.3 | 20.0 | 98.1 | -0.489 | <0.001 |
|  | FLI | 0.849 (0.801-0.887) | 79.3 | 79.3 | 22.6 | 98.0 | 82.610 | <0.001 |
|  | USFLI | 0.841 (0.770-0.898) | 87.0 | 72.3 | 19.3 | 98.7 | 29.829 | <0.001 |
|  | ZJU | 0.822 (0.766-0.869) | 79.4 | 72.2 | 17.9 | 97.9 | 44.631 | <0.001 |
|  | LAP | 0.770 (0.712-0.819) | 76.9 | 69.5 | 16.1 | 97.5 | 54.573 | <0.001 |
|  | HSI | 0.804 (0.746-0.859) | 79.9 | 73.8 | 18.9 | 98.0 | 43.079 | <0.001 |
|  | NHHR | 0.555 (0.480-0.634) | 52.4 | 60.9 | 9.3 | 94.4 | 2.480 | 0.204 |
|  | NFS | 0.810 (0.763-0.853) | 78.4 | 75.9 | 19.9 | 97.9 | -0.628 | <0.001 |
|  | FIB-4 | 0.640 (0.571-0.696) | 70.4 | 59.2 | 11.6 | 96.3 | 1.001 | <0.001 |
|  | BARD | 0.750 (0.696-0.804) | 78.8 | 63.0 | 14.0 | 97.5 | 1.941 | <0.001 |
| Male | TyG | 0.654 (0.585-0.715) | 65.5 | 61.4 | 15.4 | 94.3 | 8.673 | <0.001 |
|  | TyG-BMI | 0.744 (0.661-0.822) | 62.4 | 83.5 | 28.9 | 95.4 | 290.811 | <0.001 |
|  | TyG-WC | 0.758 (0.672-0.832) | 71.6 | 74.8 | 23.4 | 96.1 | 949.669 | <0.001 |
|  | TyG-WHtR | 0.754 (0.668-0.822) | 69.7 | 75.9 | 23.7 | 95.9 | 5.483 | <0.001 |
|  | TyG-WWI | 0.714 (0.637-0.782) | 67.2 | 69.8 | 19.3 | 95.2 | 98.050 | <0.001 |
|  | VAI | 0.637 (0.566-0.700) | 58.3 | 68.0 | 16.4 | 93.8 | 1.675 | 0.003 |
|  | HOMA-IR | 0.703 (0.623-0.774) | 64.3 | 76.9 | 23.0 | 95.2 | 4.277 | 0.002 |
|  | METS-IR | 0.740 (0.650-0.821) | 65.0 | 78.6 | 24.6 | 95.4 | 49.909 | <0.001 |
|  | FSI | 0.744 (0.653-0.818) | 67.7 | 73.0 | 21.2 | 95.5 | -0.555 | <0.001 |
|  | FLI | 0.764 (0.676-0.840) | 71.0 | 72.8 | 21.9 | 95.9 | 76.647 | <0.001 |
|  | USFLI | 0.748 (0.672-0.822) | 71.5 | 70.6 | 20.7 | 95.8 | 38.056 | <0.001 |
|  | ZJU | 0.743 (0.656-0.817) | 66.9 | 76.3 | 23.2 | 95.5 | 43.151 | <0.001 |
|  | LAP | 0.708 (0.634-0.773) | 65.9 | 73.7 | 21.2 | 95.3 | 58.233 | <0.001 |
|  | HSI | 0.728 (0.636-0.810) | 63.5 | 77.9 | 23.6 | 95.2 | 42.000 | <0.001 |
|  | NHHR | 0.542 (0.472-0.614) | 48.3 | 63.8 | 12.6 | 92.0 | 3.145 | 0.380 |
|  | NFS | 0.723 (0.647-0.788) | 66.0 | 66.5 | 17.4 | 94.8 | -0.836 | <0.001 |
|  | FIB-4 | 0.590 (0.525-0.655) | 60.3 | 58.7 | 13.6 | 93.2 | 1.023 | <0.001 |
|  | BARD | 0.687 (0.616-0.754) | 51.4 | 72.9 | 16.9 | 93.3 | 1.957 | <0.001 |
| Mexican American | TyG | 0.652 (0.517-0.775) | 64.4 | 56.9 | 12.9 | 94.2 | 8.689 | 0.062 |
|  | TyG-BMI | 0.796 (0.720-0.862) | 78.3 | 73.5 | 22.7 | 97.2 | 283.126 | <0.001 |
|  | TyG-WC | 0.804 (0.733-0.869) | 70.8 | 79.2 | 25.2 | 96.5 | 974.138 | <0.001 |
|  | TyG-WHtR | 0.804 (0.736-0.863) | 82.3 | 71.0 | 21.9 | 97.6 | 5.636 | <0.001 |
|  | TyG-WWI | 0.749 (0.663-0.826) | 83.9 | 61.8 | 17.9 | 97.5 | 98.355 | <0.001 |
|  | VAI | 0.584 (0.476-0.677) | 65.4 | 56.3 | 12.9 | 94.3 | 1.626 | 0.991 |
|  | HOMA-IR | 0.756 (0.632-0.855) | 73.8 | 66.8 | 18.0 | 96.3 | 4.372 | 0.029 |
|  | METS-IR | 0.768 (0.669-0.841) | 77.2 | 72.4 | 21.7 | 97.0 | 50.268 | <0.001 |
|  | FSI | 0.771 (0.677-0.849) | 79.4 | 68.8 | 20.1 | 97.1 | -0.632 | 0.005 |
|  | FLI | 0.817 (0.760-0.877) | 88.9 | 73.3 | 24.8 | 98.5 | 77.977 | <0.001 |
|  | USFLI | 0.829 (0.731-0.904) | 78.7 | 80.6 | 28.7 | 97.4 | 56.375 | <0.001 |
|  | ZJU | 0.797 (0.703-0.869) | 78.3 | 73.4 | 22.6 | 97.2 | 45.455 | <0.001 |
|  | LAP | 0.700 (0.619-0.772) | 72.6 | 67.1 | 17.9 | 96.1 | 55.845 | 0.119 |
|  | HSI | 0.741 (0.646-0.827) | 73.9 | 70.8 | 20.0 | 96.5 | 43.486 | <0.001 |
|  | NHHR | 0.570 (0.450-0.677) | 50.0 | 73.7 | 15.8 | 93.7 | 2.087 | 0.138 |
|  | NFS | 0.801 (0.725-0.894) | 83.7 | 63.9 | 18.6 | 97.5 | -1.426 | <0.001 |
|  | FIB-4 | 0.643 (0.493-0.808) | 51.2 | 77.9 | 18.7 | 94.2 | 1.138 | 0.001 |
|  | BARD | 0.770 (0.670-0.847) | 78.6 | 63.2 | 17.4 | 96.8 | 1.936 | <0.001 |
| Non-Hispanic Black | TyG | 0.687 (0.615-0.758) | 71.9 | 54.5 | 11.4 | 96.0 | 8.152 | <0.001 |
|  | TyG-BMI | 0.713 (0.629-0.794) | 62.4 | 75.8 | 17.3 | 96.1 | 298.069 | <0.001 |
|  | TyG-WC | 0.753 (0.672-0.829) | 74.4 | 73.2 | 18.4 | 97.2 | 933.177 | <0.001 |
|  | TyG-WHtR | 0.736 (0.647-0.822) | 70.1 | 75.7 | 19.0 | 96.9 | 5.681 | <0.001 |
|  | TyG-WWI | 0.715 (0.632-0.791) | 72.4 | 67.3 | 15.2 | 96.8 | 93.198 | <0.001 |
|  | VAI | 0.655 (0.583-0.724) | 69.0 | 56.8 | 11.5 | 95.8 | 1.039 | 0.033 |
|  | HOMA-IR | 0.701 (0.610-0.790) | 66.1 | 74.6 | 17.4 | 96.4 | 4.224 | 0.001 |
|  | METS-IR | 0.710 (0.620-0.788) | 70.8 | 65.4 | 14.2 | 96.5 | 47.685 | <0.001 |
|  | FSI | 0.736 (0.650-0.814) | 68.9 | 73.9 | 17.6 | 96.7 | -0.454 | <0.001 |
|  | FLI | 0.745 (0.660-0.823) | 74.6 | 67.7 | 15.8 | 97.0 | 75.264 | 0.001 |
|  | USFLI | 0.755 (0.676-0.834) | 72.1 | 72.4 | 17.5 | 97.0 | 22.880 | <0.001 |
|  | ZJU | 0.704 (0.618-0.794) | 64.4 | 74.0 | 16.7 | 96.2 | 46.641 | <0.001 |
|  | LAP | 0.712 (0.624-0.790) | 75.1 | 63.0 | 14.1 | 96.9 | 37.777 | 0.005 |
|  | HSI | 0.703 (0.601-0.785) | 67.6 | 69.3 | 15.1 | 96.3 | 43.079 | <0.001 |
|  | NHHR | 0.607 (0.522-0.686) | 73.5 | 47.5 | 10.2 | 95.7 | 2.121 | 0.008 |
|  | NFS | 0.676 (0.586-0.768) | 63.9 | 65.6 | 13.1 | 95.7 | -0.748 | <0.001 |
|  | FIB-4 | 0.569 (0.488-0.653) | 50.2 | 65.7 | 10.6 | 94.2 | 1.025 | 0.128 |
|  | BARD | 0.626 (0.531-0.716) | 49.3 | 74.6 | 13.6 | 94.8 | 2.333 | 0.003 |
| Non-Hispanic White | TyG | 0.683 (0.617-0.748) | 62.2 | 65.7 | 14.6 | 94.9 | 8.724 | <0.001 |
|  | TyG-BMI | 0.783 (0.705-0.854) | 73.8 | 73.7 | 20.9 | 96.8 | 273.489 | <0.001 |
|  | TyG-WC | 0.806 (0.730-0.869) | 77.3 | 75.4 | 22.9 | 97.2 | 955.102 | <0.001 |
|  | TyG-WHtR | 0.800 (0.722-0.869) | 75.0 | 74.7 | 21.8 | 96.9 | 5.642 | <0.001 |
|  | TyG-WWI | 0.755 (0.689-0.821) | 75.6 | 65.5 | 17.1 | 96.6 | 98.208 | <0.001 |
|  | VAI | 0.656 (0.593-0.718) | 63.9 | 65.6 | 14.9 | 95.1 | 1.675 | 0.002 |
|  | HOMA-IR | 0.737 (0.654-0.811) | 69.5 | 73.6 | 19.9 | 96.2 | 3.685 | 0.016 |
|  | METS-IR | 0.775 (0.699-0.845) | 65.4 | 81.3 | 24.8 | 96.1 | 51.538 | <0.001 |
|  | FSI | 0.790 (0.712-0.856) | 74.5 | 73.0 | 20.6 | 96.8 | -0.555 | <0.001 |
|  | FLI | 0.809 (0.737-0.873) | 75.4 | 77.4 | 23.9 | 97.1 | 83.036 | <0.001 |
|  | USFLI | 0.792 (0.721-0.860) | 77.7 | 71.6 | 20.5 | 97.1 | 34.570 | <0.001 |
|  | ZJU | 0.778 (0.695-0.850) | 72.1 | 75.0 | 21.3 | 96.6 | 43.618 | <0.001 |
|  | LAP | 0.746 (0.669-0.811) | 76.4 | 68.2 | 18.4 | 96.8 | 54.573 | <0.001 |
|  | HSI | 0.765 (0.678-0.838) | 70.8 | 76.0 | 21.8 | 96.5 | 42.500 | <0.001 |
|  | NHHR | 0.565 (0.487-0.640) | 61.2 | 53.1 | 10.9 | 93.6 | 2.500 | 0.062 |
|  | NFS | 0.762 (0.689-0.817) | 73.2 | 72.4 | 20.0 | 96.6 | -0.628 | <0.001 |
|  | FIB-4 | 0.605 (0.542-0.670) | 68.7 | 56.1 | 12.8 | 95.0 | 1.015 | <0.001 |
|  | BARD | 0.697 (0.625-0.768) | 61.2 | 68.7 | 15.6 | 95.0 | 1.926 | <0.001 |
| Other Hispanic | TyG | 0.721 (0.598-0.830) | 70.8 | 72.9 | 21.6 | 96.0 | 8.800 | 0.001 |
|  | TyG-BMI | 0.829 (0.716-0.917) | 81.4 | 83.0 | 33.5 | 97.7 | 297.623 | <0.001 |
|  | TyG-WC | 0.830 (0.705-0.923) | 75.1 | 89.5 | 43.0 | 97.2 | 1013.834 | <0.001 |
|  | TyG-WHtR | 0.836 (0.717-0.928) | 79.4 | 84.2 | 34.6 | 97.5 | 5.936 | <0.001 |
|  | TyG-WWI | 0.783 (0.655-0.891) | 83.8 | 67.0 | 21.1 | 97.5 | 98.064 | <0.001 |
|  | VAI | 0.667 (0.534-0.778) | 73.2 | 58.7 | 15.7 | 95.4 | 1.480 | 0.027 |
|  | HOMA-IR | 0.758 (0.599-0.883) | 68.3 | 91.7 | 46.5 | 96.5 | 6.626 | 0.105 |
|  | METS-IR | 0.828 (0.723-0.916) | 78.1 | 81.8 | 31.2 | 97.3 | 51.700 | <0.001 |
|  | FSI | 0.832 (0.729-0.913) | 81.4 | 79.4 | 29.4 | 97.6 | -0.312 | <0.001 |
|  | FLI | 0.848 (0.748-0.940) | 78.4 | 88.2 | 41.2 | 97.5 | 90.086 | 0.010 |
|  | USFLI | 0.807 (0.673-0.915) | 77.3 | 89.7 | 44.3 | 97.4 | 52.264 | <0.001 |
|  | ZJU | 0.813 (0.702-0.904) | 76.5 | 78.2 | 27.0 | 96.9 | 45.587 | <0.001 |
|  | LAP | 0.776 (0.656-0.879) | 77.7 | 72.2 | 22.8 | 96.8 | 51.876 | <0.001 |
|  | HSI | 0.786 (0.673-0.890) | 65.3 | 87.8 | 36.0 | 96.0 | 47.282 | 0.001 |
|  | NHHR | 0.575 (0.424-0.697) | 56.2 | 64.9 | 14.4 | 93.4 | 3.037 | 0.446 |
|  | NFS | 0.823 (0.726-0.906) | 63.0 | 88.6 | 36.7 | 95.8 | -0.235 | <0.001 |
|  | FIB-4 | 0.689 (0.557-0.812) | 66.6 | 63.9 | 16.3 | 94.8 | 0.984 | 0.001 |
|  | BARD | 0.808 (0.701-0.897) | 77.8 | 81.2 | 30.4 | 97.2 | 2.154 | 0.001 |
| Other Race | TyG | 0.610 (0.480-0.746) | 55.9 | 70.2 | 11.5 | 95.8 | 8.844 | 0.035 |
|  | TyG-BMI | 0.760 (0.652-0.852) | 76.6 | 71.3 | 15.6 | 97.8 | 250.769 | 0.001 |
|  | TyG-WC | 0.748 (0.636-0.841) | 66.5 | 72.8 | 14.5 | 96.9 | 877.710 | 0.004 |
|  | TyG-WHtR | 0.727 (0.608-0.837) | 67.6 | 68.7 | 13.0 | 96.8 | 5.269 | 0.008 |
|  | TyG-WWI | 0.652 (0.532-0.779) | 66.4 | 67.3 | 12.3 | 96.7 | 98.249 | 0.050 |
|  | VAI | 0.578 (0.453-0.700) | 59.4 | 67.8 | 11.3 | 96.0 | 1.800 | 0.347 |
|  | HOMA-IR | 0.717 (0.593-0.837) | 67.1 | 66.2 | 12.1 | 96.7 | 3.160 | 0.023 |
|  | METS-IR | 0.764 (0.647-0.852) | 76.6 | 73.3 | 16.6 | 97.8 | 44.575 | <0.001 |
|  | FSI | 0.765 (0.655-0.852) | 75.3 | 67.1 | 13.7 | 97.5 | -1.163 | <0.001 |
|  | FLI | 0.779 (0.673-0.874) | 78.0 | 70.4 | 15.4 | 97.9 | 58.655 | 0.003 |
|  | USFLI | 0.791 (0.672-0.893) | 65.8 | 77.8 | 17.0 | 97.0 | 34.477 | <0.001 |
|  | ZJU | 0.759 (0.631-0.860) | 77.3 | 72.2 | 16.2 | 97.9 | 41.288 | 0.001 |
|  | LAP | 0.670 (0.547-0.784) | 59.0 | 76.8 | 15.0 | 96.4 | 59.057 | 0.011 |
|  | HSI | 0.748 (0.618-0.848) | 73.7 | 72.7 | 15.8 | 97.5 | 39.547 | 0.001 |
|  | NHHR | 0.520 (0.392-0.641) | 45.3 | 67.2 | 8.7 | 94.7 | 3.193 | 0.630 |
|  | NFS | 0.735 (0.627-0.843) | 63.9 | 78.8 | 17.3 | 96.9 | -0.709 | 0.003 |
|  | FIB-4 | 0.581 (0.478-0.702) | 51.5 | 64.9 | 9.2 | 95.1 | 1.072 | 0.012 |
|  | BARD | 0.761 (0.689-0.824) | 83.5 | 60.8 | 12.9 | 98.2 | 1.731 | <0.001 |
| Age ≤50 | TyG | 0.685 (0.608-0.757) | 73.6 | 59.4 | 11.3 | 97.0 | 8.434 | <0.001 |
|  | TyG-BMI | 0.812 (0.715-0.886) | 75.5 | 81.1 | 21.9 | 97.9 | 292.239 | <0.001 |
|  | TyG-WC | 0.815 (0.724-0.892) | 77.4 | 81.2 | 22.3 | 98.1 | 949.669 | <0.001 |
|  | TyG-WHtR | 0.811 (0.705-0.887) | 77.6 | 78.0 | 19.8 | 98.0 | 5.483 | <0.001 |
|  | TyG-WWI | 0.759 (0.669-0.836) | 67.8 | 80.1 | 19.3 | 97.3 | 98.064 | <0.001 |
|  | VAI | 0.655 (0.565-0.733) | 67.8 | 63.5 | 11.5 | 96.6 | 1.411 | <0.001 |
|  | HOMA-IR | 0.765 (0.674-0.844) | 68.5 | 80.4 | 19.6 | 97.3 | 4.306 | <0.001 |
|  | METS-IR | 0.810 (0.716-0.885) | 76.2 | 80.0 | 21.1 | 98.0 | 51.212 | <0.001 |
|  | FSI | 0.816 (0.721-0.892) | 76.7 | 76.8 | 18.8 | 97.9 | -0.555 | <0.001 |
|  | FLI | 0.824 (0.726-0.901) | 81.1 | 76.4 | 19.4 | 98.3 | 77.482 | <0.001 |
|  | USFLI | 0.813 (0.722-0.887) | 72.1 | 83.7 | 23.6 | 97.7 | 40.955 | <0.001 |
|  | ZJU | 0.803 (0.716-0.878) | 77.5 | 75.2 | 17.9 | 97.9 | 43.618 | <0.001 |
|  | LAP | 0.754 (0.655-0.827) | 72.7 | 76.4 | 17.7 | 97.6 | 55.845 | <0.001 |
|  | HSI | 0.794 (0.700-0.875) | 73.2 | 76.6 | 17.9 | 97.6 | 43.300 | <0.001 |
|  | NHHR | 0.636 (0.548-0.716) | 63.1 | 66.7 | 11.7 | 96.3 | 3.019 | 0.065 |
|  | NFS | 0.783 (0.700-0.864) | 80.0 | 69.1 | 15.3 | 98.0 | -1.940 | <0.001 |
|  | FIB-4 | 0.601 (0.512-0.685) | 62.0 | 56.7 | 9.1 | 95.5 | 0.651 | 0.001 |
|  | BARD | 0.660 (0.589-0.730) | 79.4 | 50.2 | 10.0 | 97.2 | 1.610 | 0.001 |
| Age >50 | TyG | 0.650 (0.588-0.707) | 59.2 | 66.1 | 16.6 | 93.4 | 8.807 | <0.001 |
|  | TyG-BMI | 0.749 (0.675-0.807) | 70.2 | 71.0 | 21.6 | 95.4 | 273.489 | <0.001 |
|  | TyG-WC | 0.767 (0.701-0.824) | 70.9 | 73.5 | 23.4 | 95.7 | 960.545 | <0.001 |
|  | TyG-WHtR | 0.757 (0.690-0.818) | 74.9 | 68.3 | 21.2 | 96.0 | 5.642 | <0.001 |
|  | TyG-WWI | 0.711 (0.646-0.774) | 65.3 | 69.4 | 19.6 | 94.6 | 103.244 | <0.001 |
|  | VAI | 0.622 (0.567-0.676) | 60.7 | 62.6 | 15.6 | 93.3 | 1.675 | 0.024 |
|  | HOMA-IR | 0.704 (0.629-0.776) | 68.9 | 67.9 | 19.7 | 95.0 | 3.685 | 0.007 |
|  | METS-IR | 0.744 (0.682-0.805) | 73.6 | 66.2 | 19.9 | 95.6 | 45.216 | <0.001 |
|  | FSI | 0.744 (0.674-0.800) | 71.2 | 71.8 | 22.4 | 95.6 | -0.479 | <0.001 |
|  | FLI | 0.781 (0.716-0.836) | 69.2 | 76.0 | 24.8 | 95.6 | 83.036 | <0.001 |
|  | USFLI | 0.754 (0.680-0.822) | 70.9 | 70.0 | 21.2 | 95.5 | 38.056 | <0.001 |
|  | ZJU | 0.747 (0.670-0.809) | 67.8 | 74.3 | 23.2 | 95.3 | 44.302 | <0.001 |
|  | LAP | 0.703 (0.642-0.760) | 71.0 | 63.7 | 18.3 | 95.1 | 54.573 | <0.001 |
|  | HSI | 0.734 (0.659-0.796) | 66.8 | 74.6 | 23.1 | 95.2 | 42.114 | <0.001 |
|  | NHHR | 0.497 (0.434-0.562) | 54.7 | 51.7 | 11.4 | 90.9 | 2.500 | 0.946 |
|  | NFS | 0.727 (0.673-0.784) | 75.3 | 60.0 | 17.7 | 95.5 | -0.315 | <0.001 |
|  | FIB-4 | 0.568 (0.507-0.632) | 58.4 | 50.5 | 11.9 | 91.4 | 1.271 | <0.001 |
|  | BARD | 0.732 (0.673-0.790) | 63.8 | 71.4 | 20.3 | 94.5 | 2.107 | <0.001 |
| Never-smoker | TyG | 0.655 (0.585-0.719) | 60.5 | 65.5 | 13.6 | 94.9 | 8.652 | <0.001 |
|  | TyG-BMI | 0.755 (0.678-0.828) | 70.5 | 74.8 | 20.1 | 96.6 | 278.411 | <0.001 |
|  | TyG-WC | 0.779 (0.704-0.846) | 71.0 | 79.2 | 23.5 | 96.8 | 955.102 | <0.001 |
|  | TyG-WHtR | 0.772 (0.691-0.846) | 70.2 | 77.1 | 21.6 | 96.7 | 5.681 | <0.001 |
|  | TyG-WWI | 0.731 (0.657-0.797) | 69.3 | 68.7 | 16.6 | 96.1 | 98.064 | <0.001 |
|  | VAI | 0.623 (0.552-0.686) | 59.6 | 64.2 | 13.0 | 94.7 | 1.549 | 0.004 |
|  | HOMA-IR | 0.729 (0.654-0.795) | 72.6 | 67.3 | 16.6 | 96.5 | 3.287 | 0.004 |
|  | METS-IR | 0.749 (0.678-0.819) | 67.8 | 73.2 | 18.5 | 96.2 | 47.655 | <0.001 |
|  | FSI | 0.759 (0.681-0.825) | 71.9 | 74.0 | 19.9 | 96.7 | -0.555 | <0.001 |
|  | FLI | 0.783 (0.709-0.851) | 79.2 | 71.1 | 19.7 | 97.4 | 70.025 | <0.001 |
|  | USFLI | 0.781 (0.707-0.845) | 73.9 | 73.4 | 19.9 | 96.9 | 34.477 | <0.001 |
|  | ZJU | 0.748 (0.671-0.823) | 73.0 | 73.0 | 19.5 | 96.8 | 43.475 | <0.001 |
|  | LAP | 0.713 (0.641-0.777) | 65.2 | 72.8 | 17.7 | 95.9 | 55.845 | <0.001 |
|  | HSI | 0.736 (0.663-0.812) | 72.5 | 73.7 | 19.8 | 96.8 | 42.158 | <0.001 |
|  | NHHR | 0.539 (0.468-0.612) | 57.4 | 51.3 | 9.6 | 93.1 | 2.514 | 0.184 |
|  | NFS | 0.741 (0.674-0.804) | 65.1 | 77.7 | 20.7 | 96.1 | -0.519 | <0.001 |
|  | FIB-4 | 0.607 (0.543-0.668) | 63.1 | 59.3 | 12.2 | 94.7 | 1.001 | <0.001 |
|  | BARD | 0.667 (0.592-0.734) | 58.2 | 64.6 | 12.8 | 94.5 | 1.926 | <0.001 |
| Former smoker | TyG | 0.737 (0.665-0.804) | 75.2 | 60.9 | 15.8 | 96.2 | 8.680 | <0.001 |
|  | TyG-BMI | 0.847 (0.786-0.896) | 76.0 | 83.1 | 30.5 | 97.3 | 302.735 | <0.001 |
|  | TyG-WC | 0.868 (0.817-0.907) | 82.1 | 77.6 | 26.4 | 97.8 | 978.795 | <0.001 |
|  | TyG-WHtR | 0.858 (0.803-0.901) | 86.4 | 73.8 | 24.4 | 98.2 | 5.624 | <0.001 |
|  | TyG-WWI | 0.804 (0.749-0.854) | 72.5 | 76.1 | 22.8 | 96.6 | 103.172 | <0.001 |
|  | VAI | 0.725 (0.650-0.793) | 75.1 | 68.8 | 19.1 | 96.6 | 1.749 | 0.014 |
|  | HOMA-IR | 0.788 (0.705-0.857) | 73.1 | 76.9 | 23.6 | 96.7 | 4.306 | <0.001 |
|  | METS-IR | 0.844 (0.783-0.890) | 75.2 | 83.3 | 30.6 | 97.2 | 53.516 | <0.001 |
|  | FSI | 0.859 (0.804-0.902) | 76.0 | 82.2 | 29.5 | 97.2 | 0.155 | <0.001 |
|  | FLI | 0.870 (0.818-0.914) | 75.9 | 85.3 | 33.5 | 97.3 | 90.086 | <0.001 |
|  | USFLI | 0.855 (0.790-0.907) | 80.4 | 72.6 | 22.3 | 97.4 | 40.485 | <0.001 |
|  | ZJU | 0.843 (0.783-0.892) | 78.0 | 79.0 | 26.6 | 97.4 | 45.551 | <0.001 |
|  | LAP | 0.813 (0.754-0.858) | 89.1 | 66.9 | 20.8 | 98.4 | 54.573 | 0.004 |
|  | HSI | 0.825 (0.757-0.879) | 72.4 | 80.0 | 26.2 | 96.7 | 44.033 | <0.001 |
|  | NHHR | 0.623 (0.528-0.710) | 60.5 | 60.8 | 13.1 | 94.0 | 2.767 | 0.042 |
|  | NFS | 0.798 (0.724-0.853) | 80.4 | 65.8 | 18.7 | 97.2 | -0.657 | <0.001 |
|  | FIB-4 | 0.618 (0.528-0.709) | 72.7 | 54.4 | 13.5 | 95.3 | 1.023 | <0.001 |
|  | BARD | 0.768 (0.693-0.843) | 72.9 | 67.4 | 17.9 | 96.2 | 1.941 | <0.001 |
| Current smoker | TyG | 0.595 (0.480-0.736) | 66.4 | 45.6 | 9.3 | 94.2 | 8.434 | 0.028 |
|  | TyG-BMI | 0.730 (0.602-0.832) | 79.0 | 63.4 | 15.3 | 97.3 | 252.039 | <0.001 |
|  | TyG-WC | 0.712 (0.596-0.811) | 78.3 | 66.1 | 16.2 | 97.3 | 892.771 | <0.001 |
|  | TyG-WHtR | 0.726 (0.608-0.831) | 81.7 | 63.6 | 15.8 | 97.6 | 5.261 | <0.001 |
|  | TyG-WWI | 0.634 (0.521-0.769) | 72.9 | 55.0 | 12.0 | 96.0 | 94.054 | 0.044 |
|  | VAI | 0.536 (0.439-0.651) | 60.8 | 49.6 | 9.2 | 93.8 | 1.374 | 0.193 |
|  | HOMA-IR | 0.618 (0.407-0.805) | 56.4 | 79.7 | 18.9 | 95.6 | 4.224 | 0.109 |
|  | METS-IR | 0.701 (0.575-0.809) | 76.2 | 60.0 | 13.8 | 96.8 | 42.714 | <0.001 |
|  | FSI | 0.725 (0.610-0.815) | 69.6 | 70.9 | 16.7 | 96.5 | -0.844 | <0.001 |
|  | FLI | 0.742 (0.637-0.843) | 83.0 | 61.9 | 15.5 | 97.8 | 61.457 | <0.001 |
|  | USFLI | 0.673 (0.505-0.832) | 65.2 | 77.7 | 19.7 | 96.4 | 36.251 | 0.004 |
|  | ZJU | 0.730 (0.617-0.828) | 74.7 | 70.6 | 17.6 | 97.1 | 42.295 | <0.001 |
|  | LAP | 0.652 (0.541-0.751) | 58.5 | 71.4 | 14.6 | 95.4 | 59.134 | 0.020 |
|  | HSI | 0.700 (0.574-0.803) | 71.9 | 64.4 | 14.5 | 96.5 | 39.000 | <0.001 |
|  | NHHR | 0.540 (0.378-0.682) | 46.7 | 73.9 | 13.0 | 94.3 | 1.962 | 0.644 |
|  | NFS | 0.749 (0.652-0.845) | 60.8 | 75.0 | 16.9 | 95.8 | -0.742 | <0.001 |
|  | FIB-4 | 0.602 (0.427-0.765) | 49.8 | 81.8 | 18.7 | 95.1 | 1.263 | 0.005 |
|  | BARD | 0.759 (0.652-0.838) | 73.1 | 77.2 | 21.2 | 97.2 | 2.055 | <0.001 |
| Non-Obese | TyG | 0.607 (0.486-0.715) | 59.1 | 61.4 | 4.8 | 97.9 | 8.466 | 0.074 |
|  | TyG-BMI | 0.573 (0.455-0.701) | 48.9 | 69.2 | 4.9 | 97.6 | 229.437 | 0.203 |
|  | TyG-WC | 0.609 (0.490-0.739) | 53.1 | 69.5 | 5.4 | 97.8 | 813.613 | 0.050 |
|  | TyG-WHtR | 0.600 (0.477-0.731) | 51.6 | 75.0 | 6.3 | 97.9 | 4.955 | 0.076 |
|  | TyG-WWI | 0.614 (0.495-0.729) | 49.5 | 77.0 | 6.6 | 97.9 | 97.326 | 0.054 |
|  | VAI | 0.559 (0.437-0.670) | 52.0 | 61.9 | 4.3 | 97.5 | 1.208 | 0.049 |
|  | HOMA-IR | 0.557 (0.448-0.671) | 50.6 | 59.9 | 4.0 | 97.4 | 2.066 | 0.044 |
|  | METS-IR | 0.565 (0.455-0.683) | 40.6 | 78.2 | 5.8 | 97.6 | 41.045 | 0.123 |
|  | FSI | 0.611 (0.500-0.738) | 54.6 | 63.6 | 4.7 | 97.7 | -1.847 | 0.009 |
|  | FLI | 0.638 (0.509-0.775) | 53.7 | 71.6 | 5.8 | 97.9 | 42.553 | 0.004 |
|  | USFLI | 0.641 (0.523-0.769) | 56.0 | 73.7 | 6.5 | 98.1 | 20.560 | <0.001 |
|  | ZJU | 0.528 (0.412-0.661) | 48.9 | 62.5 | 4.1 | 97.4 | 37.174 | 0.382 |
|  | LAP | 0.581 (0.466-0.703) | 51.7 | 63.7 | 4.4 | 97.6 | 33.421 | 0.014 |
|  | HSI | 0.498 (0.384-0.621) | 54.2 | 56.4 | 3.9 | 97.4 | 33.991 | 0.991 |
|  | NHHR | 0.487 (0.387-0.599) | 43.3 | 59.7 | 3.4 | 97.0 | 2.509 | 0.959 |
|  | NFS | 0.685 (0.576-0.784) | 65.5 | 63.7 | 5.6 | 98.3 | -1.503 | 0.001 |
|  | FIB-4 | 0.682 (0.567-0.781) | 75.7 | 59.9 | 5.8 | 98.7 | 1.056 | <0.001 |
|  | BARD | 0.606 (0.519-0.707) | 56.9 | 63.9 | 4.9 | 97.8 | 1.586 | 0.017 |
| Obese | TyG | 0.618 (0.557-0.674) | 65.7 | 53.2 | 21.3 | 89.0 | 8.724 | <0.001 |
|  | TyG-BMI | 0.722 (0.664-0.776) | 70.5 | 66.3 | 28.7 | 92.1 | 317.755 | <0.001 |
|  | TyG-WC | 0.755 (0.698-0.801) | 61.4 | 77.6 | 34.6 | 91.2 | 1079.414 | <0.001 |
|  | TyG-WHtR | 0.752 (0.696-0.805) | 73.5 | 67.8 | 30.5 | 93.0 | 6.155 | <0.001 |
|  | TyG-WWI | 0.694 (0.641-0.749) | 62.4 | 70.2 | 28.8 | 90.6 | 104.390 | <0.001 |
|  | VAI | 0.562 (0.509-0.618) | 67.1 | 48.8 | 20.2 | 88.5 | 1.675 | 0.206 |
|  | HOMA-IR | 0.692 (0.626-0.750) | 74.5 | 58.0 | 25.5 | 92.2 | 4.277 | 0.006 |
|  | METS-IR | 0.702 (0.643-0.762) | 72.7 | 61.3 | 26.6 | 92.1 | 54.781 | <0.001 |
|  | FSI | 0.719 (0.663-0.773) | 73.6 | 61.8 | 27.1 | 92.4 | 0.242 | <0.001 |
|  | FLI | 0.748 (0.694-0.807) | 78.2 | 64.7 | 29.9 | 93.9 | 90.578 | <0.001 |
|  | USFLI | 0.743 (0.683-0.799) | 66.3 | 72.6 | 31.8 | 91.8 | 52.038 | <0.001 |
|  | ZJU | 0.727 (0.669-0.780) | 65.2 | 74.4 | 32.9 | 91.7 | 50.032 | <0.001 |
|  | LAP | 0.648 (0.592-0.700) | 65.9 | 59.0 | 23.7 | 90.0 | 72.732 | 0.001 |
|  | HSI | 0.707 (0.644-0.762) | 61.2 | 72.6 | 30.1 | 90.7 | 48.200 | <0.001 |
|  | NHHR | 0.509 (0.446-0.577) | 50.3 | 53.6 | 17.3 | 84.8 | 2.791 | 0.764 |
|  | NFS | 0.709 (0.655-0.757) | 70.2 | 65.4 | 28.1 | 91.9 | -0.315 | <0.001 |
|  | FIB-4 | 0.622 (0.563-0.678) | 59.6 | 63.1 | 23.7 | 89.0 | 1.015 | <0.001 |
|  | BARD | 0.610 (0.543-0.678) | 51.9 | 77.2 | 30.5 | 89.3 | 2.409 | <0.001 |
| Non-Hypertension | TyG | 0.649 (0.561-0.728) | 69.7 | 59.6 | 7.9 | 97.5 | 8.434 | 0.012 |
|  | TyG-BMI | 0.741 (0.615-0.847) | 71.7 | 73.8 | 12.0 | 98.1 | 263.654 | <0.001 |
|  | TyG-WC | 0.750 (0.638-0.847) | 69.0 | 81.3 | 15.5 | 98.1 | 935.471 | <0.001 |
|  | TyG-WHtR | 0.739 (0.616-0.849) | 67.1 | 78.8 | 13.6 | 98.0 | 5.492 | 0.001 |
|  | TyG-WWI | 0.684 (0.590-0.773) | 61.8 | 71.0 | 9.6 | 97.4 | 96.382 | 0.006 |
|  | VAI | 0.603 (0.518-0.682) | 67.6 | 54.5 | 6.9 | 97.1 | 1.147 | 0.173 |
|  | HOMA-IR | 0.726 (0.625-0.820) | 62.5 | 84.1 | 16.4 | 97.8 | 4.277 | <0.001 |
|  | METS-IR | 0.741 (0.627-0.840) | 72.0 | 71.9 | 11.3 | 98.1 | 45.346 | <0.001 |
|  | FSI | 0.740 (0.622-0.839) | 67.5 | 73.4 | 11.2 | 97.8 | -1.200 | <0.001 |
|  | FLI | 0.759 (0.633-0.866) | 70.4 | 76.9 | 13.2 | 98.1 | 70.352 | 0.001 |
|  | USFLI | 0.759 (0.648-0.854) | 72.9 | 75.8 | 13.0 | 98.2 | 29.829 | <0.001 |
|  | ZJU | 0.725 (0.596-0.844) | 68.6 | 79.2 | 14.1 | 98.1 | 43.080 | <0.001 |
|  | LAP | 0.687 (0.582-0.782) | 63.5 | 71.2 | 9.9 | 97.5 | 47.606 | 0.003 |
|  | HSI | 0.711 (0.592-0.825) | 64.7 | 77.0 | 12.3 | 97.8 | 41.442 | <0.001 |
|  | NHHR | 0.560 (0.467-0.645) | 60.8 | 54.4 | 6.2 | 96.5 | 2.523 | 0.166 |
|  | NFS | 0.712 (0.611-0.798) | 82.8 | 54.5 | 8.3 | 98.5 | -1.986 | <0.001 |
|  | FIB-4 | 0.580 (0.485-0.678) | 59.4 | 56.8 | 6.4 | 96.6 | 0.850 | <0.001 |
|  | BARD | 0.656 (0.571-0.740) | 72.3 | 55.1 | 7.4 | 97.6 | 1.696 | 0.004 |
| Hypertension | TyG | 0.640 (0.579-0.700) | 52.5 | 73.6 | 24.7 | 90.4 | 9.014 | <0.001 |
|  | TyG-BMI | 0.758 (0.702-0.807) | 66.3 | 77.1 | 32.3 | 93.3 | 309.459 | <0.001 |
|  | TyG-WC | 0.778 (0.728-0.826) | 74.0 | 72.0 | 30.3 | 94.4 | 992.930 | <0.001 |
|  | TyG-WHtR | 0.775 (0.724-0.824) | 71.9 | 70.5 | 28.7 | 93.8 | 5.886 | <0.001 |
|  | TyG-WWI | 0.722 (0.665-0.778) | 67.3 | 67.4 | 25.4 | 92.6 | 103.244 | <0.001 |
|  | VAI | 0.620 (0.563-0.675) | 68.8 | 56.2 | 20.6 | 91.6 | 1.678 | 0.003 |
|  | HOMA-IR | 0.699 (0.629-0.761) | 58.4 | 75.5 | 28.2 | 91.7 | 5.144 | 0.004 |
|  | METS-IR | 0.750 (0.693-0.804) | 63.4 | 79.5 | 33.8 | 92.9 | 55.455 | <0.001 |
|  | FSI | 0.748 (0.699-0.797) | 68.2 | 71.6 | 28.4 | 93.2 | 0.191 | <0.001 |
|  | FLI | 0.786 (0.732-0.832) | 68.3 | 79.4 | 35.3 | 93.8 | 89.311 | <0.001 |
|  | USFLI | 0.763 (0.700-0.823) | 67.0 | 75.1 | 30.8 | 93.2 | 46.972 | <0.001 |
|  | ZJU | 0.759 (0.701-0.811) | 63.0 | 78.0 | 32.1 | 92.7 | 48.272 | <0.001 |
|  | LAP | 0.713 (0.657-0.761) | 68.5 | 67.7 | 25.9 | 92.9 | 66.319 | <0.001 |
|  | HSI | 0.746 (0.687-0.802) | 70.8 | 67.5 | 26.4 | 93.3 | 43.300 | <0.001 |
|  | NHHR | 0.533 (0.467-0.601) | 52.2 | 56.7 | 16.6 | 87.8 | 2.766 | 0.141 |
|  | NFS | 0.714 (0.663-0.764) | 69.6 | 63.6 | 24.0 | 92.7 | -0.316 | <0.001 |
|  | FIB-4 | 0.555 (0.496-0.610) | 68.8 | 44.7 | 17.0 | 89.7 | 1.030 | <0.001 |
|  | BARD | 0.684 (0.628-0.737) | 57.9 | 72.6 | 25.8 | 91.3 | 2.222 | <0.001 |

TyG: triglyceride-glucose index; BMI: body mass index; WC: waist circumference; WHtR: waist-to-height ratio; WWI: weight-adjusted waist index; VAI: visceral adiposity index; HOMA-IR: homeostatic model assessment of insulin resistance; METS-IR: metabolic score for insulin resistance; FSI: Framingham steatosis index; FLI: fatty liver index; ZJU: Zhejiang University index; LAP: lipid accumulation product; HSI: hepatic steatosis index; NHHR: non-high-density lipoprotein cholesterol (HDL-C) to HDL-C ratio; NFS: nonalcoholic fatty liver disease fibrosis score; FIB-4: fibrosis-4 index; BARD: BMI-aspartate aminotransferase/alanine aminotransferase ratio and diabetes score

**Table. S8**. Diagnostic efficacy of 18 indices for significant fibrosis in patients with normoglycemia across different demographic characteristics

| Group | Variables | AUC (95%CI) | Sensitivity (%) | Specificity (%) | PPV | NPV | Cut-off Values | p-Value |
| --- | --- | --- | --- | --- | --- | --- | --- | --- |
| Female | TyG | 0.692 (0.557-0.837) | 64.7 | 65.6 | 3.8 | 98.9 | 8.273 | 0.029 |
|  | TyG-BMI | 0.733 (0.530-0.911) | 65.1 | 84.7 | 8.2 | 99.1 | 284.569 | 0.008 |
|  | TyG-WC | 0.790 (0.628-0.950) | 65.1 | 91.2 | 13.4 | 99.2 | 975.376 | 0.006 |
|  | TyG-WHtR | 0.823 (0.694-0.947) | 87.7 | 64.3 | 4.9 | 99.6 | 4.955 | 0.001 |
|  | TyG-WWI | 0.847 (0.728-0.931) | 83.4 | 81.4 | 8.6 | 99.6 | 98.220 | <0.001 |
|  | VAI | 0.690 (0.545-0.850) | 69.0 | 69.2 | 4.5 | 99.1 | 1.391 | 0.099 |
|  | HOMA-IR | 0.732 (0.594-0.867) | 67.8 | 71.3 | 4.7 | 99.1 | 2.377 | 0.008 |
|  | METS-IR | 0.700 (0.474-0.905) | 65.1 | 75.5 | 5.3 | 99.0 | 44.203 | 0.013 |
|  | FSI | 0.810 (0.682-0.935) | 65.1 | 89.1 | 11.1 | 99.2 | -0.249 | <0.001 |
|  | FLI | 0.805 (0.650-0.952) | 65.1 | 92.9 | 16.0 | 99.2 | 91.886 | 0.043 |
|  | USFLI | 0.861 (0.771-0.940) | 87.7 | 80.0 | 8.4 | 99.7 | 20.560 | <0.001 |
|  | ZJU | 0.708 (0.498-0.896) | 65.1 | 82.0 | 7.0 | 99.1 | 43.808 | 0.012 |
|  | LAP | 0.764 (0.596-0.914) | 60.9 | 88.3 | 9.8 | 99.1 | 69.978 | 0.015 |
|  | HSI | 0.674 (0.465-0.876) | 58.5 | 86.2 | 8.1 | 99.0 | 45.129 | 0.028 |
|  | NHHR | 0.527 (0.314-0.755) | 58.6 | 46.7 | 2.2 | 98.2 | 2.037 | 0.766 |
|  | NFS | 0.812 (0.609-0.933) | 77.0 | 78.9 | 7.1 | 99.4 | -1.160 | <0.001 |
|  | FIB-4 | 0.749 (0.503-0.891) | 74.3 | 70.0 | 4.9 | 99.2 | 1.066 | <0.001 |
|  | BARD | 0.691 (0.563-0.816) | 93.1 | 49.0 | 3.7 | 99.7 | 1.600 | 0.006 |
| Male | TyG | 0.538 (0.338-0.736) | 57.4 | 51.8 | 7.4 | 94.8 | 8.223 | 0.576 |
|  | TyG-BMI | 0.622 (0.414-0.820) | 68.9 | 65.2 | 11.6 | 96.9 | 232.727 | 0.026 |
|  | TyG-WC | 0.641 (0.409-0.837) | 63.1 | 68.4 | 11.7 | 96.5 | 850.555 | 0.040 |
|  | TyG-WHtR | 0.652 (0.422-0.853) | 59.6 | 82.1 | 18.2 | 96.8 | 5.158 | 0.051 |
|  | TyG-WWI | 0.605 (0.386-0.788) | 56.4 | 78.5 | 14.9 | 96.4 | 93.198 | 0.349 |
|  | VAI | 0.514 (0.332-0.709) | 50.5 | 60.6 | 7.9 | 94.8 | 0.781 | 0.891 |
|  | HOMA-IR | 0.486 (0.293-0.665) | 40.8 | 69.4 | 8.2 | 94.6 | 2.394 | 0.525 |
|  | METS-IR | 0.616 (0.414-0.802) | 61.2 | 69.8 | 11.9 | 96.4 | 42.267 | 0.023 |
|  | FSI | 0.672 (0.445-0.871) | 63.1 | 81.9 | 18.8 | 97.1 | -0.889 | 0.006 |
|  | FLI | 0.663 (0.422-0.863) | 63.1 | 79.0 | 16.7 | 97.0 | 70.025 | 0.067 |
|  | USFLI | 0.598 (0.371-0.801) | 55.9 | 73.0 | 12.1 | 96.1 | 20.389 | 0.017 |
|  | ZJU | 0.630 (0.444-0.825) | 68.9 | 63.9 | 11.3 | 96.9 | 37.449 | 0.015 |
|  | LAP | 0.606 (0.388-0.801) | 56.5 | 75.9 | 13.5 | 96.3 | 44.810 | 0.048 |
|  | HSI | 0.633 (0.457-0.816) | 72.4 | 61.0 | 11.0 | 97.1 | 35.200 | 0.017 |
|  | NHHR | 0.529 (0.341-0.712) | 50.2 | 61.7 | 8.0 | 94.9 | 2.349 | 0.692 |
|  | NFS | 0.681 (0.456-0.853) | 60.1 | 80.3 | 16.9 | 96.8 | -1.503 | 0.044 |
|  | FIB-4 | 0.623 (0.436-0.778) | 62.9 | 71.8 | 13.0 | 96.7 | 1.023 | 0.017 |
|  | BARD | 0.598 (0.472-0.738) | 67.8 | 59.9 | 10.1 | 96.5 | 1.659 | 0.177 |
| Non-Hispanic Black | TyG | 0.657 (0.521-0.758) | 71.2 | 63.3 | 5.0 | 98.8 | 8.066 | 0.048 |
|  | TyG-BMI | 0.666 (0.345-0.912) | 76.1 | 63.8 | 5.4 | 99.0 | 250.612 | 0.058 |
|  | TyG-WHtR | 0.629 (0.332-0.910) | 52.2 | 87.3 | 10.0 | 98.5 | 5.681 | 0.199 |
|  | TyG-WWI | 0.551 (0.333-0.759) | 76.1 | 45.2 | 3.6 | 98.6 | 81.194 | 0.667 |
|  | VAI | 0.442 (0.283-0.597) | 49.5 | 55.6 | 2.9 | 97.6 | 0.692 | 0.622 |
|  | FSI | 0.679 (0.391-0.943) | 54.9 | 85.4 | 9.2 | 98.6 | -0.501 | 0.044 |
|  | USFLI | 0.630 (0.406-0.857) | 76.1 | 57.0 | 4.6 | 98.9 | 6.417 | 0.138 |
|  | ZJU | 0.642 (0.361-0.906) | 76.1 | 60.7 | 5.0 | 98.9 | 40.204 | 0.121 |
|  | HSI | 0.664 (0.374-0.892) | 73.5 | 68.8 | 6.0 | 99.0 | 40.600 | 0.090 |
|  | NFS | 0.466 (0.219-0.766) | 39.9 | 70.2 | 3.5 | 97.7 | -1.471 | 0.968 |
|  | FIB-4 | 0.569 (0.354-0.775) | 67.4 | 56.4 | 4.0 | 98.5 | 0.651 | 0.263 |
|  | BARD | 0.550 (0.301-0.777) | 47.6 | 64.0 | 3.4 | 97.8 | 2.077 | 0.665 |
| Non-Hispanic White | TyG | 0.561 (0.345-0.784) | 62.4 | 55.9 | 5.3 | 97.4 | 8.223 | 0.865 |
|  | TyG-BMI | 0.629 (0.405-0.836) | 65.3 | 63.8 | 6.7 | 97.9 | 232.727 | 0.087 |
|  | TyG-WC | 0.691 (0.475-0.891) | 59.3 | 83.5 | 12.5 | 98.1 | 923.368 | 0.045 |
|  | TyG-WHtR | 0.691 (0.458-0.882) | 71.3 | 68.4 | 8.2 | 98.4 | 4.955 | 0.098 |
|  | TyG-WWI | 0.693 (0.452-0.892) | 71.3 | 71.3 | 9.0 | 98.4 | 94.533 | 0.238 |
|  | VAI | 0.549 (0.334-0.766) | 50.9 | 71.0 | 6.5 | 97.3 | 1.391 | 0.626 |
|  | HOMA-IR | 0.583 (0.373-0.794) | 63.6 | 62.3 | 6.3 | 97.7 | 1.834 | 0.130 |
|  | METS-IR | 0.623 (0.427-0.831) | 57.6 | 73.3 | 7.9 | 97.7 | 42.878 | 0.058 |
|  | FSI | 0.716 (0.494-0.903) | 65.3 | 81.9 | 12.6 | 98.3 | -0.889 | 0.005 |
|  | FLI | 0.707 (0.481-0.916) | 66.7 | 80.0 | 11.7 | 98.4 | 68.374 | 0.092 |
|  | USFLI | 0.725 (0.476-0.905) | 77.3 | 77.9 | 12.3 | 98.9 | 20.389 | <0.001 |
|  | ZJU | 0.621 (0.407-0.830) | 65.3 | 60.2 | 6.1 | 97.8 | 37.449 | 0.136 |
|  | LAP | 0.653 (0.428-0.861) | 59.3 | 80.1 | 10.6 | 98.0 | 51.450 | 0.018 |
|  | HSI | 0.617 (0.432-0.815) | 65.3 | 59.2 | 6.0 | 97.7 | 35.200 | 0.131 |
|  | NHHR | 0.514 (0.310-0.706) | 42.9 | 77.6 | 7.1 | 97.2 | 1.471 | 0.953 |
|  | NFS | 0.726 (0.495-0.924) | 72.7 | 71.8 | 9.3 | 98.5 | -1.503 | 0.040 |
|  | FIB-4 | 0.693 (0.496-0.872) | 77.4 | 66.0 | 8.3 | 98.7 | 1.023 | <0.001 |
|  | BARD | 0.579 (0.427-0.753) | 71.6 | 52.5 | 5.7 | 97.9 | 1.586 | 0.397 |
| Other Hispanic | TyG | 0.656 (0.377-0.909) | 81.3 | 56.8 | 10.5 | 98.0 | 8.392 | 0.189 |
|  | TyG-WHtR | 0.784 (0.563-0.954) | 76.4 | 76.5 | 16.9 | 98.1 | 5.219 | 0.039 |
|  | VAI | 0.672 (0.464-0.874) | 81.3 | 66.9 | 13.3 | 98.3 | 1.368 | 0.168 |
|  | HOMA-IR | 0.583 (0.243-0.853) | 50.2 | 83.2 | 15.7 | 96.4 | 3.576 | 0.190 |
|  | METS-IR | 0.791 (0.602-0.933) | 62.8 | 91.9 | 32.7 | 97.5 | 51.700 | 0.017 |
|  | FLI | 0.784 (0.586-0.937) | 76.4 | 70.7 | 14.0 | 98.0 | 57.198 | 0.053 |
|  | USFLI | 0.672 (0.358-0.948) | 50.2 | 98.1 | 62.2 | 96.9 | 53.254 | 0.060 |
|  | ZJU | 0.687 (0.482-0.878) | 62.8 | 78.7 | 15.6 | 97.1 | 43.475 | 0.080 |
|  | HSI | 0.622 (0.416-0.824) | 62.8 | 58.7 | 8.7 | 96.2 | 37.900 | 0.201 |
|  | NHHR | 0.626 (0.383-0.851) | 56.5 | 76.7 | 13.1 | 96.6 | 3.328 | 0.694 |
| Age ≤50 | TyG | 0.556 (0.325-0.769) | 60.0 | 56.7 | 3.8 | 98.0 | 8.158 | 0.756 |
|  | TyG-BMI | 0.644 (0.410-0.872) | 66.1 | 71.3 | 6.2 | 98.7 | 248.446 | 0.028 |
|  | TyG-WC | 0.675 (0.419-0.901) | 59.0 | 84.6 | 9.9 | 98.6 | 923.368 | 0.024 |
|  | TyG-WHtR | 0.642 (0.379-0.877) | 62.1 | 80.0 | 8.2 | 98.7 | 5.158 | 0.078 |
|  | TyG-WWI | 0.594 (0.345-0.816) | 54.7 | 78.0 | 6.7 | 98.4 | 93.198 | 0.358 |
|  | VAI | 0.556 (0.323-0.785) | 48.7 | 76.2 | 5.5 | 98.1 | 1.484 | 0.326 |
|  | HOMA-IR | 0.546 (0.306-0.773) | 51.4 | 70.3 | 4.7 | 98.1 | 2.394 | 0.066 |
|  | METS-IR | 0.667 (0.456-0.867) | 70.5 | 65.9 | 5.6 | 98.7 | 41.398 | 0.010 |
|  | FSI | 0.682 (0.426-0.898) | 59.0 | 85.7 | 10.6 | 98.6 | -0.632 | 0.005 |
|  | FLI | 0.673 (0.410-0.891) | 66.1 | 73.6 | 6.7 | 98.7 | 57.198 | 0.062 |
|  | USFLI | 0.615 (0.362-0.856) | 51.4 | 84.0 | 8.4 | 98.4 | 25.431 | 0.004 |
|  | ZJU | 0.615 (0.362-0.838) | 66.1 | 63.3 | 4.9 | 98.5 | 37.909 | 0.068 |
|  | LAP | 0.628 (0.375-0.867) | 58.8 | 75.6 | 6.5 | 98.5 | 42.772 | 0.006 |
|  | HSI | 0.611 (0.394-0.838) | 70.5 | 58.8 | 4.7 | 98.6 | 35.200 | 0.073 |
|  | NHHR | 0.610 (0.415-0.799) | 52.0 | 78.6 | 6.5 | 98.3 | 3.146 | 0.180 |
|  | NFS | 0.645 (0.401-0.861) | 63.0 | 69.6 | 5.6 | 98.5 | -2.145 | 0.157 |
|  | FIB-4 | 0.628 (0.429-0.819) | 64.7 | 63.3 | 4.8 | 98.4 | 0.687 | 0.081 |
|  | BARD | 0.533 (0.388-0.713) | 58.3 | 58.8 | 3.9 | 98.0 | 1.684 | 0.562 |
| Age >50 | TyG | 0.510 (0.363-0.672) | 66.7 | 44.8 | 6.6 | 95.8 | 8.223 | 0.862 |
|  | TyG-BMI | 0.658 (0.457-0.831) | 49.6 | 88.8 | 20.7 | 96.8 | 284.569 | 0.033 |
|  | TyG-WC | 0.710 (0.506-0.871) | 57.0 | 90.5 | 26.1 | 97.3 | 976.149 | 0.023 |
|  | TyG-WHtR | 0.725 (0.580-0.853) | 60.3 | 82.9 | 17.2 | 97.3 | 5.691 | 0.007 |
|  | TyG-WWI | 0.711 (0.521-0.852) | 67.0 | 73.3 | 12.8 | 97.4 | 99.888 | 0.022 |
|  | VAI | 0.516 (0.365-0.696) | 50.8 | 62.5 | 7.4 | 95.6 | 1.368 | 0.930 |
|  | HOMA-IR | 0.584 (0.430-0.736) | 63.5 | 58.6 | 8.2 | 96.5 | 1.834 | 0.190 |
|  | METS-IR | 0.642 (0.458-0.840) | 58.5 | 75.3 | 12.2 | 96.9 | 42.878 | 0.046 |
|  | FSI | 0.746 (0.587-0.882) | 69.2 | 78.4 | 15.8 | 97.7 | -0.889 | 0.003 |
|  | FLI | 0.752 (0.587-0.900) | 71.1 | 78.9 | 16.5 | 97.9 | 68.374 | 0.039 |
|  | USFLI | 0.759 (0.649-0.856) | 84.3 | 66.8 | 13.0 | 98.6 | 20.389 | 0.001 |
|  | ZJU | 0.657 (0.481-0.831) | 58.5 | 78.1 | 13.6 | 97.0 | 41.144 | 0.042 |
|  | LAP | 0.642 (0.470-0.807) | 60.3 | 75.9 | 12.8 | 97.0 | 51.450 | 0.094 |
|  | HSI | 0.656 (0.461-0.827) | 58.0 | 85.9 | 19.4 | 97.2 | 41.645 | 0.052 |
|  | NHHR | 0.613 (0.426-0.784) | 56.8 | 59.4 | 7.6 | 95.9 | 2.037 | 0.193 |
|  | NFS | 0.783 (0.623-0.901) | 76.5 | 70.4 | 13.2 | 98.1 | -0.588 | <0.001 |
|  | FIB-4 | 0.672 (0.494-0.834) | 68.4 | 62.1 | 9.6 | 97.1 | 1.517 | 0.009 |
|  | BARD | 0.673 (0.549-0.788) | 94.8 | 46.6 | 9.4 | 99.4 | 1.586 | 0.008 |
| Never-smoker | TyG | 0.533 (0.362-0.703) | 58.4 | 52.4 | 5.1 | 96.6 | 8.158 | 0.917 |
|  | TyG-BMI | 0.654 (0.466-0.845) | 63.0 | 70.8 | 8.7 | 97.8 | 248.446 | 0.010 |
|  | TyG-WC | 0.697 (0.530-0.870) | 63.2 | 72.0 | 9.0 | 97.8 | 850.555 | 0.008 |
|  | TyG-WHtR | 0.687 (0.482-0.859) | 69.4 | 67.7 | 8.6 | 98.1 | 4.955 | 0.022 |
|  | TyG-WWI | 0.652 (0.462-0.818) | 63.8 | 72.2 | 9.2 | 97.8 | 93.198 | 0.140 |
|  | VAI | 0.537 (0.371-0.696) | 48.6 | 69.4 | 6.5 | 96.8 | 1.368 | 0.822 |
|  | HOMA-IR | 0.549 (0.378-0.721) | 50.6 | 67.9 | 6.5 | 96.9 | 2.377 | 0.266 |
|  | METS-IR | 0.661 (0.505-0.847) | 68.3 | 68.9 | 8.8 | 98.0 | 42.267 | 0.005 |
|  | FSI | 0.717 (0.524-0.892) | 63.2 | 82.8 | 13.9 | 98.1 | -0.632 | <0.001 |
|  | FLI | 0.703 (0.512-0.893) | 66.0 | 76.4 | 10.9 | 98.1 | 64.638 | 0.028 |
|  | USFLI | 0.680 (0.489-0.843) | 65.0 | 75.5 | 10.5 | 98.0 | 20.389 | 0.003 |
|  | ZJU | 0.642 (0.446-0.833) | 66.0 | 63.2 | 7.3 | 97.7 | 38.280 | 0.019 |
|  | LAP | 0.645 (0.461-0.830) | 57.6 | 73.8 | 8.8 | 97.5 | 42.772 | 0.012 |
|  | HSI | 0.630 (0.453-0.831) | 53.9 | 76.4 | 9.1 | 97.4 | 40.600 | 0.022 |
|  | NHHR | 0.536 (0.361-0.730) | 46.7 | 68.4 | 6.1 | 96.7 | 2.633 | 0.587 |
|  | NFS | 0.697 (0.493-0.873) | 68.2 | 75.4 | 10.9 | 98.2 | -1.503 | 0.018 |
|  | FIB-4 | 0.643 (0.472-0.800) | 59.2 | 71.1 | 8.3 | 97.5 | 1.046 | 0.004 |
|  | BARD | 0.568 (0.444-0.727) | 73.6 | 50.6 | 6.1 | 97.8 | 1.600 | 0.316 |
| Former smoker | TyG-BMI | 0.778 (0.509-0.943) | 81.8 | 61.8 | 6.8 | 99.0 | 240.114 | 0.012 |
|  | TyG-WC | 0.878 (0.588-0.986) | 81.8 | 97.5 | 52.3 | 99.4 | 997.461 | <0.001 |
|  | TyG-WWI | 0.841 (0.461-0.979) | 81.8 | 86.8 | 17.5 | 99.3 | 98.220 | 0.005 |
|  | HOMA-IR | 0.603 (0.203-0.873) | 56.4 | 80.3 | 8.9 | 98.2 | 2.624 | 0.043 |
|  | METS-IR | 0.764 (0.463-0.947) | 68.0 | 72.8 | 7.9 | 98.5 | 41.398 | 0.031 |
|  | USFLI | 0.838 (0.560-0.969) | 86.3 | 84.4 | 15.9 | 99.4 | 24.045 | <0.001 |
| Non-Obese | TyG | 0.531 (0.280-0.781) | 61.7 | 48.2 | 2.8 | 98.1 | 8.050 | 0.760 |
|  | TyG-BMI | 0.487 (0.243-0.748) | 65.7 | 38.6 | 2.5 | 97.9 | 183.851 | 0.982 |
|  | TyG-WC | 0.545 (0.298-0.793) | 52.5 | 62.9 | 3.3 | 98.2 | 727.849 | 0.558 |
|  | TyG-WHtR | 0.554 (0.257-0.802) | 46.1 | 86.7 | 7.6 | 98.5 | 4.955 | 0.617 |
|  | TyG-WWI | 0.559 (0.289-0.810) | 47.5 | 89.2 | 9.4 | 98.6 | 98.208 | 0.613 |
|  | VAI | 0.580 (0.345-0.810) | 77.7 | 48.4 | 3.5 | 98.9 | 0.813 | 0.720 |
|  | HOMA-IR | 0.569 (0.339-0.794) | 57.4 | 60.3 | 3.3 | 98.3 | 1.103 | 0.932 |
|  | METS-IR | 0.524 (0.290-0.711) | 57.9 | 60.0 | 3.3 | 98.4 | 31.438 | 0.906 |
|  | FSI | 0.597 (0.354-0.851) | 58.6 | 69.2 | 4.3 | 98.6 | -2.241 | 0.376 |
|  | FLI | 0.565 (0.293-0.807) | 59.2 | 60.3 | 3.4 | 98.4 | 18.951 | 0.272 |
|  | USFLI | 0.571 (0.271-0.842) | 56.5 | 67.1 | 3.9 | 98.5 | 9.367 | 0.063 |
|  | ZJU | 0.559 (0.323-0.779) | 57.9 | 56.2 | 3.0 | 98.2 | 32.905 | 0.589 |
|  | LAP | 0.511 (0.240-0.782) | 59.5 | 53.0 | 2.9 | 98.2 | 19.444 | 0.581 |
|  | HSI | 0.577 (0.380-0.755) | 55.4 | 65.7 | 3.7 | 98.4 | 30.000 | 0.427 |
|  | NHHR | 0.652 (0.415-0.821) | 68.5 | 74.6 | 6.0 | 99.0 | 1.471 | 0.330 |
|  | NFS | 0.636 (0.369-0.877) | 57.3 | 78.2 | 5.9 | 98.7 | -1.503 | 0.175 |
|  | FIB-4 | 0.628 (0.365-0.873) | 62.3 | 66.8 | 4.3 | 98.7 | 1.056 | 0.006 |
|  | BARD | 0.576 (0.419-0.770) | 54.7 | 66.9 | 3.8 | 98.4 | 1.529 | 0.431 |
| Obese | TyG | 0.557 (0.415-0.689) | 71.4 | 47.5 | 9.3 | 95.7 | 8.273 | 0.397 |
|  | TyG-BMI | 0.705 (0.550-0.838) | 69.1 | 67.2 | 13.7 | 96.7 | 309.680 | 0.002 |
|  | TyG-WC | 0.797 (0.648-0.906) | 85.6 | 72.1 | 18.7 | 98.5 | 975.376 | <0.001 |
|  | TyG-WHtR | 0.764 (0.601-0.891) | 85.2 | 65.7 | 15.7 | 98.3 | 5.681 | <0.001 |
|  | TyG-WWI | 0.660 (0.513-0.800) | 57.7 | 69.9 | 12.6 | 95.6 | 98.220 | 0.025 |
|  | VAI | 0.571 (0.409-0.701) | 68.4 | 56.5 | 10.6 | 96.0 | 1.411 | 0.566 |
|  | HOMA-IR | 0.533 (0.391-0.688) | 58.1 | 55.4 | 8.9 | 94.6 | 3.200 | 0.395 |
|  | METS-IR | 0.699 (0.555-0.838) | 64.9 | 73.1 | 15.4 | 96.5 | 54.974 | 0.008 |
|  | FSI | 0.799 (0.692-0.885) | 81.8 | 70.3 | 17.1 | 98.1 | -0.070 | 0.002 |
|  | FLI | 0.800 (0.688-0.900) | 78.2 | 75.8 | 19.6 | 97.9 | 91.886 | 0.007 |
|  | USFLI | 0.716 (0.563-0.842) | 67.2 | 76.8 | 17.9 | 96.9 | 42.719 | 0.009 |
|  | ZJU | 0.676 (0.522-0.821) | 78.1 | 53.6 | 11.2 | 97.0 | 45.110 | 0.005 |
|  | LAP | 0.677 (0.511-0.802) | 72.9 | 65.2 | 13.6 | 97.0 | 63.469 | 0.014 |
|  | HSI | 0.671 (0.533-0.793) | 73.8 | 54.3 | 10.8 | 96.5 | 44.247 | 0.008 |
|  | NHHR | 0.544 (0.406-0.681) | 64.1 | 50.5 | 8.9 | 94.9 | 2.736 | 0.611 |
|  | NFS | 0.739 (0.588-0.859) | 70.8 | 73.2 | 16.6 | 97.1 | -1.160 | <0.001 |
|  | FIB-4 | 0.754 (0.628-0.834) | 78.5 | 74.1 | 18.6 | 97.9 | 0.893 | 0.006 |
|  | BARD | 0.554 (0.390-0.705) | 55.7 | 68.3 | 11.7 | 95.3 | 1.857 | 0.531 |
| Non-Hypertension | TyG | 0.516 (0.239-0.832) | 38.2 | 92.2 | 7.6 | 98.9 | 8.969 | 0.997 |
|  | TyG-BMI | 0.538 (0.263-0.848) | 53.6 | 73.8 | 3.3 | 99.0 | 248.446 | 0.538 |
|  | TyG-WC | 0.554 (0.278-0.876) | 43.1 | 92.7 | 9.0 | 99.0 | 975.376 | 0.490 |
|  | TyG-WHtR | 0.526 (0.231-0.886) | 47.8 | 79.1 | 3.7 | 98.9 | 5.219 | 0.581 |
|  | TyG-WWI | 0.499 (0.205-0.866) | 43.6 | 88.6 | 6.0 | 98.9 | 99.187 | 0.752 |
|  | VAI | 0.504 (0.231-0.845) | 47.8 | 76.8 | 3.3 | 98.9 | 1.484 | 0.267 |
|  | HOMA-IR | 0.517 (0.230-0.830) | 43.1 | 78.2 | 3.2 | 98.8 | 2.624 | 0.043 |
|  | METS-IR | 0.575 (0.336-0.853) | 53.6 | 73.0 | 3.2 | 98.9 | 42.267 | 0.352 |
|  | FSI | 0.563 (0.292-0.875) | 53.6 | 70.0 | 2.9 | 98.9 | -1.768 | 0.430 |
|  | FLI | 0.558 (0.277-0.889) | 53.6 | 73.0 | 3.2 | 98.9 | 57.198 | 0.430 |
|  | USFLI | 0.516 (0.240-0.861) | 43.1 | 87.6 | 5.5 | 98.9 | 31.342 | 0.066 |
|  | ZJU | 0.506 (0.216-0.827) | 49.0 | 71.8 | 2.8 | 98.8 | 40.204 | 0.645 |
|  | LAP | 0.513 (0.205-0.846) | 47.8 | 75.9 | 3.2 | 98.9 | 42.772 | 0.134 |
|  | HSI | 0.514 (0.263-0.801) | 49.0 | 69.8 | 2.6 | 98.8 | 37.900 | 0.614 |
|  | NHHR | 0.502 (0.279-0.685) | 48.3 | 63.9 | 2.2 | 98.7 | 1.827 | 0.893 |
|  | NFS | 0.550 (0.279-0.790) | 49.3 | 65.1 | 2.3 | 98.7 | -2.768 | 0.840 |
|  | FIB-4 | 0.558 (0.329-0.750) | 40.7 | 83.8 | 4.0 | 98.8 | 0.442 | 0.683 |
|  | BARD | 0.549 (0.385-0.732) | 61.6 | 55.3 | 2.3 | 98.8 | 1.611 | 0.632 |
| Hypertension | TyG | 0.531 (0.388-0.668) | 82.8 | 42.0 | 15.2 | 95.1 | 8.507 | 0.840 |
|  | TyG-BMI | 0.659 (0.492-0.802) | 61.3 | 65.8 | 18.4 | 93.1 | 255.616 | 0.024 |
|  | TyG-WC | 0.726 (0.575-0.866) | 66.4 | 75.0 | 25.0 | 94.6 | 923.368 | 0.004 |
|  | TyG-WHtR | 0.703 (0.566-0.824) | 55.8 | 80.8 | 26.9 | 93.6 | 5.691 | 0.003 |
|  | TyG-WWI | 0.655 (0.524-0.773) | 78.7 | 53.7 | 17.6 | 95.2 | 93.166 | 0.010 |
|  | VAI | 0.509 (0.363-0.659) | 42.3 | 71.4 | 15.7 | 90.8 | 0.813 | 0.426 |
|  | HOMA-IR | 0.539 (0.403-0.663) | 64.9 | 53.1 | 14.8 | 92.3 | 1.834 | 0.790 |
|  | METS-IR | 0.639 (0.467-0.797) | 67.5 | 61.0 | 17.9 | 93.7 | 42.878 | 0.034 |
|  | FSI | 0.687 (0.521-0.823) | 64.7 | 70.3 | 21.6 | 94.1 | -0.555 | 0.018 |
|  | FLI | 0.743 (0.590-0.869) | 76.7 | 67.6 | 23.0 | 95.8 | 68.374 | 0.034 |
|  | USFLI | 0.741 (0.620-0.839) | 80.2 | 66.0 | 22.9 | 96.4 | 20.389 | 0.005 |
|  | ZJU | 0.647 (0.494-0.793) | 57.3 | 67.3 | 18.1 | 92.6 | 41.144 | 0.028 |
|  | LAP | 0.655 (0.496-0.803) | 57.9 | 76.7 | 23.9 | 93.5 | 58.496 | 0.172 |
|  | HSI | 0.645 (0.493-0.804) | 56.9 | 76.6 | 23.5 | 93.4 | 41.645 | 0.027 |
|  | NHHR | 0.514 (0.325-0.715) | 47.8 | 62.1 | 13.7 | 90.4 | 2.094 | 0.855 |
|  | NFS | 0.765 (0.640-0.876) | 72.6 | 73.0 | 25.3 | 95.5 | -0.827 | <0.001 |
|  | FIB-4 | 0.673 (0.528-0.799) | 84.0 | 49.7 | 17.4 | 96.1 | 1.046 | 0.002 |
|  | BARD | 0.584 (0.446-0.725) | 86.4 | 40.8 | 15.5 | 96.0 | 1.586 | 0.130 |

TyG: triglyceride-glucose index; BMI: body mass index; WC: waist circumference; WHtR: waist-to-height ratio; WWI: weight-adjusted waist index; VAI: visceral adiposity index; HOMA-IR: homeostatic model assessment of insulin resistance; METS-IR: metabolic score for insulin resistance; FSI: Framingham steatosis index; FLI: fatty liver index; ZJU: Zhejiang University index; LAP: lipid accumulation product; HSI: hepatic steatosis index; NHHR: non-high-density lipoprotein cholesterol (HDL-C) to HDL-C ratio; NFS: nonalcoholic fatty liver disease fibrosis score; FIB-4: fibrosis-4 index; BARD: BMI-aspartate aminotransferase/alanine aminotransferase ratio and diabetes score

Note: We were unable to complete the subgroup analyses due to the insufficient positive cases of Mexican American and other race subgroups.

**Table. S9.** Diagnostic efficacy of 18 indices for significant fibrosis in patients with prediabetes across different demographic characteristics

| Group | Variables | AUC (95%CI) | Sensitivity (%) | Specificity (%) | PPV | NPV | Cut-off Values | p-Value |
| --- | --- | --- | --- | --- | --- | --- | --- | --- |
| Female | TyG | 0.501 (0.405-0.602) | 68.5 | 39.0 | 7.4 | 94.5 | 8.323 | 0.800 |
|  | TyG-BMI | 0.815 (0.725-0.889) | 89.6 | 63.7 | 15.0 | 98.9 | 261.901 | <0.001 |
|  | TyG-WC | 0.799 (0.717-0.871) | 84.1 | 68.2 | 15.9 | 98.4 | 892.771 | <0.001 |
|  | TyG-WHtR | 0.789 (0.693-0.870) | 74.7 | 73.6 | 16.8 | 97.6 | 5.751 | <0.001 |
|  | TyG-WWI | 0.612 (0.506-0.728) | 61.0 | 58.6 | 9.5 | 95.5 | 97.905 | 0.050 |
|  | VAI | 0.537 (0.431-0.659) | 55.0 | 50.1 | 7.3 | 94.0 | 1.411 | 0.830 |
|  | HOMA-IR | 0.649 (0.463-0.820) | 61.2 | 71.9 | 13.4 | 96.3 | 3.851 | 0.006 |
|  | METS-IR | 0.795 (0.704-0.875) | 68.7 | 76.0 | 17.0 | 97.1 | 49.896 | <0.001 |
|  | FSI | 0.808 (0.727-0.876) | 88.7 | 66.7 | 16.0 | 98.8 | -0.844 | <0.001 |
|  | FLI | 0.836 (0.746-0.908) | 75.3 | 77.0 | 18.9 | 97.8 | 78.943 | <0.001 |
|  | USFLI | 0.748 (0.597-0.889) | 78.1 | 67.9 | 14.8 | 97.7 | 29.829 | <0.001 |
|  | ZJU | 0.813 (0.719-0.893) | 92.5 | 61.6 | 14.7 | 99.1 | 42.200 | <0.001 |
|  | LAP | 0.694 (0.597-0.789) | 66.1 | 65.4 | 12.0 | 96.4 | 54.573 | 0.018 |
|  | HSI | 0.802 (0.698-0.882) | 74.0 | 73.5 | 16.6 | 97.5 | 43.300 | <0.001 |
|  | NHHR | 0.510 (0.373-0.630) | 50.0 | 63.7 | 9.0 | 94.7 | 2.118 | 0.632 |
|  | NFS | 0.709 (0.611-0.797) | 71.1 | 72.8 | 15.7 | 97.2 | -0.748 | <0.001 |
|  | FIB-4 | 0.515 (0.384-0.640) | 54.1 | 63.2 | 9.5 | 95.1 | 1.118 | 0.038 |
|  | BARD | 0.693 (0.601-0.769) | 74.7 | 66.0 | 13.5 | 97.3 | 1.952 | <0.001 |
| Male | TyG | 0.625 (0.511-0.717) | 56.9 | 66.8 | 10.2 | 95.9 | 8.728 | 0.010 |
|  | TyG-BMI | 0.689 (0.480-0.855) | 59.9 | 84.0 | 19.9 | 96.9 | 292.239 | 0.005 |
|  | TyG-WC | 0.685 (0.481-0.838) | 63.9 | 75.0 | 14.5 | 96.9 | 951.629 | 0.017 |
|  | TyG-WHtR | 0.682 (0.496-0.845) | 64.5 | 77.0 | 15.7 | 97.0 | 5.492 | 0.019 |
|  | TyG-WWI | 0.654 (0.503-0.779) | 66.4 | 62.6 | 10.5 | 96.6 | 95.172 | 0.039 |
|  | VAI | 0.630 (0.507-0.729) | 76.0 | 54.7 | 10.0 | 97.2 | 1.202 | 0.058 |
|  | HOMA-IR | 0.659 (0.494-0.797) | 60.3 | 77.2 | 15.0 | 96.7 | 4.224 | 0.004 |
|  | METS-IR | 0.692 (0.498-0.855) | 61.5 | 83.2 | 19.6 | 97.0 | 51.538 | 0.005 |
|  | FSI | 0.684 (0.500-0.853) | 54.2 | 83.5 | 17.9 | 96.5 | 0.155 | 0.014 |
|  | FLI | 0.701 (0.509-0.854) | 60.5 | 79.1 | 16.1 | 96.8 | 84.968 | 0.123 |
|  | USFLI | 0.685 (0.512-0.829) | 67.3 | 68.5 | 12.4 | 96.9 | 37.252 | 0.007 |
|  | ZJU | 0.682 (0.482-0.861) | 63.7 | 78.2 | 16.3 | 97.0 | 43.618 | 0.008 |
|  | LAP | 0.664 (0.506-0.814) | 62.8 | 75.6 | 14.6 | 96.8 | 58.605 | 0.004 |
|  | HSI | 0.677 (0.469-0.841) | 63.7 | 77.4 | 15.7 | 97.0 | 42.500 | 0.014 |
|  | NHHR | 0.596 (0.462-0.718) | 57.2 | 66.4 | 10.2 | 95.9 | 3.315 | 0.058 |
|  | NFS | 0.675 (0.518-0.814) | 56.3 | 68.9 | 10.7 | 96.0 | -0.836 | 0.045 |
|  | FIB-4 | 0.533 (0.426-0.632) | 69.2 | 46.3 | 7.9 | 95.8 | 0.850 | 0.680 |
|  | BARD | 0.511 (0.422-0.602) | 72.4 | 46.1 | 8.2 | 96.2 | 1.591 | 0.580 |
| Mexican American | TyG | 0.501 (0.363-0.657) | 82.2 | 39.3 | 10.9 | 96.1 | 8.399 | 0.981 |
|  | TyG-BMI | 0.755 (0.609-0.879) | 78.3 | 66.7 | 17.6 | 97.1 | 274.925 | 0.006 |
|  | TyG-WC | 0.705 (0.576-0.837) | 78.3 | 59.9 | 15.0 | 96.8 | 882.535 | 0.001 |
|  | TyG-WHtR | 0.758 (0.639-0.857) | 78.3 | 71.9 | 20.2 | 97.3 | 5.636 | <0.001 |
|  | TyG-WWI | 0.658 (0.526-0.783) | 77.8 | 61.5 | 15.5 | 96.8 | 98.355 | <0.001 |
|  | VAI | 0.465 (0.306-0.629) | 56.3 | 47.0 | 8.8 | 92.2 | 1.626 | 0.661 |
|  | HOMA-IR | 0.647 (0.492-0.796) | 59.5 | 64.6 | 13.2 | 94.6 | 4.372 | 0.196 |
|  | METS-IR | 0.700 (0.534-0.847) | 78.3 | 64.4 | 16.7 | 97.0 | 47.219 | 0.032 |
|  | FSI | 0.687 (0.546-0.825) | 74.6 | 64.5 | 16.0 | 96.5 | -0.820 | 0.046 |
|  | FLI | 0.767 (0.656-0.870) | 84.7 | 68.7 | 19.7 | 98.0 | 77.977 | 0.003 |
|  | USFLI | 0.736 (0.600-0.862) | 65.9 | 77.3 | 20.9 | 96.2 | 56.375 | 0.013 |
|  | ZJU | 0.741 (0.579-0.883) | 68.5 | 74.1 | 19.3 | 96.3 | 45.610 | 0.007 |
|  | LAP | 0.643 (0.515-0.776) | 66.6 | 64.8 | 14.7 | 95.5 | 55.845 | 0.151 |
|  | HSI | 0.692 (0.520-0.857) | 68.5 | 71.6 | 17.9 | 96.2 | 44.300 | 0.029 |
|  | NHHR | 0.581 (0.406-0.761) | 42.3 | 80.9 | 16.7 | 93.9 | 2.047 | 0.349 |
|  | NFS | 0.788 (0.658-0.897) | 69.1 | 77.3 | 21.7 | 96.5 | -0.854 | 0.003 |
|  | FIB-4 | 0.640 (0.436-0.828) | 48.1 | 82.6 | 20.0 | 94.6 | 1.138 | 0.006 |
|  | BARD | 0.682 (0.536-0.818) | 58.7 | 74.2 | 17.1 | 95.2 | 1.936 | 0.041 |
| Non-Hispanic Black | TyG | 0.598 (0.440-0.742) | 80.2 | 40.3 | 11.0 | 95.7 | 8.100 | 0.128 |
|  | TyG-BMI | 0.652 (0.445-0.809) | 61.4 | 78.6 | 20.8 | 95.7 | 318.362 | 0.010 |
|  | TyG-WC | 0.691 (0.517-0.852) | 68.5 | 73.1 | 18.9 | 96.2 | 947.735 | 0.024 |
|  | TyG-WHtR | 0.666 (0.469-0.827) | 61.4 | 78.4 | 20.6 | 95.7 | 5.834 | 0.056 |
|  | TyG-WWI | 0.640 (0.475-0.780) | 67.2 | 66.3 | 15.5 | 95.7 | 93.701 | 0.198 |
|  | VAI | 0.623 (0.469-0.751) | 63.1 | 59.1 | 12.4 | 94.6 | 1.139 | 0.115 |
|  | HOMA-IR | 0.654 (0.483-0.785) | 65.7 | 65.8 | 15.0 | 95.4 | 3.967 | 0.017 |
|  | METS-IR | 0.658 (0.489-0.817) | 59.8 | 80.9 | 22.4 | 95.6 | 57.606 | 0.004 |
|  | FSI | 0.671 (0.446-0.847) | 63.6 | 75.5 | 19.2 | 95.8 | -0.178 | 0.017 |
|  | FLI | 0.691 (0.505-0.845) | 65.0 | 75.3 | 19.4 | 95.9 | 89.311 | 0.102 |
|  | USFLI | 0.714 (0.541-0.845) | 68.5 | 66.9 | 16.0 | 95.9 | 23.042 | 0.001 |
|  | ZJU | 0.626 (0.433-0.789) | 61.4 | 74.5 | 18.1 | 95.5 | 47.966 | 0.014 |
|  | LAP | 0.670 (0.500-0.820) | 66.2 | 68.0 | 15.9 | 95.6 | 45.225 | 0.047 |
|  | HSI | 0.634 (0.437-0.792) | 58.5 | 81.0 | 22.0 | 95.5 | 47.642 | 0.012 |
|  | NHHR | 0.619 (0.474-0.751) | 83.8 | 44.6 | 12.2 | 96.8 | 2.158 | 0.035 |
|  | NFS | 0.634 (0.485-0.779) | 70.6 | 63.8 | 15.2 | 95.9 | -0.748 | 0.122 |
|  | FIB-4 | 0.532 (0.418-0.661) | 54.4 | 59.2 | 10.9 | 93.4 | 1.025 | 0.831 |
|  | BARD | 0.542 (0.394-0.692) | 57.1 | 57.2 | 10.9 | 93.6 | 1.905 | 0.736 |
| Non-Hispanic White | TyG | 0.591 (0.487-0.698) | 49.2 | 65.3 | 9.0 | 94.9 | 8.724 | 0.152 |
|  | TyG-BMI | 0.761 (0.581-0.909) | 71.2 | 75.6 | 16.8 | 97.4 | 276.386 | 0.003 |
|  | TyG-WC | 0.760 (0.604-0.896) | 74.0 | 75.3 | 17.2 | 97.7 | 955.102 | 0.006 |
|  | TyG-WHtR | 0.756 (0.598-0.904) | 75.0 | 71.9 | 15.6 | 97.6 | 5.492 | 0.009 |
|  | TyG-WWI | 0.659 (0.546-0.788) | 60.2 | 65.9 | 10.9 | 96.0 | 98.218 | 0.014 |
|  | VAI | 0.618 (0.504-0.724) | 72.5 | 49.7 | 9.1 | 96.3 | 1.202 | 0.249 |
|  | HOMA-IR | 0.648 (0.477-0.813) | 58.9 | 81.2 | 17.8 | 96.6 | 4.306 | 0.002 |
|  | METS-IR | 0.756 (0.585-0.900) | 68.2 | 83.1 | 21.8 | 97.4 | 51.538 | 0.002 |
|  | FSI | 0.757 (0.589-0.893) | 82.0 | 62.6 | 13.2 | 98.0 | -0.990 | 0.003 |
|  | FLI | 0.774 (0.608-0.918) | 69.8 | 78.1 | 18.1 | 97.4 | 84.159 | 0.037 |
|  | USFLI | 0.707 (0.538-0.879) | 66.6 | 75.3 | 15.7 | 97.0 | 38.056 | 0.003 |
|  | ZJU | 0.758 (0.584-0.906) | 80.1 | 68.2 | 14.9 | 98.0 | 42.200 | 0.004 |
|  | LAP | 0.706 (0.565-0.831) | 70.7 | 68.1 | 13.3 | 97.1 | 54.573 | 0.004 |
|  | HSI | 0.750 (0.567-0.893) | 71.2 | 75.2 | 16.6 | 97.4 | 42.500 | 0.004 |
|  | NHHR | 0.567 (0.426-0.691) | 50.8 | 65.3 | 9.2 | 95.0 | 3.054 | 0.328 |
|  | NFS | 0.680 (0.550-0.813) | 59.7 | 73.7 | 13.6 | 96.3 | -0.628 | 0.025 |
|  | FIB-4 | 0.507 (0.390-0.616) | 44.0 | 58.7 | 6.9 | 93.8 | 0.873 | 0.858 |
|  | BARD | 0.575 (0.456-0.687) | 75.0 | 47.4 | 9.0 | 96.5 | 1.610 | 0.253 |
| Other Race | TyG | 0.486 (0.264-0.741) | 41.1 | 74.3 | 7.6 | 96.1 | 8.086 | 0.976 |
|  | TyG-BMI | 0.778 (0.632-0.892) | 89.5 | 71.7 | 14.0 | 99.3 | 257.724 | <0.001 |
|  | TyG-WC | 0.681 (0.515-0.840) | 54.7 | 74.6 | 9.9 | 97.0 | 887.012 | 0.023 |
|  | TyG-WHtR | 0.659 (0.481-0.838) | 68.4 | 60.9 | 8.2 | 97.4 | 5.106 | 0.056 |
|  | TyG-WWI | 0.467 (0.258-0.688) | 31.6 | 84.5 | 9.4 | 96.0 | 83.651 | 0.893 |
|  | VAI | 0.497 (0.273-0.745) | 41.1 | 70.4 | 6.6 | 95.9 | 0.864 | 0.638 |
|  | HOMA-IR | 0.698 (0.508-0.868) | 72.4 | 64.3 | 9.4 | 97.9 | 3.160 | 0.190 |
|  | METS-IR | 0.786 (0.647-0.884) | 92.7 | 67.9 | 12.9 | 99.5 | 44.575 | 0.003 |
|  | FSI | 0.768 (0.611-0.901) | 79.1 | 65.0 | 10.4 | 98.4 | -1.163 | <0.001 |
|  | FLI | 0.753 (0.609-0.870) | 88.7 | 68.7 | 12.7 | 99.2 | 59.682 | 0.002 |
|  | USFLI | 0.746 (0.595-0.890) | 74.4 | 61.9 | 9.1 | 97.9 | 23.824 | 0.007 |
|  | ZJU | 0.803 (0.666-0.899) | 92.7 | 71.6 | 14.3 | 99.5 | 41.288 | 0.001 |
|  | LAP | 0.585 (0.368-0.785) | 58.9 | 60.8 | 7.1 | 96.7 | 44.227 | 0.129 |
|  | HSI | 0.801 (0.669-0.904) | 92.7 | 71.6 | 14.3 | 99.5 | 39.547 | 0.004 |
|  | NHHR | 0.491 (0.224-0.736) | 48.6 | 61.2 | 6.0 | 95.9 | 3.193 | 0.806 |
|  | NFS | 0.596 (0.409-0.833) | 48.6 | 75.5 | 9.2 | 96.6 | -1.237 | 0.347 |
|  | FIB-4 | 0.447 (0.274-0.665) | 30.3 | 78.6 | 6.8 | 95.7 | 1.272 | 0.589 |
|  | BARD | 0.700 (0.589-0.801) | 85.3 | 60.7 | 10.0 | 98.8 | 1.629 | 0.004 |
| Age ≤50 | TyG | 0.599 (0.492-0.693) | 56.7 | 60.9 | 10.6 | 94.5 | 8.652 | 0.088 |
|  | TyG-BMI | 0.820 (0.664-0.918) | 80.3 | 79.1 | 23.8 | 98.0 | 292.239 | <0.001 |
|  | TyG-WC | 0.797 (0.648-0.903) | 78.4 | 77.7 | 22.3 | 97.8 | 955.102 | <0.001 |
|  | TyG-WHtR | 0.809 (0.660-0.905) | 80.3 | 74.7 | 20.5 | 97.9 | 5.492 | <0.001 |
|  | TyG-WWI | 0.721 (0.587-0.823) | 63.6 | 75.1 | 17.2 | 96.2 | 98.456 | 0.005 |
|  | VAI | 0.601 (0.471-0.700) | 67.6 | 56.0 | 11.1 | 95.5 | 1.411 | 0.165 |
|  | HOMA-IR | 0.736 (0.629-0.835) | 74.0 | 73.5 | 18.5 | 97.2 | 4.306 | 0.002 |
|  | METS-IR | 0.808 (0.646-0.909) | 80.3 | 78.0 | 22.9 | 98.0 | 51.538 | <0.001 |
|  | FSI | 0.812 (0.668-0.911) | 69.6 | 83.8 | 25.9 | 97.1 | 0.211 | <0.001 |
|  | FLI | 0.826 (0.688-0.921) | 80.2 | 78.1 | 22.9 | 98.0 | 82.610 | 0.011 |
|  | USFLI | 0.801 (0.671-0.890) | 74.7 | 80.0 | 23.3 | 97.5 | 43.309 | <0.001 |
|  | ZJU | 0.816 (0.666-0.912) | 84.4 | 71.4 | 19.3 | 98.3 | 43.618 | <0.001 |
|  | LAP | 0.733 (0.598-0.841) | 74.5 | 74.0 | 18.9 | 97.3 | 58.605 | <0.001 |
|  | HSI | 0.808 (0.658-0.907) | 83.4 | 68.7 | 17.8 | 98.1 | 42.500 | <0.001 |
|  | NHHR | 0.583 (0.427-0.693) | 63.0 | 61.6 | 11.8 | 95.3 | 3.084 | 0.228 |
|  | NFS | 0.771 (0.632-0.877) | 80.5 | 63.8 | 15.3 | 97.6 | -1.940 | <0.001 |
|  | FIB-4 | 0.575 (0.415-0.709) | 63.9 | 54.7 | 10.3 | 94.9 | 0.656 | 0.038 |
|  | BARD | 0.600 (0.504-0.703) | 78.8 | 49.2 | 11.2 | 96.6 | 1.610 | 0.042 |
| Age >50 | TyG | 0.544 (0.435-0.675) | 62.0 | 45.4 | 6.1 | 95.4 | 8.466 | 0.549 |
|  | TyG-BMI | 0.660 (0.472-0.815) | 68.8 | 66.7 | 10.6 | 97.4 | 261.901 | 0.068 |
|  | TyG-WC | 0.651 (0.479-0.798) | 71.5 | 61.1 | 9.5 | 97.4 | 887.012 | 0.079 |
|  | TyG-WHtR | 0.635 (0.441-0.800) | 57.3 | 72.0 | 10.5 | 96.7 | 5.582 | 0.138 |
|  | TyG-WWI | 0.549 (0.432-0.668) | 53.2 | 58.2 | 6.8 | 95.6 | 97.823 | 0.441 |
|  | VAI | 0.580 (0.486-0.689) | 88.1 | 39.2 | 7.7 | 98.3 | 1.005 | 0.839 |
|  | HOMA-IR | 0.538 (0.375-0.745) | 44.0 | 74.4 | 9.0 | 95.9 | 3.851 | 0.187 |
|  | METS-IR | 0.662 (0.498-0.818) | 74.8 | 58.1 | 9.3 | 97.6 | 42.461 | 0.056 |
|  | FSI | 0.646 (0.472-0.793) | 70.3 | 63.9 | 10.0 | 97.4 | -0.844 | 0.089 |
|  | FLI | 0.673 (0.512-0.835) | 77.8 | 55.6 | 9.1 | 97.8 | 60.233 | 0.121 |
|  | USFLI | 0.591 (0.407-0.794) | 63.3 | 59.1 | 8.1 | 96.6 | 29.829 | 0.116 |
|  | ZJU | 0.658 (0.469-0.813) | 67.9 | 68.5 | 11.0 | 97.4 | 42.200 | 0.099 |
|  | LAP | 0.607 (0.469-0.740) | 69.4 | 53.0 | 7.8 | 96.8 | 42.084 | 0.136 |
|  | HSI | 0.645 (0.447-0.814) | 66.8 | 62.8 | 9.3 | 97.1 | 39.133 | 0.112 |
|  | NHHR | 0.509 (0.383-0.633) | 61.0 | 49.3 | 6.4 | 95.7 | 2.546 | 0.758 |
|  | NFS | 0.631 (0.502-0.760) | 67.2 | 57.9 | 8.4 | 96.9 | -0.628 | 0.072 |
|  | FIB-4 | 0.545 (0.466-0.639) | 80.8 | 42.5 | 7.4 | 97.5 | 1.153 | 0.061 |
|  | BARD | 0.607 (0.454-0.742) | 49.6 | 71.7 | 9.1 | 96.1 | 1.952 | 0.174 |
| Never-smoker | TyG | 0.598 (0.494-0.694) | 71.3 | 47.6 | 8.7 | 96.0 | 8.486 | 0.035 |
|  | TyG-BMI | 0.708 (0.535-0.865) | 67.9 | 75.4 | 16.1 | 97.1 | 281.969 | 0.005 |
|  | TyG-WC | 0.710 (0.554-0.853) | 63.5 | 78.7 | 17.2 | 96.9 | 955.102 | 0.009 |
|  | TyG-WHtR | 0.693 (0.532-0.838) | 69.3 | 69.5 | 13.6 | 97.0 | 5.492 | 0.011 |
|  | TyG-WWI | 0.632 (0.510-0.754) | 51.8 | 75.6 | 12.9 | 95.7 | 100.873 | 0.011 |
|  | VAI | 0.612 (0.492-0.718) | 48.7 | 76.9 | 12.8 | 95.6 | 2.299 | 0.079 |
|  | HOMA-IR | 0.700 (0.542-0.829) | 63.6 | 77.5 | 16.5 | 96.8 | 4.406 | <0.001 |
|  | METS-IR | 0.714 (0.554-0.870) | 69.9 | 73.3 | 15.4 | 97.2 | 48.430 | 0.005 |
|  | FSI | 0.708 (0.554-0.857) | 67.1 | 72.1 | 14.4 | 96.9 | -0.489 | 0.007 |
|  | FLI | 0.731 (0.566-0.885) | 67.9 | 76.3 | 16.6 | 97.2 | 78.943 | 0.042 |
|  | USFLI | 0.737 (0.579-0.876) | 68.5 | 75.5 | 16.3 | 97.2 | 38.056 | <0.001 |
|  | ZJU | 0.703 (0.529-0.863) | 73.2 | 72.1 | 15.4 | 97.5 | 43.618 | 0.010 |
|  | LAP | 0.661 (0.518-0.802) | 59.7 | 71.7 | 12.8 | 96.2 | 59.057 | 0.006 |
|  | HSI | 0.703 (0.530-0.858) | 72.3 | 72.3 | 15.4 | 97.4 | 42.500 | 0.008 |
|  | NHHR | 0.569 (0.455-0.686) | 44.1 | 73.6 | 10.4 | 95.0 | 3.454 | 0.101 |
|  | NFS | 0.633 (0.519-0.746) | 56.1 | 69.5 | 11.4 | 95.8 | -0.854 | 0.013 |
|  | FIB-4 | 0.507 (0.385-0.612) | 51.3 | 64.7 | 9.2 | 95.0 | 1.118 | 0.454 |
|  | BARD | 0.515 (0.418-0.613) | 77.9 | 41.0 | 8.4 | 96.4 | 1.551 | 0.623 |
| Former smoker | TyG | 0.615 (0.497-0.722) | 62.5 | 65.9 | 9.1 | 97.0 | 8.680 | 0.244 |
|  | TyG-BMI | 0.880 (0.711-0.946) | 77.2 | 87.8 | 25.7 | 98.6 | 311.083 | <0.001 |
|  | TyG-WC | 0.858 (0.710-0.938) | 82.8 | 78.9 | 17.7 | 98.8 | 978.795 | <0.001 |
|  | TyG-WHtR | 0.874 (0.715-0.945) | 89.2 | 75.9 | 16.9 | 99.2 | 5.582 | <0.001 |
|  | TyG-WWI | 0.740 (0.618-0.830) | 86.1 | 65.9 | 12.1 | 98.9 | 97.823 | 0.003 |
|  | VAI | 0.640 (0.486-0.753) | 56.6 | 73.6 | 10.5 | 96.9 | 1.758 | 0.846 |
|  | HOMA-IR | 0.687 (0.520-0.826) | 54.8 | 80.0 | 13.1 | 97.0 | 4.306 | 0.069 |
|  | METS-IR | 0.872 (0.712-0.940) | 80.8 | 86.9 | 25.2 | 98.8 | 53.516 | <0.001 |
|  | FSI | 0.853 (0.706-0.934) | 74.0 | 83.4 | 19.7 | 98.3 | 0.155 | <0.001 |
|  | FLI | 0.878 (0.724-0.950) | 74.0 | 90.3 | 29.5 | 98.4 | 92.458 | 0.020 |
|  | USFLI | 0.790 (0.600-0.910) | 61.0 | 86.0 | 19.2 | 97.6 | 50.546 | 0.015 |
|  | ZJU | 0.877 (0.680-0.947) | 80.8 | 83.0 | 20.7 | 98.8 | 45.553 | <0.001 |
|  | LAP | 0.786 (0.639-0.877) | 86.2 | 70.6 | 13.8 | 98.9 | 54.573 | 0.030 |
|  | HSI | 0.865 (0.680-0.940) | 80.8 | 80.9 | 18.8 | 98.7 | 44.257 | <0.001 |
|  | NHHR | 0.599 (0.391-0.761) | 55.7 | 69.8 | 9.2 | 96.6 | 3.147 | 0.483 |
|  | NFS | 0.842 (0.707-0.923) | 83.6 | 76.8 | 16.5 | 98.8 | -0.302 | 0.003 |
|  | FIB-4 | 0.549 (0.426-0.701) | 90.0 | 40.1 | 7.6 | 98.7 | 0.850 | 0.253 |
|  | BARD | 0.662 (0.544-0.789) | 86.1 | 57.5 | 10.0 | 98.7 | 1.700 | 0.013 |
| Current smoker | TyG | 0.621 (0.447-0.749) | 81.9 | 56.5 | 16.5 | 96.7 | 8.484 | 0.039 |
|  | TyG-BMI | 0.712 (0.472-0.863) | 75.2 | 67.9 | 19.7 | 96.3 | 259.675 | 0.037 |
|  | TyG-WC | 0.639 (0.372-0.796) | 77.6 | 67.0 | 19.8 | 96.6 | 892.771 | 0.241 |
|  | TyG-WHtR | 0.666 (0.433-0.810) | 84.1 | 61.9 | 18.8 | 97.4 | 5.261 | 0.101 |
|  | TyG-WWI | 0.536 (0.329-0.710) | 72.9 | 46.0 | 12.4 | 94.2 | 94.054 | 0.332 |
|  | VAI | 0.581 (0.400-0.717) | 79.4 | 53.6 | 15.2 | 96.1 | 1.411 | 0.090 |
|  | HOMA-IR | 0.570 (0.230-0.859) | 42.3 | 99.1 | 83.4 | 94.3 | 0.825 | 0.301 |
|  | METS-IR | 0.660 (0.404-0.841) | 78.8 | 59.7 | 17.0 | 96.4 | 42.714 | 0.080 |
|  | FSI | 0.665 (0.390-0.821) | 71.4 | 69.4 | 19.7 | 95.9 | -0.844 | 0.091 |
|  | FLI | 0.678 (0.471-0.856) | 84.1 | 60.3 | 18.2 | 97.3 | 61.457 | 0.050 |
|  | USFLI | 0.492 (0.225-0.791) | 49.4 | 75.4 | 17.4 | 93.4 | 39.340 | 0.611 |
|  | ZJU | 0.717 (0.468-0.857) | 75.8 | 71.5 | 21.8 | 96.6 | 42.295 | 0.024 |
|  | LAP | 0.433 (0.266-0.626) | 54.6 | 49.6 | 10.2 | 91.3 | 42.084 | 0.839 |
|  | HSI | 0.663 (0.420-0.829) | 75.8 | 61.4 | 17.1 | 96.0 | 39.133 | 0.038 |
|  | NHHR | 0.675 (0.440-0.869) | 54.6 | 81.5 | 23.6 | 94.5 | 1.804 | 0.008 |
|  | NFS | 0.654 (0.472-0.796) | 91.4 | 48.1 | 15.6 | 98.2 | -1.565 | 0.017 |
|  | FIB-4 | 0.543 (0.235-0.802) | 48.8 | 79.6 | 20.1 | 93.7 | 1.263 | 0.301 |
|  | BARD | 0.782 (0.525-0.904) | 75.8 | 78.6 | 27.0 | 96.9 | 2.000 | 0.022 |
| Non-Obese | TyG | 0.577 (0.475-0.705) | 68.2 | 56.2 | 3.9 | 98.5 | 8.466 | 0.232 |
|  | TyG-BMI | 0.577 (0.306-0.731) | 55.1 | 69.1 | 4.4 | 98.3 | 200.018 | 0.506 |
|  | TyG-WC | 0.559 (0.327-0.703) | 55.1 | 73.2 | 5.1 | 98.4 | 706.911 | 0.591 |
|  | TyG-WHtR | 0.585 (0.344-0.718) | 55.1 | 80.6 | 6.9 | 98.6 | 4.089 | 0.474 |
|  | TyG-WWI | 0.531 (0.375-0.654) | 69.6 | 53.6 | 3.8 | 98.5 | 89.424 | 0.808 |
|  | VAI | 0.526 (0.364-0.685) | 59.6 | 59.8 | 3.7 | 98.3 | 1.208 | 0.981 |
|  | HOMA-IR | 0.558 (0.356-0.698) | 53.0 | 69.4 | 4.3 | 98.3 | 1.668 | 0.816 |
|  | METS-IR | 0.574 (0.328-0.711) | 51.1 | 80.8 | 6.5 | 98.5 | 31.874 | 0.437 |
|  | FSI | 0.557 (0.320-0.697) | 50.0 | 77.1 | 5.4 | 98.3 | -2.881 | 0.779 |
|  | FLI | 0.493 (0.312-0.770) | 39.7 | 79.7 | 4.8 | 98.1 | 52.199 | 0.713 |
|  | USFLI | 0.495 (0.322-0.747) | 40.2 | 73.0 | 3.7 | 97.9 | 23.292 | 0.391 |
|  | ZJU | 0.627 (0.396-0.784) | 50.0 | 88.3 | 10.0 | 98.5 | 32.293 | 0.188 |
|  | LAP | 0.542 (0.315-0.678) | 55.1 | 67.7 | 4.2 | 98.3 | 19.837 | 0.894 |
|  | HSI | 0.646 (0.437-0.786) | 51.6 | 82.1 | 7.0 | 98.5 | 30.392 | 0.127 |
|  | NHHR | 0.497 (0.376-0.633) | 51.7 | 54.1 | 2.8 | 97.7 | 2.509 | 0.955 |
|  | NFS | 0.617 (0.522-0.764) | 86.8 | 47.6 | 4.1 | 99.3 | -1.973 | 0.024 |
|  | FIB-4 | 0.687 (0.571-0.790) | 78.1 | 65.2 | 5.5 | 99.1 | 1.153 | <0.001 |
|  | BARD | 0.533 (0.386-0.635) | 60.0 | 57.7 | 3.6 | 98.2 | 1.214 | 0.518 |
| Obese | TyG | 0.499 (0.398-0.600) | 65.1 | 46.3 | 14.2 | 90.7 | 8.731 | 0.967 |
|  | TyG-BMI | 0.737 (0.609-0.838) | 67.1 | 73.5 | 25.7 | 94.2 | 324.893 | <0.001 |
|  | TyG-WC | 0.706 (0.591-0.804) | 74.5 | 57.8 | 19.5 | 94.3 | 1000.316 | 0.002 |
|  | TyG-WHtR | 0.720 (0.604-0.819) | 68.2 | 71.9 | 24.9 | 94.3 | 6.169 | 0.002 |
|  | TyG-WWI | 0.584 (0.473-0.677) | 57.9 | 60.0 | 16.5 | 91.2 | 100.873 | 0.125 |
|  | VAI | 0.481 (0.376-0.585) | 57.4 | 46.6 | 12.8 | 88.9 | 1.774 | 0.614 |
|  | HOMA-IR | 0.617 (0.487-0.737) | 73.3 | 56.7 | 18.8 | 93.9 | 4.306 | 0.049 |
|  | METS-IR | 0.722 (0.589-0.824) | 70.2 | 64.7 | 21.4 | 94.1 | 54.781 | <0.001 |
|  | FSI | 0.710 (0.597-0.807) | 60.8 | 78.3 | 27.7 | 93.6 | 0.915 | 0.001 |
|  | FLI | 0.742 (0.604-0.845) | 74.5 | 70.0 | 25.4 | 95.3 | 92.372 | 0.020 |
|  | USFLI | 0.675 (0.539-0.794) | 60.8 | 72.2 | 23.0 | 93.1 | 52.038 | 0.021 |
|  | ZJU | 0.746 (0.617-0.845) | 63.7 | 82.7 | 33.5 | 94.3 | 50.312 | <0.001 |
|  | LAP | 0.624 (0.510-0.710) | 56.7 | 64.2 | 17.8 | 91.6 | 74.902 | 0.131 |
|  | HSI | 0.738 (0.592-0.846) | 64.6 | 78.5 | 29.1 | 94.2 | 48.640 | <0.001 |
|  | NHHR | 0.508 (0.395-0.633) | 35.0 | 78.1 | 18.0 | 89.8 | 2.176 | 0.788 |
|  | NFS | 0.644 (0.529-0.746) | 70.2 | 61.3 | 19.9 | 93.8 | -0.628 | 0.043 |
|  | FIB-4 | 0.495 (0.386-0.601) | 43.2 | 62.0 | 13.5 | 88.8 | 1.025 | 0.835 |
|  | BARD | 0.541 (0.428-0.648) | 47.1 | 66.4 | 16.1 | 90.2 | 1.806 | 0.586 |
| Non-Hypertension | TyG | 0.552 (0.447-0.650) | 65.1 | 49.4 | 7.3 | 95.9 | 8.434 | 0.403 |
|  | TyG-BMI | 0.718 (0.520-0.879) | 70.6 | 74.9 | 14.6 | 97.7 | 272.953 | 0.006 |
|  | TyG-WC | 0.694 (0.518-0.837) | 66.1 | 76.3 | 14.5 | 97.4 | 928.608 | 0.012 |
|  | TyG-WHtR | 0.705 (0.523-0.851) | 70.5 | 76.6 | 15.6 | 97.7 | 5.492 | 0.018 |
|  | TyG-WWI | 0.614 (0.477-0.739) | 62.9 | 59.9 | 8.7 | 96.4 | 94.557 | 0.134 |
|  | VAI | 0.445 (0.342-0.563) | 48.5 | 48.3 | 5.4 | 93.9 | 1.208 | 0.893 |
|  | HOMA-IR | 0.654 (0.510-0.787) | 58.1 | 80.9 | 15.7 | 96.9 | 4.306 | 0.002 |
|  | METS-IR | 0.712 (0.525-0.864) | 68.8 | 76.8 | 15.3 | 97.6 | 48.430 | 0.007 |
|  | FSI | 0.714 (0.540-0.862) | 70.6 | 68.3 | 12.0 | 97.4 | -1.163 | 0.006 |
|  | FLI | 0.725 (0.518-0.885) | 68.5 | 77.3 | 15.5 | 97.6 | 76.647 | 0.034 |
|  | USFLI | 0.710 (0.540-0.850) | 71.1 | 69.7 | 12.5 | 97.5 | 29.829 | <0.001 |
|  | ZJU | 0.712 (0.499-0.874) | 73.1 | 76.0 | 15.7 | 97.9 | 43.080 | 0.008 |
|  | LAP | 0.642 (0.477-0.783) | 61.0 | 73.8 | 12.5 | 96.9 | 54.573 | 0.098 |
|  | HSI | 0.707 (0.520-0.871) | 68.2 | 77.4 | 15.5 | 97.6 | 42.500 | 0.009 |
|  | NHHR | 0.548 (0.419-0.675) | 46.0 | 66.1 | 7.6 | 95.2 | 3.084 | 0.470 |
|  | NFS | 0.666 (0.525-0.790) | 87.7 | 47.4 | 9.2 | 98.4 | -1.986 | 0.034 |
|  | FIB-4 | 0.531 (0.391-0.649) | 62.8 | 51.2 | 7.3 | 95.8 | 0.850 | 0.180 |
|  | BARD | 0.588 (0.487-0.699) | 68.0 | 56.4 | 8.7 | 96.6 | 1.700 | 0.170 |
| Hypertension | TyG | 0.598 (0.488-0.727) | 61.7 | 56.1 | 10.2 | 94.8 | 8.652 | 0.105 |
|  | TyG-BMI | 0.780 (0.671-0.883) | 63.3 | 82.7 | 22.8 | 96.5 | 311.312 | <0.001 |
|  | TyG-WC | 0.781 (0.673-0.884) | 65.5 | 85.0 | 26.1 | 96.8 | 1036.124 | <0.001 |
|  | TyG-WHtR | 0.764 (0.634-0.876) | 64.5 | 84.8 | 25.5 | 96.7 | 6.169 | <0.001 |
|  | TyG-WWI | 0.666 (0.551-0.771) | 62.6 | 67.8 | 13.6 | 95.8 | 100.873 | 0.001 |
|  | VAI | 0.642 (0.539-0.744) | 62.6 | 63.9 | 12.3 | 95.5 | 1.773 | 0.058 |
|  | HOMA-IR | 0.667 (0.485-0.823) | 55.9 | 88.6 | 28.3 | 96.1 | 6.346 | 0.016 |
|  | METS-IR | 0.772 (0.645-0.883) | 62.9 | 83.3 | 23.2 | 96.5 | 55.007 | <0.001 |
|  | FSI | 0.768 (0.639-0.875) | 67.7 | 73.8 | 17.2 | 96.6 | 0.211 | <0.001 |
|  | FLI | 0.797 (0.688-0.897) | 66.4 | 82.5 | 23.4 | 96.8 | 89.311 | 0.001 |
|  | USFLI | 0.719 (0.533-0.863) | 63.1 | 84.0 | 24.1 | 96.6 | 50.716 | 0.003 |
|  | ZJU | 0.778 (0.655-0.874) | 63.3 | 84.0 | 24.2 | 96.6 | 48.272 | <0.001 |
|  | LAP | 0.730 (0.611-0.834) | 67.9 | 66.1 | 13.9 | 96.2 | 58.605 | <0.001 |
|  | HSI | 0.762 (0.636-0.865) | 59.1 | 86.9 | 26.6 | 96.3 | 47.367 | <0.001 |
|  | NHHR | 0.542 (0.395-0.691) | 47.4 | 71.3 | 11.7 | 94.4 | 3.315 | 0.580 |
|  | NFS | 0.710 (0.618-0.791) | 77.7 | 66.0 | 15.5 | 97.3 | -0.628 | <0.001 |
|  | FIB-4 | 0.482 (0.377-0.585) | 54.8 | 54.9 | 8.9 | 93.8 | 1.191 | 0.764 |
|  | BARD | 0.600 (0.479-0.720) | 47.2 | 71.2 | 11.6 | 94.4 | 1.960 | 0.072 |

TyG: triglyceride-glucose index; BMI: body mass index; WC: waist circumference; WHtR: waist-to-height ratio; WWI: weight-adjusted waist index; VAI: visceral adiposity index; HOMA-IR: homeostatic model assessment of insulin resistance; METS-IR: metabolic score for insulin resistance; FSI: Framingham steatosis index; FLI: fatty liver index; ZJU: Zhejiang University index; LAP: lipid accumulation product; HSI: hepatic steatosis index; NHHR: non-high-density lipoprotein cholesterol (HDL-C) to HDL-C ratio; NFS: nonalcoholic fatty liver disease fibrosis score; FIB-4: fibrosis-4 index; BARD: BMI-aspartate aminotransferase/alanine aminotransferase ratio and diabetes score

Note: We were unable to complete the subgroup analyses due to the insufficient positive cases of other Hispanic subgroups.

**Table. S10**. Diagnostic efficacy of 18 indices for significant fibrosis in patients with T2DM across different demographic characteristics

| Group | Variables | AUC (95%CI) | Sensitivity (%) | Specificity (%) | PPV | NPV | Cut-off Values | p-Value |
| --- | --- | --- | --- | --- | --- | --- | --- | --- |
| Female | TyG | 0.571 (0.480-0.660) | 68.9 | 53.2 | 28.8 | 86.2 | 9.020 | 0.079 |
|  | TyG-BMI | 0.692 (0.596-0.786) | 62.9 | 68.8 | 35.6 | 87.1 | 333.395 | <0.001 |
|  | TyG-WC | 0.708 (0.617-0.793) | 64.5 | 70.3 | 37.3 | 87.8 | 1087.053 | <0.001 |
|  | TyG-WHtR | 0.727 (0.640-0.813) | 74.7 | 63.6 | 36.0 | 90.2 | 6.589 | <0.001 |
|  | TyG-WWI | 0.699 (0.603-0.782) | 68.7 | 63.2 | 33.8 | 88.0 | 109.071 | 0.002 |
|  | VAI | 0.518 (0.415-0.627) | 84.8 | 34.2 | 26.1 | 89.1 | 1.678 | 0.190 |
|  | HOMA-IR | 0.685 (0.576-0.805) | 60.5 | 75.3 | 40.1 | 87.4 | 6.993 | 0.040 |
|  | METS-IR | 0.680 (0.584-0.771) | 70.0 | 63.5 | 34.5 | 88.5 | 56.060 | 0.001 |
|  | FSI | 0.675 (0.583-0.764) | 72.6 | 59.4 | 32.9 | 88.8 | 0.223 | <0.001 |
|  | FLI | 0.726 (0.644-0.807) | 71.2 | 67.3 | 37.4 | 89.5 | 90.755 | <0.001 |
|  | USFLI | 0.734 (0.627-0.833) | 62.6 | 72.4 | 38.4 | 87.6 | 55.163 | 0.001 |
|  | ZJU | 0.699 (0.601-0.787) | 64.4 | 68.1 | 35.6 | 87.4 | 50.501 | <0.001 |
|  | LAP | 0.614 (0.514-0.713) | 76.8 | 48.4 | 29.0 | 88.4 | 69.443 | 0.023 |
|  | HSI | 0.691 (0.600-0.780) | 67.2 | 65.0 | 34.5 | 87.9 | 47.800 | 0.001 |
|  | NHHR | 0.519 (0.391-0.630) | 55.5 | 55.6 | 25.5 | 82.0 | 2.568 | 0.650 |
|  | NFS | 0.695 (0.605-0.784) | 67.8 | 62.7 | 33.2 | 87.7 | 0.370 | <0.001 |
|  | FIB-4 | 0.633 (0.551-0.723) | 78.6 | 52.5 | 31.2 | 90.0 | 1.001 | <0.001 |
|  | BARD | 0.537 (0.434-0.634) | 68.8 | 51.0 | 27.8 | 85.6 | 2.769 | 0.265 |
| Male | TyG | 0.564 (0.472-0.652) | 63.4 | 49.6 | 29.7 | 80.1 | 9.060 | 0.381 |
|  | TyG-BMI | 0.762 (0.684-0.830) | 71.4 | 69.8 | 44.3 | 87.9 | 302.218 | <0.001 |
|  | TyG-WC | 0.749 (0.665-0.823) | 78.6 | 58.2 | 38.7 | 89.0 | 1023.134 | <0.001 |
|  | TyG-WHtR | 0.734 (0.650-0.816) | 67.8 | 73.0 | 45.7 | 87.1 | 6.127 | <0.001 |
|  | TyG-WWI | 0.620 (0.520-0.708) | 59.3 | 66.9 | 37.5 | 83.0 | 107.951 | 0.011 |
|  | VAI | 0.553 (0.465-0.640) | 66.4 | 47.5 | 29.8 | 80.8 | 1.714 | 0.243 |
|  | HOMA-IR | 0.686 (0.593-0.767) | 72.4 | 63.3 | 39.9 | 87.2 | 6.278 | 0.004 |
|  | METS-IR | 0.748 (0.668-0.820) | 64.5 | 73.4 | 44.9 | 86.1 | 55.495 | <0.001 |
|  | FSI | 0.741 (0.656-0.816) | 70.6 | 72.1 | 46.0 | 88.0 | 0.277 | 0.002 |
|  | FLI | 0.791 (0.716-0.860) | 71.5 | 75.1 | 49.1 | 88.7 | 90.429 | <0.001 |
|  | USFLI | 0.758 (0.662-0.834) | 72.0 | 75.7 | 49.8 | 89.0 | 63.918 | <0.001 |
|  | ZJU | 0.738 (0.656-0.820) | 60.6 | 79.0 | 49.2 | 85.7 | 49.101 | <0.001 |
|  | LAP | 0.653 (0.569-0.726) | 69.1 | 58.9 | 36.1 | 85.0 | 66.319 | 0.088 |
|  | HSI | 0.749 (0.664-0.824) | 66.6 | 76.1 | 48.3 | 87.2 | 43.960 | <0.001 |
|  | NHHR | 0.512 (0.423-0.611) | 59.4 | 49.4 | 28.3 | 78.4 | 2.775 | 0.929 |
|  | NFS | 0.560 (0.465-0.665) | 56.3 | 57.4 | 30.7 | 79.7 | 0.160 | 0.139 |
|  | FIB-4 | 0.496 (0.382-0.598) | 43.0 | 66.5 | 30.1 | 77.6 | 1.452 | 0.329 |
|  | BARD | 0.676 (0.594-0.751) | 80.2 | 63.6 | 42.5 | 90.6 | 2.576 | <0.001 |
| Mexican American | TyG | 0.614 (0.427-0.769) | 61.4 | 74.9 | 40.8 | 87.3 | 9.695 | 0.605 |
|  | TyG-BMI | 0.655 (0.505-0.808) | 72.7 | 60.3 | 34.0 | 88.7 | 317.755 | 0.037 |
|  | TyG-WC | 0.727 (0.585-0.865) | 79.2 | 67.4 | 40.6 | 92.0 | 1026.932 | 0.027 |
|  | TyG-WHtR | 0.672 (0.524-0.845) | 88.4 | 53.1 | 34.7 | 94.2 | 6.155 | 0.041 |
|  | TyG-WWI | 0.647 (0.500-0.820) | 80.4 | 53.0 | 32.5 | 90.6 | 107.973 | 0.208 |
|  | VAI | 0.500 (0.351-0.666) | 70.8 | 45.9 | 26.9 | 84.8 | 2.219 | 0.415 |
|  | HOMA-IR | 0.742 (0.530-0.879) | 75.3 | 67.4 | 39.4 | 90.7 | 7.739 | 0.002 |
|  | METS-IR | 0.644 (0.493-0.789) | 74.8 | 62.3 | 35.8 | 89.8 | 55.495 | 0.098 |
|  | FSI | 0.667 (0.460-0.808) | 64.5 | 75.2 | 42.3 | 88.3 | 0.670 | 0.398 |
|  | FLI | 0.698 (0.537-0.878) | 94.7 | 47.7 | 33.8 | 97.0 | 77.328 | 0.010 |
|  | USFLI | 0.849 (0.703-0.936) | 75.6 | 74.9 | 45.9 | 91.6 | 70.548 | 0.003 |
|  | ZJU | 0.626 (0.459-0.771) | 68.7 | 63.7 | 34.8 | 87.8 | 50.694 | 0.242 |
|  | LAP | 0.560 (0.407-0.725) | 81.3 | 43.3 | 28.8 | 89.2 | 61.817 | 0.949 |
|  | HSI | 0.543 (0.386-0.697) | 74.5 | 52.6 | 30.7 | 88.0 | 46.192 | 0.485 |
|  | NHHR | 0.641 (0.456-0.787) | 55.1 | 77.5 | 40.9 | 86.0 | 2.087 | 0.156 |
|  | NFS | 0.646 (0.394-0.926) | 62.6 | 72.1 | 38.8 | 87.3 | 0.338 | 0.302 |
|  | FIB-4 | 0.595 (0.364-0.883) | 59.8 | 61.7 | 30.6 | 84.5 | 1.178 | 0.095 |
|  | BARD | 0.696 (0.573-0.824) | 87.1 | 60.0 | 38.0 | 94.3 | 2.684 | 0.029 |
| Non-Hispanic Black | TyG | 0.582 (0.454-0.705) | 45.2 | 68.9 | 23.5 | 85.6 | 8.963 | 0.238 |
|  | TyG-BMI | 0.669 (0.566-0.785) | 57.7 | 70.1 | 29.0 | 88.7 | 322.991 | 0.008 |
|  | TyG-WC | 0.712 (0.592-0.824) | 56.5 | 75.4 | 32.7 | 89.1 | 1088.282 | 0.006 |
|  | TyG-WHtR | 0.701 (0.593-0.805) | 79.7 | 60.3 | 29.9 | 93.4 | 5.886 | 0.003 |
|  | TyG-WWI | 0.685 (0.573-0.779) | 67.2 | 67.4 | 30.4 | 90.7 | 103.529 | 0.013 |
|  | VAI | 0.603 (0.492-0.719) | 69.8 | 52.2 | 23.6 | 89.1 | 1.226 | 0.282 |
|  | HOMA-IR | 0.627 (0.516-0.736) | 66.7 | 64.7 | 28.6 | 90.2 | 5.824 | 0.279 |
|  | METS-IR | 0.665 (0.551-0.771) | 60.5 | 65.4 | 27.0 | 88.7 | 53.933 | 0.008 |
|  | FSI | 0.690 (0.582-0.796) | 64.5 | 67.0 | 29.2 | 89.9 | 0.410 | 0.004 |
|  | FLI | 0.703 (0.601-0.801) | 60.3 | 69.7 | 29.6 | 89.3 | 91.777 | 0.006 |
|  | USFLI | 0.675 (0.566-0.773) | 69.5 | 67.5 | 31.1 | 91.3 | 40.955 | 0.004 |
|  | ZJU | 0.675 (0.560-0.791) | 60.1 | 73.1 | 32.1 | 89.7 | 50.714 | 0.013 |
|  | LAP | 0.656 (0.553-0.753) | 68.0 | 64.6 | 28.9 | 90.5 | 56.518 | 0.038 |
|  | HSI | 0.673 (0.552-0.784) | 59.1 | 66.5 | 27.2 | 88.5 | 47.086 | 0.010 |
|  | NHHR | 0.535 (0.413-0.658) | 68.7 | 45.1 | 20.9 | 87.2 | 2.274 | 0.329 |
|  | NFS | 0.595 (0.441-0.745) | 53.8 | 68.0 | 26.2 | 87.4 | 0.576 | 0.193 |
|  | FIB-4 | 0.538 (0.408-0.665) | 45.2 | 73.1 | 26.2 | 86.3 | 1.353 | 0.549 |
|  | BARD | 0.621 (0.495-0.732) | 78.4 | 50.5 | 25.1 | 91.7 | 2.459 | 0.097 |
| Non-Hispanic White | TyG | 0.526 (0.423-0.628) | 74.3 | 41.9 | 30.7 | 82.5 | 9.019 | 0.540 |
|  | TyG-BMI | 0.703 (0.603-0.786) | 64.8 | 69.8 | 42.7 | 85.1 | 323.264 | <0.001 |
|  | TyG-WC | 0.708 (0.613-0.802) | 71.8 | 61.9 | 39.6 | 86.4 | 1087.053 | <0.001 |
|  | TyG-WHtR | 0.704 (0.603-0.798) | 71.0 | 64.6 | 41.0 | 86.5 | 6.322 | <0.001 |
|  | TyG-WWI | 0.639 (0.527-0.741) | 67.2 | 60.9 | 37.4 | 84.3 | 109.205 | 0.030 |
|  | VAI | 0.477 (0.369-0.589) | 82.3 | 29.5 | 28.8 | 82.7 | 1.675 | 0.191 |
|  | HOMA-IR | 0.659 (0.554-0.752) | 61.8 | 67.9 | 40.1 | 83.7 | 7.143 | 0.032 |
|  | METS-IR | 0.692 (0.596-0.783) | 68.2 | 65.4 | 40.6 | 85.6 | 56.210 | <0.001 |
|  | FSI | 0.696 (0.601-0.787) | 71.8 | 63.4 | 40.5 | 86.6 | 0.253 | <0.001 |
|  | FLI | 0.743 (0.649-0.828) | 76.5 | 67.5 | 44.9 | 89.2 | 90.755 | <0.001 |
|  | USFLI | 0.722 (0.601-0.825) | 66.5 | 69.2 | 42.8 | 85.6 | 61.911 | 0.003 |
|  | ZJU | 0.694 (0.595-0.788) | 64.4 | 68.6 | 41.6 | 84.8 | 49.604 | <0.001 |
|  | LAP | 0.607 (0.513-0.714) | 66.4 | 51.4 | 32.1 | 81.5 | 77.927 | 0.009 |
|  | HSI | 0.707 (0.615-0.805) | 70.1 | 67.6 | 42.8 | 86.7 | 44.933 | 0.002 |
|  | NHHR | 0.519 (0.406-0.630) | 55.9 | 57.2 | 31.1 | 78.9 | 2.766 | 0.683 |
|  | NFS | 0.587 (0.482-0.691) | 66.6 | 48.9 | 31.1 | 80.8 | 0.160 | 0.009 |
|  | FIB-4 | 0.528 (0.429-0.633) | 52.1 | 53.8 | 28.1 | 76.4 | 1.241 | 0.382 |
|  | BARD | 0.570 (0.472-0.663) | 74.1 | 54.8 | 36.2 | 85.9 | 2.647 | 0.110 |
| Other Hispanic | TyG | 0.647 (0.487-0.804) | 93.9 | 45.3 | 41.9 | 94.6 | 8.800 | 0.143 |
|  | TyG-BMI | 0.888 (0.785-0.961) | 89.0 | 78.8 | 63.8 | 94.5 | 314.358 | <0.001 |
|  | TyG-WC | 0.860 (0.753-0.944) | 87.3 | 73.8 | 58.3 | 93.3 | 1031.300 | <0.001 |
|  | TyG-WHtR | 0.866 (0.754-0.955) | 76.4 | 87.5 | 72.1 | 89.8 | 6.552 | 0.016 |
|  | TyG-WWI | 0.747 (0.605-0.871) | 76.1 | 70.3 | 51.8 | 87.5 | 107.353 | 0.052 |
|  | VAI | 0.662 (0.484-0.830) | 61.1 | 61.4 | 39.9 | 79.0 | 2.035 | 0.090 |
|  | HOMA-IR | 0.235 (0.105-0.382) | 79.3 | 15.5 | 28.3 | 64.1 | 10.402 | 0.967 |
|  | METS-IR | 0.891 (0.789-0.960) | 82.9 | 83.0 | 67.2 | 92.0 | 56.873 | <0.001 |
|  | FSI | 0.854 (0.731-0.937) | 90.2 | 70.6 | 56.3 | 94.5 | 0.158 | <0.001 |
|  | FLI | 0.914 (0.821-0.978) | 86.7 | 87.1 | 73.9 | 94.0 | 94.242 | 0.029 |
|  | USFLI | 0.838 (0.710-0.944) | 92.3 | 82.3 | 68.7 | 96.2 | 66.862 | 0.026 |
|  | ZJU | 0.893 (0.807-0.961) | 86.4 | 82.0 | 66.9 | 93.5 | 49.324 | 0.001 |
|  | LAP | 0.780 (0.635-0.901) | 74.7 | 71.0 | 52.0 | 87.0 | 66.319 | 0.011 |
|  | HSI | 0.909 (0.820-0.971) | 86.6 | 85.2 | 71.0 | 93.8 | 48.686 | <0.001 |
|  | NHHR | 0.566 (0.350-0.748) | 45.0 | 86.7 | 58.8 | 79.0 | 3.592 | 0.248 |
|  | NFS | 0.689 (0.487-0.886) | 64.6 | 79.9 | 57.5 | 84.3 | 0.370 | 0.121 |
|  | FIB-4 | 0.576 (0.368-0.796) | 46.7 | 71.6 | 40.9 | 76.2 | 1.200 | 0.044 |
|  | BARD | 0.633 (0.465-0.780) | 60.2 | 71.7 | 47.2 | 81.1 | 2.771 | 0.241 |
| Other Race | TyG | 0.617 (0.441-0.769) | 69.9 | 51.1 | 20.2 | 90.6 | 8.942 | 0.131 |
|  | TyG-BMI | 0.670 (0.525-0.802) | 75.6 | 52.0 | 21.8 | 92.3 | 250.769 | 0.013 |
|  | TyG-WC | 0.679 (0.546-0.813) | 62.5 | 70.8 | 27.5 | 91.4 | 940.630 | 0.028 |
|  | TyG-WHtR | 0.660 (0.497-0.811) | 61.7 | 69.0 | 26.1 | 91.0 | 5.709 | 0.043 |
|  | TyG-WWI | 0.616 (0.443-0.776) | 58.5 | 70.1 | 25.8 | 90.5 | 108.410 | 0.256 |
|  | VAI | 0.609 (0.463-0.744) | 74.6 | 59.1 | 24.4 | 92.9 | 1.968 | 0.503 |
|  | HOMA-IR | 0.715 (0.533-0.879) | 67.7 | 80.3 | 37.8 | 93.3 | 7.476 | 0.155 |
|  | METS-IR | 0.679 (0.544-0.811) | 73.1 | 64.4 | 26.7 | 93.1 | 44.910 | 0.002 |
|  | FSI | 0.686 (0.545-0.802) | 65.5 | 70.3 | 28.1 | 92.0 | -0.541 | 0.003 |
|  | FLI | 0.721 (0.577-0.854) | 66.4 | 70.4 | 28.5 | 92.2 | 74.059 | 0.010 |
|  | USFLI | 0.766 (0.612-0.907) | 71.8 | 84.8 | 45.6 | 94.4 | 63.368 | 0.013 |
|  | ZJU | 0.662 (0.500-0.812) | 69.4 | 59.2 | 23.2 | 91.6 | 42.812 | 0.005 |
|  | LAP | 0.642 (0.501-0.776) | 70.9 | 65.0 | 26.4 | 92.6 | 60.766 | 0.130 |
|  | HSI | 0.656 (0.503-0.798) | 61.6 | 69.5 | 26.4 | 91.1 | 42.114 | 0.009 |
|  | NHHR | 0.575 (0.432-0.731) | 52.2 | 65.1 | 21.0 | 88.5 | 2.982 | 0.428 |
|  | NFS | 0.660 (0.499-0.805) | 58.1 | 74.9 | 29.1 | 91.0 | 0.128 | 0.071 |
|  | FIB-4 | 0.596 (0.438-0.767) | 74.0 | 53.5 | 22.0 | 92.1 | 1.072 | 0.089 |
|  | BARD | 0.670 (0.540-0.784) | 81.4 | 60.4 | 26.7 | 94.8 | 2.500 | 0.014 |
| Age ≤50 | TyG | 0.616 (0.480-0.730) | 61.3 | 63.5 | 36.8 | 82.5 | 9.092 | 0.109 |
|  | TyG-BMI | 0.728 (0.611-0.843) | 70.8 | 65.3 | 41.4 | 86.6 | 333.540 | 0.005 |
|  | TyG-WC | 0.762 (0.648-0.870) | 88.1 | 54.4 | 40.1 | 92.9 | 1031.300 | <0.001 |
|  | TyG-WHtR | 0.724 (0.598-0.839) | 83.1 | 57.3 | 40.3 | 90.7 | 6.155 | <0.001 |
|  | TyG-WWI | 0.669 (0.547-0.770) | 68.7 | 61.6 | 38.3 | 85.0 | 105.612 | 0.015 |
|  | VAI | 0.547 (0.414-0.695) | 71.7 | 45.0 | 31.1 | 82.1 | 2.044 | 0.353 |
|  | HOMA-IR | 0.726 (0.595-0.848) | 78.4 | 60.4 | 40.7 | 88.9 | 8.145 | 0.009 |
|  | METS-IR | 0.750 (0.635-0.871) | 66.7 | 74.1 | 47.2 | 86.5 | 61.850 | 0.003 |
|  | FSI | 0.737 (0.618-0.841) | 80.1 | 67.4 | 46.0 | 90.7 | 0.880 | 0.012 |
|  | FLI | 0.760 (0.629-0.887) | 78.3 | 65.2 | 43.8 | 89.6 | 93.941 | 0.098 |
|  | USFLI | 0.818 (0.698-0.903) | 82.2 | 74.4 | 52.7 | 92.3 | 70.645 | <0.001 |
|  | ZJU | 0.738 (0.613-0.852) | 86.3 | 59.5 | 42.5 | 92.6 | 50.711 | 0.004 |
|  | LAP | 0.618 (0.487-0.746) | 74.6 | 43.6 | 31.5 | 83.2 | 64.485 | 0.131 |
|  | HSI | 0.732 (0.596-0.847) | 71.2 | 70.4 | 45.5 | 87.6 | 50.629 | 0.052 |
|  | NHHR | 0.563 (0.423-0.718) | 59.3 | 63.2 | 35.9 | 81.7 | 3.516 | 0.650 |
|  | NFS | 0.636 (0.483-0.786) | 47.8 | 79.4 | 44.6 | 81.4 | 0.198 | 0.070 |
|  | FIB-4 | 0.616 (0.477-0.759) | 64.5 | 56.4 | 34.0 | 82.1 | 0.608 | 0.143 |
|  | BARD | 0.568 (0.434-0.703) | 70.8 | 58.7 | 37.3 | 85.3 | 2.576 | 0.364 |
| Age >50 | TyG | 0.551 (0.470-0.624) | 67.6 | 48.2 | 27.7 | 83.5 | 9.020 | 0.285 |
|  | TyG-BMI | 0.716 (0.645-0.782) | 68.5 | 66.8 | 37.7 | 87.8 | 302.218 | <0.001 |
|  | TyG-WC | 0.719 (0.649-0.779) | 61.2 | 73.6 | 40.6 | 86.6 | 1087.053 | <0.001 |
|  | TyG-WHtR | 0.719 (0.639-0.789) | 65.4 | 70.7 | 39.6 | 87.4 | 6.322 | <0.001 |
|  | TyG-WWI | 0.657 (0.564-0.735) | 64.3 | 64.3 | 34.6 | 86.0 | 109.071 | 0.002 |
|  | VAI | 0.527 (0.446-0.606) | 77.5 | 39.8 | 27.4 | 85.7 | 1.675 | 0.235 |
|  | HOMA-IR | 0.673 (0.585-0.760) | 60.5 | 70.9 | 37.9 | 85.9 | 6.641 | 0.029 |
|  | METS-IR | 0.701 (0.625-0.765) | 60.1 | 73.3 | 39.8 | 86.2 | 55.753 | <0.001 |
|  | FSI | 0.700 (0.625-0.771) | 69.3 | 65.9 | 37.4 | 87.9 | 0.158 | <0.001 |
|  | FLI | 0.755 (0.692-0.819) | 68.2 | 75.0 | 44.5 | 88.9 | 90.429 | <0.001 |
|  | USFLI | 0.730 (0.649-0.807) | 64.1 | 71.4 | 39.7 | 87.1 | 55.163 | <0.001 |
|  | ZJU | 0.704 (0.631-0.776) | 69.3 | 62.1 | 34.9 | 87.3 | 46.641 | <0.001 |
|  | LAP | 0.642 (0.568-0.713) | 72.4 | 56.1 | 32.7 | 87.4 | 69.265 | <0.001 |
|  | HSI | 0.713 (0.636-0.780) | 73.3 | 64.4 | 37.7 | 89.2 | 43.079 | <0.001 |
|  | NHHR | 0.497 (0.411-0.576) | 60.6 | 47.3 | 25.3 | 80.3 | 2.465 | 0.846 |
|  | NFS | 0.630 (0.554-0.707) | 60.1 | 58.4 | 29.8 | 83.3 | 0.370 | <0.001 |
|  | FIB-4 | 0.568 (0.489-0.653) | 59.4 | 52.3 | 26.8 | 81.4 | 1.271 | <0.001 |
|  | BARD | 0.615 (0.541-0.688) | 77.5 | 55.2 | 33.7 | 89.3 | 2.647 | <0.001 |
| Never-smoker | TyG | 0.480 (0.389-0.577) | 72.1 | 40.3 | 27.1 | 82.4 | 9.175 | 0.968 |
|  | TyG-BMI | 0.738 (0.661-0.808) | 70.3 | 66.3 | 39.1 | 87.9 | 298.069 | <0.001 |
|  | TyG-WC | 0.744 (0.663-0.822) | 79.2 | 60.3 | 38.1 | 90.4 | 995.998 | <0.001 |
|  | TyG-WHtR | 0.757 (0.666-0.835) | 68.7 | 74.6 | 45.5 | 88.6 | 6.224 | <0.001 |
|  | TyG-WWI | 0.671 (0.558-0.765) | 71.2 | 59.5 | 35.1 | 87.0 | 106.753 | 0.008 |
|  | VAI | 0.496 (0.414-0.584) | 73.5 | 43.2 | 28.5 | 84.1 | 2.245 | 0.573 |
|  | HOMA-IR | 0.668 (0.564-0.774) | 59.3 | 72.1 | 39.6 | 85.2 | 6.993 | 0.016 |
|  | METS-IR | 0.716 (0.626-0.793) | 68.0 | 64.9 | 37.4 | 86.8 | 52.526 | <0.001 |
|  | FSI | 0.704 (0.621-0.786) | 68.5 | 71.8 | 42.8 | 88.1 | 0.229 | <0.001 |
|  | FLI | 0.777 (0.705-0.846) | 67.8 | 76.4 | 47.0 | 88.5 | 90.698 | <0.001 |
|  | USFLI | 0.740 (0.642-0.834) | 60.5 | 78.6 | 46.6 | 86.6 | 55.163 | <0.001 |
|  | ZJU | 0.722 (0.628-0.803) | 61.7 | 72.8 | 41.1 | 86.0 | 49.066 | <0.001 |
|  | LAP | 0.627 (0.539-0.713) | 66.4 | 59.8 | 33.7 | 85.2 | 66.319 | 0.021 |
|  | HSI | 0.730 (0.635-0.822) | 82.4 | 62.2 | 40.2 | 92.0 | 43.079 | <0.001 |
|  | NHHR | 0.559 (0.450-0.666) | 70.9 | 43.2 | 27.8 | 82.8 | 2.775 | 0.494 |
|  | NFS | 0.640 (0.557-0.726) | 65.0 | 60.9 | 33.9 | 84.9 | 0.218 | <0.001 |
|  | FIB-4 | 0.549 (0.446-0.644) | 73.3 | 39.9 | 27.3 | 82.9 | 1.001 | 0.007 |
|  | BARD | 0.616 (0.523-0.697) | 78.2 | 56.0 | 35.4 | 89.3 | 2.522 | <0.001 |
| Former smoker | TyG | 0.615 (0.491-0.721) | 64.3 | 63.0 | 36.6 | 84.2 | 9.249 | 0.081 |
|  | TyG-BMI | 0.682 (0.572-0.784) | 71.5 | 60.9 | 37.8 | 86.5 | 317.755 | 0.003 |
|  | TyG-WC | 0.710 (0.601-0.812) | 53.3 | 80.3 | 47.3 | 83.8 | 1167.678 | <0.001 |
|  | TyG-WHtR | 0.665 (0.546-0.765) | 61.3 | 65.1 | 36.8 | 83.5 | 6.589 | 0.004 |
|  | TyG-WWI | 0.622 (0.507-0.733) | 70.5 | 52.4 | 33.0 | 84.2 | 107.423 | 0.077 |
|  | VAI | 0.586 (0.479-0.696) | 52.1 | 62.4 | 31.5 | 79.7 | 2.770 | 0.133 |
|  | HOMA-IR | 0.703 (0.593-0.799) | 71.4 | 65.3 | 40.6 | 87.3 | 7.166 | 0.009 |
|  | METS-IR | 0.687 (0.581-0.782) | 75.4 | 59.9 | 38.5 | 88.0 | 55.753 | <0.001 |
|  | FSI | 0.707 (0.594-0.793) | 67.2 | 63.2 | 37.8 | 85.3 | 0.676 | 0.008 |
|  | FLI | 0.711 (0.600-0.810) | 72.2 | 63.3 | 39.5 | 87.2 | 91.777 | 0.001 |
|  | USFLI | 0.771 (0.676-0.854) | 72.3 | 73.4 | 47.4 | 88.8 | 66.862 | <0.001 |
|  | ZJU | 0.682 (0.573-0.781) | 69.2 | 67.3 | 41.3 | 86.8 | 49.604 | 0.004 |
|  | LAP | 0.650 (0.539-0.758) | 65.0 | 57.6 | 33.8 | 83.2 | 88.257 | 0.063 |
|  | HSI | 0.665 (0.556-0.774) | 67.9 | 63.3 | 38.1 | 85.6 | 45.445 | 0.016 |
|  | NHHR | 0.606 (0.493-0.713) | 62.4 | 56.3 | 32.2 | 81.8 | 2.925 | 0.171 |
|  | NFS | 0.566 (0.440-0.701) | 55.0 | 61.8 | 32.4 | 80.5 | 0.902 | 0.340 |
|  | FIB-4 | 0.537 (0.406-0.663) | 65.7 | 55.8 | 33.1 | 83.0 | 1.259 | 0.154 |
|  | BARD | 0.577 (0.465-0.690) | 76.4 | 54.3 | 35.7 | 87.3 | 2.684 | 0.233 |
| Current smoker | TyG | 0.694 (0.533-0.839) | 60.0 | 72.0 | 32.0 | 89.1 | 9.244 | 0.036 |
|  | TyG-BMI | 0.756 (0.578-0.896) | 74.0 | 75.4 | 39.9 | 93.0 | 327.829 | 0.026 |
|  | TyG-WC | 0.766 (0.594-0.909) | 74.0 | 76.1 | 40.6 | 93.0 | 1130.596 | 0.025 |
|  | TyG-WHtR | 0.741 (0.559-0.896) | 79.8 | 70.1 | 37.0 | 94.0 | 6.493 | 0.045 |
|  | TyG-WWI | 0.675 (0.493-0.843) | 71.3 | 66.7 | 32.0 | 91.4 | 109.524 | 0.060 |
|  | VAI | 0.546 (0.379-0.730) | 70.0 | 47.3 | 22.6 | 87.8 | 2.044 | 0.107 |
|  | HOMA-IR | 0.678 (0.508-0.832) | 60.1 | 78.2 | 37.8 | 89.9 | 7.211 | 0.772 |
|  | METS-IR | 0.742 (0.551-0.888) | 68.2 | 79.1 | 41.7 | 91.9 | 61.686 | 0.046 |
|  | FSI | 0.773 (0.600-0.893) | 68.2 | 88.0 | 55.6 | 92.6 | 0.902 | 0.010 |
|  | FLI | 0.795 (0.627-0.934) | 80.6 | 79.3 | 46.1 | 94.9 | 94.554 | 0.082 |
|  | USFLI | 0.732 (0.576-0.873) | 72.1 | 73.8 | 37.7 | 92.3 | 65.075 | 0.021 |
|  | ZJU | 0.733 (0.569-0.882) | 70.6 | 71.2 | 35.0 | 91.7 | 51.904 | 0.017 |
|  | LAP | 0.676 (0.497-0.836) | 78.7 | 58.8 | 29.6 | 92.6 | 69.642 | 0.092 |
|  | HSI | 0.729 (0.546-0.883) | 74.0 | 77.9 | 42.4 | 93.2 | 48.200 | 0.077 |
|  | NHHR | 0.598 (0.384-0.797) | 51.7 | 79.8 | 36.0 | 88.2 | 3.520 | 0.206 |
|  | NFS | 0.739 (0.540-0.917) | 60.1 | 89.8 | 56.6 | 91.1 | 0.632 | 0.027 |
|  | FIB-4 | 0.589 (0.364-0.768) | 60.2 | 62.6 | 26.2 | 87.7 | 0.891 | 0.038 |
|  | BARD | 0.602 (0.402-0.778) | 68.6 | 54.5 | 24.9 | 88.7 | 2.603 | 0.119 |
| Non-Obese | TyG | 0.580 (0.419-0.723) | 69.5 | 55.0 | 13.4 | 94.7 | 9.019 | 0.262 |
|  | TyG-BMI | 0.668 (0.536-0.774) | 71.0 | 62.1 | 15.8 | 95.5 | 243.457 | 0.038 |
|  | TyG-WC | 0.672 (0.545-0.786) | 59.9 | 63.8 | 14.2 | 94.1 | 920.572 | 0.013 |
|  | TyG-WHtR | 0.645 (0.496-0.769) | 63.9 | 64.3 | 15.2 | 94.7 | 5.409 | 0.024 |
|  | TyG-WWI | 0.599 (0.455-0.737) | 66.9 | 49.2 | 11.7 | 93.7 | 101.235 | 0.089 |
|  | VAI | 0.603 (0.437-0.736) | 65.7 | 56.3 | 13.1 | 94.3 | 1.968 | 0.106 |
|  | HOMA-IR | 0.625 (0.475-0.756) | 61.7 | 69.6 | 16.9 | 94.8 | 5.144 | 0.705 |
|  | METS-IR | 0.684 (0.543-0.800) | 75.5 | 57.0 | 15.0 | 95.9 | 42.102 | 0.017 |
|  | FSI | 0.657 (0.504-0.778) | 55.2 | 74.5 | 17.8 | 94.3 | -0.961 | 0.028 |
|  | FLI | 0.758 (0.627-0.860) | 68.6 | 74.8 | 21.4 | 96.0 | 63.759 | 0.004 |
|  | USFLI | 0.712 (0.575-0.836) | 66.9 | 71.7 | 19.2 | 95.6 | 44.149 | 0.010 |
|  | ZJU | 0.618 (0.486-0.735) | 59.8 | 63.0 | 13.9 | 94.0 | 40.905 | 0.113 |
|  | LAP | 0.636 (0.489-0.762) | 62.9 | 63.4 | 14.7 | 94.5 | 56.518 | 0.049 |
|  | HSI | 0.612 (0.471-0.721) | 67.2 | 56.4 | 13.4 | 94.5 | 37.095 | 0.128 |
|  | NHHR | 0.550 (0.386-0.692) | 54.3 | 60.3 | 12.1 | 92.9 | 2.925 | 0.421 |
|  | NFS | 0.566 (0.449-0.709) | 42.3 | 68.7 | 11.9 | 92.2 | 0.128 | 0.194 |
|  | FIB-4 | 0.631 (0.498-0.779) | 70.8 | 53.5 | 13.2 | 94.8 | 1.317 | 0.013 |
|  | BARD | 0.654 (0.514-0.778) | 59.6 | 65.6 | 14.8 | 94.2 | 2.238 | 0.017 |
| Obese | TyG | 0.554 (0.477-0.635) | 62.5 | 49.8 | 37.1 | 73.7 | 9.060 | 0.130 |
|  | TyG-BMI | 0.615 (0.522-0.706) | 63.0 | 60.9 | 43.3 | 77.7 | 333.395 | 0.007 |
|  | TyG-WC | 0.637 (0.558-0.721) | 64.7 | 55.5 | 40.8 | 76.9 | 1105.631 | <0.001 |
|  | TyG-WHtR | 0.635 (0.548-0.722) | 62.5 | 59.3 | 42.1 | 77.0 | 6.649 | 0.009 |
|  | TyG-WWI | 0.613 (0.524-0.702) | 63.1 | 58.5 | 41.8 | 77.0 | 109.071 | 0.040 |
|  | VAI | 0.469 (0.386-0.555) | 59.5 | 39.6 | 31.8 | 67.4 | 2.031 | 0.797 |
|  | HOMA-IR | 0.643 (0.549-0.740) | 67.0 | 61.8 | 45.3 | 79.8 | 7.211 | 0.006 |
|  | METS-IR | 0.593 (0.501-0.686) | 73.8 | 46.4 | 39.4 | 78.9 | 56.541 | 0.015 |
|  | FSI | 0.611 (0.530-0.700) | 56.4 | 63.2 | 42.0 | 75.4 | 0.831 | 0.015 |
|  | FLI | 0.655 (0.568-0.745) | 60.5 | 65.8 | 45.6 | 77.9 | 94.235 | 0.003 |
|  | USFLI | 0.697 (0.607-0.788) | 68.4 | 63.5 | 47.0 | 81.0 | 63.368 | <0.001 |
|  | ZJU | 0.627 (0.537-0.715) | 63.1 | 60.7 | 43.2 | 77.7 | 50.613 | 0.008 |
|  | LAP | 0.534 (0.447-0.615) | 51.2 | 51.5 | 33.3 | 69.0 | 88.434 | 0.086 |
|  | HSI | 0.622 (0.536-0.711) | 60.2 | 62.2 | 42.9 | 76.7 | 48.830 | 0.021 |
|  | NHHR | 0.502 (0.416-0.588) | 65.2 | 41.4 | 34.5 | 71.6 | 3.047 | 0.897 |
|  | NFS | 0.588 (0.507-0.672) | 68.8 | 45.9 | 37.5 | 75.7 | 0.198 | 0.038 |
|  | FIB-4 | 0.589 (0.499-0.670) | 70.9 | 46.8 | 38.7 | 77.3 | 1.001 | 0.009 |
|  | BARD | 0.483 (0.404-0.568) | 73.4 | 39.2 | 36.3 | 75.7 | 2.684 | 0.619 |
| Non-Hypertension | TyG | 0.529 (0.389-0.663) | 58.0 | 46.0 | 19.0 | 83.3 | 9.175 | 0.554 |
|  | TyG-BMI | 0.714 (0.601-0.828) | 74.3 | 62.5 | 30.3 | 91.7 | 279.190 | 0.025 |
|  | TyG-WC | 0.760 (0.645-0.864) | 70.0 | 68.4 | 32.7 | 91.2 | 1024.136 | 0.001 |
|  | TyG-WHtR | 0.656 (0.509-0.799) | 67.2 | 69.5 | 32.5 | 90.6 | 6.224 | 0.061 |
|  | TyG-WWI | 0.529 (0.384-0.671) | 54.7 | 55.4 | 21.2 | 84.8 | 106.753 | 0.624 |
|  | VAI | 0.549 (0.429-0.668) | 76.8 | 42.6 | 22.7 | 89.4 | 2.447 | 0.307 |
|  | HOMA-IR | 0.698 (0.570-0.823) | 65.7 | 73.5 | 35.2 | 90.7 | 7.211 | 0.005 |
|  | METS-IR | 0.731 (0.614-0.847) | 88.3 | 55.7 | 30.4 | 95.6 | 45.346 | 0.028 |
|  | FSI | 0.699 (0.568-0.828) | 60.3 | 76.8 | 36.3 | 89.8 | 0.289 | 0.083 |
|  | FLI | 0.790 (0.664-0.901) | 71.2 | 74.5 | 38.0 | 92.2 | 90.770 | 0.006 |
|  | USFLI | 0.787 (0.654-0.914) | 69.3 | 83.1 | 47.3 | 92.5 | 70.224 | <0.001 |
|  | ZJU | 0.666 (0.534-0.803) | 68.2 | 64.4 | 29.5 | 90.2 | 46.851 | 0.085 |
|  | LAP | 0.599 (0.468-0.736) | 54.5 | 65.8 | 25.9 | 86.8 | 78.590 | 0.512 |
|  | HSI | 0.683 (0.518-0.830) | 64.8 | 75.5 | 36.7 | 90.7 | 44.933 | 0.048 |
|  | NHHR | 0.553 (0.408-0.683) | 74.8 | 45.3 | 23.0 | 89.1 | 3.094 | 0.305 |
|  | NFS | 0.637 (0.515-0.773) | 59.2 | 68.4 | 29.1 | 88.5 | 0.198 | 0.010 |
|  | FIB-4 | 0.616 (0.437-0.780) | 49.2 | 81.4 | 36.7 | 88.0 | 1.547 | 0.068 |
|  | BARD | 0.664 (0.545-0.773) | 77.6 | 63.3 | 31.7 | 92.8 | 2.522 | 0.011 |
| Hypertension | TyG | 0.598 (0.518-0.676) | 70.6 | 50.1 | 32.4 | 83.4 | 9.011 | 0.047 |
|  | TyG-BMI | 0.718 (0.649-0.781) | 69.6 | 70.1 | 44.1 | 87.2 | 317.755 | <0.001 |
|  | TyG-WC | 0.714 (0.645-0.781) | 62.6 | 69.4 | 41.0 | 84.5 | 1087.053 | <0.001 |
|  | TyG-WHtR | 0.735 (0.670-0.799) | 65.3 | 72.0 | 44.2 | 85.9 | 6.487 | <0.001 |
|  | TyG-WWI | 0.691 (0.612-0.757) | 62.6 | 69.2 | 40.8 | 84.5 | 109.205 | <0.001 |
|  | VAI | 0.556 (0.478-0.634) | 81.0 | 38.8 | 31.0 | 85.8 | 1.678 | 0.011 |
|  | HOMA-IR | 0.683 (0.595-0.762) | 63.4 | 69.5 | 41.4 | 84.8 | 6.993 | 0.009 |
|  | METS-IR | 0.702 (0.625-0.766) | 70.8 | 67.2 | 42.3 | 87.1 | 56.060 | <0.001 |
|  | FSI | 0.707 (0.639-0.777) | 74.4 | 60.7 | 39.1 | 87.5 | 0.223 | <0.001 |
|  | FLI | 0.740 (0.672-0.802) | 71.4 | 70.3 | 44.9 | 87.9 | 90.429 | <0.001 |
|  | USFLI | 0.740 (0.652-0.812) | 65.1 | 75.3 | 47.3 | 86.4 | 61.911 | <0.001 |
|  | ZJU | 0.718 (0.644-0.785) | 67.4 | 69.7 | 43.0 | 86.3 | 49.604 | <0.001 |
|  | LAP | 0.645 (0.571-0.716) | 75.5 | 52.9 | 35.2 | 86.4 | 69.265 | <0.001 |
|  | HSI | 0.710 (0.639-0.779) | 70.3 | 64.7 | 40.3 | 86.5 | 45.348 | <0.001 |
|  | NHHR | 0.539 (0.450-0.624) | 53.7 | 57.0 | 29.8 | 78.4 | 2.766 | 0.068 |
|  | NFS | 0.618 (0.534-0.697) | 60.9 | 58.6 | 33.3 | 81.5 | 0.370 | 0.005 |
|  | FIB-4 | 0.541 (0.457-0.621) | 50.5 | 58.9 | 29.5 | 77.8 | 1.241 | 0.003 |
|  | BARD | 0.575 (0.499-0.653) | 78.3 | 49.6 | 34.5 | 87.1 | 2.576 | 0.036 |

T2DM, type 2 diabetes mellitus; TyG: triglyceride-glucose index; BMI: body mass index; WC: waist circumference; WHtR: waist-to-height ratio; WWI: weight-adjusted waist index; VAI: visceral adiposity index; HOMA-IR: homeostatic model assessment of insulin resistance; METS-IR: metabolic score for insulin resistance; FSI: Framingham steatosis index; FLI: fatty liver index; ZJU: Zhejiang University index; LAP: lipid accumulation product; HSI: hepatic steatosis index; NHHR: non-high-density lipoprotein cholesterol (HDL-C) to HDL-C ratio; NFS: nonalcoholic fatty liver disease fibrosis score; FIB-4: fibrosis-4 index; BARD: BMI-aspartate aminotransferase/alanine aminotransferase ratio and diabetes score

**Supplementary References**

1. Simental-Mendía LE, Rodríguez-Morán M, Guerrero-Romero F. The Product of Fasting Glucose and Triglycerides As Surrogate for Identifying Insulin Resistance in Apparently Healthy Subjects. *Metabolic Syndrome and Related Disorders*. 2008/12/01 2008;6(4):299-304. doi:10.1089/met.2008.0034

2. Zheng S, Shi S, Ren X, et al. Triglyceride glucose-waist circumference, a novel and effective predictor of diabetes in first-degree relatives of type 2 diabetes patients: cross-sectional and prospective cohort study. *Journal of translational medicine*. Sep 7 2016;14(1):260. doi:10.1186/s12967-016-1020-8

3. Er LK, Wu S, Chou HH, et al. Triglyceride Glucose-Body Mass Index Is a Simple and Clinically Useful Surrogate Marker for Insulin Resistance in Nondiabetic Individuals. *PloS one*. 2016;11(3):e0149731. doi:10.1371/journal.pone.0149731

4. Lim J, Kim J, Koo SH, Kwon GC. Comparison of triglyceride glucose index, and related parameters to predict insulin resistance in Korean adults: An analysis of the 2007-2010 Korean National Health and Nutrition Examination Survey. *PloS one*. 2019;14(3):e0212963. doi:10.1371/journal.pone.0212963

5. Park Y, Kim NH, Kwon TY, Kim SG. A novel adiposity index as an integrated predictor of cardiometabolic disease morbidity and mortality. *Sci Rep*. Nov 13 2018;8(1):16753. doi:10.1038/s41598-018-35073-4

6. Harrison SA, Oliver D, Arnold HL, Gogia S, Neuschwander-Tetri BA. Development and validation of a simple NAFLD clinical scoring system for identifying patients without advanced disease. *Gut*. Oct 2008;57(10):1441-7. doi:10.1136/gut.2007.146019

7. Amato MC, Giordano C, Galia M, et al. Visceral Adiposity Index: A reliable indicator of visceral fat function associated with cardiometabolic risk. *Diabetes Care*. 2010;33(4):920-922. doi:10.2337/dc09-1825 %J Diabetes Care

8. Katsuki A, Sumida Y, Gabazza EC, et al. Homeostasis model assessment is a reliable indicator of insulin resistance during follow-up of patients with type 2 diabetes. *Diabetes Care*. Feb 2001;24(2):362-5. doi:10.2337/diacare.24.2.362

9. Bello-Chavolla OY, Almeda-Valdes P, Gomez-Velasco D, et al. METS-IR, a novel score to evaluate insulin sensitivity, is predictive of visceral adiposity and incident type 2 diabetes. *Eur J Endocrinol*. May 2018;178(5):533-544. doi:10.1530/eje-17-0883

10. Long MT, Pedley A, Colantonio LD, et al. Development and Validation of the Framingham Steatosis Index to Identify Persons With Hepatic Steatosis. *Clinical Gastroenterology and Hepatology*. 2016/08/01/ 2016;14(8):1172-1180.e2. doi:<https://doi.org/10.1016/j.cgh.2016.03.034>

11. Bedogni G, Bellentani S, Miglioli L, et al. The Fatty Liver Index: a simple and accurate predictor of hepatic steatosis in the general population. *BMC Gastroenterology*. 2006/11/02 2006;6(1):33. doi:10.1186/1471-230X-6-33

12. Ruhl CE, Everhart JE. Fatty liver indices in the multiethnic United States National Health and Nutrition Examination Survey. *Aliment Pharmacol Ther*. Jan 2015;41(1):65-76. doi:10.1111/apt.13012

13. Wang J, Xu C, Xun Y, et al. ZJU index: a novel model for predicting nonalcoholic fatty liver disease in a Chinese population. *Scientific reports*. 2015;5:16494. doi:10.1038/srep16494

14. Kahn HS. The lipid accumulation product is better than BMI for identifying diabetes: a population-based comparison. *Diabetes Care*. Jan 2006;29(1):151-3. doi:10.2337/diacare.29.1.151

15. Lee J, Kim D, Kim H, et al. Hepatic steatosis index: a simple screening tool reflecting nonalcoholic fatty liver disease. *Digestive and Liver Disease*. 2010;42(7):503-8. doi:10.1016/j.dld.2009.08.002

16. Angulo P, Hui JM, Marchesini G, et al. The NAFLD fibrosis score: a noninvasive system that identifies liver fibrosis in patients with NAFLD. *Hepatology*. Apr 2007;45(4):846-54. doi:10.1002/hep.21496

17. Sterling RK, Lissen E, Clumeck N, et al. Development of a simple noninvasive index to predict significant fibrosis in patients with HIV/HCV coinfection. *Hepatology*. Jun 2006;43(6):1317-25. doi:10.1002/hep.21178

18. Hou K, Song W, He J, Ma Z. The association between non-high-density lipoprotein cholesterol to high-density lipoprotein cholesterol ratio (NHHR) and prevalence of periodontitis among US adults: a cross-sectional NHANES study. *Sci Rep*. Mar 6 2024;14(1):5558. doi:10.1038/s41598-024-56276-y
